# Efficacy of inhaled nebulised unfractionated heparin to prevent intubation or death in hospitalised patients with COVID-19: an investigator-initiated international meta-trial of randomised clinical studies

**DOI:** 10.1016/j.eclinm.2025.103339

**Published:** 2025-09-27

**Authors:** Frank M.P. van Haren, Sarah J. Valle, Ary Serpa Neto, Marcus J. Schultz, John G. Laffey, Antonio Artigas, Barry Dixon, Alicia B. Vilaseca, Ruben A. Barbera, Tarek I. Ismail, Rabab S. Mahrous, Mohamed Badr, Gilberto DeNucci, Carlos Sverdloff, Marta Camprubi-Rimblas, David W. Cosgrave, Bairbre McNicholas, Catriona Cody, Gerard Curley, Thomas L. Smoot, Sabrina Staas, Khine Sann, Caitlin Sas, Anusha Belani, Christopher Hillman, Sidharta Kusuma Manggala, Dita Aditianingsih, Adhrie Sugiarto, Mira Yulianti, Herikurniawan Herikurniawan, Robert Sinto, Aino Nindya Auerkari, Septian Adi Permana, Ashley Woodcock, Mary Carroll, Tom Wilkinson, Dave Singh, Janis Kay Shute, Miles Carroll, Clive Page

**Affiliations:** a Australian National University, College of Health and Medicine, Canberra, Australia; b Intensive Care Unit, St George Hospital, Sydney, Australia; c Australian and New Zealand Intensive Care Research Centre, Monash University, Melbourne, Australia; d Department of Intensive Care, Austin Hospital, Melbourne, Australia; e Department of Intensive Care, Amsterdam UMC, Amsterdam, the Netherlands; f Mahidol Oxford Tropical Medicine Research Unit, Mahidol University, Bangkok, Thailand; g Anaesthesia and Intensive Care Medicine, School of Medicine, University of Galway, Galway, Ireland; h Department of Anaesthesia and Intensive Care Medicine, University Hospital Galway, Ireland; i Critical Care Center, Corporació Sanitaria Universitaria Parc Tauli, Institut d'Investigació i Innovació Parc Tauli (I3PT CERCA), CIBER Enfermedades Respiratorias, Autonomous University of Barcelona, Sabadell, Spain; j Department of Critical Care Medicine, St Vincent’s Hospital, Melbourne, Australia; k Service of Haematology and Haemostasis, San Camilo Clinic, Buenos Aires, Argentina; l Department of Anaesthesia and Surgical Intensive Care, Faculty of Medicine, Helwan University, Cairo, Egypt; m Department of Anaesthesia and Surgical Intensive Care, Faculty of Medicine, Alexandria University, Alexandria, Egypt; n Department of Critical Care Medicine, Faculty of Medicine, Helwan University, Cairo, Egypt; o Department of Pharmacology, Faculty of Medical Sciences, University of Campinas, Campinas, Brazil; p Department of Pharmacology, Institute of Biomedical Sciences, University of São Paulo, Brazil; q ACTGen, Brazil; r Department of Anaesthesia and Intensive Care Medicine, Connolly Memorial Hospital Blanchardstown, Dublin, Ireland; s Department of Anaesthesia and Intensive Care Medicine, Royal College of Surgeons in Ireland, Beaumont Hospital, Dublin, Ireland; t Frederick Memorial Hospital, Frederick, MD, USA; u RSUP Dr Cipto Mangunkusumo, Jakarta, Indonesia; v RS Universitas Indonesia, Depok, Indonesia; w RSUD Dr Moewardi, Surakarta, Indonesia; x Division of Immunology, Immunity to Infection & Respiratory Medicine, University of Manchester, Manchester, United Kingdom; y Department of Respiratory Medicine, University of Southampton, Southampton, United Kingdom; z Centre for Human Genetics & Pandemic Sciences Institute, Nuffield Dept of Medicine, University of Oxford, Oxford, United Kingdom; aa School of Pharmacy and Biomedical Science, University of Portsmouth, Portsmouth, United Kingdom; ab Medicines Evaluation Unit, University of Manchester, Manchester, United Kingdom; ac Unit of Pulmonary Pharmacology, Institute of Pharmaceutical Science, King's College London, London, United Kingdom

**Keywords:** COVID-19, ARDS, Inhaled heparin, Nebulised heparin, Unfractionated heparin, SARS-CoV-2, Respiratory failure, Pandemic, Randomised trial, Meta-trial, Intubation, Mortality

## Abstract

**Background:**

Inhaled nebulised unfractionated heparin (UFH) has a strong rationale as a treatment for severe respiratory infections, including COVID-19, due to its antiviral, anti-inflammatory, and anti-coagulant properties, which may prevent viral entry, lung injury progression, and pulmonary thrombosis. We aimed to evaluate the efficacy of inhaled nebulised UFH to prevent intubation or death in hospitalised COVID-19 patients.

**Methods:**

In this prospective, a priori set up and defined, collaborative meta-trial of six randomised clinical studies, adult hospitalised but not intubated COVID-19 patients were randomly assigned to inhaled nebulised UFH on top of standard of care or standard of care alone. The dose and method of nebulisation was specific to each study. The primary outcome was intubation or death, assessed at the longest follow-up after randomisation. The meta-trial was registered at ClinicalTrials.gov ID NCT04635241.

**Findings:**

Between June 2020 and December 2022, 478 patients from 10 hospitals in six countries (Argentina, Brazil, Egypt, Indonesia, Ireland and USA) were enrolled. The odds ratio (OR) for intubation or death was 0.43 (0.26–0.73, p = 0.001); the OR for in-hospital mortality was 0.26 (0.13–0.54, p < 0.001) with inhaled nebulised UFH compared to standard of care alone. There were no safety issues reported, including no instances of pulmonary or systemic bleeding in the nebulised UFH group.

**Interpretation:**

In patients hospitalised but not intubated for COVID-19, inhaled nebulised UFH prevented intubation and reduced mortality, without causing pulmonary or systemic bleeding.

**Funding:**

The Sponsor of the meta-trial was the INHALE-HEP Collaborative Research Group (CRG); investigators of each contributing study were members of the INHALE-HEP CRG. No funding was received for the meta-trial. The Brazilian study was funded by The J.R. Moulton Charity Trust. The Irish study was funded by a Grant from 10.13039/501100001602Science Foundation Ireland to Cúram, the SFI's Centre for Research in Medical Devices (Research Centre Award Reference: 13/RC/2073).

**Research in context**

**Evidence before this study**

Inhaled nebulised unfractionated heparin (UFH) has been shown to improve outcomes and has been shown to be safe in experimental and clinical studies of acute lung injury and ARDS. UFH has antiviral properties against SARS-CoV-2 and other respiratory viruses, as well as anti-inflammatory and anticoagulant effects. Inhaled nebulised UFH has been shown to improve oxygenation parameters in an uncontrolled study of hospitalised COVID-19 patients. Whether these improvements translate into a reduced need for intubation or reduced mortality of hospitalised COVID-19 patients remains unknown.

We searched MEDLINE, EMBASE, and PubMed, Web of Science, Scopus, medRxiv, bioRxiv, and ClinicalTrials.gov for completed randomised clinical studies published to June 2020, published in any language, evaluating the effect of inhaled nebulised UFH to treat hospitalised non-intubated patients with COVID-19. The keywords (“heparin” OR “hep∗”) AND (“COVID-19” OR “SARS” OR “coronavirus”) AND (“awake” OR “non-intubated” OR “conscious”) were used to search the databases. We did not identify any completed published randomised clinical studies of inhaled nebulised UFH in hospitalised COVID-19 patients.

**Added value of this study**

This prospective meta-trial of six randomised clinical studies in low-, middle-, and high-income countries, shows that inhaled nebulised UFH significantly reduces the rate of intubation and mortality in hospitalised non-intubated COVID-19 patients.

We also showed that inhaled nebulised UFH is safe, with no pulmonary or systemic bleeding complications, and no clinically relevant effect on systemic coagulation parameters.

**Implications of all the available evidence**

The positive results from this study hold significant implications for the future treatment of lung injury, not only in the context of COVID-19 but also potentially for other infectious and non-infectious causes. Robust clinical trials evaluating inhaled nebulised UFH in hospitalised patients with lung injury, particularly those resulting from viral and bacterial respiratory infections, are now needed.

## Introduction

In December 2019, a novel coronavirus (severe acute respiratory syndrome coronavirus 2, SARS-CoV-2) emerged in China and has since spread globally. During the first pandemic wave, nearly 20% of patients with coronavirus disease 2019 (COVID-19) experienced hypoxaemia, which was the primary reason for hospitalisation.^1^ A significant proportion of patients admitted to hospital for COVID-19 developed acute respiratory failure, with 12–24% requiring intubation for invasive mechanical ventilation.^2^, ^3^, ^4^, ^5^, ^6^ Subsequent waves of COVID-19 involving different strains of SARS-CoV-2 and increased levels of herd immunity, were associated with lower rates of hospitalisation, acute respiratory failure and death, and although the mortality of intubated patients with COVID-19 did not improve over time, there was an initial decline early in the pandemic from extremely high rates, later stabilising at a still disappointingly high level.^7^, ^8^, ^9^

The pathophysiology of COVID-19 associated lung injury is characterised by diffuse alveolar damage, hyperinflammation, coagulopathy, DNA neutrophil extracellular traps (NETS), hyaline membranes and microvascular thrombosis.^10^^,^^11^ Our group and others have previously outlined the scientific rationale for the investigation of inhaled nebulised UFH as a treatment for COVID-19.^12^^,^^13^ Inhaled nebulised UFH has antiviral and anti-inflammatory properties, as well as effects on the pulmonary coagulation cascades. Previous preclinical and clinical studies of inhaled nebulised UFH in lung injury, have shown a positive effect on pulmonary coagulation, inflammation, oxygenation and other lung injury parameters.^12^

We conducted a prospective meta-trial, which involved pooling of individual patient data from randomised clinical studies initiated in seven different countries, that compared inhaled nebulised UFH on top of standard care with standard care alone in patients hospitalised but not yet intubated for COVID-19. We hypothesised that inhaled nebulised UFH prevents intubation and reduces mortality.

## Methods

### Study design, setting and patients

Prospective meta-trial of investigator initiated randomised clinical studies of inhaled nebulised UFH in hospitalised patients with COVID-19 who did not immediately require invasive mechanical ventilation. The protocol for this meta-trial was previously published and is available along with each individual trial protocol in the appendix.^14^

We prospectively planned to combine multiple trials that were not originally configured as a network of sites. All respective study teams agreed for their study to be included in the meta-trial, and data sharing agreements that included a meta-trial governance document, were executed before the data was shared. Individual studies were eligible to be included in this meta-trial if they were prospective randomised studies, with an intervention arm (inhaled nebulised UFH) and a control arm (standard care or placebo). Patients could be included in the individual studies if they were 18 years or older, admitted to hospital for COVID-19 with a PCR-positive sample for SARS-CoV-2 within the past 21 days. Patients were excluded if they were receiving invasive mechanical ventilation, or requiring immediate intubation as per the treating clinician's assessment; had heparin allergy or heparin-induced thrombocytopenia; had APTT >120 s, not due to anticoagulant therapy, or platelet count <20 × 109/L; had pulmonary bleeding or uncontrolled bleeding; were pregnant or might be pregnant; had acute brain injury that may result in long-term disability, myopathy, spinal cord injury, or nerve injury or disease with a likely prolonged incapacity to breathe independently e.g., Guillain-Barre syndrome; had treatment limitations in place, i.e., not for intubation, not for ICU admission; or when death was imminent or inevitable within 24 h.

We prospectively enrolled 7 studies into the meta-trial from the following 7 countries: Argentina, Australia, Brazil, Egypt, Indonesia, Ireland and USA. The studies were conducted at the following 10 hospitals: San Camilo Clinic, Buenos Aires (Argentina), Sao Roque Hospital and Santa Casa de Sorocaba Hospital, Sao Paulo (Brazil), 15th May Hospital, Cairo (Egypt), RSUP Dr Cipto Mangunkusumo, Jakarta, RS Universitas Indonesia, Depok, and RSUD Dr Moewardi, Surakarta (Indonesia), University Hospital Galway, Galway, Connolly Memorial Hospital, Blanchardstown, Dublin, and Beaumont Hospital, Dublin (Ireland), Frederick Health Hospital, Baltimore (USA). The Australian multicentre study failed to recruit any patients because of administrative delays in getting the study up and running before the end of 2023 and is therefore excluded from this meta-trial. Each individual trial was approved by the relevant local ethics committees. The individual study protocols outlined the process and requirements for obtaining patients' consent to participate in their study and as required by local laws and regulations. The meta-trial was supervised by an international steering committee.

### Interventions

All contributing investigations were randomised clinical studies. At randomisation each participant was assigned to nebulised UFH on top of standard care or standard care alone in a one-to-one allocation ratio. All contributing studies were open label by design except for the USA study, which was placebo controlled and blinded.

Participants assigned to “nebulised UFH” received nebulised UFH in addition to the standard care required as determined by the treating team. The dose, frequency, duration and delivery method differed between the protocols of participating studies as follows:

• Argentina: 5000 IU UFH every 8 h for 7 days using a Venturi system connected to a full-face mask (Free Breath) fitted with an HMF anti-viral expiratory filter.
• Brazil and USA: 25,000 IU UFH in 5 ml every 6 h using a vibrating mesh nebuliser, for a maximum of 21 days or until the WHO MOCS was 1 or 2.
• Egypt: 1000 IU/kg predicted body weight UFH every 6 h for 7 days using a compressed air nebuliser (Beurer IH18).
• Indonesia: 25,000 IU UFH every 8 h using a standard jet nebuliser, for a maximum of 21 days or until symptoms resolved.
• Ireland: 25,000 IU UFH in 5 ml every 6 h using a vibrating mesh nebuliser, for a maximum of 10 days.

Participants assigned to “control” received the standard care required as determined by the treating team and did not receive nebulised heparin. The USA study was placebo controlled, and participants assigned to “control” received placebo (in addition to the standard care), which was inhaled nebulised sodium chloride 0.9% (5 ml every 6 h).

### Endpoints

The primary outcome for the meta-trial was intubation or death (to include patients who died but were never intubated) at 28 days after randomisation. Because of trial heterogeneity, we decided to report intubation or death at the longest follow up after randomisation. Secondary outcomes were hospital mortality, 28-day mortality, and progression or resolution of COVID-19 assessed by the WHO modified ordinal clinical score at day 7 (MOCS, Table 1). The WHO MOCS is based on the WHO Clinical Progression Scale, which was designed to provide a standardised common set of outcome parameters for studies involving patients with COVID-19.^15^

**Table 1:** WHO modified ordinal clinical scale (MOCS) for COVID-19.

Modified Ordinal Clinical Scale
1. Not hospitalised
2. Hospitalised, not requiring supplemental oxygen and no longer requiring medical care for COVID-19
3. Hospitalised not requiring supplemental oxygen but needing medical care for COVID-19
4. Hospitalised requiring supplemental oxygen
5. Hospitalised requiring non-invasive ventilation or high flow oxygen
6. Hospitalised requiring intubation and mechanical ventilation or ECMO
7. Death

COVID-19, Coronavirus Disease 2019; ECMO, Extracorporeal Membrane Oxygenation.

Individual studies have included other primary and secondary outcomes, as listed in the individual study protocols (appendix).

Treatment with any of the following therapies which patients received at any point in time while in the study, was collected if available: systemic UFH or low molecular weight heparin (LMWH); remdesivir; oseltamivir; tocilizumab; antibiotics; antifungals; corticosteroid therapy; convalescent plasma.

The safety outcomes included the number of patients with complications: major systemic bleeding, pulmonary bleeding, heparin-induced thrombocytopenia (HIT), prolongation of aPTT, and any other adverse events and reactions that were not part of the expected clinical course and could be related to the study and were medically significant or had serious sequelae for the patient. Major systemic bleeding was defined as bleeding that results in death and/or bleeding that is symptomatic and occurs in a critical area or organ (intracranial, intraspinal, intra-ocular, retroperitoneal, intra-articular or intramuscular with compartment syndrome) and/or bleeding that results in a decrease in haemoglobin of 20 g/L or more, or results in transfusion of 2 or more units of whole blood or red cells. Pulmonary bleeding was defined as frank bleeding in the lungs, trachea or bronchi with repeated haemoptysis or requiring repeated suctioning and associated with acute deterioration in respiratory status.

### Statistical analysis

The term “meta-trial” refers to a prospective pooled analysis of individual de-identified patient-level data from multiple individual trials.^16^^,^^17^ Due to the meta-trial design, we used multilevel modelling (patients nested in trials), with trial as a random effect, along with testing the effect of other covariates as collected in the common variable set. We performed a one-stage individual patient data meta-analysis of the pooled data, as well as a two-stage individual patient data meta-analysis of study data. As an additional post-hoc sensitivity analysis, we also assessed the primary outcome after excluding data from the only randomised controlled study (USA).

Continuous data is reported as median (quartile 25th–quartile 75th) and compared using Wilcoxon rank-sum tests. Categorical data is reported using number and percentages and compared using Fisher exact test. Binary outcomes were compared between groups using a mixed effect generalised linear model with binomial distribution, including trials as random effect and reported as an odds ratio and its 95% confidence interval (CI). Continuous outcomes were compared using a median regression, including trials as cluster effect and reported as median difference and its 95% CI. Time-to-event outcomes are reported in Kaplan–Meier curves and compared using log-rank tests. Difference in hospital length of stay was assessed using Fine–Gray competing risk models with death before discharge treated as a competing event and including study as clustering.

Analyses were performed by intention-to-treat according to the participants’ randomly allocated group, regardless of treatment compliance. Missing data or data that were not collected by individual studies, were not imputed. The multilevel models described in the analysis can handle missing data due to loss to follow-up. Where there were missing observations, the number of observations used was reported. Two-sided hypothesis testing at a significance level of 0.05 were used. No adjustment for multiple tests was made, with the interpretation of the significance of the tests being appropriate for the primary or secondary nature of the outcome. Analyses were conducted using “R” version 4.3.3.

We calculated a sample size of 712 patients to demonstrate a clinically important reduction in the primary outcome, assuming a decrease in the proportion of patients receiving invasive mechanical ventilation from 12% to 6%, with power 80% and a two-sided significance level of 0.05.

### Ethics and registration

The Argentinian study protocol was approved by the Independent Ethics Committee for Clinical Pharmacology Trials, Buenos Aires (ID N 3183). The Australian protocol was approved by the Southeastern Sydney Local Health District HREC (2021/ETH11368). The Brazilian study protocol was approved by the Institute of Biomedical Sciences (ICB) Ethics Committee, Sao Paulo (ID 38660320.0.0000.5467). The Egyptian study protocol was approved by the Ethics committee, Faculty of Medicine, Alexandria University (ID 2158_11456_4737). The Indonesian protocol was approved by the Ethics Committee of the Faculty of Medicine, University of Indonesia–Cipto Mangunkusumo Hospital (KET- 115/UN2.F1/ETIK/PPM.00.02/2022). The Irish study protocol was approved by the National Research Ethics Committee for COVID-19-related Health Research (20-NREC-COV-104). The USA study protocol was approved by the Frederick Health Institutional Review Board.

Contributing studies were registered individually as follows: NCT04530578 (Argentina), NCT05184101 (Australia), UTN U1111-1263-3136 (Brazil), PACTR202007606032743 (Egypt), EudraCT 2020-003349-12 (Ireland), NCT04723563 (USA). The Indonesian study was not independently registered. The INHALE HEP meta-trial was registered on ClinicalTrials.gov NCT04635241.

### Role of the funding source

No funding was obtained for the meta-trial. The funders of the Brazilian and Irish studies had no role in study design, data collection, data analysis, data interpretation, or writing of the meta-trial report. The Sponsor of the meta-trial was the INHALE-HEP Collaborative Research Group (CRG), of which investigators of each contributing study were members. The INHALE-HEP CRG’s executive committee was responsible for the meta-trial’s study design; collection, management, analysis, and interpretation of data; writing and submitting the report for publication. Investigators from individual trials retained ownership of their study data. A data sharing agreement between investigators facilitated and governed the collecting and analysing of de-identified individual patient data from individual studies and set out eligibility for authorship.

## Results

### Patients

**Fig. 1:**
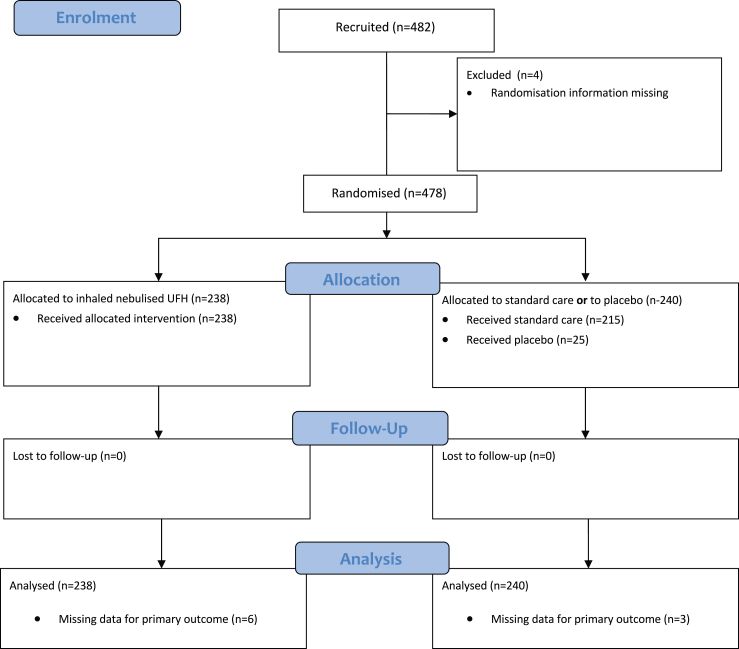
CONSORT diagram of participants in the INHALE-HEP meta-trial.

Between June 2020 and December 2022, 478 patients were recruited in the contributing studies (Fig. 1). At the end of 2023, when no patients had been enrolled for 12 consecutive months and the study in Australia failed to recruit patients, a decision was made to stop further recruitment attempts and collect all data for the meta-trial. Patients were recruited in Argentina (n = 183, 38%), Brazil (n = 76, 16%), Egypt (n = 100, 21%), Indonesia (n = 43, 9%), Ireland (n = 26, 5%), USA (n = 50, 10%). Baseline demographic and disease characteristics were well balanced between the two groups of the meta-trial (Table 2), and in each individual randomised controlled trial (Supplemental Table S1). The longest follow-up of patients was 28 days in all studies, except in the USA study (60 days) and the Ireland study (follow-up until hospital discharge, Supplemental Table S1). Median age was 54 [45–65] years and the majority were male. Just under one-third of all patients received supplemental oxygen (WHO MOCS 4) and a further 25% of patients received high flow nasal oxygen or non-invasive ventilation (WHO MOCS 5), without differences in the baseline WHO MOCS between the groups. Other COVID-19 therapies which patients received at any time point during the study including corticosteroids (79% of patients) and antiviral treatment with remdesivir (25% of patients), reflected the standard care in participating centres at the time the studies were conducted and were well balanced between the groups. Variables that were not collected in some studies, or that were missing in some patients, are summarised in Supplemental Table S2.

**Table 2:** Baseline and intervention characteristics of included patients.

	Overall (*n* = 478)	Nebulised heparin (*n* = 238)	Control group (*n* = 240)
Age, years	54.0 (45.0–65.0)	54.0 (45.0–66.0)	53.5 (45.0–63.0)
Male sex—no. (%)	293 (61.4)	139 (58.6)	154 (64.2)
From home—no. (%)	92 (100.0)	45 (100.0)	47 (100.0)
Body mass index, kg/m^2^	27.0 (23.8–30.8)	26.0 (23.5–30.3)	27.0 (24.0–31.0)
Co-morbidities—no. (%)			
Smoking	32 (12.8)	20 (15.2)	12 (10.2)
Asthma or COPD	26 (7.9)	12 (7.1)	14 (8.8)
Hypertension	81 (32.0)	43 (32.6)	38 (31.4)
Cardiac disease	16 (10.4)	7 (9.0)	9 (11.8)
Diabetes	60 (23.7)	27 (20.5)	33 (27.3)
Chronic kidney disease	8 (5.2)	2 (2.6)	6 (7.9)
COVID-19 therapies—no. (%)			
Corticosteroids	373 (78.5)	192 (81.7)	181 (75.4)
Tocilizumab	14 (28.0)	4 (16.0)	10 (40.0)
Remdesivir	68 (24.9)	38 (28.4)	30 (21.6)
Macrolides	158 (70.9)	81 (74.3)	77 (67.5)
Convalescent plasma	13 (2.7)	9 (3.8)	4 (1.7)
Non-COVID-19 therapies—no. (%)			
Oseltamivir	9 (4.0)	7 (6.4)	2 (1.8)
Antibiotics	389 (82.9)	195 (84.4)	194 (81.5)
Antifungal	9 (3.6)	4 (3.3)	5 (3.9)
IV or SC heparin	376 (84.3)	188 (86.2)	188 (82.5)
Baseline WHO MOCS			
Not hospitalised	0 (0.0)	0 (0.0)	0 (0.0)
Hospitalised, no oxygen, no COVID-19 care	12 (2.9)	2 (0.9)	10 (4.8)
Hospitalised, no oxygen, COVID-19 care	170 (40.6)	86 (40.8)	84 (40.4)
Hospitalised, oxygen	133 (31.7)	72 (34.1)	61 (29.3)
Hospitalised, NIV or HFNO	104 (24.8)	51 (24.2)	53 (25.5)
Hospitalised, IMV or ECMO	0 (0.0)	0 (0.0)	0 (0.0)
Death	0 (0.0)	0 (0.0)	0 (0.0)
Baseline status			
SpO_2_, %	95.0 (93.0–96.0)	95.0 (93.0–96.0)	95.0 (93.0–96.0)
SpO_2_/FiO_2_	207 (131–263)	212 (138–267)	194 (122–263)
PaO_2_/FiO_2_	345 (174–363)	345 (153–363)	344 (206–361)
Intervention characteristics			
Number of doses	15 (7–22)	15 (7–22)	–
Total dose of heparin, U × 100	2250 (1050–4500)	2250 (1050–4500)	–

Data are median (quartile 25th–quartile 75th) or number (percentage).

COPD is chronic obstructive pulmonary disease; COVID is coronavirus disease; IV is intravenous; SC is subcutaneous; MOCS is Modified Ordinal Clinical Scale; U is units; NIV is non-invasive ventilation; HFNO is high flow nasal oxygen; IMV is invasive mechanical ventilation; ECMO is extracorporeal membrane oxygenation.

### Exposure

In total, 238 patients were randomised to the nebulised UFH group and 240 patients to the control group. In the control group, 215 patients received standard care, and 25 patients received placebo (nebulised normal saline). In patients where this was collected, the median number of nebulised UFH doses administered was 15 (IQR 7–22) and the median total dose of nebulised UFH was 225,000 (IQR 10,500–450,000) IU.

### Intubation or death

**Fig. 2:**
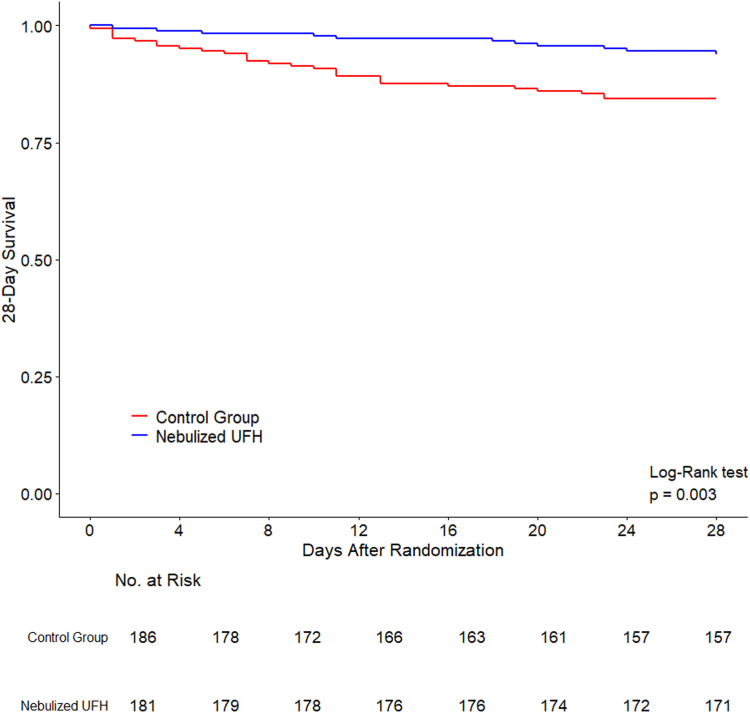
Kaplan–Meier plot for 28-day mortality.

**Supplemental Fig. S1:**
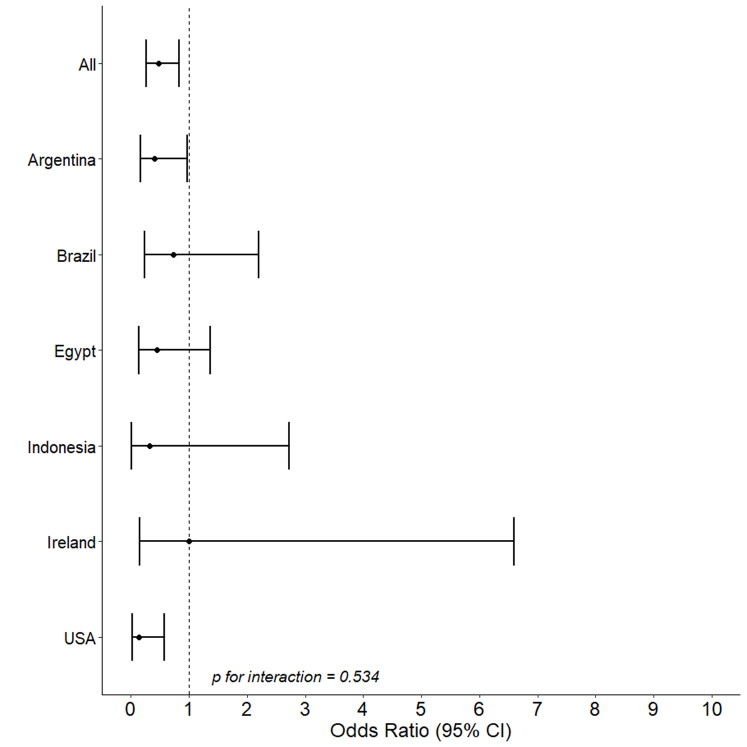
Supplemental Fig. 1: Results intubation or death per study—analysis for heterogeneity.

**Supplemental Fig. S2:**
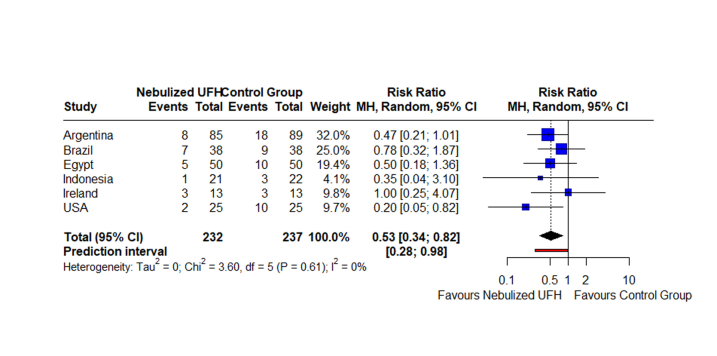
Supplemental Fig. 2: Stage 2 IPD Meta-analysis intubation or death.

**Supplemental Fig. S3:**
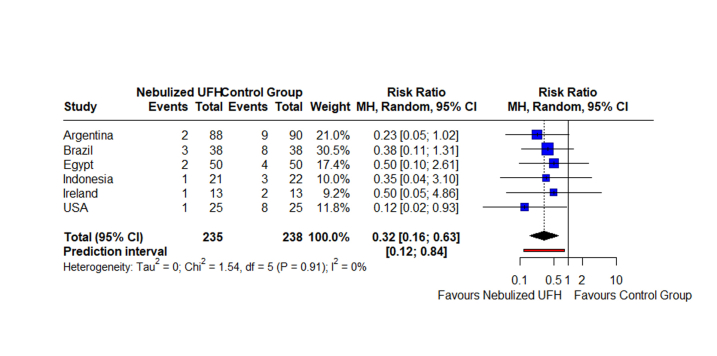
Supplemental Fig. 3: Stage 2 IPD Meta-analysis in-hospital mortality.

Patients assigned to nebulised UFH had lower rates of intubation or death at the longest available follow up (26/232, 11.2%), compared to the control group (53/237, 22.4%; OR 0.43 [0.26 to 0.73], p = 0.001, Table 3). In-hospital mortality in the nebulised UFH group was 4.3% versus 14.3% in the control group (OR, 0.26 [0.13 to 0.54], p < 0.001). Fig. 2 shows the Kaplan–Meier graph for 28-day mortality, which was significantly lower in the nebulised UFH group (hazard ratio 0.36, 95% CI, 0.18–0.73, p = 0.004). There was no heterogeneity in treatment effect between studies, suggesting that the overall effect was consistent between studies (Supplemental Fig. S1). There was no difference in treatment effect between patients who received oxygen therapy at baseline versus patients who did not (p = 0.50 for interaction). To further test the robustness of our findings, we analysed the studies by using a two-stage individual patient data meta-analysis combining aggregate data, which confirmed the significant differences between the groups regarding intubation or death (risk ratio 0.53, 95% CI 0.34–0.82, Supplemental Fig. S2) and in-hospital mortality (risk ratio 0.32, 95% CI 0.16–0.63, Supplemental Fig. S3). After excluding data from the only randomised controlled study (USA), we still found a consistent reduction of intubation or death at the longest follow up in the nebulised UFH group compared to the control group (OR, 0.51 [0.30 to 0.88], p = 0.016).

**Table 3:** Clinical outcomes.

	Overall (*n* = 478)	Nebulised Heparin (*n* = 238)	Control Group (*n* = 240)	Effect Estimate^a^ (95% CI)	p value
Intubation or death at the longest follow-up—no. (%)	79/469 (16.8)	26/232 (11.2)	53/237 (22.4)	OR, 0.43 (0.26–0.73)	0.001
Intubation at the longest follow-uo	61/469 (13.0)	21/234 (9.0)	40/235 (17.0)	OR, 0.48 (0.27–0.84)	0.011
Hospital mortality	44/473 (9.3)	10/235 (4.3)	34/238 (14.3)	OR, 0.26 (0.13–0.54)	<0.001
Days from randomisation to mortality	11.0 (4.5–19.5)	18.5 (8.8–23.2)	9.0 (3.5–14.5)	MD, 9.71 (−0.09 to 19.51)	0.059
28-day mortality—no. (%)	41/368 (11.1)	12/182 (6.6)	29/186 (15.6)	OR, 0.38 (0.18–0.77)	0.007
Hospital length of stay, days	6.0 (4.0–11.0)	6.0 (3.0–10.5)	7.0 (4.0–11.0)	SHR, 1.30 (1.05–1.63)	0.018
Day 7 WHO MOCS					
Not hospitalised	122/390 (31.3)	61/193 (31.6)	61/197 (31.0)		
Hospitalised, no oxygen, no COVID-19 care	10/390 (2.6)	1/193 (0.5)	9/197 (4.6)		
Hospitalised, no oxygen, COVID-19 care	130/390 (33.3)	73/193 (37.8)	57/197 (28.9)		
Hospitalised, oxygen	37/390 (9.5)	21/193 (10.9)	16/197 (8.1)		
Hospitalised, NIV or HFNO	40/390 (10.3)	17/193 (8.8)	23/197 (11.7)		
Hospitalised, IMV or ECMO	36/390 (9.2)	16/193 (8.3)	20/197 (10.2)		
Death	15/390 (3.8)	4/193 (2.1)	11/197 (5.6)		
Adverse events—no. (%)					
Major bleeding	0/475 (0.0)	0/235 (0.0)	0/240 (0.0)	–	–
Pulmonary bleeding	0/475 (0.0)	0/235 (0.0)	0/240 (0.0)	–	–
Heparin-induced thrombocytopenia	0/432 (0.0)	0/214 (0.0)	0/218 (0.0)	–	–

Data are median (quartile 25th–quartile 75th) or number (percentage).

Abbreviations: CI is confidence interval; OR is odds ratio; MD is median difference; SHR is subdistribution hazard ratio; NIV is non-invasive ventilation; HFNO is high flow nasal oxygen; IMV is invasive mechanical ventilation; ECMO is extracorporeal membrane oxygenation; MOCS is Modified Ordinal Clinical Scale.

a Odds ratio estimated from a mixed-effect logistic regression with trial as random effect. Median difference estimated with a mixed-effect median regression using an interior–point algorithm with trial as clustering effect. Subdistribution hazard ratio assessed using Fine–Gray competing risk models with death before discharge treated as a competing event and including study as clustering.

### Other endpoints

**Fig. 3:**
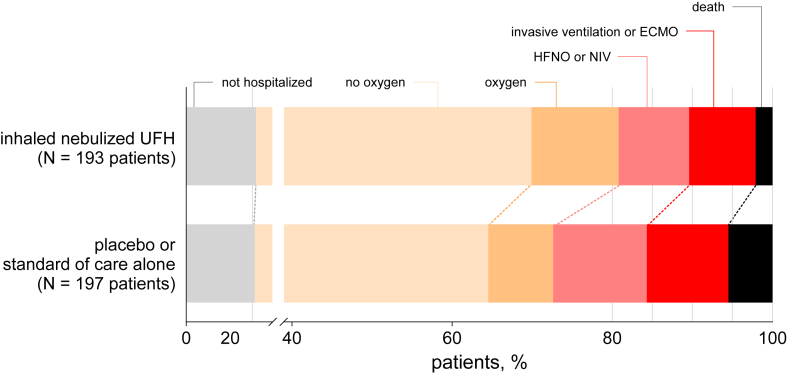
Day 7 distribution of the WHO modified ordinal clinical scale (MOCS) for COVID-19.

**Supplemental Fig. S4:**
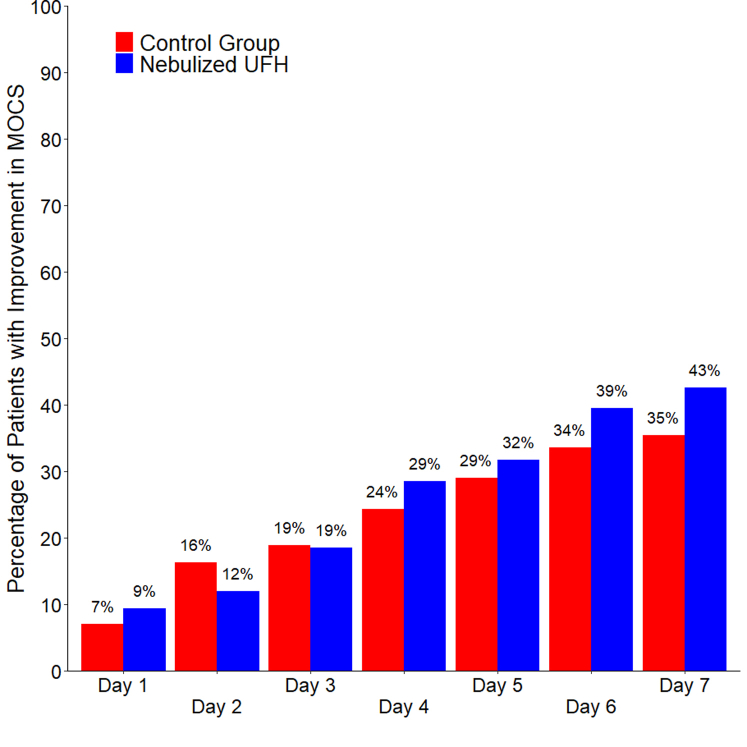
Supplemental Fig. 4: Proportion of patients with improvement in MOCS over time.

More patients in the nebulised UFH group had an improvement of their WHO MOCS over time compared to the control group. On day 7, the percentage of patients with an improvement in their MOCS was 43% in the nebulised UFH group versus 35% in the control group (Supplemental Fig. S4). At day 7, there was a significant difference in MOCS between groups (p = 0.032), with a larger proportion of patients in the control group compared to the nebulised UFH group still requiring HFNO or NIV (12% versus 9%), or IMV (10% versus 8%) (Fig. 3).

The nebulised UFH group had a shorter median hospital length of stay compared to the control group (6 [IQR 3–10.5] versus 7 [IQR 4–11] days) when death before discharge was treated as a competing event and including study as clustering (SHR 1.30, 95% CI 1.05–1.63, p = 0.018; Table 3).

**Fig. 4:**
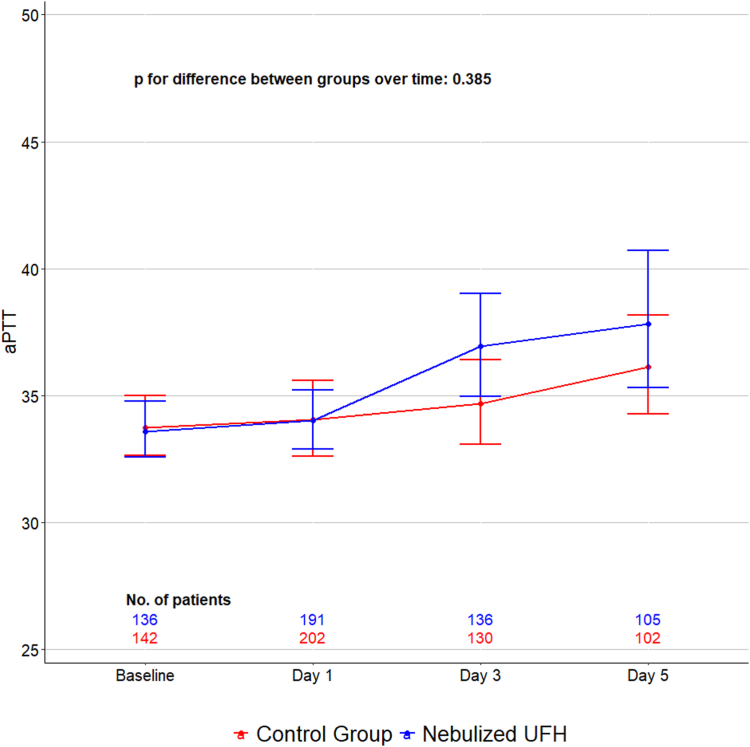
Activated partial thromboplastin time.

There were no patients reported with pulmonary haemorrhage, major bleeding or HIT in either group. No other serious adverse events were reported. In patients receiving nebulised UFH, aPTT levels remained in the normal range and were not significantly higher than in the control group (Fig. 4).

## Discussion

In this prospective, multicentre, international, meta-trial of randomised clinical studies, inhaled nebulised UFH on top of standard of care prevented intubation and death in hospitalised non-intubated COVID-19 patients. A larger proportion of patients who received nebulised UFH showed improvement of their COVID-19 respiratory disease severity as assessed by the WHO MOCS, and patients in this group had a shorter hospital length of stay. The administration of nebulised UFH was safe in our study. There were no serious adverse effects reported, no pulmonary or major bleeding in either group, and there was no effect on systemic coagulation, as assessed by aPTT.

Our results are consistent with previous preclinical and clinical studies of acute lung injury.^12^ In a double-blind randomised placebo-controlled trial in 50 patients, nebulised heparin or placebo was administered to patients expected to require mechanical ventilation for more than 48 h. Nebulised heparin was associated with a significant improvement in ventilator-free days amongst survivors at day 28.^18^ In a pre-pandemic double-blind randomised study in 256 critically ill ventilated patients, nebulised UFH limited progression of lung injury including ARDS and accelerated return to home in survivors.^19^ In a multicentre case series of 98 hospitalised COVID-19 patients, published by our group to confirm safety of the use of inhaled nebulised UFH in this patient group, UFH was associated with an improvement in oxygenation variables in intubated as well as non-intubated patients.^20^

Although we did not analyse whether there was an association between the dose of treatment with nebulised UFH and the reported outcomes, the contributing study from Brazil, which was published recently, suggested there may have been an effect of treatment duration on outcome.^21^ In that study, the data were analysed using a modified intention to treat method, which excluded subjects who died or who were admitted to ICU within 24 h of randomization and only included patients randomised to nebulised UFH who received at least 4 administrations of nebulised UFH. Using this method, there was a significant difference between groups on mortality and on WHO MOCS improvement. A post-hoc analysis showed that the effect on mortality was greatest in patients who received at least 6 administrations of nebulised UFH.^21^

It is also important to point out that some of the studies included individuals with very mild respiratory disease, who were not receiving oxygen therapy at randomisation. However, the use of oxygen or lack thereof at baseline did not affect the treatment effect of nebulised UFH.

The proposed mechanisms of action of inhaled nebulised UFH are a combination of antiviral, anti-inflammatory and anti-coagulant effects, which have recently been reviewed elsewhere.^22^ UFH has demonstrated antiviral activity in pre-clinical studies in concentrations relevant for administration to humans. The SARS-CoV-2 Spike S1 protein receptor binding domain attaches to UFH and undergoes conformational change that prevents it from binding to the Angiotensin Converting Enzyme 2 receptor, inhibiting infection.^23^, ^24^, ^25^, ^26^ This antimicrobial effect of UFH is not unique to SARS-CoV-2, as a large number of viral but also bacterial pathogens depend upon interactions with proteoglycan molecules such as heparan sulphate, which is expressed on a range of human tissue surfaces, for adhesion and invasion of host tissues.^27^ UFH also limits adhesion of a number of pathogens including Pseudomonas aeruginosa, Burkholderia cenocepacia, Burkholderia pseudomallei, Legionella pneumophila, Staph aureus, Strep pyogenes, Strep pneumonia, Respiratory syncytial virus and Influenza A.^28^^,^^29^ Human and animal studies suggest these actions may reduce the development of pneumonia and bacteraemia.^30^^,^^31^ The anti-inflammatory effects of inhaled UFH are thought to reduce pulmonary hyperinflammation and the generation of DNA NETs, both of which contribute to COVID-19 lung injury.^12^^,^^22^ The anticoagulant actions of nebulised UFH may limit fibrin deposition, hyaline membrane formation and microvascular thrombosis, which are also important features of COVID-19.^12^^,^^22^ Animal studies of nebulised UFH in different acute lung injury models have shown a positive effect on pulmonary coagulation, inflammation and oxygenation. These effects seem to be at least partly modulated by alveolar macrophages.^32^ Early-phase trials in patients with acute lung injury and related conditions found that nebulised UFH limited pulmonary fibrin deposition, coagulation activation and microvascular thrombosis, reduced pulmonary dead space, improved lung injury and increased time free of ventilatory support.^18^^,^^33^, ^34^, ^35^ In the contributing study from Egypt, the investigators measured D-dimer levels in bronchoalveolar lavage (BAL) fluid from patients with COVID-19 obtained by performing mini-BAL at baseline and on days 3 and 7.^36^ Although both the nebulised UFH and the control group showed a reduction of D-dimer levels in the BAL fluid over time, the reduction was significantly greater on day 3 in the nebulised UFH group compared to controls, suggesting nebulised UFH had an effect on the coagulopathy in the lungs of patients with COVID-19.^36^

The safety profile of inhaled nebulised UFH in our meta-trial is consistent with results from previous studies. In our previously mentioned case series of hospitalised COVID-19 patients, the addition of inhaled nebulised UFH had no clinically relevant effect on systemic coagulation as assessed by aPTT and was not associated with significant bleeding complications.^20^ All patients in that study also received systemic anticoagulation, with 53/98 receiving prophylactic doses and 45/98 patients receiving therapeutic anticoagulation, mostly with low molecular weight heparin (LMWH). In the also previously referenced randomised placebo-controlled phase 3 trial, nebulised UFH was administered in a dose of 25,000 IU every 6 h using a vibrating mesh nebuliser, and treatment with nebulised UFH modestly increased peak APTT in patients who concomitantly received intravenous or subcutaneous UFH. There was no effect on the peak APTT in patients who did not receive intravenous or subcutaneous UFH or in patients who received intravenous or subcutaneous LMWH.^19^

The strengths of this prospective meta-trial include the prospective study design, the sample size and the international scope. The inclusion of studies conducted in low-, middle- and high-income countries increases effect size estimates across different conditions as well as the external validity of the results. All studies were investigator-initiated without pharmaceutical industry involvement. Another strength is the assessment of a biologically plausible intervention, based on robust previous pre-clinical and clinical research.

The present work has limitations. First, although the USA trial was blinded and placebo controlled, all other studies were unblinded without placebo. During the pandemic, there were significant uncertainties and concerns regarding the use of nebulised treatments and the potential effects on viral transmission. Therefore, the control groups in all but one studies did not receive nebulised placebo treatment. Second, the meta-trial design has disadvantages compared with a multisite trial following a common protocol at all sites. Individual studies had different protocols and used different delivery methods and doses. For example, vibrating mesh nebulisers deliver finer particle sizes than other approaches and therefore may deliver proportionally more of the dose to the distal airways and alveoli.^37^ In addition, different types of UFH were used in each of the countries. Heparins are sourced and produced from either porcine mucosal tissue or bovine lung. Whilst all heparin preparations are standardised internationally according to their anti-coagulant effect, heparins from different sources and methods of preparation are heterogenous polysaccharides and have not been standardised for other pharmacological effects such as anti-inflammatory or anti-viral effects. This could have introduced a level of variability in the results obtained from different countries as it has been previously reported that different types of UFH may have different effects on coagulation and inflammation.^38^ However, despite these differences between the study protocols, we did not find heterogeneity between study results, suggesting that the overall effect was consistent between studies. Third, recruitment into the studies ceased before the pre-specified meta-trial sample size could be reached, in line with the unpredictable nature of the pandemic. However, the effect size estimate was larger than predicted, statistically significant, clinically meaningful and consistent across the different studies and across additional sensitivity analyses. Fourth, the current meta trial excluded patients who were already intubated and therefore our results cannot be extrapolated to COVID-19 patients who require invasive mechanical ventilation. It is possible that the effects of the intervention are different for this more severe group of patients, and that is the topic of another meta-trial of which the results are awaited.^39^ Finally, the studies in this meta-trial were done amid the pandemic, which created significant challenges for all researchers, and which resulted in missing data for some variables. For example, oxygenation variables were not consistently collected in all studies, which prevented us from presenting a more granular picture of the effect of the intervention on COVID-19 associated respiratory symptoms, other than the change in WHO MOCS over time which we reported. In conclusion, in this investigator-initiated international individual patient data meta-trial of randomised clinical studies, inhaled nebulised UFH was safe and significantly reduced intubation and mortality rates in hospitalised COVID-19 patients. The positive results from this study have implications for the future treatment of lung injury arising from COVID-19 and potentially from other causes. Further controlled clinical studies of nebulised UFH in hospitalised patients with lung injury, particularly resulting from respiratory infections, are now urgently required.

## Contributors

FvH, CP, AA, BD and JL designed the meta-trial project. AV, RA, TI, RM, MB, GDN, CS, TW, DS, AW, JS, DC, BM, CC, GC, TS, SS, KS, CS, AB, CH, SKM, DA, AS, MY, HH, RS, ANA, and SAP designed and conducted the individual trials. All authors significantly contributed to the conduct of the meta-trial. ASN provided statistical analysis. SV collected, merged and cleaned the data from all studies. FvH, ASN and SV had full access to the data and verified the data. FvH drafted the manuscript. All authors vouch for the accuracy and completeness of data and for adherence to the individual study protocols. All authors reviewed the manuscript for important intellectual content and approved the final manuscript. The members of the INHALE HEP CRG Executive Committee FvH (Chair), CP, AA, BD, MS and JL equally contributed to the overall project described in this article and were responsible for the decision to submit the manuscript.

## Data sharing statement

The research protocols for the meta-trial and each individual trial are available in the appendix. De-identified data will be available from 9 months to 36 months after article publication to researchers who provide a methodologically sound and ethically approved proposal, for any purpose of analysis.

## Declaration of interests

AA reports a research grant and/or fees from Grifols, Loop Diagnostic, and Fundacion Lilly, and a patent Regen4sepsis, and serves on several DSMBs, outside the submitted work. MCR reports a research grant from Grifols and from Loop Diagnostic, and a patent Regen4sepsis, outside the submitted work. TW is Chief Investigator of the ACCORD COVID Research Programme, Senior Clinical Lead of the National Respiratory Audit Programme, and reports grants and/or fees and/or honoraria from Sanofi, Janssen, GSK, AZ, MMH, Syairgen, Enanta, Ockham, Tidalsense and Biomerieux, patents with the University of Southampton, and stock or stock options with MMH Ltd and Tidalsense, all outside the submitted work. DS reports fees from Adovate, Aerogen, Almirall, Apogee, Arrowhead, AstraZeneca, Bial, Boehringer Ingelheim, Chiesi, Cipla, CONNECT Biopharm, Covis, CSL Behring, DevPro Biopharma LCC, Elpen, Empirico, EpiEndo, Genentech, Generate Biomedicines, GlaxoSmithKline, Glenmark, Kamada, Kinaset Therapeutics, Kymera, Menarini, MicroA, OM Pharma, Orion, Pieris Pharmaceuticals, Pulmatrix, Revolo, Roivant Sciences, Sanofi, Synairgen, Tetherex, Teva, Theravance Biopharma, Upstream, Verona Pharma, outside the submitted work. JL reports receiving personal fees from Cellenkos, and sits on several DSMBs, outside the submitted work. CP reports receiving fees from EpiEndo, Eurodrug, Recipharma, Ananda Pharma, Signant Healthcare, and Microa outside of the submitted work. He also has equity in Ananda Developments and EpiEndo, outside of the submitted work. He is Past President of the British Pharmacological Society; Trustee of the William Harvey Research Foundation, London, United Kingdom; Trustee and Chairman of the Board of trustees of the Fraunhofer Institute of Experimental Medicine and Toxicology, Hannover, Germany; Trustee of the Alec Oxford Charitable Trust, United Kingdom, outside of the submitted work. ASN reports receiving honoraria for presentations from Drager and Hamilton Medical, outside of the submitted work. All other authors have nothing to disclose.

## Appendix ASupplementary data

Supplemental Table S2

ANU Data Sharing Agreement INHALE-HEP

Protocols meta-trial and individual studies
