# Supplementary material for: Efficacy of inhaled nebulised unfractionated heparin to prevent intubation or death in hospitalised patients with COVID-19: an investigator-initiated international meta-trial of randomised clinical studies
Source: eClinicalMedicine. 2025 Sep 27;88:103339. doi: 10.1016/j.eclinm.2025.103339 (PMC12572793; doi:10.1016/j.eclinm.2025.103339)
Supplement: Supplemental Table S1 [file mmc1.docx]

| **Supplemental Table 1 – Characteristics per Study** | | | | | | |
| --- | --- | --- | --- | --- | --- | --- |
|  | **Argentina**  **(*n* = 183)** | **Brazil**  **(*n* = 76)** | **Egypt**  **(*n* = 100)** | **Indonesia**  **(*n* = 43)** | **Ireland**  **(*n* = 26)** | **USA**  **(*n* = 50)** |
| Age, years | 59.0 (49.2 - 70.0) | 52.0 (45.0 - 58.2) | 48.0 (41.0 - 54.0) | 51.0 (40.5 - 62.5) | 54.0 (48.2 - 61.2) | 62.5 (54.2 - 70.5) |
| Male sex – no. (%) | 112 (61.5) | 48 (63.2) | 63 (63.0) | 22 (51.2) | 18 (69.2) | 30 (60.0) |
| From home – no. (%) | --- | --- | --- | 42 (100.0) | --- | 50 (100.0) |
| Body mass index, kg/m^2^ | 24.0 (18.4 - 30.0) | --- | 27.0 (24.0 - 29.0) | 23.4 (21.9 - 24.7) | 31.5 (27.0 - 36.0) | 31.1 (27.3 - 36.2) |
| Co-morbidities – no. (%) |  |  |  |  |  |  |
| Smoking | 8 (22.9) | --- | 15 (15.0) | 5 (11.9) | 3 (12.5) | 1 (2.0) |
| Asthma or COPD | 8 (22.9) | 0 (0.0) | 9 (9.0) | 0 (0.0) | 3 (11.5) | 6 (12.0) |
| Hypertension | 21 (60.0) | --- | 23 (23.0) | 7 (16.7) | 10 (38.5) | 20 (40.0) |
| Cardiac disease | 0 (0.0) | --- | 13 (13.0) | --- | --- | 3 (6.0) |
| Diabetes | 13 (37.1) | --- | 22 (22.0) | 8 (19.0) | 4 (15.4) | 13 (26.0) |
| Chronic kidney disease | 0 (0.0) | --- | 5 (5.0) | --- | --- | 3 (6.0) |
| COVID-19 therapies – no. (%) |  |  |  |  |  |  |
| Corticosteroids | 157 (87.2) | 32 (42.1) | 87 (87.0) | 21 (48.8) | 26 (100.0) | 50 (100.0) |
| Tocilizumab | --- | --- | --- | --- | --- | 14 (28.0) |
| Remdesivir | 0 (0.0) | --- | --- | 19 (44.2) | --- | 49 (98.0) |
| Macrolides | 146 (81.1) | --- | --- | 12 (27.9) | --- | --- |
| Convalescent plasma | 13 (7.1) | 0 (0.0) | 0 (0.0) | 0 (0.0) | 0 (0.0) | 0 (0.0) |
| Non-COVID-19 therapies – no. (%) |  |  |  |  |  |  |
| Oseltamivir | 3 (1.7) | --- | --- | 6 (14.0) | --- | --- |
| Antibiotics | 148 (82.2) | 53 (70.7) | 96 (100.0) | 23 (53.5) | 25 (96.2) | 44 (89.8) |
| Antifungal | 3 (1.7) | --- | --- | 1 (2.3) | 5 (19.2) | --- |
| IV or SC heparin | 125 (78.6) | 56 (76.7) | 94 (97.9) | 27 (64.3) | 26 (100.0) | 48 (96.0) |
| Baseline WHO MOCS |  |  |  |  |  |  |
| Not hospitalised | 0 (0.0) | 0 (0.0) | 0 (0.0) | 0 (0.0) | 0 (0.0) | 0 (0.0) |
| Hospitalised, no oxygen, no COVID-19 care | 12 (9.4) | 0 (0.0) | 0 (0.0) | 0 (0.0) | 0 (0.0) | 0 (0.0) |
| Hospitalised, no oxygen, COVID-19 care | 59 (46.1) | 7 (9.3) | 100 (100.0) | 1 (2.5) | 0 (0.0) | 3 (6.0) |
| Hospitalised, oxygen | 44 (34.4) | 44 (58.7) | 0 (0.0) | 13 (32.5) | 0 (0.0) | 32 (64.0) |
| Hospitalised, NIV or HFNO | 13 (10.2) | 24 (32.0) | 0 (0.0) | 26 (65.0) | 26 (100.0) | 15 (30.0) |
| Hospitalised, IMV or ECMO | 0 (0.0) | 0 (0.0) | 0 (0.0) | 0 (0.0) | 0 (0.0) | 0 (0.0) |
| Death | 0 (0.0) | 0 (0.0) | 0 (0.0) | 0 (0.0) | 0 (0.0) | 0 (0.0) |
| Baseline status |  |  |  |  |  |  |
| SpO_2_, % | 95.0 (92.8 - 97.0) | 92.0 (90.0 - 95.0) | 95.0 (94.0 - 95.0) | 97.0 (94.0 - 98.0) | 96.0 (94.2 - 98.0) | 94.0 (91.2 - 96.0) |
| SpO_2_ / FiO_2_ | --- | 227 (140 - 260) | --- | 163 (116 - 239) | --- | 210 (150 - 278) |
| PaO_2_ / FiO_2_ | --- | --- | 356 (345 - 365) | 125 (82 - 172) | 154 (129 - 206) | --- |
| Intervention characteristics |  |  |  |  |  |  |
| Nebulized heparin group |  |  |  |  |  |  |
| Number of doses | 21 (15 - 21) | 12 (5 - 23) | --- | --- | 12 (4- 17) | 14 (8 - 22) |
| Total dose of heparin, Ux100 | 1050 (750 - 1050) | 3000 (1250 - 5875) | --- | 3000 (2000 - 3000) | 3125 (1562 - 4192) | 3500 (2000 - 5500) |
| Control group |  |  |  |  |  |  |
| Number of doses | --- | --- | --- | --- | --- | --- |
| Total dose of heparin, Ux100 | --- | --- | --- | --- | --- | --- |
| Longest follow-up (days) | 28 | 28 | 28 | 28 | 21* | 60 |

* Follow-up until hospital discharge, mean 21.4 days (SD 15.6 days)
