# Supplementary material for: Efficacy of inhaled nebulised unfractionated heparin to prevent intubation or death in hospitalised patients with COVID-19: an investigator-initiated international meta-trial of randomised clinical studies
Source: eClinicalMedicine. 2025 Sep 27;88:103339. doi: 10.1016/j.eclinm.2025.103339 (PMC12572793; doi:10.1016/j.eclinm.2025.103339)
Supplement: Supplemental Table S2 [file mmc2.docx]

| **Supplemental Table 2 – Rate of Missing Data per Study** | | | | | | |
| --- | --- | --- | --- | --- | --- | --- |
|  | **Argentina**  **(*n* = 183)** | **Brazil**  **(*n* = 76)** | **Egypt**  **(*n* = 100)** | **Indonesia**  **(*n* = 43)** | **Ireland**  **(*n* = 26)** | **USA**  **(*n* = 50)** |
| Age | 1 (0.5) | 0 (0.0) | 0 (0.0) | 0 (0.0) | 0 (0.0) | 0 (0.0) |
| Gender | 1 (0.5) | 0 (0.0) | 0 (0.0) | 0 (0.0) | 0 (0.0) | 0 (0.0) |
| From home | NC | NC | NC | 1 (2.3) | NC | 0 (0.0) |
| Body mass index | 147 (80.3) | NC^ | 0 (0.0) | 2 (4.7) | 0 (0.0) | 0 (0.0) |
| Co-morbidities |  |  |  |  |  |  |
| Smoking | 148 (80.9) | NC | 0 (0.0) | 1 (2.3) | 2 (7.7) | 1 (2.0) |
| Asthma or COPD | 148 (80.9) | 0 (0.0) | 0 (0.0) | 1 (2.3) | 0 (0.0) | 0 (0.0) |
| Hypertension | 148 (80.9) | NC | 0 (0.0) | 1 (2.3) | 0 (0.0) | 0 (0.0) |
| Cardiac disease | 179 (97.8) | NC | 0 (0.0) | NC | NC | 0 (0.0) |
| Diabetes | 148 (80.9) | NC | 0 (0.0) | 1 (2.3) | 0 (0.0) | 0 (0.0) |
| Chronic kidney disease | 179 (97.8) | NC | 0 (0.0) | NC | NC | 0 (0.0) |
| COVID-19 therapies |  |  |  |  |  |  |
| Corticosteroids | 3 (1.6) | 0 (0.0) | 0 (0.0) | 0 (0.0) | 0 (0.0) | 0 (0.0) |
| Tocilizumab | NC | NC | NC | NC | NC | 0 (0.0) |
| Remdesivir | 3 (1.6) | NC | NC | 0 (0.0) | NC | 0 (0.0) |
| Convalescent plasma | 0 (0.0) | 0 (0.0) | 0 (0.0) | 0 (0.0) | 0 (0.0) | 0 (0.0) |
| Non-COVID-19 therapies |  |  |  |  |  |  |
| Oseltamivir | 3 (1.6) | NC | NC | 0 (0.0) | NC | NC |
| Antibiotics* | 3 (1.6) | 1 (1.3) | 4 (4.0) | 0 (0.0) | 0 (0.0) | 1 (2.0) |
| Antifungal | 3 (1.6) | NC | NC | 0 (0.0) | 0 (0.0) | NC |
| IV or SC heparin | 24 (13.1) | 3 (3.9) | 4 (4.0) | 1 (2.3) | 0 (0.0) | 0 (0.0) |
| Baseline WHO Modified Clinical Scale | 55 (30.1) | 1 (1.3) | 0 (0.0) | NC | 0 (0.0) | 0 (0.0) |
| Baseline status |  |  |  |  |  |  |
| SOFA | NC | NC | NC | NC | 4 (15.4) | NC |
| SpO_2_ | 55 (30.1) | 5 (6.6) | 0 (0.0) | 2 (4.7) | 0 (0.0) | 0 (0.0) |
| SpO_2_ / FiO_2_ | 135 (73.7) | 10 (13.2) | NC | 4 (9.3) | NC | 0 (0.0) |
| PaO_2_ / FiO_2_ | NC | NC | 0 (0.0) | 12 (27.9) | 0 (0.0) | NC |
| Intervention characteristics |  |  |  |  |  |  |
| Number of doses | 69 (37.7) | 0 (0.0) | NC | NC | 0 (0.0) | 0 (0.0) |
| Total of heparin, Ux100 | 160 (87.4) | 38 (50.0) | NC | 26 (60.5) | 14 (53.8) | 25 (50.0) |
| Intubation at the longest follow-up | 9 (4.9) | 0 (0.0) | 0 (0.0) | 0 (0.0) | 0 (0.0) | 0 (0.0) |
| Intubation or death at the longest follow-up | 9 (4.9) | 0 (0.0) | 0 (0.0) | 0 (0.0) | 0 (0.0) | 0 (0.0) |
| 28-day mortality | 2 (1.1) | 0 (0.0) | NC | 1 (2.3) | 7 (26.9) | 0 (0.0) |
| Hospital mortality | 5 (2.7) | 0 (0.0) | 0 (0.0) | 0 (0.0) | 0 (0.0) | 0 (0.0) |
| Day 7 WHO Modified Clinical Scale | 22 (12.0) | 20 (26.3) | 0 (0.0) | 43 (100.0) | 0 (0.0) | 3 (6.0) |
| Hospital length of stay | 1 (0.5) | 0 (0.0) | 100 (100.0) | 0 (0.0) | 0 (0.0) | 0 (0.0) |
| Adverse events |  |  |  |  |  |  |
| Major bleeding | 3 (1.6) | 0 (0.0) | 0 (0.0) | 0 (0.0) | 0 (0.0) | 0 (0.0) |
| Pulmonary bleeding | 3 (1.6) | 0 (0.0) | 0 (0.0) | 0 (0.0) | 0 (0.0) | 0 (0.0) |

NC = not collected under the country specific protocol

^Collected patient weight in kilograms with missing data rate of 14 (18.4)

*Antibiotics is defined as receiving either macrolide or non-macrolide antibiotics, or a combination of both
