# Supplementary material for: Efficacy of inhaled nebulised unfractionated heparin to prevent intubation or death in hospitalised patients with COVID-19: an investigator-initiated international meta-trial of randomised clinical studies
Source: eClinicalMedicine. 2025 Sep 27;88:103339. doi: 10.1016/j.eclinm.2025.103339 (PMC12572793; doi:10.1016/j.eclinm.2025.103339)
Supplement: ANU Data Sharing Agreement INHALE-HEP [file mmc3.pdf]

Data Sharing Agreement

Information Schedule

Parties

**ANU**	**Name:**	**Australian National University**
	**ABN:**	**52 234 063 906**
	**Address:**	**The Australian National University, Canberra 2600**
	**email:**	**Anums.rm@anu.edu.au**
	**Attention:**	**Research Manager, College of Health and Medicine**
	**Investigator:**	**Frank van Haren frank.vanharen@anu.edu.au**
**Party**	**Name:**	
	**ABN:**	
	**Address:**	
	**email:**	
	**Attention:**	
	**Investigator:**	

Background

A. A Meta-Trial is being conducted by the INHALE-HEP (INHALEd Unfractionated HEParin for the Treatment of Hospitalised Patients With COVID-19) Meta-Trial Collaborative Research Group (CRG)

B. The Meta-Trial is a prospective individual patient data meta-analysis of investigator-initiated, multi-centre, randomised, studies of inhaled unfractionated heparin in hospitalised COVID-19 patients

C. Principal Investigators from the Parties have gathered data from Study Sites for the INHALE-HEP Meta-Trial

D. The Parties agree to be part of the CRG and share their respective data in order to conduct the Meta-Trial.

E. The Parties agree to share with each other with the Data on the terms and conditions set out in this Agreement.

Date

**Date of Agreement**	

Details

Item No	Identifier	Detail
**1**	**Data**	No patient identifying data will be transferred to the data repository. The data set will use de-identified individual patient data. Local regulations for the management of patient data needs to be adhered to as part of agreement for involvement in the study. Individual studies are responsible for obtaining ethics approval to contribute IPD to the Meta-Trial where required/appropriate.

2	**Study Protocol**	The study protocol will be specific to each Party. The meta-trial protocol is described in the manuscript attached as Annexure A. The meta-trial has been registered on ClinicalTrials.gov NCT04635241
3	**Purpose**	Collaborative prospective individual patient data meta-analysis of investigator-initiated, multi-centre, randomised, studies of inhaled unfractionated heparin in hospitalised COVID-19 patients
4	**Period**	From signing of the agreement until all agreed manuscripts have been published
5	**Special Conditions**	Meta-trial governance structure and authorship as per Annexures B and C

Execution- Executed by the Parties as an Agreement:

ANU	**SIGNED** for and on behalf of **(party)** in the presence of:)))<br>Date signed: …../…../……….)<br><br>……………………………………………<br>Signature of witness	………………………………………………….<br><br>………………………………………………….<br>Name of signatory<br><br>………………………………………………….<br>Title of signatory
Party	**SIGNED** for and on behalf of **PARTY** in the presence of:)))<br>Date signed: …../…../……….)<br><br>……………………………………………<br>Signature of witness	………………………………………………….<br><br>………………………………………………….<br>Name of signatory<br><br>………………………………………………….<br>Title of signatory

General Terms

This document sets out the General Terms and the Special Conditions that are applicable to this Collaboration and Data Sharing Agreement. The signed Information Schedule constitutes an acceptance by the Parties of these General Terms, the Special Conditions and all other parts of this Agreement. The Special Conditions override the General Terms to the extent of any inconsistency.

1. Sharing of Data

In consideration of the obligations set out below, the Parties agree to share their Data for the Purpose described in Item 2 of the Information Schedule and for the Period specified in Item 3 of the Information Schedule.

2. Confidential Information

2.1 Each Party shall:

2.1.1 treat the Data, and information regarding the Data, received from a Party in the strictest confidence;

2.1.2 take all necessary steps to physically secure the Data from unauthorised disclosure or use;

2.1.3 not use all or any part of the Data for any purpose other than the Purpose;

2.1.4 not without the prior written consent of all Parties (which it may give or withhold at its discretion) directly or indirectly disclose all or any part of the Data to any third party;

2.1.5 reproduce all or part of the Data as may be reasonably required to complete the Purpose and produce reports or publications;

2.1.6 immediately advise all Parties in writing of any unauthorised disclosure or use of the Data of which it becomes aware;

2.1.7 comply with any guidelines a Party provides regarding processes to be followed if there is an unauthorised disclosure or use of the Data.

2.2 The obligations contained in clause 2.1 do not apply to information relating to the Data to the extent to which:

2.2.1 it was already known to a Party at the time of release of the Data to that Party as established by the prior written records of that Party;

2.2.2 it is now or hereafter becomes, through no act or failure to act on the part of the receiving Party, generally known or available to the public; or

2.2.3 it is furnished to a Party after the date of disclosure hereunder by a third party without breach by that third party of any obligation to the Party.

2.3 Each Party may allow access to the Data to its employees or students who are directly involved in carrying out the Purpose but only to the extent as may be reasonably required by such employee or student for the carrying out of their function in relation to the Purpose. In such case, the Parties shall ensure that any such employee or student to whom the Data is released has first entered into written confidentiality obligations in respect of the Data at least as strict as those contained in this Agreement.

2.4 The Parties agree that the INHALE-HEP Meta-Trial Executive Committee will have access to the Data. The Data will be made available to all members of the Meta-Trial steering committee and to all authors of papers arising, and this access will extend beyond publication in case there are any questions that arise after publication of the Data.

2.5 On the written request of a providing Party, the Parties shall return to the providing Party the Data, except to the extent that such Data may have been destroyed in the normal course of carrying out the Purpose.

3. INHALE-HEP Meta-Trial Executive Committee

3.1 Membership of the INHALE-HEP Meta-Trial Executive Committee is outlined in Annexure B.

3.2 Additional members of the Executive Committee may be appointed by the Executive Committee Chair.

3.3 The INHALE-HEP Meta-Trial Executive Committee will prepare a Data Collection and Management Protocol including for granting access to the meta-trial Dataset.

3.4 The INHALE-HEP Meta-Trial Executive Committee is responsible for the meta-trial design, collection management analysis and interpretation of data, writing meta-trial study reports, preparation of manuscripts for academic publication.

4. Database and access

4.1 The Parties agree to cooperate in building a REDCap database for collection of the Data.

4.2 The REDCap database will be hosted on ANU servers and ANU will provide the Parties with access to the database for the purpose of this Agreement.

4.3 Party's access to the database will be available for the Period of this Agreement.

4.4 Each disclosing Party will use its reasonable efforts to keep a record of the Data that it submits to the database pursuant to this Agreement.

4.5 Each Party will treat the Data, and information regarding the Data, in the strictest confidence.

5. Intellectual Property and Licence

The Parties agree and acknowledge:

5.1 the Data, and the intellectual property subsisting in it, remain at all times the property of the providing Party and this Agreement does not constitute or evidence the sale or assignment of such rights. Receiving Parties do not obtain any legal or equitable right whatsoever to or in the Data, other than the right to use the Data for the Purpose on the terms and conditions of this Agreement;

5.2 Each disclosing Party grants to each receiving Party a non-exclusive, non-transferable, royalty and fee free, revocable licence to use the disclosing Party's Data for the Purpose.

5.3 the use of the Data must be limited to experimental and research use by the Investigator, or a student under the Investigator's supervision, within the Party's premises only;

5.4 the Data must not be transferred to any third party for any purposes except that the Investigator may allow a student to use the Data for the purpose of research for a PhD thesis; and

5.5 where the Investigator is a student or not an employee of a Party, the Investigator will enter a Deed Poll with net to govern their obligations their respective Party in respect of the Data.

6. Use of the Data

6.1 The Parties warrant that each other Party may use the Data without infringing the rights of a third party.

6.2 Receiving Parties acknowledge that they may access and use the Data entirely at their own risk.

6.3 The Parties acknowledges that:

6.3.1 other than the warranty in clause 4.1, no representation of any kind whatsoever has been made, and no warranty or condition has been given (including, without limiting the generality thereof, that the Data is safe to be used for the Purpose or that it is of merchantable quality), by or on behalf of the providing Party nor is any to be implied in relation to the Data; and

6.3.2 in carrying out the Purpose and in the storage and use of the Data, the Parties shall exercise all reasonable care, employ good research practice and shall comply with all applicable laws and regulations.

7. Default And Termination

7.1 If a Party:

7.1.1 Fails to comply with any obligations under this Agreement;

7.1.2 Uses the Data for a purpose other than the Purpose;

7.1.3 Otherwise breaches a material term of this Agreement;

And fails to remedy the non-compliance or breach within 30 days after receiving notice from a Party requiring it to do so, then the non-compliance or breach may be referred to the CHARTER MT Executive Committee for resolution of the matter/ OR the Party may be terminated from this Agreement with immediate effect.

7.2 The Parties may terminate this Agreement by mutual agreement by giving 60 days written notice to the other Parties or such lesser period of notice as is agreed in writing by all Parties.

7.3 The termination of this Agreement is without prejudice to any rights which have accrued to a Party before the date of termination.

7.4 Upon termination of this Agreement, each receiving Party will

7.4.1 immediately stop accessing the database

7.4.2 immediately stop using any Data in its possession or control and the licence to the Data ceases;

7.4.3 if the Data comprises Confidential Information of a providing Party and if requested by the providing Party in the written notice, permanently destroy any hard copies of the Data and permanently delete all electronic copies of Data in its possession or control; and

7.4.4 If the Data comprises Confidential Information of a providing Party and if requested by the providing Party, confirm in writing that this clause has been fully complied with.

8. Indemnity

Each Party ("First Party") shall indemnify and keep indemnified each other Party ("Second Party), its officers and employees from and against all losses or liability suffered or incurred by the Second Party in relation to a claim arising directly from a negligent act by the First Party or its officers or employees in connection with the possession, storage, use, transport and disclosure of the Data. The First Party's liability to indemnify the Second Party will be reduced proportionally to the extent that any negligent or wrongful act or omission or wilful misconduct on the Second Party's part contributed to the relevant loss, damage, expense, or liability.

9. Publications

9.1 The Parties will acknowledge each other Party as the source of the Data in all publications containing any data or information about the Data. For the avoidance of doubt, this clause does not limit the generality of clause 2.

9.2 The Parties agree that Data from individual trials may be published by the Party that creates that Data.

9.3 The Parties agree that publications relating to the Meta-Trial will be prepared by the CRG Executive Committee as described in Annexure C.

10. Development

In the event that in using the Data for any purpose (including for the Purpose), a Party makes a discovery or derives information or knowledge or creates intellectual property (**Development**), ownership of the Development vests in the discovering Party.

11. Publicity

11.1 Neither party may use the other party's name, trade marks or service marks or refer to the other party (inclusive of its employees or officers) directly or indirectly in any media release, public announcement or public disclosure relating to this Agreement or its subject matter, including in any promotional or marketing materials, customer lists or business presentations, without obtaining the prior written consent from the other party.

11.2 The obligation in this clause does not apply to any disclosure which a Party is required to make by law, provided that the Party so required consults with the other party as to the information to be disclosed prior to disclosure.

12. **Dis**putes

12.1 The Parties agree not to commence any legal proceedings in respect of any dispute arising under this Agreement, until the procedure provided by this clause has been followed.

12.2 The parties agree that any dispute arising during the course of this Agreement will be dealt with as follows:

12.2.1 The Party claiming that there is a dispute will send the other Parties a written notice setting out the nature of the dispute;

12.2.2 the Parties will try to resolve the dispute through direct negotiation, including by referring the matter to persons who have authority to intervene and direct some form of resolution;

12.2.3 the Parties have ten (10) Business Days from the date of the notice to reach a resolution or to agree that the dispute is to be submitted to mediation; and

12.2.4 if the dispute is submitted to mediation and the parties cannot agree on a mediator within ten (10) Business Days from the date of the notice, the chairperson of the Australian Commercial Disputes centre or the chairperson's nominee will appoint a mediator; and

12.2.5 if:

12.2.5.1 there is no resolution of the dispute; or

12.2.5.2 there is a submission to mediation or some other form of alternative dispute resolution procedure, but there is no resolution within fifteen (15) Business Days of the submission, or such extended time as the parties may agree in writing, then any Party may commence legal proceedings.

12.3 This clause does not apply where any of the Parties commences legal proceedings for urgent interlocutory relief.

12.4 Despite the existence of a dispute, the Parties must (unless requested in writing by a Party not to do so) continue to perform their respective obligations under this Agreement.

12.5 Each Party must pay its own costs of complying with this clause. The Parties must equally pay the costs of any mediator.

13. General

13.1 **Co-operation:** Each party must do anything (including execute any document), and must ensure that its employees and agents do anything (including execute any document), that the other party may reasonably require to give full effect to this Agreement.

13.2 **Entire Agreement:** This Agreement constitutes the entire understanding between the parties in relation to the Data. Nothing in this Agreement shall be construed so as to oblige either party to enter into any further agreement.

13.3 **Assignment:** This Agreement may not be assigned by either party without the written consent of the other party.

13.4 **Costs:** Each party remains responsible for its own costs and expenses for entering into this Agreement.

13.5 **Signatories:** The signatories to this Agreement warrant that they have the authority to enter into this Agreement on behalf of the party they are stated to represent.

13.6 **Counterparts:** This Agreement may be executed in any number of counterparts. Each counterpart is an original but the counterparts together are one and the same agreement. This Agreement is binding on the parties on the exchange of counterparts. A copy of a counterpart sent by electronic transmission –

13.6.1 must be treated as an original counterpart;

13.6.2 is sufficient evidence of the execution of the original; and

13.6.3 may be produced in evidence for all purposes in place of the original.

13.7 **Variation:** This Agreement may only be varied by a document duly executed by the Parties.

13.8 **Severability:** If a clause or part of a clause can be read in a way that makes it illegal, unenforceable or invalid, but can also be read in a way that makes it legal, enforceable and valid, it must be read in the latter way. If any clause or part of a clause is illegal, unenforceable or invalid, that clause or part is to be treated as removed from this Agreement, but the rest of this Agreement is not affected and all other provisions will remain in full force and effect.

13.9 **Governing Law:** This Agreement shall be governed by the laws of the Australian Capital Territory, Australia. Each party submits to the non-exclusive jurisdiction of the courts of that place.

13.10 **Execution:** This Agreement is null and void unless it is executed by all parties. This Agreement may be executed using electronic signing.

13.11 **Execution:** This Agreement is null and void unless it is executed by all parties. This Agreement may be executed using electronic signing.

Glossary

**Agreement**	means this Agreement including this Glossary, the Information Schedule, the General Terms, Special Conditions, all Annexures to this Agreement, and any amendment to it in writing.
**Annexure**	means an annexure to this Agreement.
**Business Day**	means a day that is not a Saturday, Sunday or any other day which is a public holiday or a bank holiday in the place where an act is to be performed or a payment is to be made.
**Collaborative Research Group**	Means all Study Sites participating in the INHALE-HEP Meta-Trial
**Confidential Information**	means:<br>(a) the Intellectual Property subsisting in any item, material, diagram, formula, model, photograph, business plan, methodology, policy, record, memorandum, documentation or material relating to the Purpose;<br>(b) any information that relates to the Purpose, including information relating to the existence, progress and status of discussions, negotiations or agreements with respect to the Purpose and including information created, ascertained, discovered or derived directly or indirectly from the Purpose;<br>(c) business, financial, technical and other commercially valuable information including what a party regards to be confidential, proprietary or sensitive; and<br>(d) any and all including information created, ascertained, discovered or derived directly or indirectly from the Confidential Information,<br>in each case, irrespective of whether:<br>(e) the disclosure is by visual, oral, written or electronic means, directly or indirectly made to a party or its related bodies corporate, officers, agents or employees;<br>(f) the information was disclosed or created before or after the date of this Agreement; or<br>(g) whether such information is designated as "confidential" by a party,<br>but does not include information which:<br>(h) prior to disclosure is in the public domain or subsequent to disclosure to the receiving party becomes part of the public domain other than as a breach of this Agreement or other obligation owed to the disclosing party;<br>(i) is received by the receiving party from a third party without any obligation to hold in confidence and which has not been obtained by that third party directly or indirectly from the receiving party; or<br>(j) is independently developed by an employee or officer of the receiving party while having no knowledge of the disclosing party's Confidential Information.
**Data**	means the data described in Item 2 of the Information Schedule.

**Data Sharing Protocol**	means the data sharing standards for the Data as agreed by the Parties from time to time during the Period.
**Data Storage and Security Protocol**	means the Data storage and security protocols as agreed by the Parties from time to time during the Period.
**Date of Agreement**	means the date of commencement of the Agreement as detailed in the Information Schedule.
**Deed Poll**	means the deed poll a student or Investigator that is not an employee of a Party will enter to govern their use of the Data.
**Development**	has the meaning given to it in clause 10 of this Agreement.
**General Terms**	means the general terms described as such in this Agreement.
**Glossary**	means this glossary.
**Information Schedule**	means the Schedule at the start of this Agreement which details the key information relevant to this Agreement.
**Intellectual Property**	means all intellectual property rights, including:<br>(a) rights in relation to inventions, including patents, plant breeders' rights, copyright (present and future), rights in circuit layouts, registered designs, trademarks and knowhow; and<br>(b) any application or right to apply for registration of any of the rights referred to in paragraph (a),<br>but excluding Moral Rights and similar non assignable personal rights of any person.
**Investigator**	means the persons identified for each Party in the Information Schedule.
**Item**	means an item in the Information Schedule.
**Meta-trial**	Means a prospective meta-analysis planned to streamline data collection from multiple individual trials, allowing for faster accumulation of data for major clinical endpoints
**Moral Rights**	means all present and future rights of integrity of authorship, rights of attribution of authorship, rights not to have authorship falsely attributed, and rights of a similar nature conferred by statute anywhere in the world.
**Parties**	means the parties to this Agreement and their respective successors and permitted assigns, and **"Party"** means any one of them.
**Party**	means each party described in the Information Schedule. A providing Party refers to a Party that has provided Data, and a receiving Party refers to a Party that has received Data, under this Agreement.
**Period**	means the period for which the Data will be used as described in Item 4 of the Information Schedule.

**Protocol**	means the version of the protocol attached as an annexure to this Agreement, and any amended version of that protocol.
**Purpose**	means the purpose for which the Data will be used as described in Item 3 of the Information Schedule.
**Schedule**	means a schedule to this Agreement.
**Special Conditions**	means the special conditions detailed in Item 5 of the Information Schedule.
**Study**	means the INHALEd unfractionated HEParin for the treatment of hospitalised patients with COVID-19 (INHALE-HEP) Meta-Trial
**Study Site**	Means the location under the control of a Party where the Study is conducted.

Annexure A

Study Protocol Manuscript

Annexure B

INHALE HEP Meta-Trial Management Structure

Chief Investigator: Professor Frank M.P. van Haren

Australian National University Medical School

frank.vanharen@anu.edu.au

+61467051809

INHALE HEP Meta-Trial Executive Committee:

Professor Frank M.P. van Haren (Chair), Australian National University, Canberra, Australia

Professor Clive Page (Co-Chair), Institute of Pharmaceutical Science, King's College London, UK

Dr Barry Dixon St Vincent's Hospital, Melbourne, Australia

Professor Antonio Artigas, Autonomous University of Barcelona, Sabadell, Spain

Professor John Laffey, National University of Ireland, Galway, Ireland

The executive committee has overall leadership and responsibility of the INHALE-HEP Meta-Trial.

INHALE-HEP Meta-Trial Steering Committee:

All members of the Executive Committee and the Principal Investigators of each participating site will be member of the steering committee. The steering committee will monitor the progress of the study.

INHALE-HEP Meta-Trial Collaborative Research Group (CRG):

All contributing investigators and nominated research coordinators and nominated other collaborators are members of the INHALE-HEP Meta-Trial CRG. All publications will be made on behalf of the INHALE-HEP Meta-Trial Investigators.

Coordinating Centre: Australian National University Medical School

Annexure C

Dissemination plans

Dissemination plans

Results will be published by a Writing Committee on behalf of the INHALE-HEP Meta-Trial Investigators in an international peer-reviewed journal. All INHALE-HEP Meta-Trial collaborators will be acknowledged in each manuscript (usually in an appendix).

Results will be presented at international conferences by INHALE-HEP Meta-Trial collaborators, nominated and/or approved by the INHALE-HEP Meta-Trial Executive Committee. These presentations will be on behalf of the INHALE-HEP Meta-Trial Investigators.

1.1. Roles and Duties of the Writing Committee

The INHALE-HEP Meta-Trial Executive Committee will select the Writing Committee. The size and composition of the Writing Committee will vary by presentation or manuscript topic and complexity but will include all members of the INHALE-HEP Meta-Trial Executive committee.

The Writing Committee will recommend a format and destination for each manuscript. The Writing Committee chairman will be the contact person to request analyses and data from the coordinating centre.

Manuscripts or presentations are generally expected to be in submission ready form for review by the Executive Committee within three months of designation of the Writing Committee.

Changes in the chair or composition of the Writing Committee can be made by the Executive Committee for failure to perform in a timely fashion.

All publicly disseminated information from the INHALE-HEP Meta-Trial or information generated using INHALE-HEP Meta-Trial Investigators resources, including but not limited to: Press releases, manuscripts, posters, abstracts, presentation slides, transparencies, video or audio tape materials must be approved by the Executive Committee before publication or presentation.

Exceptions to this requirement includes: Materials (e.g. Posters, handouts, recruitment cards) or presentations used solely to promote enrolment or inform professional audiences of the INHALE-HEP Meta-Trial structure, purpose, or clinical study design. Such presentations should not include discussion of previously unpublished INHALE-HEP Meta-Trial data and must not result in publication of study results.

1.2. Proposals for Publications arising from the INHALE-HEP Meta-Trial.

The Executive Committee encourages INHALE HEP Meta-Trial Investigators to submit suggestions for publications and presentations.

1.3. Membership of INHALE-HEP Meta-Trial Writing Committee

The Writing Committee will include all members of the Steering Committee and may include other members designated by the executive committee who are independent of the study.

1.4. Publication and Presentation Approval Process

The INHALE-HEP Meta-Trial Executive Committee Chair must approve all publications and presentations of unpublished material before journal submission or public presentation. The Chair will decide whether material needs to be circulated to the entire Executive or Steering Committee. Review of any submitted draft material by the Executive Committee will occur within 4 weeks of receipt. Revision of manuscripts and presentations will be mediated by the chairmen of the Writing Committees in consultation of their respective members. Majority vote by the Executive Committee will be used to resolve disputes.

1.5. Rules of authorship

The main benefit of this collaboration to participants is expediting high quality research on the effect of nebulised heparin in hospitalised COVID-19 patients. Recognition of the importance of authorship as a marker of academic effort is acknowledged in this document. As part of acknowledging this, efforts of individual participants and centres in coordination, recruitment, writing, analysis or other work to bring this work to completion will be recognised in attribution of authorship. Attribution of authorship is expected to vary by type of publication or presentations. Procedures for each are detailed below.

Publications using INHALE-HEP Meta-Trial data will have all Executive Committee members in the main authorship section, with additional authors as agreed by the Executive Committee if required/as appropriate.

Manuscripts *(those describing the core dataset)*
Manuscripts will be generated by the Writing Committee and reviewed by the Executive Committee as outlined above. The Executive Committee approved, edited, manuscript for submission will be circulated to all co-authors for comment prior to publication. Co-authors’ comments will be sent to the Writing Committee chairman for distribution within two weeks.

For all major publications the named authors will comprise the members of the Writing Committee, with the phrase ‘on behalf of the INHALE-HEP Meta-Trial Investigators’. The Chair of the Writing committee and the Chair of the Executive Committee will decide on the order of the authors, including first, corresponding and senior author, in consultation with the other members of the Writing Committee. Majority vote by the Executive Committee will be used to resolve disputes.

All INHALE-HEP Meta-Trial investigators will receive a Collaborator Credit on the Major Manuscript(s). Each clinical centre will be listed in alphabetical order, subdivided by country, each listed in alphabetical order. Unless otherwise agreed upon by centre personnel, the site coordinator will be listed first followed by a second nominated person from that centre. Members of the steering committee will be listed as collaborators on the manuscript, and separately listed in a Table in the appendix to the Document.

1.6. Abstracts

Abstracts will be generated by a Writing Committee appointed by the Executive Committee. All abstracts will be reviewed by the Executive Committee.
The approved, edited, abstract will be circulated to all co-authors for comment prior to submission.
Co- authors comments are expected to be sent to the Writing Committee chairman within two weeks.
Because of limited space in abstracts, "The INHALE-HEP Meta-Trial Investigators" will be listed as the primary author, followed by the name of a single presenter, (the chairman of the writing committee or his or her designee) written as: "presented by...".

1.7. Summary of steps in manuscript, presentation preparation:

- Idea submitted to Executive Committee chairman.
- Proposal distributed to Executive Committee and reviewed within two weeks.
    - If approved, interest in writing committee membership solicited from network PI's. (If disapproved, alternate suggestions entertained. Appeals made to Executive Committee.)
    - Executive Committee appoints Writing Committee and chairman.
- Publication or presentation draft submitted to Writing Committee within 3 months.
    - Writing Committee review draft within 4 weeks.
    - Revisions made by Writing Committee chairman.
    - Revisions reviewed by Writing Committee within two weeks.
    - Approved manuscript sent to all PIs. Comments returned within two weeks.
- Final comments incorporated by Writing Committee and Executive Committee chairmen.
- Manuscript submitted, or presentation occurs.
- Manuscript revisions, based upon journal reviewers' comments made by Writing Committee and Executive Committee chairmen in consultation with committee members
