# Supplementary material for: Efficacy of inhaled nebulised unfractionated heparin to prevent intubation or death in hospitalised patients with COVID-19: an investigator-initiated international meta-trial of randomised clinical studies
Source: eClinicalMedicine. 2025 Sep 27;88:103339. doi: 10.1016/j.eclinm.2025.103339 (PMC12572793; doi:10.1016/j.eclinm.2025.103339)
Supplement: Protocols meta-trial and individual studies [file mmc4.pdf]

Received: 14 December 2020 | Accepted: 19 December 2020

DOI: 10.1111/bcp.14714

ORIGINAL ARTICLE

INHALEd nebulised unfractionated HEParin for the treatment of hospitalised patients with COVID-19 (INHALE-HEP): Protocol and statistical analysis plan for an investigator-initiated international metatrial of randomised studies

Frank M.P. van Haren[1,2] | Alice Richardson[3] | Hwan-Jin Yoon[3] | Antonio Artigas[4] | John G. Laffey[5,6] | Barry Dixon[7] | Roger Smith[7] | Alicia B. Vilaseca[8] | Ruben A. Barbera[8] | Tarek I. Ismail[9] | Rabab S. Mahrous[10] | Mohamed Badr[11] | Gilberto De Nucci[12,13] | Carlos Sverdloff[12] | Lex M. van Loon[1] | Marta Camprubi-Rimblas[4] | David W. Cosgrave[6] | Thomas L. Smoot[14] | Sabrina Staas[14] | Khine Sann[14] | Caitlin Sas[14] | Anusha Belani[14] | Christopher Hillman[14] | Janis Shute[15] | Mary Carroll[16] | Tom Wilkinson[16] | Miles Carroll[17] | Dave Singh[18] | Clive Page[19]

[1]Australian National University, College of Health and Medicine, Canberra, Australia

[2]Faculty of Health, University of Canberra, Canberra, Australia

[3]Statistical Consulting Unit, Australian National University, Canberra, Australia

[4]Critical Center, Corporació Universitaria Sanitaria Parc Tauli, CIBER Enfermedades Respiratorias, Autonomous University of Barcelona, Sabadell, Spain

[5]Anaesthesia and Intensive Care Medicine, School of Medicine, and Regenerative Medicine Institute (REMEDI) at CÚRAM Centre for Research in Medical Devices, Biomedical Sciences Building, National University of Ireland Galway, Galway, Ireland

[6]Department of Anaesthesia, University Hospital Galway, Saolta Hospital Group, Ireland

[7]Department of Critical Care Medicine, St Vincent's Hospital, Melbourne, Australia

[8]Service of Haematology and Haemostasis, San Camilo Clinic, Buenos Aires, Argentina

[9]Department of Anaesthesia and Surgical Intensive Care, Faculty of Medicine, Helwan University, Cairo, Egypt

Aims: Inhaled nebulised unfractionated heparin (UFH) has a strong scientific and biological rationale that warrants urgent investigation of its therapeutic potential in patients with COVID-19. UFH has antiviral effects and prevents the SARS-CoV-2 virus' entry into mammalian cells. In addition, UFH has significant anti-inflammatory and anticoagulant properties, which limit progression of lung injury and vascular pulmonary thrombosis.

Methods: The INHALEd nebulised unfractionated HEParin for the treatment of hospitalised patients with COVID-19 (INHALE-HEP) metatrial is a prospective individual patient data analysis of on-going randomised controlled trials and early phase studies. Individual studies are being conducted in multiple countries. Participating studies randomise adult patients admitted to the hospital with confirmed SARS-CoV-2 infection, who do not require immediate mechanical ventilation, to inhaled nebulised UFH or standard care. All studies collect a minimum core dataset. The primary outcome for the metatrial is intubation (or death, for patients who died before intubation) at day 28. The secondary outcomes are oxygenation, clinical worsening and mortality, assessed in time-to-event analyses. Individual studies may have additional outcomes.

Analysis: We use a Bayesian approach to monitoring, followed by analysing individual patient data, outcomes and adverse events. All analyses will follow the intention-to-

PI Statement: The authors confirm that the PI for this paper is Professor Frank M.P. van Haren and that he has direct responsibility for the described metatrial. Metatrial Combined Protocol and Statistical Analysis Plan date and version: 14 December 2020, INHALE-HEP metatrial version 2.0.

 © 2021 British Pharmacological Society

[10]Department of Anaesthesia and Surgical Intensive Care, Faculty of Medicine, Alexandria University, Alexandria, Egypt
[11]Department of Critical Care Medicine, Faculty of Medicine, Helwan University, Cairo, Egypt
[12]Department of Pharmacology, Faculty of Medical Sciences, University of Campinas, Campinas, Brazil
[13]Department of Pharmacology, Institute of Biomedical Sciences, University of São Paulo, Brazil
[14]Frederick Memorial Hospital, Frederick, Maryland, USA
[15]School of Pharmacy and Biomedical Science, University of Portsmouth, Portsmouth, UK
[16]Department of Respiratory Medicine, University of Southampton, Southampton, UK
[17]National Infection Service, Public Health England, Porton Down, UK
[18]Medicines Evaluation Unit, University of Manchester, Manchester, UK
[19]Sackler Institute of Pulmonary Pharmacology, King's College London, UK

Correspondence
Frank van Haren, Australian National University, College of Health and Medicine. Canberra, Australia.
Email: frank.vanharen@anu.edu.au

treat principle, considering all participants in the treatment group to which they were assigned, except for cases lost to follow-up or withdrawn.

Trial registration, ethics and dissemination: The metatrial is registered at ClinicalTrials.gov ID NCT04635241. Each contributing study is individually registered and has received approval of the relevant ethics committee or institutional review board. Results of this study will be shared with the World Health Organisation, published in scientific journals and presented at scientific meetings.

KEYWORDS
acute respiratory distress syndrome, COVID-19, inhaled heparin, metatrial, nebulised heparin, pandemic, randomised controlled trial, respiratory failure, SARS, SARS-CoV-2, unfractionated heparin

1 | INTRODUCTION

In December 2019, a novel coronavirus (severe acute respiratory syndrome coronavirus 2, SARS-CoV-2) emerged in China and has since spread globally. Nearly 20% of patients with coronavirus disease 2019 (COVID-19) experience hypoxaemia, which is the primary reason for hospitalisation.[1] A significant proportion of patients admitted to hospital for COVID-19 develop acute respiratory failure, with 12–24% requiring intubation for invasive mechanical ventilation.[2–6]

The pathophysiology of COVID-19 associated lung injury is characterised by diffuse alveolar damage, hyperinflammation, coagulopathy, DNA neutrophil extracellular traps, hyaline membranes and microvascular thrombosis.[7]

Our group and others have previously outlined the scientific rationale for the use of nebulised unfractionated heparin (UFH) as a treatment for COVID-19.[8,9] Nebulised UFH has antiviral, anti-inflammatory, anticoagulant and mucolytic effects. Our metatrial of inhaled nebulised UFH as a repurposed drug for COVID-19 adheres to the 5 core principles and recommendations as described by the pharmacology community in its ASCEPT-BPS statement as follows.[10] Firstly, UFH has demonstrated antiviral activity in preclinical studies in concentrations relevant for administration to humans. The SARS-CoV-2 Spike S1 protein receptor binding domain attaches to UFH and undergoes conformational change that prevents it from binding to the angiotensin converting enzyme 2 (ACE-2) receptor.[11,12] It was recently demonstrated that spike protein binding to human epithelial cells requires engagement of both cell surface heparan sulfate and ACE-2, with heparan sulfate acting as a co-receptor for ACE-2 interaction, and UFH blocked the binding and infectivity of SARS-CoV-2 to human bronchial epithelial cells.[13] The inhibition of SARS-CoV-2 infection of Vero E6 cells by an UFH preparation was found to be concentration dependent, occurred at therapeutically relevant concentrations and is significantly stronger compared to low molecular weight heparins (LMWHs).[14]

Secondly, the optimal concentrations of UFH can be achieved for the proposed mode of administration in the lungs (data on file in the Investigator's Brochure).

Thirdly, in our metatrial we concurrently quantify in vivo dynamics and time course of COVID-19. Specifically, we collect and report patient-relevant clinical outcomes including rates of intubation, time course of disease progression and mortality.

Fourthly, previous studies have provided information relevant to posology optimisation for the immunomodulatory and anticoagulant effects of inhaled nebulised UFH in acute lung injury and acute respiratory distress syndrome, to ensure the appropriate intensity and timing of therapy. Animal studies of nebulised UFH in different acute lung injury models have consistently shown a positive effect on pulmonary coagulation, inflammation and oxygenation.[8] Small human studies indicate that nebulised UFH limits pulmonary fibrin deposition, attenuates progression of acute lung injury and hastens recovery.[8] Early-phase trials in patients with acute lung injury and related conditions found that nebulised UFH reduced pulmonary dead space,

coagulation activation, microvascular thrombosis, improved lung injury and increased time free of ventilatory support.[15–18] In a prepandemic double-blind randomised study in 256 critically ill ventilated patients, nebulised UFH limited progression of lung injury including acute respiratory distress syndrome and accelerated return to home in survivors.[19] The anti-inflammatory effects of inhaled UFH are thought to reduce pulmonary hyperinflammation and the generation of DNA neutrophil extracellular traps, both of which contribute to COVID-19 lung injury. The anticoagulant actions of nebulised UFH limit fibrin deposition, hyaline membrane formation and microvascular thrombosis, which are also important features of COVID-19.

Finally, our metatrial is innovative, robustly designed, and combines randomised controlled studies to determine efficacy and safety so that the benefit–harm balance of inhaled nebulised UFH is identified. We hypothesise that treatment with inhaled nebulised UFH of hospitalised patients with COVID-19 limits progression to acute respiratory failure requiring intubation, reduces the risk of death, reduces the risk of clinical worsening and improves oxygenation. The collective goal of the proposed metatrial is to reach a conclusion about the efficacy of inhaled UFH in COVID-19 as quickly as possible by pooling information from multiple clinical trials not originally configured as a network.[20] This protocol and statistical analysis plan manuscript has been prepared in accordance with the Standard Protocol Items: Recommendations for Interventional Trials (SPIRIT) 2013 guideline and in accordance with published guidelines for the content of statistical analysis plans in clinical trials (Appendix 1 and 2).[21,22]

What is already known about this subject

- Unfractionated heparin (UFH) has antiviral properties against SARS-CoV-2 at therapeutically relevant concentrations, as well as anti-inflammatory and anticoagulant effects.
- Inhaled nebulised UFH has shown to improve outcomes in prepandemic experimental and clinical studies of acute lung injury and acute respiratory distress syndrome.
- There is a strong scientific rationale for urgent investigation of the therapeutic potential of inhaled nebulised UFH for COVID-19.

What this study adds

- This metatrial is a prospective individual patient data analysis of on-going randomised trials and early phase studies, to determine whether inhaled nebulised UFH improves outcomes in hospitalised patients with COVID-19.
- The collective goal of this metatrial is to reach a conclusion about the efficacy of inhaled UFH in COVID-19 as quickly as possible by pooling information from multiple clinical trials not originally configured as a network.
- The pragmatic design effectively deals with recruitment difficulties that could occur in individual studies given the uncertainties of the international dynamics of the COVID-19 pandemic.
- Individual studies contributing to the metatrial are conducted in multiple countries, which improves effect size estimates across different conditions as well as the external validity of the results.

2 | OBJECTIVE

The primary objective of the metatrial is to investigate whether inhaled nebulised UFH in hospitalised patients with COVID-19 who do not require immediate invasive mechanical ventilation, significantly reduces rates of intubation (or death, for patients who died before intubation) at day 28, compared to standard care alone. Primary outcomes for individual studies may be different clinical or biochemical endpoints and are listed in the individual trial protocols.

3 | CONCEPT AND DESIGN

A metatrial employs prospective pooling of individual patient data from ongoing individual clinical trials and early phase studies.[20] The term *metatrial* refers to a prospective pooled analysis planned to streamline data collection from multiple individual trials, allowing for faster accumulation of data for major clinical endpoints during the pandemic.[23] The metatrial concept enables researchers to combine the agility of smaller national trials into a much larger international project in a short period of time.[23,24] Metatrial interim analysis enables detection of a positive or negative response to the scientific question as soon as an adequate sample size is reached across several countries, thus potentially speeding up the research process dramatically.[23,25,26] Adherence to methodological standards of individual trials represents a guarantee of a high level of overall final quality. Furthermore, by estimating the treatment effect across the various trials upfront, the metatrial may provide stronger evidence in favour of external validity and replicability of the individual trials. Our metatrial is designed as a collaborative prospective individual patient data analysis of on-going investigator-initiated, randomised studies of inhaled nebulised UFH in addition to standard care compared to standard care alone in hospitalised patients with confirmed COVID-19.

4 | SETTING

This metatrial includes studies of inhaled nebulised UFH in hospitalised patients with COVID-19 who do not immediately require

invasive mechanical ventilation. A full list of participating institutions is or will be made available in each individual trial record on respective trial registries. New studies from other institutions and countries may be added to this metatrial after publication of the metatrial's protocol and statistical analysis plan, provided the studies meet the eligibility criteria for the metatrial (patient eligibility criteria, intervention, core set of outcome measures).

5 | STUDY ELIGIBILITY CRITERIA

Individual studies are eligible to be included in this prospective metatrial if they meet the following requirements:

- Design: prospective randomised study with an intervention group and a control group
- Patients: inclusion and exclusion criteria as described in Table 1
- Intervention: inhaled nebulised unfractionated heparin (the dose, frequency, delivery method and treatment duration are not specified or prescribed by the metatrial)
- Data collection: able to collect and provide data required for the metatrial outcomes: intubation, death, ratio of oxygen saturation by pulse oximetry to the fraction of inspired oxygen (SpO_2/FiO_2 ratio), modified ordinal clinical scale (Table 2)[27]
- Ethics: approval of the protocols and related documents obtained from the relevant Human Research Ethics Committee (HREC) or Institutional Review Board (IRB) prior to the commencement of each individual study.

TABLE 1 Patient eligibility criteria for enrolment in studies contributing to the INHALE-HEP metatrial

Inclusion criteria	Age 18 y or older<br>Currently admitted to hospital<br>There is a PCR-positive sample for SARS-CoV-2 within the past 21 days. The sample can be a nasal or pharyngeal swab, sputum, tracheal aspirate, bronchoalveolar lavage, or another sample from the patient<br>Modified ordinal clinical scale 3–5
Exclusion criteria	Intubated and on mechanical ventilation, or requiring immediate intubation as per the treating clinician's assessment<br>Heparin allergy or heparin-induced thrombocytopaenia<br>APTT >120 s, not due to anticoagulant therapy and does not correct with administration of fresh frozen plasma<br>Platelet count <20×10^9/L<br>Pulmonary bleeding or uncontrolled bleeding<br>Pregnant or might be pregnant<br>Acute brain injury that may result in long-term disability<br>Myopathy, spinal cord injury, or nerve injury or disease with a likely prolonged incapacity to breathe independently e.g. Guillain-Barre syndrome<br>Treatment limitations in place, i.e. not for intubation, not for ICU admission<br>Death is imminent or inevitable within 24 h<br>Clinician objection<br>Refusal of participant (person responsible) consent

PCR, polymerase chain reaction; SARS-CoV-2, severe acute respiratory syndrome coronavirus 2; APTT, activated partial thromboplastin time; ICU, intensive care unit

6 | RECRUITMENT

Due to the rapidly evolving pandemic situation, we have a strong uncertainty about the pace of enrolment. There is likely to be considerable variation in the number of COVID-19 infections requiring hospitalisation in different regions. The pragmatic prespecified pooled analysis design overcomes recruitment difficulties that could occur in the individual studies given the international dynamics of the COVID-19 pandemic.

Research coordinators and investigators at each site and for each study will work with clinicians to identify potential candidates for enrolment. Logs will be maintained of patients who met the inclusion criteria but were not enrolled, with the reason for exclusion recorded on the log.

7 | INTERVENTIONS

Participants assigned to *nebulised UFH* receive nebulised UFH in addition to the standard care required as determined by the treating team. The dose, frequency, duration and delivery method differ between participating studies as follows:

- Brazil and USA: 25 000 IU UFH every 6 hours using a vibrating mesh nebuliser (Aerogen Solo), for a maximum of 21 days or until the modified ordinal scale is 1 or 2 (Table 2)
- Egypt: 1000 IU/kg predicted body weight UFH every 6 hours for 7 days using a compressed air nebuliser (Beurer IH18)

TABLE 2 Modified ordinal clinical scale for COVID-19

Modified ordinal scale	
1	Not hospitalised
2	Hospitalised, not requiring supplemental oxygen and no longer requiring medical care for COVID-19
3	Hospitalised not requiring supplemental oxygen but needing medical care for COVID-19
4	Hospitalised requiring supplemental oxygen
5	Hospitalised requiring noninvasive ventilation or high flow oxygen
6	Hospitalised requiring intubation and mechanical ventilation or ECMO
7	Death

COVID-19, coronavirus disease 2019; ECMO, extracorporeal membrane oxygenation

- Argentina: 5000 IU UFH every 8 hours for 7 days using a Venturi system connected to a full-face mask (Free Breath) fitted with an HMF antiviral expiratory filter

Participants assigned to *standard care* will receive the standard care required as determined by the treating team and will not be treated with nebulised heparin.

Nebulised UFH will be withheld if any of the following occurs:

- The treating physician deems that there is a clinically unacceptable increase in activated partial thromboplastin time (APTT)
- The treating physician deems that there is excessive bloodstaining of respiratory secretions
- There is pulmonary bleeding, major bleeding, or suspected or confirmed heparin-induced thrombocytopaenia (HIT)

Nebulised UFH will be recommenced if:

- Having been withheld because the APTT was unacceptably prolonged, the APTT becomes acceptable
- Having been withheld because there was excessive bloodstaining of upper or lower respiratory secretions, the bloodstaining of the respiratory secretions has resolved
- Having been withheld for pulmonary bleeding or major bleeding, the bleeding is definitively controlled
- Having been withheld for suspected HIT, the patient is found not to have this condition

8 | RELEVANT CONCOMITANT CARE PERMITTED OR PROHIBITED DURING THE TRIAL

Treatment with any or all of the following therapies is permitted during the participating studies and not a reason to withhold study medication: deep vein thrombosis prophylaxis with UFH or LMWH; *full* therapeutic dose UFH or LMWH for a recognised clinical indication; nonheparin anticoagulants; antithrombotic medications; protamine; prone positioning; and inhaled nitric oxide. There are no prohibitions during the trial.

9 | PROVISIONS FOR POST-TRIAL CARE

Post-trial care will be standard care through the standard healthcare system from each institution and jurisdiction in each individual study.

10 | OUTCOME DEFINITIONS

10.1 | Primary outcome

The primary outcome is intubation (or death, for patients who died before intubation) at day 28 after randomisation.

10.2 | Secondary outcomes

The secondary outcomes are:

- Survival to day 28; survival to day 60; and survival to hospital discharge, censored at day 60
- Daily ratio of oxygen saturation by pulse oximetry to the fraction of inspired oxygen (SpO_2/FiO_2 ratio, highest and lowest levels)
- Daily change in modified ordinal score from baseline to day 14
- Worsening on the modified ordinal scale (see Table 2) at 3, 7 and 14 days

10.3 | Safety outcomes

The safety outcomes are as follows:

- Number who record major bleeding. Major bleeding is defined as: bleeding that results in death and/or bleeding that is symptomatic, and occurs in a critical area or organ (intracranial, intraspinal, intraocular, retroperitoneal, intra-articular or intramuscular with compartment syndrome) and/or bleeding that results in a decrease in haemoglobin of 20 g/L or more, or results in transfusion of 2 or more units of whole blood or red cells.
- Number who record pulmonary bleeding. Pulmonary bleeding is frank bleeding in the lungs, trachea or bronchi with repeated haemoptysis or requiring repeated suctioning and associated with acute deterioration in respiratory status.
- Number who record epistaxis.
- Number who record HIT. HIT is defined as an unexplained fall in platelet count and a positive heparin antibody test.
- Number who record other adverse events and reactions. Adverse events and reactions are those that, in the site Principal Investigator's judgement, are not part of the expected clinical course and could be related (at least possibly) to the study and were medically significant or had serious sequelae for the patient.

10.4 | Process of care assessments

Process of care assessments are as follows:

- Time from hospital admission to randomisation
- Total cumulative dose of nebulised heparin
- Days of treatment with nebulised heparin
- Mean daily APTT among all participants, and among those treated with intravenous or subcutaneous unfractionated heparin, and among those not treated with intravenous or subcutaneous unfractionated heparin
- Highest APTT among all participants, and among those treated with intravenous or subcutaneous unfractionated heparin, and

among those not treated with intravenous or subcutaneous unfractionated heparin
- Days of treatment with each of the following therapies while in the study: unfractionated heparin, IV and SC; LMWH, IV and SC; lopinavir-ritonavir; remdesivir; hydroxychloroquine; interferon-β; interleukin antagonists; oseltamivir, laninamivir, zaninamivir or peramivir; macrolide; nonmacrolide antibacterial; antifungal; corticosteroid; inotrope or vasopressor infusion; and renal replacement.

10.5 | Other outcomes

Individual studies may have various other primary and secondary outcomes, which are listed in the individual study protocols.

11 | DATA COLLECTION

In each study, data will be collected by trained staff under the supervision of the site principal investigator using a case report form and data dictionary. Data will be collected at baseline, from day 0–14 (blood tests, SpO_2/FiO_2 ratio, modified ordinal scale); on day 28 and day 60 (invasive mechanical ventilation status, vital status, discharge status). The detailed list of collected data items and the schedule for data collection are described in the individual study protocols.

Deidentified individual patient data from each contributing study will be shared with the metatrial executive committee under a signed data-sharing agreement and stored in a secure data collection platform, administered by the Australian National University. Electronic information will be kept on password protected computers accessible only to authorised personnel. All study material, including case report forms and the study database, will be stored for a minimum of 15 years after the conclusion of the study or for a period as required by local laws and regulations. Any paper study material that requires disposal will be shredded using a commercial grade shredder or other means that preserves the confidentiality of participants. Any electronic data requiring disposal will be thoroughly erased from its electronic media. Each participating study will maintain a log of enrolled patients that includes patient identifiers. Patient identifiers are not transferred to the metatrial coordinating centre, but it must be possible to reidentify patients by each participating study to allow future audit against source documents.

12 | DATA MANAGEMENT

12.1 | Randomisation, allocation concealment and blinding

All contributing studies are randomised controlled studies. At randomisation, each participant is assigned to nebulised heparin or standard care in a 1-to-1 allocation ratio. Allocation concealment is performed at the level of each study as specified in the respective study protocols. All contributing studies are open label by design. The metatrial data analysts are blinded. Unblinding is permissible when prespecified Bayesian stopping rules for efficacy or safety have been met.

12.2 | Data safety monitoring board

There is no independent data safety monitoring board for the metatrial. Data safety monitoring boards are recommended for individual studies and will be specified in the protocols of the individual studies if applicable. The INHALE-HEP executive committee and the trial statisticians are responsible for Bayesian monitoring of the metatrial.

12.3 | Quality assurance monitoring

Conduct and progress of this metatrial will be monitored on an ongoing basis by the metatrial executive committee.

13 | STATISTICAL ANALYSIS

13.1 | Principles

This prospective analysis will be carried out on studies conducted in multiple countries, which increases effect size estimates across different conditions as well as the external validity of the results. We plan a prospective analysis of individual de-identified patient-level data. Common variables from all datasets will be combined to conduct the analysis.

If consent for participation is withdrawn or consent to continue is not given, the data will not be used unless consent to do so is obtained, including for all mortality time points. Analyses will be performed by intention-to-treat according to the participants' randomly allocated group, regardless of treatment compliance. These analyses will include participants for whom consent to continue is refused but the use of data already collected is allowed, including the primary outcome, and will exclude patients who do not fulfil the study entry criteria.[28]

Missing data will not be imputed. The multilevel models described in the analysis are able to handle missing data due to loss to follow-up. Where there are missing observations, the number of observations used will be reported. Two-sided hypothesis testing at a significance level of 0.05 will be used. No adjustment for multiple tests will be made, with the interpretation of the significance of the tests being appropriate for the primary or secondary nature of the outcome. Analyses will be conducted using the SPSS Research Engine, Version 24.0 IBM SPSS Statistics or later, and R version 3.5.0 or later.

13.2 | Sample size

To demonstrate a clinically important reduction in the primary outcome, a sample size of 712 is required, assuming a decrease in the

proportion of patients receiving invasive mechanical ventilation from 12 to 6%, with power 80% and a 2-sided significance level of 0.05.

Each contributing study may have a different sample size based on their primary outcome, which will have been reported on the individual trial registrations.

13.3 | Monitoring and interim analyses

We plan to perform monthly monitoring and analysis of the primary outcome in the accumulating data, with use of Bayesian monitoring rules that allow timely decisions without the penalties for multiple data looks and alpha spending associated with the classic randomised controlled trial monitoring approach.[20,29,30] At the first interim analysis, the prior distribution of the proportion of patients intubated will be multiplied by the likelihood of the observed data to give a posterior distribution of the proportion of patients intubated. At each subsequent interim analysis, the previous posterior distribution becomes the new prior, and a new posterior distribution of the proportion of patients who were intubated will be reported. The pooling of data into the prior distributions and the Bayesian updating of posterior distributions prevent the stopping rule from being overly influenced by potential bias from differential recruitment rates in different trials. Prespecified monitoring criteria will guide the recommendations of the metatrial's executive committee. If the probability of a difference in proportions of intubated patients in the 2 groups of 6% or more rises > 0.90, then the executive committee can recommend that interim analyses be conducted following the methods in the analyses section, to support a decision to stop the metatrial for efficacy. If the probability of a difference in proportions of 6% or more falls <0.10, then the executive committee can recommend that interim analyses be conducted following the methods in the analyses section, to support a decision to stop the metatrial for futility.[30,31]

13.4 | Trial profile

Patient flow through the metatrial will be presented in a Consolidated Standards of Reporting Trials diagram (Figure 1).[32] We will report the

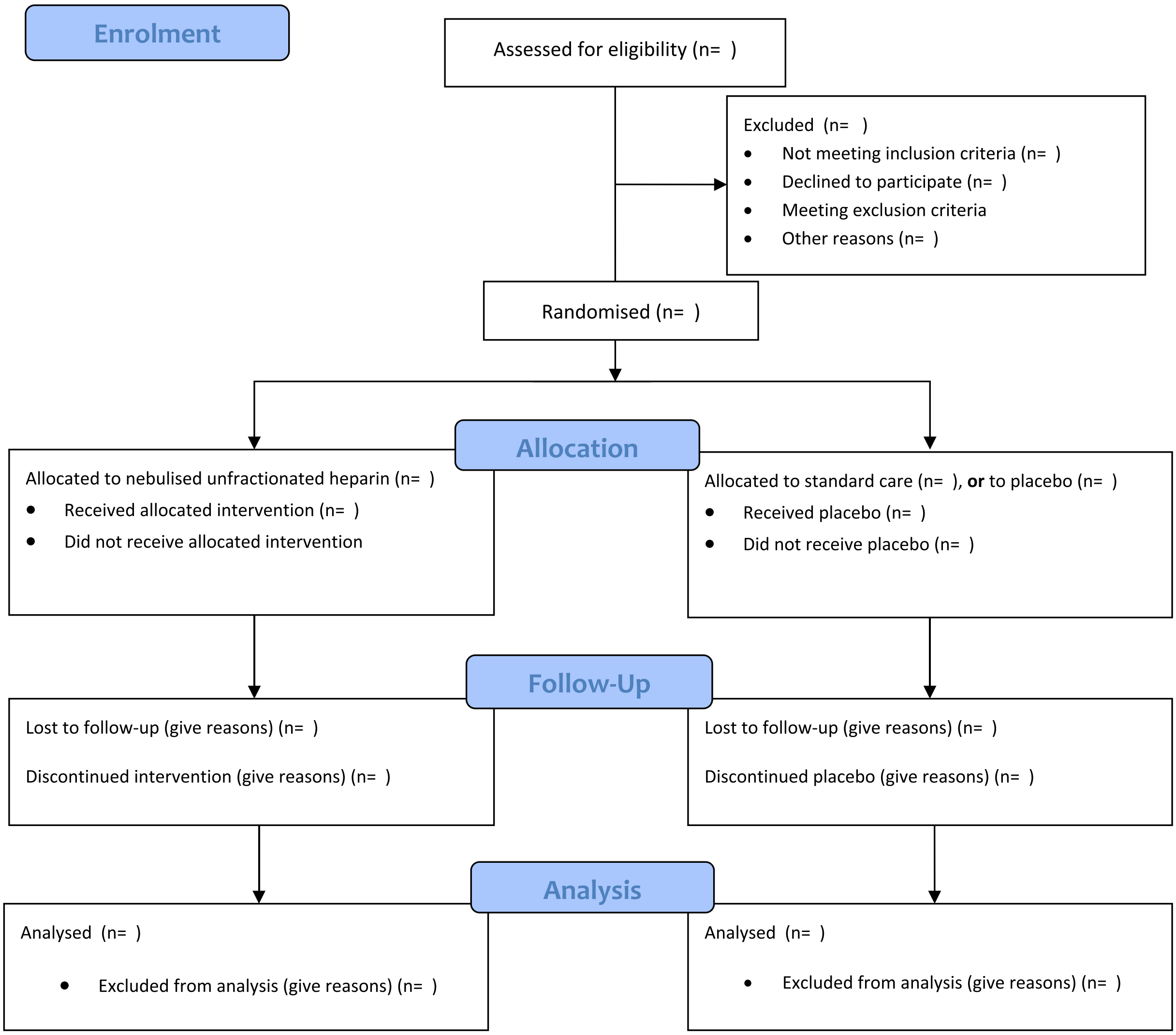

FIGURE 1 Consolidated Standards of Reporting Trials (CONSORT) diagram of participants in the INHALE-HEP metatrial

TABLE 3 Presentation of baseline characteristics of the patients

	Nebulised heparin	Control group	All patients
**Demographics**			
Age (y)	Mean ± SD, *n* =	Mean ± SD, *n* =	
Males	*n* (%), *n* =	*n* (%), *n* =	
Place of residence			
Home	*n* (%), *n* =	*n* (%), *n* =	
Nursing or chronic care facility	*n* (%), *n* =	n (%), *n* =	
**Body habitus**			
Body mass index (kg/m^2)	Mean ± SD, *n* =	Mean ± SD, *n* =	
**Comorbid conditions**			
Tobacco smoking			
Current	*n* (%), *n* =	*n* (%), *n* =	
Previous	*n* (%), *n* =	*n* (%), *n* =	
Never	*n* (%), *n* =	*n* (%), *n* =	
COPD	*n* (%), *n* =	*n* (%), *n* =	
Asthma	*n* (%), *n* =	*n* (%), *n* =	
Diabetes mellitus	*n* (%), *n* =	*n* (%), *n* =	
Hypertension	*n* (%), *n* =	*n* (%), *n* =	
**COVID-19 duration**			
Time from symptom onset (d)	Mean ± SD, *n* =	Mean ± SD, *n* =	
Time from hospital admission (d)	Mean ± SD, *n* =	Mean ± SD, *n* =	
**Other risk factors for lung injury**			
Non-COVID pulmonary sepsis	*n* (%), *n* =	*n* (%), *n* =	
Non-COVID nonpulmonary sepsis	*n* (%), *n* =	*n* (%), *n* =	
Inhalation of food or gastric contents	*n* (%), *n* =	*n* (%), *n* =	
Inhalation of smoke or airborne toxins	*n* (%), *n* =	*n* (%), *n* =	
Sternotomy, thoracotomy or laparotomy	*n* (%), *n* =	*n* (%), *n* =	
Pancreatitis			
**COVID-19 therapies**			
Lopinavir-ritonavir	*n* (%), *n* =	*n* (%), *n* =	
Remdesivir	*n* (%), *n* =	*n* (%), *n* =	
Interferon	*n* (%), *n* =	*n* (%), *n* =	
Interleukin antagonist	*n* (%), *n* =	*n* (%), *n* =	
Hydroxychloroquine	*n* (%), *n* =	*n* (%), *n* =	
Macrolide	*n* (%), *n* =	*n* (%), *n* =	
Corticosteroid	*n* (%), *n* =	*n* (%), *n* =	
**Non-COVID-19 therapies**			
Oseltamivir	*n* (%), *n* =	*n* (%), *n* =	
Antibacterial (nonmacrolide)	*n* (%), *n* =	*n* (%), *n* =	
Antifungal	*n* (%), *n* =	*n* (%), *n* =	
**Heparin therapies (IV and/or SC)**			
Unfractionated heparin	*n* (%), *n* =	*n* (%), *n* =	
Unfractionated heparin dose	Mean ± SD, *n* =	Mean ± SD, *n* =	
Enoxaparin	*n* (%), *n* =	*n* (%), *n* =	
Enoxaparin dose	Mean ± SD, *n* =	Mean ± SD, *n* =	
Tinzaparin	*n* (%), *n* =	*n* (%), *n* =	
Tinzaparin dose	Mean ± SD, *n* =	Mean ± SD, *n* =	

(Continues)

TABLE 3 (Continued)

	Nebulised heparin	Control group	All patients
Other LMWH	*n* (%), *n* =	*n* (%), *n* =	
Other LMWH dose	Mean ± SD, *n* =	Mean ± SD, *n* =	
LMWH, any type	*n* (%), *n* =	*n* (%), *n* =	

COPD, chronic obstructive pulmonary disease; COVID-19, coronavirus disease 2019; IV, intravenous; LMWH, low molecular weight heparin; SC, subcutaneous; SD, standard deviation

number of patients who meet the trial eligibility criteria, the number of patients randomised, and the number of patients in the intention-to-treat dataset for whom data are available for evaluation of the primary outcome.

13.5 | Participant characteristics and baseline comparisons

Patient characteristics at baseline will be tabulated by treatment group (Table 3). The categorical variables will be presented as frequency counts (*n*) and as a proportion of the number of patients with available data (%). Continuous variables will be presented as summary statistics for location (mean or median) and variability (standard deviation or interquartile range). The total counts for variables with missing data will be indicated.

13.6 | Analyses

13.6.1 | Primary outcome

The primary outcome is intubation (or death, for patients who died before intubation) after randomisation. This will be assessed in a time to event analysis and a regression analysis of the proportion of patients receiving intubation by day 28 after randomisation.

Because of the metatrial design, we use multilevel modelling (patients nested in sites nested in trials), with site as a random effect and trial as a fixed effect, along with testing the effect of other covariates as collected in the common variable set. The fixed effect of dose and device will also be estimated across the sites which use different combinations. The fixed effect of country can also be assessed amongst the trials which use the same dose-device combination.

We analyse binomial outcomes using multilevel logistic regression, reported as odds ratios and 95% confidence intervals. We analyse time to death using multilevel Cox proportional-hazards regression, reported as hazard ratios and 95% confidence intervals. For time to intubation, death will be treated as a competing risk. The analysis will compare the cause-specific hazard in the treatment groups using the same multilevel Cox proportional hazards model.[33] Continuous outcomes will be analysed using multilevel linear regression, reported as differences in means and 95% confidence intervals. We will present intubation to 28 days using a Kaplan–Meier survival curve and compare groups using a stratified log-rank test.

13.6.2 | Secondary outcomes

Secondary outcomes will be analysed with the same analyses as described for the primary outcome.

13.6.3 | Subgroup analyses

We plan to undertake subgroup analyses of the following variables: severity of COVID-19 (according to the PaO_2/FiO_2 ratio and the modified ordinal scale), duration of intervention, time from admission to start of intervention, time from development of symptoms to start of intervention, administration of other therapies, age and sex of the patients.

13.6.4 | Safety outcomes and adverse events

Adverse events are categorised as *not related*, *unlikely*, *possibly*, *probably* or *definitely related* to treatment, as determined by site investigators. Events will be tabulated by treatment group and reported as frequency counts (*n*) and proportions (%).

13.6.5 | Future analyses

Individual studies contributing to the metatrial may be analysed and published separately as per the original protocols of these studies. We will consider conducting hypothesis-generating exploratory analyses other than those prespecified above to further evaluate the impact of nebulised heparin on outcomes in this dataset. Any such analyses conducted after knowing the main results of the INHALE-HEP metatrial will be cautiously interpreted and clearly indicated in any subsequent publications.

13.7 | Ethics and dissemination

All contributing studies and the metatrial will be performed in accordance with the ethical principles of the Declaration of Helsinki. Approval of the protocols and related documents will be obtained from the relevant Human Research Ethics Committee (HREC) or Institutional Review Board (IRB) prior to the commencement of each individual study. These authorisations will include data inclusion in the

pooled analysis. The investigators of the individual studies will ensure that all HREC/IRB conditions for the conduct of each study are met and that all requisite information is submitted to the responsible HREC/IRB. Any protocol modifications will be communicated timely to relevant parties, including investigators and HREC/IRBs.

The individual study protocols outline the process and requirements for obtaining patients' consent to participate in their study and as required by local laws and regulations.

The results of this study will be provided to the World Health Organisation, published in peer-reviewed medical journals and presented to the medical community and other stakeholders.

13.8 | Study status

At the time of submitting for publication, the studies in Argentina, Egypt and Brazil are recruiting patients. Preparations for studies in the USA, Ireland, Italy, Pakistan, Israel, UK and Australia are underway.

14 | CONCLUSION

Nebulised UFH has a strong scientific and biological rationale, and warrants urgent investigation of its therapeutic potential for COVID-19. This investigator-initiated international individual patient data metatrial of randomised controlled trials and early phase studies investigates the efficacy and safety of nebulised UFH, on relevant outcomes in patients who are hospitalised for COVID-19. Our prespecified metatrial protocol and statistical analysis plan was prepared before completion of patient recruitment and data collection. The protocol provides a detailed description of the principles and methods for analysing and reporting the trial results and is in keeping with best research practice.

14.1 | Nomenclature of targets and ligands

Key protein targets and ligands in this article are hyperlinked to corresponding entries in http://www.guidetopharmacology.org, and are permanently archived in the Concise Guide to PHARMACOLOGY 2019/20.[34]

COMPETING INTERESTS

A.A. and M.C.R. report a research grant for preclinical research from Grifols and from Fisher&Paykel, and payment as Scientific Advisor for Grifols, outside the submitted work. J.L. reports receiving an Academic Collaboration grant funded by Science Foundation Ireland and Aerogen Inc. for a different study (CHARTER-Ireland). J.S. is Scientific Advisor for and has shares in Ockham Biotech Ltd, which holds patents around the use of inhaled heparin. T.W. is Chief Investigator of the ACCORD COVID Research Programme. D.S. reports personal fees from AstraZeneca, personal fees from Boehringer Ingelheim, personal fees from Chiesi, personal fees from Cipla, personal fees from Genentech, personal fees from GlaxoSmithKline, personal fees from Glenmark, personal fees from Gossamerbio, personal fees from Menarini, personal fees from Mundipharma, personal fees from Novartis, personal fees from Peptinnovate, personal fees from Pfizer, personal fees from Pulmatrix, personal fees from Theravance, personal fees from Verona, outside the submitted work. C.P. reports receiving personal fees from Cipla, Immune Regulation, EpiEndo and Glycosynnovation, and has equity in Verona Pharma, outside of the submitted work. All other authors have nothing to disclose.

DATA AVAILABILITY STATEMENT

The datasets used for the current manuscript are available from the corresponding author on reasonable request.

CONTRIBUTORS

Principal Investigator F.v.H. drafted the manuscript with advice from senior statisticians A.R. and H.J.Y. All authors contributed to revision and finalisation of the manuscript. All authors read and approved the final draft for submission.

ORCID

Frank M.P. van Haren https://orcid.org/0000-0001-8037-4229

REFERENCES

1. Wu Z, McGoogan JM. Characteristics of and Important Lessons From the Coronavirus Disease 2019 (COVID-19) Outbreak in China: Summary of a Report of 72314 Cases From the Chinese Center for Disease Control and Prevention. *JAMA*. 2020;323(13):1239-1242.
2. Grasselli G, Zangrillo A, Zanella A, et al. Baseline Characteristics and Outcomes of 1591 Patients Infected With SARS-CoV-2 Admitted to ICUs of the Lombardy Region, Italy. *JAMA*. 2020;323(16):1574-1581.
3. Wu C, Chen X, Cai Y, et al. Risk Factors Associated With Acute Respiratory Distress Syndrome and Death in Patients With Coronavirus Disease 2019 Pneumonia in Wuhan, China. *JAMA Intern Med*. 2020;180(7):934-943.
4. Wang D, Hu B, Hu C, et al. Clinical Characteristics of 138 Hospitalized Patients With 2019 Novel Coronavirus-Infected Pneumonia in Wuhan, China. *JAMA*. 2020;323(11):1061-1069.
5. Richardson S, Hirsch JS, Narasimhan M, et al. Presenting Characteristics, Comorbidities, and Outcomes Among 5700 Patients Hospitalized With COVID-19 in the New York City Area. *JAMA*. 2020;323(20):2052-2059.
6. Petrilli CM, Jones SA, Yang J, et al. Factors associated with hospital admission and critical illness among 5279 people with coronavirus disease 2019 in New York City: prospective cohort study. *BMJ*. 2020;369:m1966.
7. Ackermann M, Verleden SE, Kuehnel M, et al. Pulmonary Vascular Endothelialitis, Thrombosis, and Angiogenesis in Covid-19. *N Engl J Med*. 2020;383(2):120-128.
8. van Haren FMP, Page C, Laffey JG, et al. Nebulised heparin as a treatment for COVID-19: scientific rationale and a call for randomised evidence. *Crit Care*. 2020;24(1):454. https://doi.org/10.1186/s13054-020-03148-2
9. Conzelmann C, Muller JA, Perkhofer L, et al. Inhaled and systemic heparin as a repurposed direct antiviral drug for prevention and treatment of COVID-19. *Clin Med (Lond)*. 2020;20(6):e218-e221.

10. Baker EH, Gnjidic D, Kirkpatrick CMJ, Pirmohamed M, Wright DFB, Zecharia AY. A call for the appropriate application of clinical pharmacological principles in the search for safe and efficacious COVID-19 (SARS-COV-2) treatments. *Br J Clin Pharmacol*. 2020;1-5. https://doi.org/10.1111/bcp.14416
11. Mycroft-West C, Su D, Pagani I, et al. Heparin Inhibits Cellular Invasion by SARS-CoV-2: Structural Dependence of the Interaction of the Spike S1 Receptor-Binding Domain with Heparin. *Thromb Haemost*. 2020;120(12):1700-1715. https://doi.org/10.1055/s-0040-1721319
12. Kwon PS, Oh H, Kwon SJ, et al. Sulfated polysaccharides effectively inhibit SARS-CoV-2 in vitro. *Cell Discov*. 2020;6(1):50. https://doi.org/10.1038/s41421-020-00192-8
13. Clausen TM, Sandoval DR, Spliid CB, et al. SARS-CoV-2 Infection Depends on Cellular Heparan Sulfate and ACE2. *Cell*. 2020;183(4):1043-1057.e15. https://doi.org/10.1016/j.cell.2020.09.033
14. Tree JA, Turnbull JE, Buttigieg KR, et al. Unfractionated heparin inhibits live wild-type SARS-CoV-2 cell infectivity at therapeutically relevant concentrations. *Br J Pharmacol*. 2021;178:626-635. https://doi.org/10.1111/bph.15304
15. Dixon B, Schultz MJ, Hofstra JJ, Campbell DJ, Santamaria JD. Nebulized heparin reduces levels of pulmonary coagulation activation in acute lung injury. *Crit Care*. 2010;14(5):445. https://doi.org/10.1186/cc9269
16. Dixon B, Campbell DJ, Santamaria JD. Elevated pulmonary dead space and coagulation abnormalities suggest lung microvascular thrombosis in patients undergoing cardiac surgery. *Intensive Care Med*. 2008;34(7):1216-1223.
17. Dixon B, Schultz MJ, Smith R, Fink JB, Santamaria JD, Campbell DJ. Nebulized heparin is associated with fewer days of mechanical ventilation in critically ill patients: a randomized controlled trial. *Crit Care*. 2010;14(5):R180. https://doi.org/10.1186/cc9286
18. Dixon B, Smith R, Santamaria JD, et al. A trial of nebulised heparin to limit lung injury following cardiac surgery. *Anaesth Intensive Care*. 2016;44(1):28-33.
19. Dixon B, Smith R, Campbell D, et al. Nebulised heparin for patients with or at risk of acute respiratory distress syndrome: a multicentre, randomised, double-blind, placebo-controlled phase 3 trial. *Lancet Respir Med*. [accepted for publication 05 October 2020; in-press]. https://doi.org/10.1016/S2213-2600(20)30470-7
20. Petkova E, Antman EM, Troxel AB. Pooling Data From Individual Clinical Trials in the COVID-19 Era. *JAMA*. 2020;324(6):543-545.
21. Chan AW, Tetzlaff JM, Altman DG, et al. SPIRIT 2013 statement: defining standard protocol items for clinical trials. *Ann Intern Med*. 2013;158(3):200-207.
22. Gamble C, Krishan A, Stocken D, et al. Guidelines for the Content of Statistical Analysis Plans in Clinical Trials. *JAMA*. 2017;318(23):2337-2343.
23. Li J, Pavlov I, Laffey JG, et al. Meta-trial of awake prone positioning with nasal high flow therapy: Invitation to join a pandemic collaborative research effort. *J Crit Care*. 2020;60:140-142.
24. Tavernier E, Trinquart L, Giraudeau B. Finding Alternatives to the Dogma of Power Based Sample Size Calculation: Is a Fixed Sample Size Prospective Meta-Experiment a Potential Alternative? *PLoS ONE*. 2016;11(6):e0158604.
25. Simonsen L, Higgs E, Taylor RJ. Clinical research networks are key to accurate and timely assessment of pandemic clinical severity. *Lancet Glob Health*. 2018;6(9):e956-e957.
26. Memoli MJ. Pandemic research in the ICU: always be prepared. *Crit Care Med*. 2013;41(4):1147-1148.
27. Characterisation WHOWGotC, Management of C-i. A minimal common outcome measure set for COVID-19 clinical research. *Lancet Infect Dis*. 2020;20(8):e192-e197.
28. Fergusson D, Aaron SD, Guyatt G, Hebert P. Post-randomisation exclusions: the intention to treat principle and excluding patients from analysis. *BMJ*. 2002;325(7365):652-654.
29. Lewis RJ, Angus DC. Time for Clinicians to Embrace Their Inner Bayesian?: Reanalysis of Results of a Clinical Trial of Extracorporeal Membrane Oxygenation. *JAMA*. 2018;320(21):2208-2210.
30. Saville BR, Connor JT, Ayers GD, Alvarez J. The utility of Bayesian predictive probabilities for interim monitoring of clinical trials. *Clin Trials*. 2014;11(4):485-493.
31. Pedroza C, Tyson JE, Das A, et al. Advantages of Bayesian monitoring methods in deciding whether and when to stop a clinical trial: an example of a neonatal cooling trial. *Trials*. 2016;17:335. https://doi.org/10.1186/s13063-016-1480-4
32. Schulz KF, Altman DG, Moher D, Group C. CONSORT 2010 statement: updated guidelines for reporting parallel group randomised trials. *BMJ*. 2010;340(mar23 1):c332.
33. Stone JH, Frigault MJ, Serling-Boyd NJ, et al. Efficacy of Tocilizumab in Patients Hospitalized with Covid-19. *N Engl J Med*. 2020;383(24):2333-2344.
34. Alexander SPH, Kelly E, Mathie A, et al. The Concise Guide to PHARMACOLOGY 2019/20: Introduction and Other Protein Targets. *Br J Pharmacol*. 2019;176:S1-S20.

How to cite this article: van Haren FMP, Richardson A, Yoon H-J, et al. INHALEd nebulised unfractionated HEParin for the treatment of hospitalised patients with COVID-19 (INHALE-HEP): Protocol and statistical analysis plan for an investigator-initiated international metatrial of randomised studies. *Br J Clin Pharmacol*. 2021;87:3075–3091. https://doi.org/10.1111/bcp.14714

APPENDIX 1

Reporting checklist for protocol of a clinical trial

Protocol: INHALEd nebulized unfractionated HEParin for the treatment of hospitalised patients with COVID-19 (INHALE-HEP): Protocol for an investigator-initiated international metatrial of randomised studies.

Based on the SPIRIT guidelines.

Instructions to authors

Complete this checklist by entering the page numbers from your manuscript where readers will find each of the items listed below.

Your article may not currently address all the items on the checklist. Please modify your text to include the missing information. If you are certain that an item does not apply, please write "n/a" and provide a short explanation.

Upload your completed checklist as an extra file when you submit to a journal.

In your methods section, say that you used the SPIRITreporting guidelines, and cite them as:

Chan A-W, Tetzlaff JM, Altman DG, Laupacis A, Gøtzsche PC, Krleža-Jerić K, Hróbjartsson A, Mann H, Dickersin K, Berlin J, Doré C, Parulekar W, Summerskill W, Groves T, Schulz K, Sox H, Rockhold FW, Rennie D, Moher D. SPIRIT 2013 Statement: defining standard protocol items for clinical trials. Ann Intern Med. 2013;158(3):200–207.

		Reporting item	Page number
**Administrative information**			
Title	1	Descriptive title identifying the study design, population, interventions, and, if applicable, trial acronym	1
Trial registration	2a	Trial identifier and registry name. If not yet registered, name of intended registry	8
Trial registration: Data set	2b	All items from the World Health Organization trial registration data set	Yes
Protocol version	3	Date and version identifier	4
Funding	4	Sources and types of financial, material, and other support	4
Roles and responsibilities: Contributorship	5a	Names, affiliations, and roles of protocol contributors	1–4
Roles and responsibilities: Sponsor contact information	5b	Name and contact information for the trial sponsor	4
Roles and responsibilities: Sponsor and funder	5c	Role of study sponsor and funders, if any, in study design; collection, management, analysis, and interpretation of data; writing of the report; and the decision to submit the report for publication, including whether they will have ultimate authority over any of these activities	4
Roles and responsibilities: Committees	5d	Composition, roles, and responsibilities of the coordinating Centre, steering committee, endpoint adjudication committee, data management team, and other individuals or groups overseeing the trial, if applicable (see item 21a for data monitoring committee)	4
**Introduction**			
Background and rationale	6a	Description of research question and justification for undertaking the trial, including summary of relevant studies (published and unpublished) examining benefits and harms for each intervention	9/11
Background and rationale: Choice of comparators	6b	Explanation for choice of comparators	N/A
Objectives	7	Specific objectives or hypotheses	11
Trial design	8	Description of trial design including type of trial (e.g. parallel group, crossover, factorial, single group), allocation ratio, and framework (e.g. superiority, equivalence, noninferiority, exploratory)	12

(Continues)

		Reporting item	Page number
**Methods: Participants, interventions, and outcomes**			
Study setting	9	Description of study settings (e.g. community clinic, academic hospital) and list of countries where data will be collected. Reference to where list of study sites can be obtained	12/13
Eligibility criteria	10	Inclusion and exclusion criteria for participants. If applicable, eligibility criteria for study centres and individuals who will perform the interventions (e.g. surgeons, psychotherapists)	13 and Table 1
Interventions: Description	11a	Interventions for each group with sufficient detail to allow replication, including how and when they will be administered	14
Interventions: Modifications	11b	Criteria for discontinuing or modifying allocated interventions for a given trial participant (e.g. drug dose change in response to harms, participant request, or improving/worsening disease)	14–15
Interventions: Adherance	11c	Strategies to improve adherence to intervention protocols, and any procedures for monitoring adherence (e.g. drug tablet return; laboratory tests)	17
Interventions: Concomitant care	11d	Relevant concomitant care and interventions that are permitted or prohibited during the trial	15
Outcomes	12	Primary, secondary, and other outcomes, including the specific measurement variable (e.g. systolic blood pressure), analysis metric (e.g. change from baseline, final value, time to event), method of aggregation (e.g. median, proportion), and time point for each outcome. Explanation of the clinical relevance of chosen efficacy and harm outcomes is strongly recommended	15–18
Participant timeline	13	Time schedule of enrolment, interventions (including any run-ins and washouts), assessments, and visits for participants. A schematic diagram is highly recommended (see Figure 1)	14
Sample size	14	Estimated number of participants needed to achieve study objectives and how it was determined, including clinical and statistical assumptions supporting any sample size calculations	20
Recruitment	15	Strategies for achieving adequate participant enrolment to reach target sample size	13/14
**Methods: Assignment of interventions (for controlled trials)**			
Allocation: Sequence generation	16a	Method of generating the allocation sequence (e.g. computer-generated random numbers), and list of any factors for stratification. To reduce predictability of a random sequence, details of any planned restriction (e.g. blocking) should be provided in a separate document that is unavailable to those who enrol participants or assign interventions	19
Allocation concealment mechanism	16b	Mechanism of implementing the allocation sequence (e.g. central telephone; sequentially numbered, opaque, sealed envelopes), describing any steps to conceal the sequence until interventions are assigned	19
Allocation: Implementation	16c	Who will generate the allocation sequence, who will enrol participants, and who will assign participants to interventions	19
Blinding (masking)	17a	Who will be blinded after assignment to interventions (e.g. trial participants, care providers, outcome assessors, data analysts), and how	19
Blinding (masking): Emergency unblinding	17b	If blinded, circumstances under which unblinding is permissible, and procedure for revealing a participant's allocated intervention during the trial	19
**Methods: Data collection, management, and analysis**			
Data collection plan	18a	Plans for assessment and collection of outcome, baseline, and other trial data, including any related processes to promote data quality (e.g. duplicate measurements, training of assessors) and a description of study instruments (e.g. questionnaires, laboratory tests) along with their reliability and validity, if known. Reference to where data collection forms can be found, if not in the protocol	18
Data collection plan: Retention	18b	Plans to promote participant retention and complete follow-up, including list of any outcome data to be collected for	18

(Continues)

		Reporting item	Page number
		participants who discontinue or deviate from intervention protocols	
Data management	19	Plans for data entry, coding, security, and storage, including any related processes to promote data quality (e.g. double data entry; range checks for data values). Reference to where details of data management procedures can be found, if not in the protocol	18
Statistics: Outcomes	20a	Statistical methods for analysing primary and secondary outcomes. Reference to where other details of the statistical analysis plan can be found, if not in the protocol	19/24
Statistics: Additional analyses	20b	Methods for any additional analyses (e.g. subgroup and adjusted analyses)	23/24
Statistics: Analysis population and missing data	20c	Definition of analysis population relating to protocol nonadherence (e.g. as randomised analysis), and any statistical methods to handle missing data (e.g. multiple imputation)	19/20
**Methods: Monitoring**			
Data monitoring: Formal committee	21a	Composition of data monitoring committee (DMC); summary of its role and reporting structure; statement of whether it is independent from the sponsor and competing interests; and reference to where further details about its charter can be found, if not in the protocol. Alternatively, an explanation of why a DMC is not needed	19
Data monitoring: Interim analysis	21b	Description of any interim analyses and stopping guidelines, including who will have access to these interim results and make the final decision to terminate the trial	21
Harms	22	Plans for collecting, assessing, reporting, and managing solicited and spontaneously reported adverse events and other unintended effects of trial interventions or trial conduct	16/17
Auditing	23	Frequency and procedures for auditing trial conduct, if any, and whether the process will be independent from investigators and the sponsor	17
**Ethics and dissemination**			
Research ethics approval	24	Plans for seeking research ethics committee/institutional review board (REC/IRB) approval	24
Protocol amendments	25	Plans for communicating important protocol modifications (e.g. changes to eligibility criteria, outcomes, analyses) to relevant parties (e.g. investigators, REC/IRBs, trial participants, trial registries, journals, regulators)	24
Consent or assent	26a	Who will obtain informed consent or assent from potential trial participants or authorised surrogates, and how (see item 32)	24
Consent or assent: Ancillary studies	26b	Additional consent provisions for collection and use of participant data and biological specimens in ancillary studies, if applicable	N/A
Confidentiality	27	How personal information about potential and enrolled participants will be collected, shared, and maintained in order to protect confidentiality before, during, and after the trial	18
Declaration of interests	28	Financial and other competing interests for principal investigators for the overall trial and each study site	4/5
Data access	29	Statement of who will have access to the final trial dataset, and disclosure of contractual agreements that limit such access for investigators	4
Ancillary and post-trial care	30	Provisions, if any, for ancillary and post-trial care, and for compensation to those who suffer harm from trial participation	15
Dissemination policy: Trial results	31a	Plans for investigators and sponsor to communicate trial results to participants, healthcare professionals, the public, and other relevant groups (e.g. via publication, reporting in results databases, or other data sharing arrangements), including any publication restrictions	24
Dissemination policy: Authorship	31b	Authorship eligibility guidelines and any intended use of professional writers	4
	31c		4

(Continues)

		Reporting item	Page number
Dissemination policy: Reproducible research		Plans, if any, for granting public access to the full protocol, participant-level dataset, and statistical code	
**Appendices**			
Informed consent materials	32	Model consent form and other related documentation given to participants and authorised surrogates	N/A
Biological specimens	33	Plans for collection, laboratory evaluation, and storage of biological specimens for genetic or molecular analysis in the current trial and for future use in ancillary studies, if applicable	N/A

The SPIRIT checklist is distributed under the terms of the Creative Commons Attribution License CC-BY-ND 3.0. This checklist can be completed online using https://www.goodreports.org/, a tool made by the EQUATOR Network in collaboration with Penelope.ai

APPENDIX 2

Statistical Analysis Plan (SAP) Checklist v 1.02019

Section/item	Index	Description	Reported on page
**Section 1: Administrative information**			
Trial and trial registration	1a	Descriptive title that matches the protocol, with SAP either as a forerunner or subtitle, And trial acronym (if applicable)	1
	1b	Trial registration number	7/8
SAP version	2	SAP version number with dates	4
Protocol version	3	Reference to version of protocol being used	4
SAP revisions	4a	SAP revision history	N/A
	4b	Justification for each SAP revision	N/A
	4c	Timing of SAP revisions in relation to interim analyses, etc.	N/A
Roles and responsibility	5	Names, affiliations, and roles of SAP contributors	1–3
Signatures of:	6a	Person writing the SAP	4
	6b	Senior statistician responsible	4
	6c	Chief investigator/clinical lead	4
**Section 2: Introduction**			
Background and rationale	7	Synopsis of trial background and rationale including a brief description of research question And brief justification for undertaking the trial	9–11
Objectives	8	Description of specific objectives or hypotheses	11
**Section 3: Study methods**			
Trial design	9	Brief description of trial design including type of trial (e.g. parallel group, multi-arm, crossover, factorial) And allocation ratio and may include brief description of interventions	11/12
Randomization	10	Randomization details, e.g. whether any minimization or stratification occurred (including stratifying factors used or the location of that information if it is not held within the SAP)	19
Sample size	11	Full sample size calculation or reference to sample size calculation in protocol	20

(Continues)

Section/item	Index	Description	Reported on page
		(instead of replication in SAP)	
Framework	12	Superiority, equivalence, or noninferiority hypothesis testing framework, including which comparisons will be presented on this basis	11
Statistical interim analysis and stopping guidance	13a	Information on interim analyses specifying what interim analyses will be carried out And listing of time points	21
	13b	Any planned adjustment of the significance level due to interim analysis	21
	13c	Details of guidelines for stopping the trial early	21
Timing of final analysis	14	Timing of final analysis, e.g. all outcomes analysed collectively or timing stratified By planned length of follow-up	21
Timing of outcome assessments	15	Time points at which the outcomes are measured including visit windows	22/23
**Section 4: Statistical principals**			
Confidence intervals and *P* values	16	Level of statistical significance	20
	17	Description and rationale for any adjustment for multiplicity and, if so, detailing how the type 1 error is to be controlled	20
	18	Confidence intervals to be reported	22
Adherence and protocol deviations	19a	Definition of adherence to the intervention and how this is assessed including extent Of exposure	17
	19b	Description of how adherence to the intervention will be presented	17
	19c	Definition of protocol deviations for the trial	17
	19d	Description of which protocol deviations will be summarized	N/A
Analysis populations	20	Definition of analysis populations, e.g. intention to treat, per protocol, Complete case, safety	20
**Section 5: Trial population**			
Screening data	21	Reporting of screening data (if collected) to describe representativeness Of trial sample	21, Figure 1
Eligibility	22	Summary of eligibility criteria	13; Table 1
Recruitment	23	Information to be included in the CONSORT flow diagram	Figure 1
Withdrawal/follow-up	24a	Level of withdrawal, e.g. from intervention and/or from follow-up	Figure 1
	24b	Timing of withdrawal/lost to follow-up data	Figure 1
	24c	Reasons and details of how withdrawal/lost to follow-up data will be presented	Figure 1
Baseline patient characteristics	25a	List of baseline characteristics to be summarized	Table 3
	25b	Details of how baseline characteristics will be descriptively summarized	Table 3
**Section 6: Analysis**			
Outcome definitions		List and describe each primary and secondary outcome including details of:	
	26a	Specification of outcomes and timings. If applicable include the order of importance of primary Or key secondary end points (e.g. order in which they will be tested)	22/23
	26b	Specific measurement and units (e.g. glucose control, hbA1c [mmol/mol or %])	22/23
	26c	Any calculation or transformation used to derive the outcome (e.g. change from baseline, QoL score, time to event, logarithm etc.)	22/23
Analysis methods	27a	What analysis method will be used and how the treatment effects will be presented	22/23
	27b	Any adjustment for covariates	22/23
	27c	Methods used for assumptions to be checked for statistical methods	N/A
	27d	Details of alternative methods to be used if distributional assumptions do not hold, e.g. normality, proportional hazards, etc.	N/A

(Continues)

Section/item	Index	Description	Reported on page
	27e	Any planned sensitivity analyses for each outcome where applicable	N/A
	27f	Any planned subgroup analyses for each outcome including how subgroups are defined	23
Missing data	28	Reporting and assumptions/statistical methods to handle missing data (e.g. multiple imputation)	20
Additional analyses	29	Details of any additional statistical analyses required, e.g. complier-average causal effect10 analysis	N/A
Harms	30	Sufficient detail on summarizing safety data, e.g. information on severity, expectedness, and causality; details of how adverse events are coded or categorized; how adverse event data will be analysed, i.e., grade 3/4 only, incidence case analysis, intervention emergent analysis	16/17
Statistical software	31	Details of statistical packages to be used to carry out analyses	20
References	32a	References to be provided for nonstandard statistical methods	Yes
	32b	Reference to data management plan	N/A
	32c	Reference to the trial master file and statistical master file	N/A
	32d	Reference to other standard operating procedures or documents to be adhered to	Yes (data sharing agreement)

Taken from the paper: Gamble C, Krishan A, Stocken D, Lewis S, Juszczak E, Doré C, et al. Guidelines for the content of statistical analysis plans in clinical trials. JAMA. 2017;318(23):2337–43.

Abbreviations: CONSORT, Consolidated Standards of Reporting Trials; hbA1c, haemoglobin A1c; QoL, quality of life; SAP, statistical analysis plan.

For more information visit:

The development of this checklist was funded by the MRC Hubs for Trials Methodology Research

PROTOCOL

Efficacy and safety study to evaluate the use of inhalation heparin in patients with COVID-19 pneumonia.

Prospective, randomized, comparative trial

against standard treatment.

HEPANEBU study

Version 1.0

Release date: May 2020

Dr Barbera RF[1]; Dr Mymicopulo Llambia A[1]; Dr Orden A.O [1]; Lic.Hna González Rios L.G[2]; Dr. Vilaseca A.B[3]

1.Service of Internal Medicine San Camilo Clinic Buenos Aires Argentina

2.Department of Nursing San Camilo Clinic Buenos Aires Argentina

3.Service of Hematology and Haemostasis San Camilo Clinic Buenos Aires Argentina

Contact avilaseca@clinicasancamilo.org.ar

This protocol should be treated as a confidential document.

A written authorization must be obtained from your manager

SCIENTIFIC DESIGN OF THE PROTOCOL

Dr. Vilaseca Alicia

Principal investigator

Hematologist Physician

San Camilo Clinical Hematology Service Buenos Aires Argentina

Dr Barbera RF

Sub-investigator

Doctor

Medical Clinic Service Clínica San Camilo Buenos Aires Argentinaif any part of this protocol is going to be presented or published.

STUDY SYNOPSIS

HEPANEBU protocol code

Monitor Center: San Camilo Clinic

CABA-Argentina

Study title: Efficacy and safety study to evaluate the use of inhalation heparin in patients with COVID-19 pneumonia.

Version number and date V. 1.0 - May 2020

Type of study: Trial, prospective, randomized, comparative against standard treatment.

Justification: The emergence of COVID-19 requires the urgent development of strategies to avoid the impact of the disease on our population, the saturation of the health system and the mortality of the disease.

Severe acute respiratory syndrome coronavirus 2 (SARS-CoV-2) was first reported in Wuhan, Hubei province, China and has subsequently spread to the world population. Factors associated with the development of SARS and its mortality include advanced age, lymphopenia, organ dysfunction, and bleeding disorders. (1-3)

Different manifestations have been described (deep vein thrombosis, pulmonary thromboembolism, digital ischemia and cerebral infarcts), and different mechanisms, such as the presence of antiphospholipid antibodies in COVID-19. There is evidence of the presence of a hypercoagulable state in the majority of deaths from SARS associated with COVID -19. (4-6)

Increased plasma D-dimer concentrations is a common finding and also appears to be an independent predictor of mortality. These patients and those who meet criteria for sepsis-induced coagulopathy (SIC) would benefit from anticoagulant therapy primarily with low molecular weight heparin (LMWH). (4-6)

Antithrombotic therapies have been used in clinical practice for almost a century. In clinical practice, unfractionated heparin (UFH) and heparin derivatives remain the predominant antithrombotic therapies administered parenterally.

Heparin binds to antithrombin III (AT-III), a plasma glycoprotein, and to a small extent also to the heparin II cofactor. The result of this binding produces a conformational change and a strong increase in the inhibitory effect of thrombin, which becomes approximately 1000 times more potent than before. Other targets of heparin on coagulation are the inhibition or reduced activation of factors V, VIII and IX and the inhibition of thrombocyte function, due to a nonspecific binding of platelet factor IV. (7)

However, heparin is a drug not only with anticoagulant properties, it has many other properties (interaction with growth factors, regulation of cell proliferation and angiogenesis, modulation of proteases and antiproteases), making it an interesting subject of research in the

field of inflammation, allergy and immunology, interstitial lung fibrosis and oncology. Inhalation of heparin produces local anti-inflammatory and antifibrotic effects (7-10). In addition, possible effects have been described to prevent viral infection, including coronaviridae (11), especially the current work published by Mark Skidmore et al. Where he describes the capacity of SARS-CoV-2 S1 RBD to bind heparin. Such binding capacity is an important prerequisite for research related to the development of SARS-CoV-2 inhalation unfractionated heparin therapeutics. (12)

Experimental studies of inhaled UFH in healthy subjects showed that doses of less than 32,000 IU of UFH through the lower respiratory tract were safe (13-15). In a prospective cohort study in young adults, Harenberg determined that the inhaled dose of LMWH had to be 10 times greater than that administered subcutaneously to achieve similar levels of anti-factor Xa assay and Heptest. (16)

Considering the role of coagulopathy and inflammation in the induction of ventilator-induced lung injury, nebulized heparin improved lung function in ventilated patients, equivalent to the use of corticosteroids. (17) It has also been compared with other interventions to stimulate the fibrinolysis or block coagulation to suppress the inflammatory response and reduce lung injury in adult acute respiratory distress syndrome (18) from health personnel contagion.

Working hypothesis: The combination of inhalational heparin combined with prophylactic doses of LMWH reduces the progression to severe forms of the disease, and consequently the requirement for intensive care units and mechanical ventilation.

Main objective: The objective of this work is to evaluate the safety and efficacy of the use of inhalational heparin in patients with pulmonary involvement / pneumonia / SARS associated with COVID-19, laboratory with marked inflammation parameters, and prothrombotic state secondary to it (Fibrinogen , Ferritin and / or D-Dimer elevated), from admission to hospitalization.

Study variables: Comparison between both treatment groups of the following variables:

Effectiveness

Primary variables:

• Need to transfer the patient to the Intensive Care Unit (ICU)

Secondary variables:

• ARM requirement

• Days of stay in ICU.

• Days of hospitalization.

• Discharge or death

Variables of improvement of severity indicators, reduction of the SOFA scale (reduction ≥ 2 points), improvement in PafI, days in ICU, days of hospitalization will be analyzed.

Security

Security variables

- Venous and arterial thrombotic events
- Major bleeding
- Minor bleeding
- Heparin-induced thrombocytopenia
- Requirement for transfusion and blood products

The patient will be clinically monitored on a daily basis and every 48 hours with laboratory parameters and imaging studies, until discharge or reaching the primary end point.

Population: Hospitalized patients with a diagnosis of moderate to severe SARS-CoV-2 will be included.

Eligibility criteria: Inclusion:

Persons over 18 years of age of any sex admitted with a diagnosis of a suspected case of COVID-19, in accordance with the definition of the Ministry of Health of the Nation (MSal) as of May 20, 2020, who present at the time of admission or in its evolution compatible pulmonary infiltrates on imaging studies (chest X-ray or chest CT) and at least one of the following biochemical parameters of systemic inflammation:

D Dimero >1ng/dl

Ferritin > 500 ng/ml

Fibrinogen > 500 mg/dl

Exclusion:

- Under 18 years old
- Pregnant women
- Known allergy to Heparin
- Participant in another clinical trial that is not approved for joint enrollment.
- APTT> 120 seconds, not due to anticoagulant therapy.
- Platelet count <20 x 109 per L
- Lung bleeding.
- Uncontrolled bleeding

• Advanced neurological impairment

• Advanced oncological disease

Discontinuation:

All those subjects who develop a serious or severe adverse drug reaction that, at the discretion of the investigator, puts the subject under study at risk will be discontinued.

Treatment to be used: Patients will be randomized to receive one of the 2 treatments under study: standard treatment + Unfractionated Heparin (UFH) in inhalation form or standard treatment alone. The randomization will be blind to the investigator although the treatment will be open once assigned.

Treatment under study:

Standard treatment + Unfractionated Heparin (UFH) by inhalation in a dose of 5000IU every 8hours, diluted in 0.5 cc of Physiological Solution for 7 days or until transfer to ICU.

Comparator treatment:

- Standard treatment for 7 days or until transfer to ICU.

Standard treatment: All patients will receive antibiotic treatment, specific treatment and adjuvant treatment according to the recommendations in force at the time of admission. All these treatments will be recorded in the protocol for later analysis.

Standard treatment includes LMWH low molecular weight heparin at a prophylactic dose:

Prophylaxis with LMWH: 40 mg sc every 24 hours, adjusted for weight if it is> 100 kg (60 mg sc every 24 hours), or <50 kg in women (20 mg sc every 24 hours).

Study design:

Prospective, randomized study design to evaluate the effects of the administration of Unfractionated Heparin (UFH) in inhalation form added to standard treatment, versus standard treatment in patients diagnosed with moderate and severe SARS-COVID-19. Standard treatment includes prophylactic-dose Low Molecular Weight Heparin (LMWH).

Admitted patients will be clinically monitored daily and every 48 hours with laboratory parameters and imaging studies, until discharge or reaching the primary end point.

The study is carried out in accordance with the principles of the Helsinki Declaration and the International Conference on Harmonization of Good Clinical Practice Guidelines and was approved by the Institutional Authorities.

Device to nebulize without producing aerosolization:

To administer heparin in inhalation form we have designed a device modifying a full face snorkel mask, in which instead of the discharge valve a connector for the Venturi has been placed, and in the air outlet / inlet of the snorkel it has been adapted a connector made with 3D printing for the insertion of a disposable antiviral filter (filters commonly used in Mechanical Respiratory Assistance devices).

The mask is made of materials that allow its sterilization with the STERRAT Hydrogen Peroxide plasma system, available at the institution.

Bibliografía

1. Zhou Y. Zhang Z. Tian J. Xiong S. Risk factors associated with disease progression in a cohort of patients infected with the 2019 novel coronavirus. Ann Palliat Med. 2020; 9(2):428–436.

2. Wu C. Chen X. Cai Y. Xia J. Zhou X. Xu S. Huang H. Zhang L. Zhou X. Du C. Zhang Y. Song J. Wang S. Chao Y. Yang Z. Xu J. Zhou X. Chen D. Xiong W. Xu L. Zhou F. Jiang J. Bai C. Zheng J. Song Y. Risk Factors Associated With Acute Respiratory Distress Syndrome and Death in Patients With Coronavirus Disease 2019 Pneumonia in Wuhan. China [published online ahead of print. 2020 Mar 13]. JAMA Intern Med. 2020;e200994.

3. Zhou F. Yu T. Du R. Fan G. Liu Y. Liu Z. Xiang J. Wang Y. Song B. Gu X. Guan L. Wei Y. Li H. Wu X. Xu J. Tu S. Zhang Y. Chen H. Cao B. Clinical course and risk factors for mortality of adult inpatients with COVID19 in Wuhan. China: a retrospective cohort study. Lancet 2020;395(10229):10541062

4. Zhang Y. Xiao M. Zhang S. et al. Coagulopathy and Antiphospholipid Antibodies in Patients with Covid19 [published online ahead of print. 2020 Apr 8]. N Engl J Med. 2020;e38. doi:10.1056/NEJMc2007575

5. Tang N. Bai H. Chen X. Gong J. Li D. Sun Z. Anticoagulant treatment is associated with decreased mortality in severe coronavirus disease 2019 patients with coagulopathy [published online ahead of print. 2020 Mar 27]. J Thromb Haemost. 2020;10.1111/jth.14817.

6. Tang N. Li D. Wang X. Sun Z. Abnormal Coagulation parameters are associated with poor prognosis in patients with novel coronavirus pneumonia. J ThrombHaemost2020[Epub ahead of print]

7. Monagle K. Ryan A. Hepponstall M. et al. Inhalational use of antithrombotics in humans: Review of the literature. Thromb Res. 2015;136(6):1059–1066.

8. Page C. Heparin and related drugs: beyond anticoagulant activity. ISRN Pharmacol. 2013;2013:910743. Published 2013 Jul 30.

9. Scazziota A. Pons S. Heparin effects beyond antithrombotic activity. Hematología Volumen 21 Nº Extraordinario: 166175 XXIII Congreso Argentino de Hematología Noviembre 2017

10. Shastri MD. Peterson GM. Stewart N. Sohal SS. Patel RP. Nonanticoagulant derivatives of heparin for the management of asthma: distant dream or close reality? Expert Opin Investig Drugs. 2014;23(3):357–373.

11. Trybala E, Liljeqvist JA, Svennerholm B, Bergström T. Herpes Simplex Virus Types 1 and 2 Differ in Their Interaction with Heparan Sulfate.. J Virol. 2000 Oct;74(19):9106-14.

12. Courtney Mycroft-West, Dunhao Su, Stefano Elli , Scott Guimond,Gavin Miller, Jeremy Turnbull , Edwin Yates , Marco Guerrini , David Fernig , Marcelo Lima and Mark Skidmore. The 2019 coronavirus (SARS-CoV-2) surface protein (Spike) S1 Receptor Binding Domain undergoes conformational change upon heparin binding. bioRxiv preprint doi: https://doi.org/10.1101/2020.02.29.971093.This version posted March 2, 2020.

13. Scheuch G. Brand P. Meyer T. et al. Anticoagulative effects of the inhaled low molecular weight heparin certoparin in healthy subjects. J Physiol Pharmacol. 2007;58 Suppl 5(Pt 2):603–614.

14. YildizPekoz A. Ozsoy Y. Inhaled Heparin: Therapeutic Efficacy and Recent Formulations. J Aerosol Med Pulm Drug Deliv. 2017;30(3):143–156.

15. Bendstrup KE. Gram J. Jensen JI. Effect of inhaled heparin on lung function and coagulation in healthy volunteers. Eur Respir J. 2002; 19: 60610.

16. Harenberg J. Malsch R. Angelescu M. Lange C. Michaelis HC. Wolf H. Heene DL. Anticoagulant effects and tissue factor pathway inhibitor after intrapulmonary lowmolecularweight heparin. Blood Coagul Fibrinolysis. 1996; 7:47783.

17. Ghiasi F. Sadeghian M. Emami M. Kiaie BA. Mousavi S. A Pilot Study of Nebulized Heparin for Prevention of Ventilator Induced Lung Injury: Comparative Effects with an Inhaled Corticosteroid. Indian J Crit Care Med. 2017;21(10):634–639.

18. Abdelaal Ahmed Mahmoud A. Mahmoud HE. Mahran MA. Khaled M. Streptokinase Versus Unfractionated Heparin Nebulization in Patients With Severe Acute Respiratory Distress

Syndrome (ARDS): A Randomized Controlled Trial With Observational Controls. J Cardiothorac Vasc Anesth. 2020;34(2):436–443.

INHALE-HEP study

INHALEd nebulised unfractionated HEParin for the treatment of hospitalised patients with COVID-19 (INHALE-HEP)

Australian Protocol Number: GI-CC464253-437

Clinical Trial Protocol

Amendment 3, Version Number: 4.0

Date: 1 May 2022

Table of Contents

1. PROTOCOL SYNOPSIS 3
2. ADMINISTRATIVE INFORMATION 5
2.1 Trial /Study Registration 5
2.2 Funding 5
2.3 Roles and Responsibilities 5
3. INTRODUCTION 7
3.1 Background 7
3.2 Dose Justification for Inhaled Heparin 8
3.3 Safety and Tolerability of Nebulised Heparin 8
3.4 Risk of viral transmission to Health Care Practitioners (HCPs) 9
4. STUDY DESIGN 9
5. STUDY PARTICIPANTS 9
5.1 Eligibility Criteria 9
6. STUDY OUTCOMES 10
6.1 Primary outcome 10
6.2 Secondary outcomes 10
6.3 Tertiary outcomes 10
6.4 Safety assessment of inhaled Heparin sodium 11
7. STUDY INTERVENTIONS 11
7.1 Randomisation 11
7.2 Study Treatment 11
7.3 Dose modifications and stopping criteria 11
7.4 Concomitant Medications 12
7.5 Provisions for post-trial care 12
8. METHODS: DATA COLLECTION, MANAGEMENT AND ANALYSIS 12
8.1 Data collection methods 12
8.2 Data collection 12
8.3 Data Management 13
8.4 Statistical Methods 13
9. SAFETY MONITORING AND REPORTING 13
9.1 Investigator Brochure 13
9.2 Data and Safety Monitoring Committee (DSMC) 13
9.3 Safety Monitoring 14
9.4 Bleeding Events 14
9.5 Adverse events 14
10. .1 ETHICS AND DISSEMINATION 16
10.1 Research Ethics Approval 16
10.2 Consent 16
10.3 Confidentiality 16
11. QUALITY ASSURANCE 17
11.1 Investigator's File 17
11.2 Study materials 17
11.3 Initiation 17
11.4 Monitoring during the study 17
11.5 Close out 17
12. REFERENCES 18

1. Protocol Synopsis

Title	INHALEd nebulised unfractionated HEParin for the treatment of hospitalised patients with COVID-19
Short Title	INHALE-HEP
Study Design	A prospective multicentre randomised, open-label trial of nebulised heparin sodium in addition to standard care compared to standard care alone in hospitalised patients with COVID-19 infection.
Outcomes	*Primary Outcome:*<br>The primary outcome is intubation and requirement for invasive mechanical ventilation up to day 28 after randomisation<br>*Secondary Outcomes:*<br>• Survival to hospital discharge, censored at day 60<br>• Duration of hospital length of stay<br>*Tertiary Outcomes:*<br>• Duration of ICU stay<br>• Ventilator free days at day 60<br>• Daily change in modified ordinal score from baseline to day 28<br>*Safety assessments*<br>• Major bleeding<br>• Pulmonary bleeding<br>• Heparin induced thrombocytopenia (HIT)
Intervention	25,000 Units Heparin Sodium nebulised administration three times a day (8 hourly) or standard care until intubation or when the patient no longer requires supplemental oxygen for respiratory support
Number of participants	200 patients
Eligibility Criteria	*Inclusion Criteria*<br>1. Age 18 years or older<br>2. Currently admitted to hospital<br>3. Confirmed SARS-CoV-2 (COVID-19) infection by polymerase chain reaction (PCR) within the past 14 days. (Sample can be nasal or pharyngeal swab, sputum, tracheal aspirate, bronchoalveolar lavage)<br>4. Requiring oxygenation as defined by the modified ordinal clinical scale score 4-5.<br>*Exclusion Criteria*<br>1. Intubated and invasively mechanically ventilated or imminently requiring intubation as per the treating clinicians' assessment<br>2. Allergy to heparin or medical history of heparin-induced thrombocytopaenia<br>3. APTT >120 seconds, not due to anticoagulant therapy or corrected by with fresh frozen plasma administration. Measured within 48 hours prior to randomisation<br>4. Platelet count <$20x10^9$ per L within 48 hours of randomisation

	5. Pulmonary bleeding or uncontrolled bleeding within 48 hours of randomisation<br>6. Known or suspected pregnancy<br>7. Acute brain injury that may result in long-term disability<br>8. Myopathy, spinal cord injury, or nerve or disease with a likely prolonged incapacity to breathe independently e.g. Guillain-Barre syndrome<br>9. Treatment limitations in place, i.e. not for intubation, not for intensive care admission<br>10. Death is imminent or inevitable within 24 hours<br>11. Consent declined

2. Administrative Information

2.1 Trial /Study Registration

The Australian trial is registered on ClinicalTrials.gov with registration number NCT05184101.

The meta trial is registered on ClinicalTrials.gov with the registration number NCT04635241.

2.2 Funding

The study has received funding from the College of Health and Medicine, Australian National University (COVID-19 Crisis Research Seed Funding scheme, 2020).

2.3 Roles and Responsibilities

2.3.1 Chief Investigator

Name: Professor Frank M.P van Haren
Address: Intensive Care Unit
St George Hospital
Gray Street, Kogarah NSW 2217 Australia
Email: Frank.VanHaren@health.nsw.gov.au

3.3.2 Coordinating Centre

Name: The George Institute for Global Health
Address: Level 5, 1 King Street Newtown NSW 2042 Australia
Phone: +61 2 8052 4300
Email: INHALE-HEP@georgeinstitute.org.au

2.3.3 Management Committee:

- Professor Frank M. P. van Haren, (Chair), Professor, Australian National University, Canberra, Australia and Director of ICU, St George Hospital Sydney, Australia
- Associate/Professor Anthony Delaney, ICU Director of Research, Royal North Shore Hospital, Sydney, Australia
- Dr Rakshit Panwar, Senior Staff Specialist, John Hunter Hospital, Newcastle, Australia
- Dr Barry Dixon, Intensive Care Consultant, St Vincent's Hospital, Melbourne, Australia
- Professor John Myburgh, Director, Critical Care Program, The George Institute for Global Health, Sydney, Australia
- Associate Professor Naomi Hammond, Program Lead, Critical Care Program, The George Institute for Global Health, Sydney, Australia
- Dr Serena Knowles, Operations Lead, Academic Project Operations, Critical Care Program, The George Institute for Global Health, Sydney, Australia
- Ms Dorrilyn Rajbhandari, Senior Project Manager, Critical Care Program, The George Institute for Global Health, Sydney, Australia

2.3.4 INHALE HEP Meta-Trial International Executive Committee

- Professor Frank M.P. van Haren (Chair), Australian National University, Canberra, Australia
- Professor John Laffey (Co-Chair) National University of Ireland, Galway, Ireland
- Dr Barry Dixon, St Vincent's Hospital, Melbourne, Australia
- Professor Antonio Artigas, Autonomous University of Barcelona, Sabadell, Spain
- Professor Marcus Schultz Amsterdam University Medical Centre, Amsterdam, the Netherlands

The executive committee has overall leadership and responsibility of the INHALE-HEP Meta-Trial.

2.3.5 INHALE-HEP Meta-Trial Collaborative Research Group (CRG):

All contributing investigators and nominated research coordinators and nominated other collaborators are members of the INHALE-HEP Meta-Trial CRG. All publications will be made on behalf of the INHALE-HEP Meta-Trial Investigators.

3. Introduction

3.1 Background

In December 2019, a novel coronavirus (severe acute respiratory syndrome coronavirus 2, SARS-CoV-2) emerged in China and has since spread globally. As of August 20th, 2021, more than two-hundred and nine million cases of Coronavirus disease 2019 (COVID-19) were confirmed globally and close to 4.5 million deaths were reported.[1] Nearly 20% of patients experience hypoxemia, which is the primary reason for hospitalisation.[2] A significant proportion of patients admitted to hospital for COVID-19 develop acute respiratory distress syndrome (ARDS) according to the Berlin definition.[3-5] ARDS is an acute inflammatory lung injury, associated with increased pulmonary vascular permeability, increased lung weight, and loss of aerated lung tissue, affecting 23% of mechanically ventilated critically ill patients. The hospital mortality of ARDS is estimated between 35% and 46% depending on ARDS severity.[6 7] However, the death rate in COVID-19 patients requiring invasive mechanical ventilation appears to be higher, around 50-60%.[4 8]

The pathophysiology of COVID-19 associated lung injury is characterised by diffuse alveolar damage, hyperinflammation, coagulopathy, DNA neutrophil extracellular traps (NETS), hyaline membranes and microvascular thrombosis.[9] Our group and others have previously outlined the scientific rationale for the use of inhaled nebulised unfractionated heparin (UFH) as a treatment for COVID-19.[10-12] UFH has demonstrated antiviral activity in pre-clinical studies in concentrations relevant for administration to humans. SARS-CoV-2 Spike protein binding to human epithelial cells requires engagement of both cell surface heparan sulphate (HS) and angiotensin converting enzyme 2 (ACE-2), with HS acting as a co-receptor for ACE-2 interaction.[13] The SARS-CoV-2 Spike S1 protein receptor binding domain attaches to UFH and undergoes conformational change that blocks the binding and infectivity of SARS-CoV-2 to human bronchial epithelial cells.[14 15] This inhibition of SARS-CoV-2 infection of Vero E6 cells by an UFH preparation is concentration dependent, occurs at therapeutically relevant concentrations likely to be achieved following inhalation and exhibits a significantly stronger anti-viral effect compared to low molecular weight heparins (LMWHs).[16] The anti-inflammatory and anti-coagulant effects of inhaled UFH are thought to reduce pulmonary hyperinflammation and the generation of DNA NETs, limit fibrin deposition, hyaline membrane formation and microvascular thrombosis, which are all important features of COVID-19 associated lung injury.[10] Animal studies of nebulised UFH in different acute lung injury models have consistently shown a positive effect on pulmonary coagulation, inflammation and oxygenation.[10] Small human studies indicate that nebulised UFH limits pulmonary fibrin deposition, attenuates progression of acute lung injury and hastens recovery.[10] Early-phase trials in patients with acute lung injury and related conditions found that nebulised UFH reduced pulmonary dead space, coagulation activation, microvascular thrombosis, improved lung injury and increased time free of ventilatory support.[17-20] In the CHARLI study, a pre-pandemic multicentre double-blind randomised controlled phase 3 study in 256 critically ill ventilated patients conducted by our group, nebulised UFH limited progression of lung injury including ARDS and accelerated return to home in survivors.[21]

Overall, these data support the conclusion that the multiple pharmacological effects of inhaled UFH may have significant therapeutic benefit in patients with COVID-19.

A recently published case series of 98 patients with COVID-19 shows that the intervention is safe and is associated with an improvement in oxygenation.[22]

We have published the protocol and statistical analysis plan for an international meta-trial of randomised studies investigating inhaled heparin in hospitalised patients with COVID-19 “INHALE-HEP”, of which the current randomised study will form part.[23]

The purpose of this study is to investigate the therapeutic effects and safety of inhaled UFH in hospitalised non-invasively ventilated patients with COVID-19. The benefit/risk to the patient is

acceptable as data show that no acute or chronic toxicological effects have been associated with the inhaled route for any dose of UFH.

3.2 Dose Justification for Inhaled Heparin

Heparin sodium 25,000 IU/8hours (equivalent to 25 mg delivered to the lungs, assuming a specific activity of 200 IU/mg and that nebulised administration delivers 20% of the total dose to the lung [data on file]) until intubation or when the patient no longer requires supplemental oxygen for respiratory support.

3.3 Safety and Tolerability of Nebulised Heparin

Inhaled UFH is not approved for any indication. Intravenous (IV) heparin is approved and is generally well-tolerated. Adverse reactions reported per the approved labelling for IV heparin include, haemorrhage, heparin-induced thrombocytopenia (HIT) and heparin-induced thrombocytopenia and thrombosis (HITT), thrombocytopenia, heparin resistance, and hypersensitivity; however, these events have not been reported with inhaled UFH due to reduced systemic absorption.

APTT. Treatment with nebulised heparin at the dose proposed for this study, while concomitantly receiving IV or SC unfractionated heparin, is likely to cause a mild (approximately 7 seconds) increase in the average APTT and a mild-moderate (approximately 18 seconds) increase in the peak APTT during the treatment period. The APTT of patients who are not receiving IV or SC unfractionated heparin is likely to be not affected by treatment with nebulised heparin[21].

Bleeding and blood transfusion. Nebulised heparin at the dose proposed in this study has not been found to increase the risk of major non-pulmonary bleeding or of blood transfusion[21]. In clinical trials where more than 150 intensive care patients were treated with nebulised heparin for ARDS and related conditions, there were no cases of pulmonary haemorrhage with patient deterioration, but one patient had a large volume haemoptysis that led the site investigators to withhold all forms of anticoagulation. In these trials, approximately 1 out of 20 patients treated with nebulised heparin experienced greater-than-usual bloodstaining of secretions and, although this bloodstaining can be unsightly, rarely is it clinically deleterious. The risk of serious haemoptysis is estimated to be less than 1 out of 100.

Use in asthma and chronic obstructive pulmonary disease (*COPD).* Use in asthma and COPD is typically well-tolerated but, on rare occasions in patients with severe bronchospasm, airflow may be further impeded immediately following the start of nebulisation. This may represent heparin accumulating on the luminal surface of terminal bronchioles, causing further narrowing. This typically resolves within minutes of stopping nebulisation and withholding the remainder of the dose. Subsequent doses are typically well-tolerated, but vigilance is required.

Heparin-induced thrombocytopenia (HIT). HIT is an immune-mediated adverse reaction caused by the emergence of antibodies that activate platelets in the presence of heparin. Hospitalised patients are commonly treated with subcutaneous heparin for the prevention of deep vein thrombosis. The risk of HIT from treatment with subcutaneous unfractionated heparin is less than 1 out of 100. Nebulised heparin is not thought to increase the risk of HIT in patients already receiving unfractionated heparin and administration by nebulisation is not thought to increase the risk of HIT compared to administration by other routes and no patient to date in our earlier trials developed HIT.

Mucolytic effect. Patients receiving inhaled UFH can expect to expectorate larger volumes of sputum, reflecting the pharmacological effect of a mucolytic, and although this is not an AE, patients may score this effect negatively on visual analogue scales reflecting body image or social marginalisation. Therefore, patients should be advised of this likely effect and that it is considered a beneficial effect.

3.4 Risk of viral transmission to Health Care Practitioners (HCPs)

There is currently no evidence that the administration of nebulised medication to patients with COVID-19 increases the risk of transmission of the virus.[24] In a study during the 2002-2003 SARS-CoV outbreaks, there was no evidence of SARS-CoV-specific nucleic acid material in air samples taken from the vicinity of a patient with SARS undergoing treatment with a large-volume nebuliser.[25] In a 2012 systematic review, no statistically significant risk of SARS-CoV transmission to health care personnel caring for patients undergoing nebuliser treatment was found.[26] According to the CDC, there are no currently known links between performing nebulised treatments and an increased risk of SARS-CoV-2 infection (https://www.cdc.gov/coronavirus/2019-ncov/hcp/faq.html#Infection-Control, accessed 6/10/2020).

Based on limited available data, it is uncertain whether aerosols generated from some procedures, such as nebuliser administration and high flow oxygen delivery, may be infectious. Aerosols generated by nebulisers are derived from medication in the nebuliser. It is uncertain whether potential associations between performing this common procedure and increased risk of infection might be due to aerosols generated by the procedure or due to increased contact between those administering the nebulised medication and infected patients. The National Institute for Health and Care Excellence and the UK government guidance from the New and Emerging Respiratory Virus Threats Advisory Group advise the continued use of nebulisers, administration of medication via nebulisation, is not considered to represent a significant infectious risk. This is because the aerosol produced during nebuliser treatment is not patient-derived but is generated from fluid from the nebuliser chamber, and therefore, does not carry patient-derived viral particles. If a particle in the aerosol comes into contact with a patient's contaminated mucous membrane, it will cease to be airborne and therefore, will not be part of an aerosol.

Administration of nebulised heparin may provide some protection for health care providers through inactivation of the virus in the patient's respiratory tree, thus reducing the expired active viral load and the risk of transmission to HCPs.

For the current study, to minimise any risk of viral transmission, we require nurses (or respiratory therapists in some health care settings) wear full PPE including an N95 mask, goggles/face shield, gloves and gown when administering nebulised treatment to patients with COVID-19.

4. Study Design

This is an investigator-initiated prospective multi-centre open labelled randomised controlled trial of nebulised heparin sodium in addition to standard care compared to standard care alone in hospitalised patients with COVID-19 infection.

5. Study Participants

The study will be conducted in participating sites in Australia.

5.1 Eligibility Criteria

5.1.1 Inclusion criteria

1. Age 18 years or older
2. Currently admitted to hospital
3. Confirmed SARS-CoV-2 (COVID-19) infection by polymerase chain reaction (PCR) within the past 14 days. (Sample can be nasal or pharyngeal swab, sputum, tracheal aspirate, bronchoalveolar lavage)
4. Requiring oxygenation as defined by the modified ordinal clinical scale score 4-5.

5.1.2 Exclusion Criteria

1. Intubated and invasively mechanically ventilated or imminently requiring intubation as per the treating clinicians' assessment
2. Allergy to heparin or medical history of heparin-induced thrombocytopaenia
3. APTT >120 seconds, not due to anticoagulant therapy or corrected by with fresh frozen plasma administration. Measured within 48 hours prior to randomisation
4. Platelet count <$20x10^9$ per L within 48 hours prior to randomisation
5. Pulmonary bleeding or uncontrolled bleeding within 48 hours of randomisation
6. Known or suspected pregnancy
7. Acute brain injury that may result in long-term disability
8. Myopathy, spinal cord injury, or nerve or disease with a likely prolonged incapacity to breathe independently e.g. Guillain-Barre syndrome
9. Treatment limitations in place, i.e. not for intubation, not for intensive care admission
10. Death is imminent or inevitable within 24 hours
11. Consent not obtained

6. Study Outcomes

6.1 Primary outcome

The primary outcome is the requirement for invasive mechanical ventilation up to day 28 after randomisation.

6.2 Secondary outcomes

- Survival to hospital discharge, censored at day 60
- Duration of hospital length of stay

6.3 Tertiary outcomes

- Duration of ICU stay
- Ventilator free days at day 60
- Daily change in modified ordinal score from baseline to day 28 (Table 2)

Table 2. Modified Ordinal Clinical Scale for COVID-19[27 28]

Modified Ordinal Scale	
1	Not hospitalised
2	Hospitalised, not requiring supplemental oxygen and no longer requiring medical care for COVID-19
3	Hospitalised not requiring supplemental oxygen but needing medical care for COVID-19
4	Hospitalised requiring supplemental oxygen
5	Hospitalised requiring non-invasive ventilation or high flow oxygen
6	Hospitalised requiring intubation and mechanical ventilation or ECMO
7	Death

6.4 Safety assessment of inhaled Heparin sodium

- Major bleeding, defined as: bleeding that results in death and/or bleeding that is symptomatic and or results in a fall in haemoglobin of 20g/L or more, or results in transfusion of two or more units of whole blood or red cells
- Pulmonary bleeding, is defined as frank bleeding in the lungs, trachea or bronchi with repeated haemoptysis and associated with acute deterioration in respiratory status
- HIT, is defined as an unexplained fall in platelet count and a positive heparin antibody test

7. Study Interventions

7.1 Randomisation

Randomisation will be performed via a central secure study database accessed via the internet at each site. A block randomisation method stratified by site with variable block sizes will be performed. Patients will be randomised to nebulised heparin sodium in addition to the standard care or standard care only. There is a one-to-one allocation ratio.

7.2 Study Treatment

Heparin sodium 25,000 IU/5 mL manufactured by Pfizer Australia Pty Ltd (Sydney, Australia) or an equivalent unfractionated heparin by another approved manufacturer will be used. The medication will be from site pharmacy stock. It will be stored and dispensed as per local policies.

Heparin sodium will be administered as a nebulised aerosol dose of 25,000 IU heparin three times a day (8 hourly) via a vibrating mesh aerosol drug nebulizer (preferred brand, Aerogen Solo (Aerogen, Ireland)). It has been estimated that 20% of the heparin dose is administered to the lungs by this method using Anderson cascade impactor (data on file). Other nebulisers are acceptable on approval from the coordinating centre.

Nebulised heparin sodium 25,000 IU can be delivered using air or oxygen three times a day (8 hourly), for up to 21 days whilst in hospital, until the patient is intubated and invasively mechanically ventilated or no longer requires supplemental oxygen for respiratory support.

The mode of delivery (via mask or mouthpiece) will be chosen by the clinical team depending on the patient's needs for oxygen and non-invasive ventilatory support.

7.3 Dose modifications and stopping criteria

There is no need for dose modification in either renal or hepatic impairment because no acute or chronic toxicological effects have been associated with the inhaled route for any dose of heparin investigated in man. Studies in healthy adults and our previous studies[21] have shown no significant systemic absorption of inhaled UFH (evaluated by blood coagulation time) at the dosage proposed.

7.3.1 Discontinuation of Study Drug

Discontinuation of the study drug should be considered by the Investigator when a patient meets one of the following conditions:

- Bronchospasm which cannot be ameliorated by pre-treatment with bronchodilators
- Sudden hypoxia; oxygen saturation should be monitored throughout nebulisation of UFH
- The treating physician deems that there is a clinically unacceptable increase in APTT
- The treating physician deems that there is excessive bloodstaining of respiratory secretions or significant haemoptysis
- There is pulmonary bleeding, major bleeding or suspected or confirmed HIT

Nebulised heparin should be recommenced if the patient continues to require oxygen support, and:

- Having been withheld because the APTT was unacceptably prolonged, the APTT becomes acceptable
- Having been withheld because there was excessive bloodstaining of respiratory secretions, the bloodstaining of the respiratory secretions has resolved
- Having been withheld for pulmonary bleeding or major bleeding, the bleeding is definitively controlled
- Having been withheld for suspected HIT, the patient is found not to have this condition

Although allergic reaction has not been reported in any study, anaphylaxis is a theoretical possibility. Should it occur, it should be treated per standard of care.

7.4 Concomitant Medications

Treatment with any or all of the following therapies is permitted during the study and not a reason to withhold study medication: deep vein thrombosis prophylaxis with UFH or LMWH; 'full' therapeutic dose UFH or LMWH for a recognised clinical indication; non-heparin anticoagulants; anti-thrombotic medications; protamine; prone positioning; and inhaled nitric oxide. There are no prohibitions during the trial.

7.5 Provisions for post-trial care

Post-trial care will be standard care through the standard healthcare system from each institution and jurisdiction in each individual study.

8. Methods: Data collection, management and analysis

8.1 Data collection methods

Research coordinators and investigators at each site will work with clinicians to identify potential candidates for enrolment. Patients admitted to the hospital with confirmed COVID-19 pneumonia will be screened for eligibility into the study. A screening log will be maintained at each participating site. With large numbers of patients in hospital, it may not be possible to have 'screened' all COVID patients, if this is the case then admission numbers will be reported to estimate the potential eligible population for the study. Potential patients who meet all inclusion criteria and no exclusion criteria will be approached to explain the study and request consent.

Once consent is obtained, patients will be randomised, study treatment will commence and participants followed up until hospital discharge or 60 days post randomisation or death, whichever comes first.

8.2 Data collection

At baseline, the following information will be collected to determine standard measures of illness severity to allow baseline balance between groups to be assessed:

- Demographics
- Hospital admission details
- Clinical information: vital signs, medical history,
- Concomitant medications
- Oxygen therapy requirements

Daily clinical information will be collected whilst the patient is in hospital for up to 28 days post randomisation and will allow assessment of response to treatment and to monitor safety:

- Vital signs and oxygen requirements
- laboratory data
- Study treatment administered
- Concomitant therapy and medication

Outcome data is collected till the patient no longer requires supplemental oxygen for respiratory support or is intubated and mechanically ventilated, or is discharged from hospital, censored at day 60.

- Date the patient no longer requires supplemental oxygen for respiratory support
- Invasive mechanical ventilation start and stop date
- ICU admission and discharge date
- Hospital discharge date

8.3 Data Management

Data will be collected using a secure online electronic case report form. All paper study records, including consent documentation, paper CRFs (if used) will be stored in locked cabinets accessible only to authorised personnel. Electronic information will be kept on password protected computers accessible only to authorised personnel.

All study material will be stored for a minimum period of 15 years after the conclusion of the study.

8.4 Statistical Methods

8.3.1 Principles

Principal analyses will be performed on an intention-to-treat basis.

Where data are missing, we report the number of available observations and make no assumptions about missing data.

The primary outcome is intubation and requirement for invasive mechanical ventilation up to day 28 after randomisation (or death, for patients who died before intubation). This will be assessed in a time to event analysis and a regression analysis of the proportion of patients receiving intubation by day 28 after randomisation. The primary outcome will be presented using Kaplan-Meier curves, analysed using the log-rank test.

Data will be presented as means ± standard deviations (SD) or medians with interquartile ranges (IQR). Proportions will be compared using a chi-squared test or Fisher’s exact test, and continuous variables will be compared using Student t-test or Wilcoxon rank sum test when two groups are compared and corresponding paired tests for matched patients, as appropriate. Comparisons of event rates in the two groups will be presented as hazard ratios (HR) with 95% confidence intervals (95% CI).

8.3.2 Sample size

This study will feed into the meta-trial. The goal is to recruit 200 patients across Australian sites. For the meta-trial, to demonstrate a clinically important reduction in the primary outcome, a sample size of 712 is required, assuming a decrease in the proportion of patients receiving invasive mechanical ventilation from 12 to 6%, with power 80% and a 2-sided significance level of 0.05.

9. Safety Monitoring and Reporting

9.1 Investigator Brochure

The Investigator Brochure will be supplied to participating sites, updated and approved as required.

9.2 Data and Safety Monitoring Committee (DSMC)

A DSMC, independent from the coordinating centre and investigators, will perform an ongoing review of predefined safety parameters, study outcomes and overall study conduct. The DSMC will be comprised of experts in clinical trials, biostatistics and intensive care medicine.

The DSMC will review all unblinded adverse reactions, SAEs and SUSARs at predetermined intervals during the study or as deemed appropriate by the DSMC.

9.3 Safety Monitoring

Bleeding events, Adverse Events (AE), Serious Adverse Events (SAE) and (Suspected Unexpected Serious Adverse Reactions (SUSARs) will be communicated by site investigators to the Coordinating Centre. In general, this will occur in writing within 7 days for Bleeding and AEs and 24 hours for SAE or SUSARs, of the site investigator becoming aware of the event. The Coordinating Centre will provide the human research ethics committee (HREC), the DSMC and regulatory authorities with safety reports and updates.

Inhaled UFH is not approved for any indication. IV heparin is approved and is generally well-tolerated. Adverse reactions reported per the approved labelling for IV heparin include: haemorrhage, heparin-induced thrombocytopenia (HIT) and heparin-induced thrombocytopenia and thrombosis (HITT), thrombocytopenia, heparin resistance, and hypersensitivity; however, these events have not been reported with inhaled UFH due to reduced systemic absorption. Patients receiving inhaled UFH can expect to expectorate larger volumes of sputum, reflecting the pharmacological effect of a mucolytic.

Additionally, possible adverse effects associated with inhaled UFH include: headache, epistaxis, systemic anticoagulation (this is not expected at the inhaled dose used in this study) and haemoptysis.

9.4 Bleeding Events

The following adverse events and reactions must be reported:

Major bleeding, which is: bleeding that results in death and/or; bleeding that is symptomatic and/or; bleeding that results in a fall in haemoglobin of 20g/L or more, or bleeding that results in transfusion of two or more units of whole blood or red cells

Pulmonary bleeding, which is frank bleeding in the lungs, trachea or bronchi with repeated haemoptysis <u>and</u> associated with acute deterioration in respiratory status

HIT, which is an unexplained fall in platelet count <u>and</u> a positive heparin antibody test.
Simple bloodstaining of the respiratory secretions is common in the study population and should not be reported as an adverse event.

Changes in APTT are common in the study population and should not be reported as a bleeding event.

All symptomatic or major bleeding, pulmonary bleeding and HIT events that occur in both study groups up to day 28 will be reported, irrespective of their perceived relationship to the study. All event reports will be assessed by the DSMC at specified time points throughout the study.

9.5 Adverse events

It is recognised that the patient population will experience a number of aberrations in laboratory values, signs and symptoms due to the severity of the underlying disease and the impact of standard critical medicine therapies. These will not necessarily constitute an adverse event or serious adverse event unless they are considered to be related to study treatment or in the Principal Investigators clinical judgement are not recognised events consistent with the patient's underlying disease and expected clinical course.

In this study, reporting of adverse events will be restricted to events that are considered to be related to study treatment (possibly, probably or definitely).

9.5.1 Adverse Drug Reactions (ADR)

Regarding marketed medicinal products, a well-accepted definition of an adverse drug reaction is[29]:

> *A response to a drug which is noxious and unintended, and which occurs in doses normally used in humans for prophylaxis, diagnosis or therapy of disease or for modification of physiological function*.

Any adverse reaction thought to be study treatment related will be reported to the coordinating centre within 7 days of discovery. The Principal Investigator will be responsible for determining the causal relationship as either possible, probable or definitely study treatment related. Notification will be by scanned document sent by email or by notification of a completed ADR form on the web-based data management system.

All adverse reactions will be reviewed by the coordinating centre staff and recorded in a safety database which will be monitored by the study executive management committee on a quarterly basis.

9.5.2 Serious Adverse Events (SAEs)

Serious adverse events are defined as any untoward medical occurrence that meets one of more of the following criteria:

- Results in death
- Is life-threatening
- Requires inpatient hospitalisation or prolongation of existing hospitalisation
- Results in persistent or significant disability/incapacity
- Is a congenital anomaly/birth defect

The classification of 'serious adverse event' is not related to the assessment of the severity of the adverse event. An event that is mild in severity may be classified as a serious adverse event based on the above criteria.

Serious adverse drug reactions should be reported to the coordinating centre within 24 hours of participating site study staff becoming aware of the occurrence. A member of the coordinating centre will be on call 24 hours a day via mobile phone for out of 'business hours' reporting.

9.5.3 Suspected Unexpected Serious Adverse Reactions (SUSARs)

A SUSAR whose nature, severity, specificity, or outcome is not consistent with the term or description used in the product information, should be considered unexpected. These will also be reported to the coordinating centre within 24 hours of participating site study staff becoming aware of the occurrence.

9.5.4 Reporting SAEs and SUSARs

The minimum information to report will include:

- Study number
- Nature of the event
- Commencement and cessation of the event
- All concomitant medications or procedures being used at the time of the event and in response at the event
- The principal or co-investigator's opinion of the relationship between study drug and the event (not, possibly, probably or definitely related)
- Whether treatment was required for the event and what treatment was administered
- The outcome of the event.

It is the responsibility of each Principal Investigator to inform the Coordinating Centre of all SADR and SUSAR events which occur at their hospital. The Coordinating Centre will report all SADRs and SUSARs to the lead HREC and is also responsible for alerting the Chief Investigator and reports for the Data Safety Management Committee. Reports of SAEs and SUSARs will be provided to the regulatory authorities and other participating sites within the required time frames.

Copies of any reporting and correspondence to and from the local RGO should also be sent to the coordinating centre.

10.1 Ethics and Dissemination

10.1 Research Ethics Approval

The study will be conducted in accordance with the ethical principles consistent with the Declaration of Helsinki[30] and all relevant national and local guidelines on the ethical conduct of research[29 31 32].

The protocol for this project will be reviewed by a lead Human Research Ethics Committee (HREC). One Principal Investigator (known as the coordinating investigator) will take responsibility for applying to a lead HREC on behalf of investigators covered by that committee. Each site Principal Investigator will then be responsible for applying for local research governance approval at their site.

The coordinating centre will assist with this process by preparing a Human Research Ethics Application (HREA) for a lead multicentre trial and template consent documents. The content and format of the standard information statements and consent forms will be adapted if necessary, to comply with local HREC guidelines and requirements. Documentation of the approval of the protocol and the consent documents will be provided to the coordinating centre before the study may begin at any site.

During the trial, any amendment or modification to the study protocol or consent documents will be notified to the HREC by the Principal Investigator and only implemented on receipt of HREC approval, unless the change is necessary to eliminate an immediate hazard to patients, in which case the HREC will be informed as soon as possible thereafter.

Each Principal Investigator will be responsible for informing the HREC of anything likely to affect the safety of patients or the continued conduct of the clinical trial. The coordinating centre will produce and submit progress reports, adverse event reports, and any other required documentation to the HREC in accordance with their guidelines. Copies of all HREC and research governance officer correspondence will be provided to the coordinating centre together with a copy of the approved consent documents. Copies of the same documents will also be kept with the study investigator files.

10.2 Consent

Prior consent will be sought from the patient before project related activities are undertaken, the study will be explained verbally to that patient by the principal investigator or their nominated delegate. The patient will be given the opportunity to read the participant information sheet and ask any questions prior to deciding on participation in the study. If the patient consents to participate, they will be given a copy of the signed and dated consent form and the participant information sheet and any other documentation discussed through the consent process.

10.3 Confidentiality

All patient data pertaining to the study will be stored in a computer database maintaining confidentiality in accordance with local legislation on privacy and use of health data. When archiving or processing data pertaining to the investigator and/or to the patients, the coordinating centre will take all appropriate measures to safeguard and prevent access to this data by any unauthorised third party.

The site Principal Investigator will maintain the confidentiality of all study documentation and take measures to prevent accidental or premature destruction of these documents. The site Principal Investigator will retain the study documents at least fifteen years after the completion or discontinuation of the study. The site Principal Investigator must notify the study management committee prior to destroying any study essential documents following the study completion or discontinuation. If the site Principal Investigator's personal situation is such that archiving can no longer be ensured by him/her, the site Principal Investigator shall inform the study management committee and the relevant records shall be transferred to a mutually agreed upon designee.

If any site Principal Investigator retires, relocates, or otherwise withdraws from conducting a study, the responsibility for maintaining records may be transferred to the coordinating centre, or other

investigator. The coordinating centre must be notified of and agree to the change. All associated documentation must also be updated.

11.Quality Assurance

The study will be conducted in accordance with the ICH Guidelines for Good Clinical Practice[29] and with all relevant local and national regulations[31 32].

11.1 Investigator's File

A file containing essential documents as outlined in the ICH guidelines for Good Clinical Practice will be maintained at each participating site. A copy of the same documents will be held at the coordinating centre.

11.2 Study materials

A comprehensive guide to the data collection with definitions and rationale will be provided together with a paper version of the data collection forms. A guide to using the web-based data management system is also provided in the guideline for CRF completion. All of these documents will be provided in PDF format for printing by the study coordinating centre. These aim to assist the research coordinator to ensure high-quality data collection and data entry.

11.3 Initiation

All site personnel will receive training, explaining the protocol and procedures, use of the web-based randomisation system and electronic case report forms (eCRF). The Site 'initiation visit' will be conducted by teleconference or video conference or face to face meeting at the participating site. Written and electronic materials will be supplied for study staff and for the education of clinical ICU staff at each participating site.

11.4 Monitoring during the study

The coordinating centre monitor will visit each study centres on several occasions during the recruitment phase. This will ensure that the study is conducted according to the protocol, good clinical practice guidelines and relevant regional regulatory requirements. The study monitor will review study records for source document verification, confirm valid patient consents, data quality, study drug compliance and the completeness of follow-up. In addition, the study may also be audited by local or national regulatory authorities. Access to source documents and other study files will be made available at all study centres for monitoring and audit purposes. In circumstances of reduced travel due to public health directives, visits will be limited to remote or virtual visits.

The coordinating centre team will conduct regular remote monitoring on the web-based database by applying validation and consistency rules and with regular data cleaning to ensure the integrity of the study data.

11.5 Close out

At completion of the study, the monitor will ensure that there are plans in place for the long-term storage of all the relevant data and source documentation (for 15 years).

12.REFERENCES

1. WHO. WHO Coronavirus Disease (COVID-19) Dashboard 2021 [Available from: https://covid19.who.int accessed 20 August 2021.

2. Wu Z, McGoogan JM. Characteristics of and Important Lessons From the Coronavirus Disease 2019 (COVID-19) Outbreak in China: Summary of a Report of 72314 Cases From the Chinese Center for Disease Control and Prevention. *JAMA* 2020 doi: 10.1001/jama.2020.2648 [published Online First: 2020/02/25]

3. Grasselli G, Zangrillo A, Zanella A, et al. Baseline Characteristics and Outcomes of 1591 Patients Infected With SARS-CoV-2 Admitted to ICUs of the Lombardy Region, Italy. *JAMA* 2020 doi: 10.1001/jama.2020.5394 [published Online First: 2020/04/07]

4. Wu C, Chen X, Cai Y, et al. Risk Factors Associated With Acute Respiratory Distress Syndrome and Death in Patients With Coronavirus Disease 2019 Pneumonia in Wuhan, China. *JAMA Intern Med* 2020 doi: 10.1001/jamainternmed.2020.0994 [published Online First: 2020/03/14]

5. Ranieri VM, Rubenfeld GD, Thompson BT, et al. Acute respiratory distress syndrome: the Berlin Definition. *JAMA* 2012;307(23):2526-33. doi: 10.1001/jama.2012.5669 [published Online First: 2012/07/17]

6. Bellani G, Laffey JG, Pham T, et al. Epidemiology, Patterns of Care, and Mortality for Patients With Acute Respiratory Distress Syndrome in Intensive Care Units in 50 Countries. *JAMA* 2016;315(8):788-800. doi: 10.1001/jama.2016.0291 [published Online First: 2016/02/24]

7. Thompson BT, Chambers RC, Liu KD. Acute Respiratory Distress Syndrome. *N Engl J Med* 2017;377(6):562-72. doi: 10.1056/NEJMra1608077 [published Online First: 2017/08/10]

8. Tzotzos SJ, Fischer B, Fischer H, et al. Incidence of ARDS and outcomes in hospitalized patients with COVID-19: a global literature survey. *Crit Care* 2020;24(1):516. doi: 10.1186/s13054-020-03240-7 [published Online First: 2020/08/23]

9. Ackermann M, Verleden SE, Kuehnel M, et al. Pulmonary Vascular Endothelialitis, Thrombosis, and Angiogenesis in Covid-19. *New England Journal of Medicine* 2020;383(2):120-28. doi: 10.1056/NEJMoa2015432

10. van Haren FMP, Page C, Laffey JG, et al. Nebulised heparin as a treatment for COVID-19: scientific rationale and a call for randomised evidence. *Crit Care* 2020;24(1):454. doi: 10.1186/s13054-020-03148-2 [published Online First: 2020/07/24]

11. Conzelmann C, Müller JA, Perkhofer L, et al. Inhaled and systemic heparin as a repurposed direct antiviral drug for prevention and treatment of COVID-19. *Clinical Medicine* 2020;20(6):e218-e21. doi: 10.7861/clinmed.2020-0351

12. Ball L, Schultz MJ, Pelosi P. Nebulised heparin for patients on ventilation: implications for COVID-19 pneumonia. *The Lancet Respiratory Medicine* 2021 doi: https://doi.org/10.1016/S2213-2600(20)30513-0

13. Clausen TM, Sandoval DR, Spliid CB, et al. SARS-CoV-2 Infection Depends on Cellular Heparan Sulfate and ACE2. *Cell* 2020;183(4):1043-57 e15. doi: 10.1016/j.cell.2020.09.033 [published Online First: 2020/09/25]

14. Kwon PS, Oh H, Kwon SJ, et al. Sulfated polysaccharides effectively inhibit SARS-CoV-2 in vitro. *Cell Discov* 2020;6:50. doi: 10.1038/s41421-020-00192-8 [published Online First: 2020/07/28]

15. Mycroft-West CJ, Su D, Pagani I, et al. Heparin Inhibits Cellular Invasion by SARS-CoV-2: Structural Dependence of the Interaction of the Spike S1 Receptor-Binding Domain with Heparin. *Thromb Haemost* 2020;120(12):1700-15. doi: 10.1055/s-0040-1721319 [published Online First: 2020/12/29]

16. Tree JA, Turnbull JE, Buttigieg KR, et al. Unfractionated heparin inhibits live wild type SARS-CoV-2 cell infectivity at therapeutically relevant concentrations. *British Journal of Pharmacology* 2021;178(3):626-35. doi: https://doi.org/10.1111/bph.15304

17. Dixon B, Schultz MJ, Hofstra JJ, et al. Nebulized heparin reduces levels of pulmonary coagulation activation in acute lung injury. *Crit Care* 2010;14(5):445. doi: 10.1186/cc9269 [published Online First: 2010/11/12]

18. Dixon B, Campbell DJ, Santamaria JD. Elevated pulmonary dead space and coagulation abnormalities suggest lung microvascular thrombosis in patients undergoing cardiac surgery. *Intensive Care Med* 2008;34(7):1216-23. doi: 10.1007/s00134-008-1042-7 [published Online First: 2008/02/28]

19. Dixon B, Schultz MJ, Smith R, et al. Nebulized heparin is associated with fewer days of mechanical ventilation in critically ill patients: a randomized controlled trial. *Crit care* 2010;14(5):R180. doi: 10.1186/cc9286 [published Online First: 2010/10/13]

20. Dixon B, Smith R, Santamaria JD, et al. A trial of nebulised heparin to limit lung injury following cardiac surgery. *Anaesth Intensive Care* 2016;44(1):28-33.

21. Dixon B, Smith RJ, Campbell DJ, et al. Nebulised heparin for patients with or at risk of acute respiratory distress syndrome: a multicentre, randomised, double-blind, placebo-controlled phase 3 trial. *The Lancet Respiratory Medicine* 2021 doi: https://doi.org/10.1016/S2213-2600(20)30470-7

22. van Haren FMP, van Loon LM, Steins A, et al. Inhaled nebulised unfractionated heparin for the treatment of hospitalised patients with COVID-19: A multicentre case series of 98 patients. *Br J Clin Pharmacol* 2022 doi: 10.1111/bcp.15212 [published Online First: 2022/01/06]

23. van Haren FMP, Richardson A, Yoon H-J, et al. INHALEd nebulised unfractionated HEParin for the treatment of hospitalised patients with COVID-19 (INHALE-HEP): Protocol and statistical analysis plan for an investigator-initiated international metatrial of randomised studies. *British Journal of Clinical Pharmacology* 2021:1-17. doi: https://doi.org/10.1111/bcp.14714

24. Tashkin DP, Barjaktarevic IZ. Nebulized Treatments and the Possible Risk of Coronavirus Transmission: Where Is the Evidence? *Chronic Obstr Pulm Dis* 2020;7(3):136-38. doi: 10.15326/jcopdf.7.3.2020.0161 [published Online First: 2020/05/16]

25. Wan GH, Tsai YH, Wu YK, et al. A large-volume nebulizer would not be an infectious source for severe acute respiratory syndrome. *Infect Control Hosp Epidemiol* 2004;25(12):1113-5. doi: 10.1086/502353 [published Online First: 2005/01/08]

26. Tran K, Cimon K, Severn M, et al. Aerosol generating procedures and risk of transmission of acute respiratory infections to healthcare workers: a systematic review. *PLoS One*

2012;7(4):e35797. doi: 10.1371/journal.pone.0035797 [published Online First: 2012/05/09]

27. Marshall J, Murthy S, Diaz J, et al. WHO Working Group on the Clinical Characterisation and Management of COVID-19 infection: A minimal common outcome measure set for COVID-19 clinical research. *Lancet Infect Dis* 2020;20(8):e192-e97.

29. International Conference on Harmonisation (ICH). Clinical safety data management: Definitions and standards for expedited reporting. 1994

30. Association WM. World Medical Association Declaration of Helsinki: ethical principles for medical research involving human subjects. *Jama* 2013;310(20):2191-94.

31. National Health Medical Research Council ARCAV-CC. National Statement on Ethical Conduct in Human Research 2007 (Updated May 2015): National Health and Medical Research Council, Australian Research Council …, 2007.

32. Australian, Group NZICSCT, Rischbieth A, et al. Ethical intensive care research: development of an ethics handbook. *Critical Care and Resuscitation* 2005;7(4)

**Protocolo número**	**GDN 019/20**
***Status*:**	***final***
Versão:	***v2***
**Data:**	**29/outubro/2020**

PROTOCOLO DE ESTUDO CLÍNICO

Ensaio clínico fase 2 de heparina não fracionada inalada para o tratamento de pacientes hospitalizados com COVID-19.

Confidencial

CENTRO DE PESQUISA CLÍNICA

Galeno Desenvolvimento de Pesquisas Clínicas Ltda
PESQUISADOR PRINCIPAL: Gilberto De Nucci
CNPJ: 05.531.007/0001-53
Rua Latino Coelho, 1.301
Campinas/SP
CEP: 13087-010
Tel: 55 19 3242-7133
E-mail: denucci@gilbertodenucci.com

DECLARAÇÃO DE CONFIDENCIALIDADE E CONFORMIDADE

Este protocolo de ensaio clínico foi desenhado de acordo com os padrões estabelecidos pelo guia de boas práticas clínicas do ICH (*International Council for Harmonisation of Technical Requirements for Pharmaceuticals for Human Use) "Integrated Addendum to ICH E6(R1): Guideline for Good Clinical Practice E6(R2) - Current Step 4 version, dated 9 November 2016*". Este protocolo é confidencial por natureza e só pode ser acessado por pessoas autorizadas.

PÁGINA DE ASSINATURA DO PROTOCOLO

Li este projeto, seu protocolo de ensaio clínico e o termo de consentimento livre e esclarecido e concordo em conduzir este estudo em conformidade com o estipulado pelo protocolo, pelas Resoluções nº 466/12 e 251/97 do Conselho Nacional de Saúde - Ministério da Saúde, bem como a RDC 56 de 08 de maio de 2014 da ANVISA e de acordo com a Declaração de Helsinque (1964) e as revisões de Tóquio (1975), Veneza (1983), Hong Kong (1989), Somerset Oeste (1996), Edimburgo (2000), Washington (2002), Tóquio (2004), Seoul (2008) e Fortaleza (2013).

	**ASSINATURA**	**DATA**
Pesquisador Principal<br>**Prof. Dr. Gilberto De Nucci**<br>Galeno Desenvolvimento de Pesquisas Clínicas Ltda.	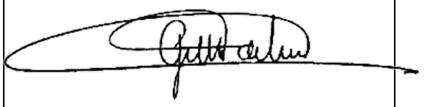	30.10.2020

SINOPSE

Produtos sob investigação, dose, e via de administração	Teste:	Heparina sódica nebulizada 25.000 UI (Solução injetável de monoparina 25.000 UI / mL, Wockhardt Reino Unido) – Será administrada uma dose aerossol nebulizada de heparina sódica 25.000 UI diluída com 4 mL em solução salina 0,9% através de nebulizador de rede vibratória.
	Controle:	Procedimento padrão protocolar em pacientes hospitalizados com COVID-19.

Código do projeto	**GDN 019/20**
**Objetivos Primários**	**Variáveis Associadas**
Avaliar o efeito da heparina na Mortalidade	Taxa de mortalidade em cada braço do estudo
Avaliar o efeito da heparina no tempo de internação	Tempo para alta hospitalar (em dias)
**Objetivos Secundários**	**Variáveis Associadas**
Avaliar o prognóstico da doença	Modificação na escala ordinal em 3, 7 e 14 dias (ou até o final da internação)<br>Dias que exigem tratamento de oxigênio
Avaliar o efeito da heparina na recuperação da COVID-19	1 – Tempo até ausência de sintomas<br>2 – Diminuição quantitativa dos sintomas
Avaliar o efeito da heparina na necessidade de ventilação (não invasiva e mecânica)	Dias até ventilação mecânica invasiva, se ocorrer

População de participantes	Os participantes elegíveis para este ensaio clínico serão homens e mulheres, de 18 a 80 anos hospitalizados com COVID-19 que não necessitam de ventilação mecânica imediata (pontos 3-5 na escala ordinal).<br>**Inicialmente, 100 pacientes, 50 em cada braço.**
Escala ordinal	1. Não hospitalizado.<br>2. Hospitalizado, não necessitando de oxigênio suplementar e não necessitando mais de cuidados médicos para COVID-19.<br>3. Hospitalizado não necessitando de oxigênio suplementar, mas precisando de cuidados médicos para COVID-19.<br>4. Hospitalizado exigindo oxigênio suplementar.<br>5. Hospitalizado exigindo ventilação não-invasiva ou oxigênio de alto fluxo.<br>6. Hospitalizado exigindo intubação e ventilação mecânica ou ECMO.<br>7. Morte

Desenho do estudo	Estudo controlado, aberto e randomizado, com administração de uma dose de aerossol nebulizada de 25.000 UI de heparina a cada 6 horas diluídas com 4 mL em solução salina a 0,9% através do Nebulizador Rede Vibratoria, e braço controle SoC (*Standard of care*).<br>As doses serão preparadas no momento da administração, como a seguir: uma ampola de 1 mL de heparina sódica 25.000 UI / mL sem conservante serão adicionados à câmara do Nebulizador Rede Vibratoria, seguidos por 4,0 mL para injeção de cloreto de sódio BP 0,9%. A câmara será montada e girada até total homogeneização da heparina.<br>A Heparina sódica nebulizada 25.000 UI será entregue utilizando Nebulizador Rede Vibratoria a cada 6 horas, diariamente por até 21 dias ou até o paciente não apresentar sintomas respiratórios.
<u>Avaliação de biomarcadores</u><br>• Tempos de coleta	Amostras de sangue serão coletadas no dia 1, 8, 15 e 29 para análises de citocinas em plasma.
• Quantidade de amostras e volume de sangue.	Serão coletadas 2 amostras contendo 5 mL de sangue cada, ou seja, 10 mL de sangue para avaliação de biomercadores. Serão coletadas ainda amostras para exames laboratoriais.
Avaliação de segurança	Segurança e tolerabilidade: tolerabilidade clínica (sinais e sintomas, eventos adversos e parâmetros laboratoriais), sinais vitais (pressão arterial, temperatura axilar e frequência de pulso).
Eventos adversos	Serão documentados os eventos adversos (EAs) observados, mencionados em função de um questionamento não específico ou relatados espontaneamente.
Plano para análise dos dados	A análise dos pontos finais co-primários não será corrigida para multiplicidade. Os dados de eficácia deste estudo de fase 2 serão avaliados após **60 pacientes** terem sido recrutados para permitir a reestimativa do tamanho da amostra para refletir os dados emergentes. Todos os pontos finais serão analisados usando uma taxa de erro tipo 1 = 0,05, mas as conclusões gerais deste primeiro estudo sobre os efeitos da heparina inalada no COVID-19 serão baseadas na avaliação de tendências numéricas em todos os pontos finais.

FLUXOGRAMA

	TRIAGEM	INCLUSÃO	Período de Tratamento (até alta hospitalar)			Acompanhamento	PÓS- ESTUDO
**Dias** * (± janela)	**-1 a 1**	**1º dia**		**15º (± 2 dias)**	**29º (± 2 dias)**	**60º (± 4 dias)**	**90º (± 6 dias)**
**ELIGIBILIDADE**							
Termo de Consentimento Livre e Esclarecido [E]	●						
Dados Demográficos	●						
História Médica [B]	●						
Diagnóstico SARS-CoV-2	●						
Verificação dos Critérios de Inclusão / Exclusão; Aprovação [H]	●						
ECG [D]	●						
**INTERVENÇÃO**							
Randomização		●					
Administração do produto (heparina não fracionda (UFH) inalada)			A cada 6 horas, diariamente, até 21 dias.				
Tratamento com cuidados padrão		●	●				
**PROCEDIMENTOS DE ESTUDO**							
Score fragilidade clínica	●						
Diagnóstico por imagem (rx e/ou tomografia computadorizada)	●						
Exame físico (incluindo sintomas, altura e peso)	●						
Exame físico direcionado (focado na ausculta pulmonar)			●				

**Dias** * (± janela)	**-1 a 1**	**1º dia**		**15º (± 2 dias)**	**29º (± 2 dias)**	**60º (± 4 dias)**	**90º (± 6 dias)**
	**TRIAGEM**	**INCLUSÃO**	**Período de Tratamento (até alta hospitalar)**			**Acompanhamento**	**PÓS- ESTUDO**
Sinais Vitais [C], incluindo frequência respiratória e SpO2		●	●	●	●		
Avaliações clínicas [F]		●	●	●			
Revisão de medicamentos direcionados (incluindo uso de vasopressores)		●	●	●	●		
Avaliação de eventos adversos		●	●	●	●	●	●
Avaliação de Co-infecção relacionada à doença (incluindo agente microbiológico/infeccioso avaliação/resultados; bactérias, virais, fungos)		●	●				
Status de sobrevivência		●	●	●	●	●	●
Gases sanguíneos e FiO2 na pior das hipóteses PO2 [I]	●	●	●	●			
**LABORATORIAL**							
Exames laboratoriais [G]	●	●	Dias 3, 5, 8, 11 (±1 dia) enquanto hospitalizado				
Teste de Gravidez (para mulheres)	●						
Avaliação de biomarcadores (coleta de sangue para análise exploratória de citocinas) [J]		●	Dia 8	●	●		
Coleta de sangue para exame laboratorial sorologia SARS-CoV-2		●					
PCR para SARS-CoV-2 swab orofaringe/nasal		●	Dias 3, 5, 8, 11 (±1 dia) enquanto hospitalizado	●	●		

FiO2=oxigênio inspirado fracionado; PCR= Reação em cadeia de polimerase; PO2= pressão parcial do oxigênio; SARS-CoV-2= síndrome respiratória aguda grave coronavírus 2; SpO2=saturação de oxigênio.

[*] Dias em relação a cada participante incluído no estudo.

[A] As visitas serão realizadas mesmo que um paciente já tenha recebido alta. Se dispensado antes da visita agendada, as visitas presenciais são preferidas, mas reconhecendo que a quarentena e outros fatores podem limitar a capacidade do paciente de retornar à clínica, essas visitas podem ser realizadas por telefone ou com uma visita domiciliar pelo pessoal do estudo. Para visitas realizadas por telefone, não será possível realizar algumas avaliações agendadas (por exemplo, sinais vitais). As avaliações do dia 29 também serão realizadas, sempre que possível, para pacientes que descontinuaram o estudo prematuramente.

[B] História Clínica incluindo data dos primeiros sintomas e comorbidades (por exemplo: resporatória, cardiovascular, matabólica, malignidade, endócrina, gastrointestinal, imunológica, renal), Exame Físico e avaliação dos Critérios de Inclusão;

[C] Pulso, Pressão Arterial, Temperatura, Frequência Respiratória e SpO2;

[D] ECG de 12 derivações;

[E] Assinatura do TCLE após esclarecidas todas as dúvidas com o pesquisador responsável;

[F] Inclui pontuação ordinal, Pontuação Nacional de Aviso Antecipado 2 (NEWS2), exigência de oxigênio, exigência de ventilador não invasivo ou invasivo, incluindo início e parada de suprimento de oxigênio de baixo ou alto fluxo ou de qualquer forma de ventilação etc.

[G] Exames laboratoriais hematologia, bioquímica e testes de coagulação (para paramêtros consulte a tabela no anexo I);
Serão aceitos exames laboratoriais realizados nas 48 horas anteriores à matrícula para determinação de elegibilidade. Quaisquer testes laboratoriais realizados como parte do atendimento clínico de rotina dentro da janela de visita especificada podem ser utilizados para testes laboratoriais de segurança. Amostras coletadas para processamento imediato de laboratório e armazenamento congelado.

[H] Após avaliação dos critérios de inclusão/exclusão e aprovação (ou não) do participante para o estudo;

[I] Os resultados dos gases sanguíneos serão totalmente registrados com data e hora;

[J] Coleta de amostras de 5 mL de sangue nos dias 1, 8, 15 (± 2 dias) e 29 (± 2 dias); Amostras coletadas, processadas e congeladas. Amostras coletadas dependentes da capacidade do centro de estudo.

LISTA DE ABREVIATURAS

PORTUGUÊS	INGLÊS	SIGNIFICADO
μg	μg	Micrograma
EA	AE	Evento adverso
EAG	SAE	Evento adverso grave
APE	TMF	Arquivo Principal do Ensaio (*Trial Master File*)
b.p.m.		Batimentos por minuto
IMC	BMI	Índice de Massa Corporal
FC		Frequência Cardíaca
FRC	CRF	Formulário de Relato de Caso
CNS		Conselho Nacional de Saúde
CONEP		Comissão Nacional de Ética em Pesquisa
ANVISA		Agência Nacional de Vigilância Sanitária
CV		Coeficiente de variação
ECG		Eletrocardiograma
EXCEL	EXCEL	Planilha de cálculos comercial
BPC	GCP	Boas Práticas Clínicas
BPF	GMP	Boas Práticas de Fabricação
BPL	GLP	Boas Práticas Laboratoriais
ECG		Eletrocardiograma
GGT		Gama glutamil transferase
h		Hora
ICH	ICH	*International Council for Harmonisation of Technical Requirements for Pharmaceuticals for Human Use* (Conselho Internacional para Harmonização dos requerimentos técnicos para os produtos farmacêuticos para uso humano)
CEP	IRB	Comitê de Ética em Pesquisa
POP	SOP	Procedimento Operacional Padrão
CQ	QC	Controle de Qualidade
EAS	SAE	Evento adverso sério
APE	APE	Arquivo Principal do Ensaio
TCLE		Termo de Consentimento Livre e Esclarecido
DPOC	COPD	Doença Pulmonar obstrutiva crônica
	SDS	Fonte de Dados/Formulário de dados fonte (Source Data Sheet)

FiO2	Oxigênio inspirado fracionado
PCR	Reação em cadeia de polimerase
PO2	Pressão parcial do oxigênio
SARS-CoV-2	Síndrome respiratória aguda grave coronavírus 2
SpO2	Saturação de oxigênio
UFH	Heparina não fracionada
IV	Intra venosa(o)
SoC	(*Standard of care)* Tratamento com cuidados padrão
BP	Brazilian Pharmacopeia, Farmacopéia Brasileira
PSI	Produto Sob Investigação

SUMÁRIO

1. INFORMAÇÕES GERAIS 12
2. INTRODUÇÃO 13
3. OBJETIVOS DO ESTUDO 17
4. PARTICIPANTES DO ESTUDO 20
5. SELEÇÃO DOS PARTICIPANTES PARA O ESTUDO E RETIRADA DOS PARTICIPANTES 20
6. ADMINISTRAÇÃO AOS PARTICIPANTES 27
7. ANÁLIDE EXPLORATÓRIA 28
8. AVALIAÇÃO DE SEGURANÇA 29
9. GERENCIAMENTO DE DADOS CLÍNICOS 36
9.1 Manuseio e documentação dos dados clínicos 36
9.2 GESTÃO E VALIDAÇÃO DOS DADOS 37
10. CONTROLE E GARANTIA DA QUALIDADE 37
11. MONITORIA 37
12. GARANTIA DE QUALIDADE 38
13. MANUTENÇÃO DOS REGISTROS 38
14. DOCUMENTOS FONTE E ACESSO AOS DADOS FONTE 38
15. Política de Publicação 38
16. ÉTICA 39
17. FINANCIAMENTO E SEGURO 41
18. DESVIOS DE PROTOCOLO 42
19. Referências 43
20. ANEXOS 45
ANEXO I 46
ANEXO II 47
ANEXO III 50
ANEXO IV 51
ANEXO V – Brochura do Investigador 52

1. INFORMAÇÕES GERAIS

1.1 Pesquisador Principal

Prof. Dr. Gilberto De Nucci
Avenida Jesuíno Marcondes Machado, 415
Nova Campinas
13092-320 - Campinas – SP - Brasil
Fone: (19) 3251-6928
denucci@gilbertodenucci.com

1.2 INSTITUIÇÕES ENVOLVIDAS E RESPONSABILIDADES

GALENO DESENVOLVIMENTO DE PESQUISAS CLÍNICAS
Rua Latino Coelho, 1.301
Campinas/SP
CEP:13087-010
Telefone:1932427133

ATCGEN PESQUISA E DESENVOLVIMENTO À SAÚDE EIRELI
Avenida Francisco Glicério, nº 1326, conjunto 64
Centro - Campinas/SP - CEP 13012-905

CASA DE SAÚDE CAMPINAS
Praça Dr. Toffoli, 28
Campinas - SP - CEP 13015-240
PABX: (19) 3736-3400

HOSPITAL VERA CRUZ
Av. Andrade Neves, 402
Campinas - SP, 13013-908
Telefone: (19) 3734-3000

DRS IMPORTADORA E SERVIÇOS
Rodovia Anhanguera, s/n km 15 – CLA 15 - GALPAO 40 SALA 04
Vila Jaguara – São Paulo - SP
CEP 05113-000

2. INTRODUÇÃO

Heparina pertence a uma família de glicosaminoglycanos polianiônicos (GAGs), polissacarídeos compostos de ácido hexaurônico e resíduos de D-glucosamina unidos por ligações glicosídicas. Atualmente, a heparina é autorizada para uso intravenoso (IV) e subcutâneo (SC) como um anticoagulante (Anatomical Therapeutic Chemical [ATC] BO1AB01) no grupo BO1A de agentes anti-trombóticos. No entanto, a heparina não fracionada (UFH) orientada para múltiplos alvos, com feridas mucolíticas atividades farmacológicas cicatrizantes, antiinflamatórias e antioxidantes que costumam ser não relacionado à anticoagulação quando inalado diretamente nas vias aéreas e ser considerado propriedades farmacológicas adicionais.

2.1 JUSTIFICATIVA PARA O ESTUDO

A heparina é amplamente utilizada como anticoagulante, no entanto, agora há uma ampla gama de evidências de que heparina pode exibir ampla atividade antiinflamatória tanto experimental quanto clinicamente, muitas vezes independente de sua atividade anticoagulante. Isso inclui a capacidade de neutralizar a atividade de um ampla gama de mediadores pró-inflamatórios[1]. Além disso, há agora uma série de estudos (descritos abaixo) que avaliaram os efeitos benéficos da UFH inalada na clínica de unidade de terapia intensiva (UTI) em pacientes queimados e em pacientes com doença pulmonar obstrutiva crônica (DPOC) [2,3,4,5] e enfatiza a importância do uso de heparina de baixo peso molecular como um anticoagulante em pacientes infectados com COVID-19, que deve ser administrado no início do tratamento.

Há evidências clínicas preliminares da China de que a heparina sistêmica mostrou algum benefício na redução do impacto do COVID-19 em pacientes gravemente enfermos.[4,5,6] Além disso, vários outros estudos em pacientes com exacerbações agudas de doença obstrutiva crônica das vias aéreas mostraram uma redução nos dias sem ventilação após a UFH inalada (25.000 UI) .[2,8,9]
Foi também observado, um benefício em pacientes com DPOC após UFH inalada em doses até 75.000 UI duas vezes ao dia (bid).[10,3] Nenhum desses estudos relatou quaisquer efeitos adversos de sangramento após UFH inalada, sugerindo que esta pode ser uma maneira relativamente segura de tratar pacientes COVID-19.

Os potenciais efeitos antivirais da UFH inalada foram revisados anteriormente.[1] Criticamente, UFH inibe a ligação ligação da proteína *spike* do vírus da síndrome respiratória aguda grave de coronavírus 2 (SARS-CoV-2) para o receptor específico da enzima de conversão da angiotensina 2 (ACE-2) receptor.[11] ACE-2 facilita a entrada viral nas células alvo[12] e é expressa em alvéolos pulmonares e brônquicos células epiteliais. [13]

Além disso, os glicosaminoglicanos (GAGs) são um alvo atraente para vírus por causa de sua ampla distribuição na superfície da maioria das células humanas, onde as interações iniciais com vírus ocorrem.[14] Vários estudos forneceram evidências de que a UFH compete com o *heparan sulphate proteoglycans* (HSPG) para a ligação a proteínas de superfície em vírus, incluindo tipos de coronavírus, e limita sua adesão a alvos celulares e, portanto, sua infecciosidade. [14]

Em pacientes com infecção das vias aéreas por SARS-CoV-2, verificou-se que as amostras de escarro retêm SARS-CoV-2 por 39 dias após os esfregaços da garganta.[15]

Portanto, os efeitos mucolíticos da UFH inalada pode também melhorar a eliminação do vírus das vias aéreas.

O objetivo deste estudo é investigar os efeitos terapêuticos e a segurança da UFH inalada em pacientes com COVID-19, pois, atualmente não há vacinas, medicamentos eficazes e apenas tratamentos para sintomas disponíveis.

O benefício / risco para o paciente é aceitável, pois, os dados demonstram que nenhum efeito toxicológico agudo ou crônico foi associado à via inalatória para qualquer dose da heparina investigada em humanos.

No geral, esses dados apoiam a conclusão de que o múltiplo efeitos farmacológicos da UFH inalada podem ter benefício terapêutico em pacientes com COVID-19.

2.2 DESCRIÇÃO DO PRODUTO

2.2.1 Justificativa de dose para Heparina Inalada

Heparina sódica 25.000 UI/6 horas (equivalente a 25 mg administrados aos pulmões/6 horas, assumindo uma atividade específica de 200 UI/mg, uma vez que, administração nebulizada fornece 20% da dose total pulmão por até 21 dias, ou até que o paciente não tenha sintomas respiratórios.

Desde a descoberta da heparina em 1916, há muitos estudos toxicológicos de UFH em roedores e outros pequenos animais, indicando a segurança desta abordagem. Tecido pulmonar de cães que receberam heparina intrapulmonar (10 a 15 mg/kg por

administração intratraqueal) por um ano e não apresentou sinais de hemorragia, anemia ou lesões ulcerativas.[16] Quando a heparina foi administrada por via intratraqueal em coelhos usando um dispositivo de micropulverização a cada 48 horas em doses crescentes de 0,2, 2, 20, 100 e 200 mg/kg, sinais de sangramento foram vistos apenas nas 2 maiores doses.[17]

Nenhum efeito toxicológico agudo ou crônico foi associado à via inalatória para qualquer dose de heparina no homem. Em indivíduos humanos saudáveis, 300 a 1500 mg de heparina foram administrados com um aerossol sem sinal de toxicidade.[16]

Não foi relatado nenhuma incidência de hemorragia pulmonar associada a UFH inalada em qualquer estudo em um total de 536 pacientes com lesão por inalação de fumaça, lesão pulmonar aguda, asma e alergia.[18] Nem mesmo a lavagem pulmonar com administração intratraqueal de 200 a 250 mg/kg de heparina para tratar o sangramento induzido por proteinose alveolar no pulmões foi associado a danos pulmonares um ano depois.[16] A UFH inalada não atravessa facilmente a mucosa brônquica. Em cães, camundongos, ratos e homens, doses altas de heparina intrapulmonar > 8mg/kg estão associadas a anticoagulação sistêmica. [16,17] Além disso, a UFH inalada não induziu reações alérgicas em qualquer estudo, nem mesmo quando acompanhados por longos períodos de até 485 dias. Em um estudo do efeito da UFH inalada em pacientes com fibrose pulmonar idiopática, a dose de 750 mg de preenchimento do nebulizador foi considerada a dose limite.[19] Em uma fase compassiva do estudo, os pacientes continuaram a usar UFH inalatória por até 100 semanas, sem efeitos colaterais. Estudos anteriores[1] indicaram que a inalação de heparina trata a inflamação local, muco hipersecreção e lesão pulmonar, sem anticoagulação sistêmica (20 estudos, 536 pacientes) [18] e é seguro e eficaz em pacientes com lesão por inalação de fumaça, lesão pulmonar aguda, asma e alergia, fibrose cística 20 e DPOC. Em outro estudo clínico em pacientes com DPOC moderada a grave, aproximadamente, 60 mg de UFH administrada ao pulmão melhorou significativamente a função pulmonar,
capacidade de exercício e dispneia sem evidência de toxicidade ou efeitos colaterais adversos nesta dose.[3]

Para COVID-19, a UFH inalada pode oferecer benefícios aos pacientes para reduzir as complicações decorrentes de uma tempestade de citocinas e para prevenir a magnitude dos efeitos das citocinas em pacientes com doença induzida por COVID-19 moderada a grave. Os dados recentes sugerem que UFH inalada também pode se ligar à proteína *Spike* [1] que o vírus COVID-19 usa para entrar nas células e pode fornecer um efeito antiviral adicional em pacientes com COVID-19. Aqueles com consolidação pneumônica e o consequente aumento da produção de expectoração será tratado pelo efeito mucolítico da inalação UFH.[3,21]

2.2.2 Indicações:

A UFH (heparina não fracionada) inalada foi investigada em lesão pulmonar aguda, em pacientes ventilados, aqueles com queimaduras por inalação, pacientes com asma, DPOC, IPF e FC. Em nenhum estudo tem qualquer evento adverso associado a esta via de administração. Apenas altas doses > 8 mg / kg estão associadas à anticoagulação sistêmica.

2.2.3 Contraindicações:

O produto sob investigação IV é contraindicado para o uso concomitante de outros anticoagulantes, antiinflamatórios não esteróide e inibidores da enzima de conversão da angiotensina. No entanto, não existe nenhuma contraindicação relatada para os efeitos pulmonares locais da inalação UFH na dose proposta.

2.2.4 Precauções e advertências:

Mesmo doses maciças de heparina intrapulmonar não estão associadas a doenças pulmonares, hemorragia ou dependência. No entanto, o sulfato de protamina é um antídoto apropriado para overdose de heparina.

2.2.5 Eventos Adversos:

A heparina intravenosa é aprovada e geralmente bem tolerada. As reações adversas relatadas de acordo com a rotulagem aprovada para heparina IV incluem, hemorragia, trombocitopenia induzida por heparina (HIT) e trombocitopenia induzida por heparina e trombose (HITT), trombocitopenia, resistência à heparina e hipersensibilidade. Contudo, esses eventos não foram relatados com UFH inalada devido à absorção sistêmica reduzida.

Os pacientes que recebem UFH inalada podem esperar expectorar grandes volumes de expectoração, refletindo o efeito farmacológico de um mucolítico, e embora este não seja um EA, os pacientes podem pontuar este efeito negativo nas escalas visuais analógicas que refletem a imagem corporal ou marginalização social.[20]

Portanto, os pacientes devem ser informados sobre este provável efeito e que é um efeito benéfico (Brochura do Investigador).

Além disso, os possíveis efeitos adversos associados à UFH inalada incluem:

• Dor de cabeça

• Epistaxe

• Anticoagulação sistêmica (isso não é esperado com a dose inalada usada)

• Hemoptise (embora, por todas as razões apresentadas acima, seja improvável que esteja associada com UFH inalada e é provável que seja observado no grupo placebo).

2.2.7 Cuidados de conservação:

Os frascos devem ser armazenados entre 2ºC e 8ºC e NÃO devem ser congelados.

2.3 DECLARAÇÃO QUANTO À ADERÊNCIA DO ESTUDO AO PROTOCOLO, BOAS PRÁTICAS CLÍNICAS (BPC) E REQUISITOS LEGAIS APLICÁVEIS

Este estudo será conduzido de acordo com as Boas Práticas Clínicas (BPC) e demais recomendações estabelecidas pela ICH, bem como em conformidade com as Resoluções 466/12 e 251/97 do Conselho Nacional de Saúde - Ministério da Saúde e Resolução RDC 56/2014 da ANVISA e resoluções complementares.

3. OBJETIVOS DO ESTUDO

Este estudo tem por **objetivos primários** avaliar o efeito da heparina não fracionada (UFH) inalada na **mortalidade** por COVID-19 em pacientes hospitalizados, quando administrada, em uma dose aerossol nebulizada, avaliando-se também o efeito da heparina na **duração da internação**.

O estudo tem como **objetivos secundários:**

- Avaliar o prognóstico da doença
- Avaliar o efeito da heparina na recuperação da COVID-19, por meio da avaliação do Tempo até ausência de sintomas, e diminuição quantitativa dos sintomas
- Avaliar o efeito da heparina na necessidade de ventilação (não invasiva e mecânica);

A heparina é um anticoagulante amplamente utilizado que possui atividades farmacológicas adicionais relevantes para o tratamento da COVID-19,[1] tem atividade anti-inflamatória [2] e atenua as replicações do SARS-CoV2. A Heparina inalada mostrou eficácia em ensaios clínicos de pacientes com ARDS, DPOC e fibrose cística,

potencialmente devido aos seus efeitos anti-inflamatórios e mucolíticos. Este estudo investigará os efeitos da heparina não refratada inalada (UFH) em pacientes hospitalizados com COVID-19. A UFH não entra na circulação sistêmica e, portanto, pode ser usada em combinação com a anticomação sistêmica.

Além disso, serão avaliadas a segurança e a tolerabilidade do produto com base na ocorrência de eventos adversos.

3.1 DESENHO DO ESTUDO

3.1.1. DESCRIÇÃO DO TIPO/DESENHO DO ESTUDO A SER CONDUZIDO

Estudo clínico fase 2, controlado, aberto, randomizado, com tratamento pela administração de uma dose de aerossol nebulizada de 25.000 UI de heparina a cada 6 horas diluídas com 4 mL em solução salina a 0,9% através do Nebulizador Rede Vibratoria.

Serão selecionados inicialmente 100 pacientes de ambos os sexos, entre 18 e 80 anos, hospitalizados com COVID-19 que não necessitam de ventilação mecânica imediata (pontos 3-5 na escala ordinal) capaz de fornecer o consentimento livre e esclarecido.

Os pacientes serão divididos em dois grupos de acordo com a randomização:

• Grupo teste: 50 pacientes receberão tratamento com o medicamento Heparina (UFH).

• Grupo controle: 50 pacientes receberão, o tratamento protocolar hospitalar para pacientes com COVID-19, e serão monitorados.

As doses serão preparadas imediatamente antes do uso, como a seguir:

O Conteúdo de uma ampola de 1 mL de heparina sódica 25.000 UI / mL sem conservante será adicionado à câmara do nebulizador, seguidos por 4,0 mL de solução para injeção de cloreto de sódio BP 0,9%. A câmara será montada e girada para misturar a heparina completamente.

A Heparina sódica nebulizada 25.000 UI será entregue utilizando Nebulizador Rede Vibratoria a cada 6 horas, diariamente por até 21 dias ou até o paciente não apresentar sintomas respiratórios.

Os participantes serão aleatoreamente designados a um dos braços de tratamento.

A randomização será efetuada por *software*. A tabela de randomização indicará o paciente que deverá receber o tratamento com o produto teste, por conseguinte, o

paciente que não receberá o produto teste, mas somente o tratamento padrão do hospital.

3.2 DESCRIÇÃO DAS MEDIDAS TOMADAS PARA MINIMIZAR/EVITAR TENDÊNCIAS

O estudo será aberto e aleatorizado. Os braço de tratamento ou observacional atribuídos a cada participante no estudo serão determinados por uma lista de aleatorização. O número apropriado do participante será alocado sequencialmente para cada participante e cuja entrada na parte aleatorizada do ensaio for confirmada.

Todos os participantes realizarão os mesmos procedimentos e avaliações previstas para cada uma das ocasiões.

3.1.1. VARIÁVEIS A SEREM ANALISADAS

Estão previstas as análises das variáveis a seguir, o que pode sofrer alteração quando da análise estatística, e a critério do investigador principal.

**Objetivos Primários**	**Variáveis**
Avaliar o efeito da heparina na Mortalidade	Taxa de mortalidade em cada braço do estudo
Avaliar o efeito da heparina no tempo de internação	Tempo para alta hospitalar (em dias)
**Objetivos Secundários**	**Variáveis**
Avaliar o prognóstico da doença	Modificação na escala ordinal em 3, 7 e 14 dias (ou até o final da internação)
	Dias que exigem tratamento de oxigênio
Avaliar o efeito da heparina na recuperação da COVID-19	1 – Tempo até ausência de sintomas<br>2 – Diminuição quantitativa dos sintomas
Avaliar o efeito da heparina na necessidade de ventilação (não invasiva e mecânica)	Dias até ventilação mecânica invasiva, se ocorrer
Avaliar a necessidade de ventilação invasiva	Número de dias que requerem ventilação
Redução da carga viral com o tratamento	Carga viral SARSCoV2 determinada por PCR a partir de swab nasofaringe ou orofaringe

4. PARTICIPANTES DO ESTUDO

4.1. POPULAÇÃO DO ESTUDO

4.1.1. População Alvo

Pacientes de ambos os sexos, hospitalizados com diagnóstico de COVID-19 que não necessitam de ventilação mecânica imediata (pontos 3-5 na escala ordinal).

4.1.2. População Acessível

A população acessível será constituída dos pacientes que se apresentarem a um dos centros de ensaios clínicos selecionados para condução deste ensaio clínico, e que não tenham participado de ensaio clínico nos últimos doze meses.

4.1.3. Amostra

Tamanho da amostra: 100 participantes de ambos os sexos, com idade igual ou superior a 18 anos.

A partir da população de participantes que comparecerem aos centros de pesquisas, serão selecionados incialmente 100 participantes, 50 em cada braço para esse estudo, conforme se enquadrem nos critérios de elegibilidade do estudo.

4.2. MÉTODO DE AMOSTRAGEM

Amostragem por conveniência, sem estratificação.

5. SELEÇÃO DOS PARTICIPANTES PARA O ESTUDO E RETIRADA DOS PARTICIPANTES

Após ter lido o protocolo do estudo, o pesquisador responsável, ou membro da equipe por ele designado, considerará os pacientes elegíveis levando em conta os critérios de inclusão e exclusão, selecionará um número de participantes adequado para atender às necessidades de inclusão no estudo, após a obtenção do consentimento informado e assinatura do Termo de Consentimento Livre e Esclarecido.

Serão selecionados e incluídos no estudo 100 participantes de ambos os sexos, diretamente da população hospitalar.

Os participantes só serão admitidos no estudo após avaliação, pelo pesquisador, dos resultados dos exames, em conjunto com as demais informações obtidas durante a avaliação médica.

Para fins de avaliação, durante o processo de triagem, os participantes realizarão os testes apresentados na Tabela 1 a seguir:

Tabela 1: Itens da história clínica e exame físico a serem explicitamente referenciados no FRC

Categoria	Exames
História Médica	Alergias; olhos, nariz e garganta; sistema respiratório, cardiovascular, gastrintestinal, genitourinário, nervoso central, hematopoiético, linfático, endócrino; dermatológico, musculoesquelético; estabilidade emocional, história familiar e cirúrgica.
Exame Físico	Olhos, orelhas, nariz, garganta, pescoço (incluindo tireoide), coração, pulmões, abdômen (incluindo fígado e baço), pele, linfonodos, urogenital, sistema nervoso, esqueleto e músculos.
Dados Antropométricos	Pressão arterial, pulso, frequencia respiratória, saturação (SPO2), altura, peso e temperatura axilar em ^{o}C.
Testes específicos	Teste positivo para COVID-19.

Participantes do sexo feminino só poderão participar do estudo caso não estejam grávidas, o que deverá ser comprovado por um exame de β-HCG durante a triagem.

Eletrocardiograma, exames hematológicos e bioquímicos, história médica e exame físico também serão utilizados para a avaliação de segurança.

Após a aprovação na consulta médica, durante o processo de seleção, os participantes realizarão os testes apresentados na Tabela 2, para complementação da avaliação.

Tabela 2: Exames Laboratoriais vinculados ao Processo de Seleção dos Participantes

Categoria	Exames
Análise hematológica	Hemoglobina, hematócrito, contagem total e diferencial de leucócitos, contagem de glóbulos vermelhos e contagem de plaquetas.
Coagulação	Dímero d<br>Fibrinogênio<br>Tempo de tromboplastina parcial ativado<br>Tempo de protrombina
Análise Bioquímica	Sódio, potássio, cálcio, magnésio, fósforo inorgânico, fosfatase alcalina, bicarbonato, creatinoquinase, glicemia de jejum, bilirrunina total e frações, TGO, TGP, GGT, desidrogenase láctica, triglicérides, proteína C reativa ultra-sensível, ferritina, troponina cardíaca-T<br>IGG e IGM

PCR	PCR Orofaringeo/nasal para SARS-Cov-2
Teste de gravidez	β-HCG para mulheres.
Imagem	Rx e/ou Tomografia computadorizada

Os valores de referência, fornecidos pelo laboratório clínico, encontram-se disponíveis no Arquivo Principal do Ensaio (APE-*TMF*). Alterações dos *kits* dos testes utilizados, após a aprovação deste protocolo, resultando em novas faixas de normalidade, serão referenciadas nos FRCs.

No que se refere ao eletrocardiograma, o médico responsável por interpretar o ECG deverá informar se os achados específicos são julgados como normal, anormal não clinicamente significativo ("n.c.s.") ou anormal. O traçado e o laudo do ECG deverão ser mantidos como parte da documentação do estudo.

Após terem sido prestadas informações adicionais relativas ao estudo clínico em questão e esclarecidas todas as dúvidas restantes, caso concordem em participar do ensaio clínico, os participantes assinarão o Termo de Consentimento Livre e Esclarecido, juntamente com o pesquisador responsável (ou membro da equipe por ele delegado, responsável pela obtenção do consentimento informado), em duas vias, sendo uma das vias entregue ao participante e a outra armazenada no centro de ensaios clínicos.

Os candidatos que fornecerem o consentimento para participação no ensaio clínico terão os critérios de inclusão e exclusão avaliados, bem como as restrições cuidadosamente verificadas. Os participantes também serão observados quanto às condições emocionais para inclusão no estudo.

Após o investigador confirmar a elegibilidade do participante para inclusão no estudo, o participante será informado e se o mesmo reafirmar o desejo de participar do ensaio clínico, será então incluído.

Após o término do tratamento, serão realizadas as avaliações de evolução, além das avaliações de alta.

No entanto, participantes que apresentem eventos adversos serão acompanhados clinicamente, independentemente da conclusão do estudo.

Além disto, para os participantes que completarem o tratamento e permanecerem no estudo, serão realizados, como acompanhamento pós-tratamento, no dia 29 os seguintes procedimentos:

- Sinais vitais (pressão arterial, pulso, temperatura axilar, frequência respiratória e SPO2);
- Verificação quanto ao uso de medicação concomitante;
- Avaliação quanto ao bem-estar geral através da avaliação de EAs;
- Coleta de sangue para avaliação de biomarcadores;

- Coleta PCR para SARS-CoV-2 swab orofaringe/nasal;

No dia 60 (acompanhamento pós-tratamento) serão realizados os seguintes procedimentos:

- Avaliação quanto ao bem-estar geral através da avaliação de EAs;

No dia 90 (avalição pós-tratamento) serão realizados os seguintes procedimentos:

- Avaliação quanto ao bem-estar geral através da avaliação de EAs;

5.1. CRITÉRIOS DE INCLUSÃO DO PARTICIPANTE

Os seguintes critérios devem ser satisfeitos para que o participante possa participar do estudo:

1. Pacientes hospitalizados com COVID-19 que não necessitam de ventilação mecânica imediata (pontos 3-5 na escala ordinal);
2. Idade de 18 a 80 anos;
3. Capaz de compreender a natureza e o objetivo do estudo, inclusive os riscos e efeitos adversos e com intenção de cooperar com o pesquisador e agir de acordo com os requerimentos de todo o ensaio, o que vem a ser confirmado mediante a assinatura do Termo de Consentimento Livre e Esclarecido, antes da realização de qualquer procedimento do estudo;
4. As mulheres com potencial para engravidar deverão fazer uso de método contraceptivo seguro e eficaz durante todo o estudo. Se a participante optar pela abstinência sexual ou praticar algum tipo de relação que não tenha riscos de gravidez, o uso do contraceptivo não será obrigatório.

5.2. CRITÉRIOS DE EXCLUSÃO DO PARTICIPANTE

A resposta positiva a qualquer um dos seguintes critérios excluirá o participante do estudo:

Problemas relacionados com a droga:

1- O participante tem sabidamente uma hipersensibilidade à droga estudada (heparina) ou a compostos quimicamente relacionados;

2- História de reações adversas graves ou hipersensibilidade a qualquer droga;

Outras condições:

3- O participante tem qualquer condição que o impede de participar do estudo pelo julgamento do investigador;

4- Participante apresenta resultado positivo para teste de β-HCG e/ou teste de urina para gravidez; parto ou aborto nas 12 semanas anteriores à data prevista para administração da vacina.

5- Apresentar DPOC

5.2.1. Critérios de Descontinuação do Participante

Solicitação por parte do participante para se retirar do estudo a qualquer momento:

1- Participante não deseja continuar no estudo por qualquer razão (motivos pessoais, indisponibilidade, intolerância aos procedimentos do estudo, ocorrência de evento adverso ou mesmo sem razão);

O pesquisador poderá retirar o participante do estudo por uma das seguintes razões:

2- Não aderência às exigências do protocolo;

3- Eventos adversos, reações adversas à droga, sintomas ou sinais de possível toxicidade;

4- Resposta positiva à reavaliação de qualquer um dos critérios de exclusão durante a condução do estudo;

5- Qualquer outra condição que, a critério do pesquisador, seja de interesse para manutenção da saúde do participante.

6- Broncoespasmo que não pode ser melhorado com broncodilatadores.

7- Hipóxia súbita: a saturação de oxigênio deve ser monitorada durante a nebulização de UFH.

8- Transferência do participante para UTI, e/ou intubação.

Todos os detalhes e razões para a retirada do participante do estudo deverão ser anotados na parte do prontuário destinada (Seção de Término do Estudo).

5.3 DESCRIÇÃO DAS "REGRAS DE PARADA" OU "CRITÉRIOS DE DESCONTINUAÇÃO" PARA OS PARTICIPANTES INDIVIDUALMENTE, PARTE DO ESTUDO E/OU TODO ESTUDO

Os participantes poderão ter sua participação no estudo encerrada antecipadamente em função dos "Critérios de Descontiunação do Participante" no item 5.2.1 deste protocolo.

Além disso, com o objetivo de garantir o bem-estar dos participantes, o estudo poderá ser interrompido em parte ou como um todo, caso, a juízo do pesquisador principal, seja estabelecido que os riscos a que os participantes estão sendo submetidos sejam superiores aos antecipadamente previstos.

Em caso de desistência individual de um participante antes do dia 1, o mesmo será considerado como falha de triagem. Não é recomendada a substituição de "*dropouts*" após o início do estudo.

5.4 Responsabilidade sobre os PSI

O envio do produto sob investigação será através da empresa DRS importadora e serviços. O uso e destino dos PSI devem ser documentados. Após a conclusão do ensaio clínico, os recipientes incluindo quaisquer PSI restantes, embalagens vazias, bem como a medicação de ensaio utilizada serão encaminhados para a central de armazenamento de medicamentos e tratados conforme descrito em um POP. É necessária uma explicação por escrito sobre a disposição de produtos ausentes ou suas embalagens.

No início do estudo, o pesquisador principal, ou responsável por ele designado, enviará, à unidade hospitalar, quantidade suficiente da formulação para a realização do ensaio. Após o ensaio, a medicação remanescente será enviada de volta ao pesquisador principal em suas embalagens originais, incluindo as parcialmente utilizadas e as não utilizadas.

Cabe ao pesquisador principal, ou ao responsável por ele designado, manter um inventário com o registro das drogas recebidas, administradas, destruídas ou retidas.

5.5 IDENTIFICAÇÃO DOS DADOS A SEREM REGISTRADOS DIRETAMENTE NO PRONTUÁRIO MÉDICO

Os seguintes dados serão registrados, sendo este a fonte de dados brutos:

- História médica e exame físico;
- Dados antropométricos (altura, peso);

- Sinais vitais (pressão arterial, pulso, frequencia cárdica, SPO2, temperatura axilar);

- Uso de medicamentos pré ou concomitantemente ao produto sob investigação;

- Administração do produto sob investigação e respectivos horários;

- Registro de eventos adversos e eventos adversos graves.

6. ADMINISTRAÇÃO AOS PARTICIPANTES

6.1. ADMINISTRAÇÕES A SEREM REALIZADAS

6.1.1. Produto Sob Investigação

FORMULAÇÃO TESTE:

Heparina sódica nebulizada 25.000 UI (Solução injetável de monoparina 25.000 UI / mL, Wockhardt Reino Unido).

A heparina será fornecida em ampolas de 1 mL.

Será administrada uma dose de 1mL de heparina sódica 25.000 UI diluída com 4 mL em solução salina 0,9% nebulizada através de nebulizador de drogas em aerossol a cada 6 horas.

As diluições serão preparadas no momento da administração como a seguir:

Uma (1) ampola de 1 mL de heparina sódica 25.000 IU/mL sem conservante será adicionado à câmara do nebulizador seguido por 4,0 mL de cloreto de sódio BP 0,9%. A câmara será montada e girada para homogeneização.

A heparina sódica nebulizada 25.000 UI será fornecida cada 6 horas, diariamente por até 21 dias, ou até que o paciente fique sem sintomas respiratórios.

A saturação de oxigênio deve ser monitorada durante a nebulização de UFH.

6.1.2. TOXICIDADE

Não foram associados efeitos toxicológicos crônicos à via de administração inalatórioa para qualquer dose de heparina investigados nos seres humanos. Estudos em participantes saudáveis não desmonstram absorção sistêmica detectável de UFH inalada.

Embora a reação alérgica não tenha sido relatada em nenhum estudo, a anafilaxia é uma possibilidade teórica. Caso ocorra, deve ser tratada de acordo com o padrão de atendimento.

6.1.3. Medicamentos Concomitantes

Não há restrições tampouco contra-indicações para medicamentos concomitantes para o estudo em questão, exceto outros anticoagulantes, antiinflamatórios não esteróide e inibidores da enzima de conversão da angiotensina conforme item 2.2.3.

7. ANÁLIDE EXPLORATÓRIA

Quando apropriado, as demais variáveis do estudo como análise de citocinas inflamatórias podem ser testadas, ou outras a critério da equipe médica, visando a segurança do participante.

7.1. OBTENÇÃO DO MATERIAL PARA ANÁLISE DE CITOCINAS

Serão coletadas amostras de 5 mL de sangue em tubos SST de acordo com fluxograma deste protocolo (página 6 e 7).

7.2. PROCESSAMENTO E ARMAZENAMENTO INICIAL DAS AMOSTRAS

As amostras de sangue (no período máximo de 1 hora após a coleta) serão centrifugadas em torno de 2000 x g por 10 minutos à baixa temperatura (4°C). Imediatamente após a centrifugação, o plasma será retirado e armazenado em frasco adequado, igualmente identificado, à temperatura de (-20ºC) em freezer específico, para armazenagem de amostras biológicas, localizado na própria Unidade Clínica do Centro Galeno.

7.3. ARMAZENAMENTO E TRANSPORTE DAS AMOSTRAS

Após o término do período de estudo, os frascos já identificados individualmente, contendo as amostras de plasma, serão embalados por participante e por período do estudo. O transporte das amostras para a Unidade Analítica se dará assim que possível, de acordo com o procedimento operacional padrão (POP) para Transporte de Amostras vigente à época de condução do estudo.

8. AVALIAÇÃO DE SEGURANÇA

8.1. ESPECIFICAÇÃO DOS PARÂMETROS DE SEGURANÇA

Para fins de acompanhamento de segurança, os participantes serão avaliados, durante o período de hospitalização e após alta hospitalar nos dias 29, 60 e 90. Os participantes serão acompanhados durante o estudo, visando à detecção de eventos adversos.

8.1.1 Definição de Evento Adverso (EA)

Um evento adverso é qualquer ocorrência médica adversa, em um paciente ou participante de ensaio clínico a quem um produto farmacêutico foi administrado, e que não necessariamente tem uma relação causal ao tratamento/produto. Como resultado, um evento adverso pode ser qualquer sinal, sintoma ou doença desfavorável e não intencional (incluindo resultados fora da faixa de referência), associado com o uso de um produto sob investigação, quer seja relacionado a ele ou não.

A "ameaça a vida" refere-se a condições que, efetivamente encontradas no momento da ocorrência do evento, requeiram intervenção imediata para evitar a ocorrência de quaisquer dos desfechos listados anteriormente; não se refere à situação hipotética de possibilidade de "ameaça a vida" caso o evento fosse mais severo.

Todos os participantes, inclusive os que desistiram da participação no estudo após a administração de pelo menos uma dose do produto sob investigação, deverão ser reavaliados clinicamente (incluindo aferição de sinais vitais, realização de exame físico e por exames laboratoriais caso seja necessário).

Além da comunicação de eventos pelo participante (relato espontâneo) ou identificados por simples observação por parte da equipe do centro de ensaios clínicos, o pesquisador deverá registrar e avaliar as seguintes variáveis, visando à detecção de eventos adversos:

• Sinais vitais dos participantes (FC, PA e temperatura) aferidos durante o estudo;
• Achados de exame físico, obtidos ao final do estudo e avaliados de forma comparativa àqueles obtidos na fase de seleção dos participantes.

Os eventos adversos deverão ser documentados e notificados de acordo com os critérios e definições constantes no presente protocolo.

8.2.2 Definição de Evento Adverso Grave

Um evento adverso grave é aquele que resulte em qualquer experiência adversa com medicamentos, produtos biológicos ou dispositivos, ocorrendo em qualquer dose e que resulte em qualquer um dos seguintes desfechos:

1- Óbito;

2- Ameaça à vida;

3- Incapacidade/invalidez persistente ou significativa;

4- Exige internação hospitalar ou prolonga a internação já existente;

5- Anomalia congênita ou defeito de nascimento;

6- Qualquer suspeita de transmissão de agente infeccioso por meio de um medicamento;

7- Evento clinicamente significante.

Reação Adversa a Medicamento:

- Para um novo medicamento ou seus novos usos, todas as respostas nocivas e não intencionais, relacionadas com qualquer dose, devem ser consideradas Reações Adversas a Medicamento. "Respostas a um medicamento" significa que uma relação causal entre um medicamento e um evento adverso é, pelo menos, uma possibilidade razoável, isto é, a relação não pode ser descartada.

- No que se refere aos medicamentos comercializados, uma resposta (nociva e não intencional), a uma droga, que ocorre com doses geralmente utilizadas no homem para a profilaxia, diagnóstico ou tratamento de doenças ou para a modificação de uma função fisiológica.

Reação Adversa a Medicamento - Inesperada: Uma Reação Adversa a Medicamento, cuja natureza ou a gravidade não é consistente com as informações aplicáveis ao produto (por exemplo: Brochura do Investigador para um produto sob investigação não aprovado ou bula / Sumário de Características do medicamento para um produto aprovado).

8.2.3 CLASSIFICAÇÃO E AVALIAÇÃO DE UM EVENTO ADVERSO

A classificação e a avaliação quanto à intensidade, à previsibilidade, à causalidade, à ação tomada e ao desfecho dos eventos adversos serão realizadas pelo investigador de acordo com as regulamentações locais aplicáveis e guias vigentes da Organização Mundial da Saúde, conforme detalhado a seguir.

8.2.3.1 Classificação e Avaliação de Intensidade

No decorrer do ensaio clínico, o investigador deverá determinar se ocorreu qualquer EA e deverá classificar sua intensidade como segue:

Leve O evento adverso prejudica o nível funcional normal do participante apenas levemente, não sendo necessária a administração de um antagonista**.**

Moderado O evento adverso prejudica o nível funcional normal do participante e/ou requer a administração de um medicamento ou a mudança do medicamento utilizado na terapia.

Severo O evento adverso representa um claro e acentuado prejuízo do nível funcional normal do participante, causando danos permanentes ou requerendo tratamento médico intensivo.

Letal O evento adverso contribui direta ou indiretamente para a morte do participante.

Deve ser considerada para a classificação a intensidade máxima observada para o evento adverso.

8.2.3.2 Classificação de Previsibilidade de Evento Adverso

Para classificação de previsibilidade dos eventos adversos relatados durante o ensaio clínico, considera-se o documento de referência de segurança do medicamento (Brochura do Investigador ou Sumário de Características do Medicamento).

Inesperado: Um evento adverso cuja natureza e/ou intensidade não são coerentes com as informações constantes no documento de referência de segurança do medicamento, ou que sejam inesperadas de acordo com as características do medicamento.

Esperado: Um evento adverso cuja natureza e/ou intensidade são coerentes com as informações constantes no documento de referência de segurança do medicamento.

8.2.3.3 Classificação de Causalidade do Evento Adverso

Cada evento adverso ocorrido durante o ensaio clínico deve ser avaliado quanto à sua relação com o produto sob investigação administrado.

A relação causal de um evento adverso com o(s) produto(s) sob investigação será classificada como segue:

- Definida/Comprovada: um evento clínico, inclusive alteração em exames laboratoriais, que se manifesta com uma sequência temporal plausível em relação à administração do medicamento e que não pode ser explicado pela doença de base nem pelo efeito de outros medicamentos ou substâncias. A resposta à suspensão do medicamento (retirada ou dechallenge, do inglês) deve ser clinicamente plausível. O evento deve ser definitivo do ponto de vista farmacológico ou fenomenológico; se necessário, pode-se lançar mão de um teste de reexposição ao medicamento (em inglês, rechallenge) para se obter resultados conclusivos;

- Provável: um evento clínico, inclusive alteração em exames laboratoriais, que se manifesta com uma sequência temporal razoavelmente plausível em relação à administração do medicamento, que dificilmente seria atribuível à doença intercorrente ou a outros medicamentos ou substâncias, e que apresenta resposta clinicamente razoável à suspensão do medicamento (dechallenge). Não é preciso ter informação sobre reexposição (rechallenge) para aplicar esta definição;

- Possível: um evento clínico, inclusive alteração em exames laboratoriais, que se manifesta com uma sequência temporal razoavelmente plausível em relação à administração do medicamento, mas que também pode ser explicado pela doença de base ou pelo efeito de outros medicamentos ou substâncias. A informação a respeito da suspensão do medicamento pode faltar ou não estar clara;

- Improvável/Duvidosa: um evento clínico, inclusive alteração em exames laboratoriais, que se manifesta com uma sequência temporal improvável em relação à administração do medicamento, e que pode ser explicado de modo mais plausível pela doença de base ou pelo efeito de outros medicamentos ou substâncias;

- Condicional: a sequência temporal é razoável e a reação não seria explicada pelo estado clínico subjacente do participante, mas o quadro apresentado não é conhecido como efeito indesejável do medicamento utilizado;

- Não avaliável: relatos nos quais não é possível estabelecer causalidade devido a evidências insuficientes, a dados conflitantes ou a documentação precária.

8.2.3.4 Informações sobre Ação Tomada quanto ao PSI

As seguintes ações poderão ser tomadas com relação à aplicação do produto sob investigação mediante a ocorrência de eventos adversos:

- Nenhuma ação;
- Suspensão do tratamento;
- Interrupção temporária do tratamento;
- Não se aplica (caso o tratamento já tenha sido finalizado).

14.2.5 Especificação do Desfecho de Evento Adverso

O desfecho de um evento adverso deverá ser classificado em:

- Resolvido;
- Resolvido com sequelas;
- Ainda não resolvido;
- Não resolvido;
- Óbito;
- Desconhecido.

8.2 ANÁLISE DOS PARÂMETROS DE SEGURANÇA

8.2.1 Procedimento durante o estudo

Nos dias de administração do produto sob investigação, os participantes permanecerão internados e serão observados pelo pesquisador responsável e/ou membros de sua equipe, durante todo o estudo, visando à detecção de eventos adversos, incluindo sinais de toxicidade.

Os participantes deverão estar instruídos sobre os efeitos adversos em potencial, bem como sobre a necessidade de comunicar imediatamente qualquer ocorrência médica indesejada ao pesquisador e/ou a sua equipe. Quaisquer eventos adversos que ocorrerem após o tratamento do estudo, ou seja, período no qual o participante não esteja sob supervisão médica direta, deverão ser imediatamente comunicados à equipe do centro de ensaios clínicos (por telefone, por exemplo).

8.2.2 Procedimento pós-estudo

Por ocasião da alta do último dia de hospitalização, todos os participantes serão novamente orientados participar de qualquer estudo clínico com medicamentos por 12 meses.

Todos os participantes, inclusive os que desistirem da participação no estudo após a administração de pelo menos uma dose do produto sob investigação, deverão ser reavaliados clinicamente (incluindo aferição dos sinais vitais, realização de exame físico e se necessário exames laboratoriais). Independentemente de alteração, todos os resultados obtidos deverão ser relatados nos protnuários individuais dos respectivos participantes do estudo.

A negativa ou o não comparecimento do participante para a realização destes procedimentos deverá ser devidamente documentada.

8.3. PROCEDIMENTOS PARA OBTER RELATOS, REGISTRAR E NOTIFICAR EVENTOS ADVERSOS E DOENÇAS INTERCORRENTES

O pesquisador responsável e/ou sua equipe farão perguntas aos participantes para identificar a ocorrência de possíveis eventos adversos. As perguntas realizadas para saber se o participante teve algum evento adverso deverão ser limitadas a perguntas gerais, tais como: "Como você está se sentindo?”

Será solicitado, aos participantes, que relatem qualquer evento adverso e quando isto ocorreu. Será também solicitado que notifique ao pesquisador e/ou a sua equipe, se foi necessário usar medicação adicional.

Qualquer evento adverso deverá ser registrado em detalhes.

No caso de evento(s) adverso(s) grave(s), estes deverão também ser reportados ao patrocinador, através do Formulário de Relato de Evento Adverso Grave. Os detalhes deverão incluir a descrição do evento, utilizando terminologia médica precisa, informação sobre o momento em que começou, sua duração, intensidade, relação causal e as ações médicas tomadas para tratar/resolver o evento adverso, bem como informações sobre o desfecho e qualquer outro dado adicional que, a juízo do pesquisador, seja relevante.

Deverão ser empreendidos todos os esforços pelo pesquisador para explicar cada evento adverso e avaliar sua relação com o produto sob investigação, se houver.

O pesquisador é responsável por reportar todos os eventos adversos graves que ocorrerem durante o estudo em até 24 horas, contadas do momento de conhecimento do evento adverso, para o patrocinador e comunicar oportunamente estes eventos ao Comitê de Ética em Pesquisa (CEP).

8.4. ACOMPANHAMENTO DOS PARTICIPANTES APÓS OCORRÊNCIA DE EVENTOS ADVERSOS

É de responsabilidade do pesquisador garantir que os participantes envolvidos no estudo recebam tratamento adequado para qualquer evento adverso, se necessário. Os eventos deverão ser acompanhados clinicamente e por exames laboratoriais (quando indicados) até que os parâmetros voltem ao normal, em caso de alterações. Este acompanhamento poderá permanecer mesmo após o estudo ter sido completado.

A equipe da Unidade Clínica do centro de ensaios clínicos deverá monitorar a segurança dos participantes, desde a ocorrência de um evento adverso até a sua resolução/recuperação satisfatória. Desta forma, poderão ser necessárias visitas adicionais às inicialmente previstas e realização de outros testes laboratoriais (quando apropriado), mesmo depois que o estudo tenha sido completado, ou seja, após a alta da unidade clínica.

9. GERENCIAMENTO DE DADOS CLÍNICOS

Os métodos serão descritos no Plano de Gestão de Dados (DMP), Plano de Validação de dados (DVP).

9.1 Manuseio e documentação dos dados clínicos

O investigador principal deve garantir que todos os dados necessários de acordo com este protocolo serão inseridos imediatamente nos formulários de dados (prontuário) e, em seguida, no eCRF.

Durante o ensaio clínico cada dado será verificado por uma pessoa independente da equipe de estudo. Campos para os quais não houver dados disponíveis (ausente ou desconhecido) serão riscados. Para efeitos de controle de qualidade, um membro da equipe de estudo irá verificar os dados constantes nos documentos fonte quanto a exatidão formal, completitude e legibilidade dos dados.

Em seguida, os dados serão transferidos para formulários CRF eletrônico e será verificado por um membro da equipe de estudo quanto a exatidão formal e completitude.

As entradas de dados nos documentos fonte deve ser feitas com uma caneta esferográfica de preferência preta e devem ser legíveis. Lápis e fluidos de correcção não serão utilizados. Caso sejam necessárias correcções, elas serão introduzidas por um membro da equipe de estudo da seguinte forma: a entrada errada será riscada; no entanto, deve permanecer legível, e a entrada correta será colocada no campo de correção (se aplicável) ou ao lado da entrada errada. Alterações e correções dos dados fonte serão rubricadas e datadas. Para mudanças e correções deve ser fornecida uma razão, salvo em caso de correcção auto-explicativa.

9.2 GESTÃO E VALIDAÇÃO DOS DADOS

Após o preenchimento dos formulários de relato de caso, os dados serão inseridos no banco de dados do ensaio clínico, gerenciados por um sistema de gerenciamento de dados, empregando-se procedimento de dupla entrada de dados ou outro método de validação, de acordo com o que for especificado, para cada caso, no Plano de Validação de Dados do ensaio clínico. O banco de dados será preparado para entrada de dados e refletirá a versão final dos formulários de relato de caso. Serão aplicados métodos de controle de qualidade adequados, conforme especificado no plano de gerenciamento de dados do ensaio clínico. A verificação de integridade, exatidão eplausibilidade (através de verificação cruzada) será executada conforme definido no Plano de Validação de Dados específico do ensaio clínico.

As discrepâncias que forem identificadas e não permitirem a aplicação de procedimentos auto evidentes de correção, serão encaminhadas pelo responsável pelo gerenciamento de dados para questionamento específico. De acordo com o que for estipulado no Plano de Gerenciamento de Dados, os questionamentos e respectivas respostas serão efetuados utilizando formulários de esclarecimento de dados (Data Clarification Form - DCF), a serem encaminhados ao

10. CONTROLE E GARANTIA DA QUALIDADE

O controle de qualidade inclui todas as atividades realizadas antes, durante e após a realização do estudo, com o objetivo de verificar e controlar a qualidade. As atividades e técnicas empregadas estarão de acordo com os procedimentos (POPs) aplicáveis, cabendo a cada uma a responsabilidade final pela qualidade de suas atividades.

11. MONITORIA

O acompanhamento do ensaio clínico será realizado pelo patrocinador, de acordo com o Guia de Boas Práticas Clínicas do ICH (ICH – E6 – R2) e com base no plano de monitoria.

Um monitor de ensaios clínicos, irá monitorar o progresso doestudo clínico, a fim de assegurar que o estudo esteja sendo conduzido, registrado e reportado de acordo com o protocolo, procedimentos operacionais padrão, boas práticas clínicas e regulamentações aplicáveis.

12. GARANTIA DE QUALIDADE

A garantia de qualidade inclui todas as atividades desenvolvidas durante e depois de um ensaio clínico para verificar e controlar sua qualidade. Ela abrange controle de qualidade interno pela própria equipe do centro de ensaios clínicos, bem como pelo patrocinador através da realização de monitoramento e atividades de auditoria.

O investigador deverá permitir a verificação da condução do ensaio clínico por parte do CEP ou inspeções regulatórias (com acesso direto aos dados fonte).

Nos casos de visitas de monitoria ou auditorias, o Investigador Principal deverá conceder ao representante do patrocinador o acesso a todos os documentos e dados fonte relacionados ao ensaio clínico, cabendo também ao patrocinador assegurar a onfidencialidade dos participantes do ensaio.

13. MANUTENÇÃO DOS REGISTROS

Todos os documentos provenientes do ensaio clínico deverão ser mantidos no arquivo do Investigador por pelo menos cinco anos após a finalização do relatório do ensaio clínico integrado, ou, de acordo com o ICH, por pelo menos dois anos após a última aprovação em uma região ICH, ou pelo menos dois anos após a interrupção formal do desenvolvimento clínico, o que for mais longo.

14. DOCUMENTOS FONTE E ACESSO AOS DADOS FONTE

Os investigadores e as instituições envolvidas se comprometem a permitir a realização de atividades de monitoria, auditorias oficiais, revisão por parte do Comitê de Ética em Pesquisa e inspeção pelos órgãos governamentais envolvidos, proporcionando, caso seja necessário, o acesso aos dados brutos, única e exclusivamente para os fins relacionados ao acompanhamento do ensaio clínico.

Não obstante, este direito de acesso implica a anuência dos envolvidos quanto aos aspectos de confidencialidade dos dados, mantendo-se o sigilo quanto à identidade dos participantes.

15. Política de Publicação

A fim de cumprir as obrigações legais e éticas, a publicação dos resultados deste ensaio clínico será favorecida e é intenção publicar os dados, independentemente se forem obtidos resultados negativos ou positivos. Afiliações institucionais e qualquer possível conflito de interesses devem ser declarados.

16. ÉTICA

16.1. COMITÊ DE ÉTICA EM PESQUISA

O projeto de pesquisa, contendo o protocolo de ensaio clínico e o termo de consentimento livre e esclarecido, deverá ser submetido ao Comitê de Ética em Pesquisa com Seres Humanos do Instituto de Ciências Biomédicas da USP, credenciado pela CONEP -Comissão Nacional de Ética em Pesquisa/CNS/MS, para análise e aprovação ética.

O ensaio não será iniciado antes que seja emitido um parecer de aprovação por escrito pelo Comitê de Ética em Pesquisa. O pesquisador será responsável por obter aprovação do estudo pelo Comitê de Ética. Uma cópia do parecer de aprovação do ensaio clínico deverá ser fornecida ao patrocinador, antes do início do estudo.

Qualquer emenda ao protocolo do ensaio clínico deverá ser submetida para aprovação do CEP. As emendas só poderão ser implementadas após a aprovação do CEP, o que poderá implicar a interrupção temporária do estudo. Nos casos em que a modificação for necessária para resguardar a segurança e eliminar riscos iminentes aos participantes da pesquisa, a modificação poderá ser implementada sem que o parecer de aprovação do comitê de ética em pesquisa tenha sido disponibilizado. A modificação de aspectos puramente administrativos da pesquisa (como por exemplo mudança de número de telefone) não requer aprovação prévia do comitê de ética em pesquisa para sua implementação.

Deverá ser fornecida ao patrocinador uma cópia da carta de aprovação, quando aplicável.

16.2. TERMO DE CONSENTIMENTO LIVRE E ESCLARECIDO

Antes do início do estudo, o pesquisador responsável deverá obter a aprovação, por escrito, do TCLE e de qualquer outra informação escrita a ser fornecida aos participantes, junto ao CEP. Antes de consentir sua participação, os participantes receberão uma explanação completa e detalhada da natureza e dos objetivos do estudo. Será enfatizado que o estudo tem a finalidade de pesquisa, e que o participante não poderá esperar que haja qualquer efeito terapêutico. O participante também será informado que sua participação no estudo é voluntária e que ele poderá se retirar a qualquer momento do estudo, sem ser obrigado a fornecer o motivo de fazê-lo e sem que isto cause qualquer prejuízo no seu atendimento junto ao centro de pesquisa.

Os participantes terão tempo suficiente e oportunidade de perguntar detalhes do estudo. Após o esclarecimento de todas as dúvidas, caso aceite participar do estudo, o participante, juntamente com o pesquisador responsável (ou membro qualificado por ele designado), deverá assinar e datar o Termo de Consentimento Livre e Esclarecido, em duas vias, antes da realização de qualquer procedimento do estudo. Uma das vias deverá ser entregue ao participante e a outra deverá ser armazenada no centro de ensaios clínicos.

Os participantes deverão ser informados, em tempo hábil, se uma nova informação que possa ser relevante para a decisão de continuar participando do estudo seja disponibilizada, por exemplo: se uma emenda ao protocolo alterar substancialmente o desenho do estudo ou o risco a que os participantes estão submetidos. Neste caso, o TCLE e qualquer outra informação escrita a ser fornecida aos participantes deverão ser revisados, bem como submetidos ao CEP para análise e aprovação ética e os participantes deverão assinar um novo consentimento relativo à participação no estudo.

16.3 CONFIDENCIALIDADE

Os resultados dos exames médicos e dos testes laboratoriais serão registrados no prontuário de cada participante.

Toda a informação obtida durante o estudo referente ao estado de saúde do participante estará disponível à equipe do centro de ensaios clínicos, bem como aos representantes do patrocinador, membros do CEP e às autoridades regulatórias.

O centro de ensaios clínicos, o CEP e as autoridades regulatórias deverão estabelecer procedimentos que assegurem a confidencialidade, a privacidade, a proteção da imagem e a não estigmatização dos participantes da pesquisa, garantindo

a não utilização das suas informações em prejuízo das pessoas e/ou das comunidades, inclusive em termos de autoestima, de prestígio e/ou de aspectos econômico-financeiros.

Uma cópia dos exames laboratoriais realizados durante o estudo será fornecida aos participantes, se assim o desejarem.

17. FINANCIAMENTO E SEGURO

17.1. SEGURO DE VIDA EM GRUPO

Os participantes terão direito a indenização caso venham a sofrer algum dano com a pesquisa.

17.2. FINANCIAMENTO

Os acordos financeiros relativos ao Estudo estarão disponíveis, sendo mantidos em separado dos demais documentos do Estudo.

18. DESVIOS DE PROTOCOLO

O pesquisador responsável deve assegurar que o centro de ensaios clínicos conduza o estudo em conformidade com o protocolo, que deve ser seguido na íntegra. O centro de ensaios clínicos deve implementar procedimentos para que não haja desvios do protocolo. Qualquer não conformidade ao protocolo do estudo deverá ser considerada como desvio de protocolo. Cabe ao pesquisador responsável documentar e providenciar as devidas justificativas para cada desvio de protocolo ocorrido. Todos os desvios de protocolo deverão ser relatados ao patrocinador e notificados ao CEP. Qualquer desvio deste protocolo, e sua respectiva justificativa, deverão ser apresentados no relatório final do estudo.

19. Referências

1. Mulloy B, Hogwood J, Gray E, Lever R, Page CP. Pharmacology of heparin and related drugs. Pharmacol Rev 2016;68:76-141.
2. McIntire A, Harris S, J Whitten, et al. Outcomes following the use of nebulised heparin after inhalation injury (HIHI study). J Burn Care Res 2017;38: 45-52.
3. Shute JK, Calzetta L, Cardaci V, di Toro S, Page CP, Cazzola M. Inhaled nebulised unfractionated heparin improves lung function in moderate to very severe COPD: A pilot study. Pulm Pharmacol Ther. 2018; 48:88-96.
4. Zhang Y, Cao W, Xiao M, et al. [Clinical and coagulation characteristics of 7 patients with critical COVID-2019 pneumonia and acro-ischemia]. Zhonghua Xue Ye Xue Za Zhi. 2020 Mar 28;41(0):E006. doi: 10.3760/cma.j.issn.0253-2727.2020.0006. [Epub ahead of print] Chinese.
5. Tang N, Bai H, Chen X, Gong J, Li D, Sun Z.Anticoagulant treatment is associated with decreased mortality in severe coronavirus disease 2019 patients with coagulopathy Thromb Haemost. 2020 Mar 27. doi: 10.1111/jth.14817. [Epub ahead of print]. PMID:32220112.
6. Li T, Lu H, Zhang W. Clinical observation and management of COVID-19 patients. Emerg Microbes Infect. 2020 Dec;9(1):687-690. doi: 10.1080/22221751.2020.1741327. PMID:32208840.
7. Chimeri L et al, Nebulized Heparin Attenuates Pulmonary Coagulopathy and Inflammation Through Alveolar Macrophages in a Rat Model of Acute Lung Injury. Throm Haem 2017; 117: 2125-2134.
8. Glas GJ, Serpa Neto A, Horn J, et al. Nebulized heparin for patients under mechanical ventilation: an individual patient data meta-analysis. Ann Intensive Care. 2016;6(1):33.
9. Dixon B, Schultz MJ, Smith R, Fink JB, Santamaria JD, Campbell DJ. Nebulized heparin is associated with fewer days of mechanical ventilation in critically ill patients: a randomized controlled trial. Crit Care. 2010;14(5):R180.
10. Ashoor TM, Hasseb AM, Esmat IM. Nebulized heparin and salbutamol versus Salbutamol alone in acute exacerbation of chronic obstructive pulmonary disease requiring mechanical ventilation: a double blind randomised controlled trial. Korean J Anesthesiol. 2020 Feb 28. doi: 10.4097/kja.19418. [Epub ahead of print]
11. Mycroft-West C, Su D, Elli S, Guimond S, et al. The 2019 coronavirus (SARS-CoV-2) surface protein (Spike) S1 Receptor Binding Domain undergoes conformational change upon heparin binding. BioRxiv 2020.
12. Hoffmann M, Kleine-Weber H, Schroeder S, et al. SARS-CoV-2 Cell Entry Depends on ACE2 and TMPRSS2 and Is Blocked by a Clinically Proven Protease Inhibitor. Cell. 2020 Mar 4. [Epub ahead of print].
13. Hamming I, Timens W, Bulthuis ML, Lely AT, Navis G, van Goor H. Tissue distribution of ACE2 protein, the functional receptor for SARS coronavirus. A first step in understanding SARS pathogenesis. J Pathol. 2004 Jun;203(2):631-7.
14. Cagno V, Tseligka ED, Jones ST, Tapparel C. Heparan Sulfate Proteoglycans and Viral Attachment: True Receptors or Adaptation Bias? Viruses. 2019 Jul 1;11(7).
15. Chen C, Gao G, Xu Y,et al. SARS-CoV-2-Positive Sputum and Feces After Conversion of Pharyngeal Samples in Patients With COVID-19. Ann Intern Med. 2020 Mar 30. [Epub ahead of print].
16. Jacques LB, Mahadoo J, Kavanagh LW. Intrapulmonary heparin. Lancet 1976; 308: 1157-1161.

17. Williams PD, Tyrrell DJ, Storm NE, Holme KR. Use of a precise intratracheal delivery system to compare the acute tolerance of heparin and the heparinoid GM2000 in rabbits. Toxicology Mechanisms and Methods 1997; 7: 1-7.
18. Monagle K, Ryan A, Hepponstall M, et al. Inhalational use of antithrombotics in humans: Review of the literature. Thrombosis Res 2015;136:1059-1066.
19. Markart P, Nass R, Ruppert C, et al. Safety and tolerability of inhaled heparin in idiopathic pulmonary fibrosis. J Aerosol Med Pulm Drug Deliv. 2010;23:161-72.
20. Serisier DJ, Shute JK, Hockey PM, Higgins B, Conway J, Carroll MP. Inhaled heparin in cystic fibrosis. Eur Respir J. 2006;27:354-8.
21. Shute JK, Puxeddu E, Calzetta L. Therapeutic use of heparin and derivatives beyond anticoagulation in patients with bronchial asthma or COPD. Curr Opin Pharmacol. 2018; Jun;40:39-45. doi: 10.1016/j.coph.2018.01.006. Epub 2018 Feb 20. Review.

20. ANEXOS

Anexo I - Exames de laboratório de patologia clínica

Anexo II - Lista de Randomização

Anexo II - Formulário de eventos adversos

Anexo IV - *Curriculum vitae* do Pesquisador Principal

Anexo V - Brochura do Investigador

ANEXO I

EXAMES DE LABORATÓRIO DE PATOLOGIA CLÍNICA

FASE	EXAME
**Seleção**	HEMOGRAMA COMPLETO
	DÍMERO D
	FIBRINOGÊNIO
	TEMPO DE TROMBOPLASTINA PARCIAL ATIVADO
	TEMPO DE PROTROMBINA
	POTÁSSIO
	SÓDIO
	CÁLCIO
	MAGNÉSIO
	FÓSFORO INORGÂNICO
	FOSFATASE ALCALINA
	BICARBONATO
	CREATINOQUINASE
	GLICEMIA EM JEJUM
	BILIRRUBINA TOTAL E FRAÇÕES
	TRANSAMINASE GLUTÂMICO OXALACÉTICA (GOT/AST)
	TRANSAMINASE GLUTÂMICO PIRÚVICA (GPT/ALT)
	GAMA GT
	PROTEÍNA C REATIVA ULTRA-SENSÍVEL
	FERRITINA
	TRIGLICÉRIDES
	DESIDROGENASE LÁCTICA
	TROPONINA CARDÍACA -T
	β-HCG para mulheres
**Exames realizados Dias: 1, 3, 5, 8, 11 (todos ± 1 dia) enquanto hospitalizado**	HEMOGRAMA COMPLETO
	DÍMERO D
	FIBRINOGÊNIO
	TEMPO DE TROMBOPLASTINA PARCIAL ATIVADO
	TEMPO DE PROTROMBINA
	POTÁSSIO
	SÓDIO
	CÁLCIO
	MAGNÉSIO
	FÓSFORO INORGÂNICO
	FOSFATASE ALCALINA
	BICARBONATO
	CREATINOQUINASE
	GLICEMIA EM JEJUM
	BILIRRUBINA TOTAL E FRAÇÕES
	TRANSAMINASE GLUTÂMICO OXALACÉTICA (GOT/AST)
	TRANSAMINASE GLUTÂMICO PIRÚVICA (GPT/ALT)
	GAMA GT
	PROTEÍNA C REATIVA ULTRA-SENSÍVEL
	FERRITINA
	TRIGLICÉRIDES
	DESIDROGENASE LÁCTICA
	TROPONINA CARDÍACA -T

ANEXO II

TABELA DE ALEATORIZAÇÃO

1. T
2. C
3. T
4. C
5. C
6. T
7. C
8. T
9. C
10. T
11. T
12. C
13. T
14. T
15. T
16. C
17. C
18. C
19. T
20. C
21. T
22. T
23. C
24. C
25. T
26. C
27. C
28. C
29. C
30. C
31. T
32. C
33. T
34. C
35. T
36. C
37. C
38. T
39. C
40. C
41. T
42. T
43. T
44. T
45. C

46. C____
47. T____
48. T____
49. T____
50. T____
51. T____
52. T____
53. C____
54. T____
55. T____
56. T____
57. C____
58. C____
59. C____
60. T____
61. C____
62. T____
63. T____
64. T____
65. C____
66. T____
67. C____
68. T____
69. T____
70. C____
71. C____
72. T____
73. C____
74. C____
75. C____
76. C____
77. C____
78. C____
79. T____
80. T____
81. T____
82. T____
83. T____
84. T____
85. T____
86. T____
87. T____
88. T____
89. C____
90. C____
91. C____
92. C____
93. T____
94. C____
95. C____
96. C____

97. C____
98. T____
99. C____
100. C____

100 subjects randomized into blocks of
50 50
To reproduce this plan, use the seed 24080
along with the number of subjects per block/number of blocks
and (case-sensitive) treatment labels as entered originally.
Randomization plan created on 28/09/2020 09:07:24

A Randomization Plan
from
http://www.randomization.com

ANEXO III

Código do Estudo	Documento Fonte de Dados Clínicos	Nº Participante	GALENO RESEARCH UNIT
	Ensaio Clínico		

FOLHA DE EVENTO ADVERSO

Código de Rastreamento **GRU_EA_0001**

Qualquer ocorrência médica adversa, doença, sinal ou sintoma desfavorável e não intencional (incluindo achados laboratoriais anormais), após a administração do PSI, quer seja ou não relacionada com o PSI.

Caso se trate de nova versão de um evento já relatado (Follow-Up), anote abaixo o código de rastreamento original ↴

Detalhes Evento	Código de Rastreamento Original: ____________		
Início do EA*	Data	Hora	☐ Atendido na Instituição ☐ Atendimento Externo ☐ Relato do Participante
Queixa / Diagnóstico			(Código)

* Ocorrências antes da administração do Produto sob Investigação no 1º Período não são Eventos Adversos

Classificações				
Gravidade	☐ Não Grave	☐ Grave	★ Nº Identificação do EA Grave:	
Intensidade	☐ Fraco	☐ Moderado	☐ Severo	☐ Letal
Previsibilidade	☐ Esperado	☐ Inesperado	(Natureza ou gravidade não consistente com as informações sobre o produto)	
Causalidade	☐ Definida/Comprovada<br>☐ Improvável/Duvidosa	☐ Provável<br>☐ Condicional	☐ Possível<br>☐ Não avaliável	

Conduta Imediata*	* Conduta tomada logo após a ocorrência do evento quer se trate de atendimento na Galeno, Externo ou simples Relato				
Ação quanto ao PSI	☐ Nenhuma ação	☐ Suspensão	☐ Interrupção temporária	☐ N/A	
☐ Tratamento Farmacológico	☐ Nenhuma / Observação		☐ Outros	☐ Retirada do Estudo	☐ N/A
Detalhes:				Rubrica e data	

Dados Complementares	A ser preenchido somente se houver informação específica
Evolução: (Data) / (Hora)	
Evolução: (Data) / (Hora)	
Desligamento	☐ O médico retirou o Participante do Estudo em função do Evento<br>☐ O Participante resolveu interromper sua participação no estudo em função do Evento
Outras Informações	

Desfecho	☐ Preenchido em conjunto com/ a avaliação acima		
Término	Data	Hora	Não Encerrado (Ver abaixo)
Desfecho (escolha apenas 1 opção)	☐ Resolvido<br>☐ Não resolvido	☐ Resolvido com sequelas<br>☐ Óbito	☐ Ainda não resolvido<br>☐ Desconhecido*
Outras Informações			

* (Participante não compareceu ao agendamento e/ou não deu retorno a telefonemas, etc.) ★ Fornecer detalhes em "Outras Informações"

Médico Responsável pelas Informações / Conclusão   Assinatura   Data

NDN = Nada Digno de Nota | N/A = Não se Aplica | n.d. = Não Disponível | n.r. = Não Requerido neste Protocolo | n.c.s. = Não Clinicamente Significativo

Origem: POP_PEC_pro 22
FORM_PEC_pro_22.1v01
elaborado: 01/10/19

Galeno Desenvolvimento de Pesquisas Ltda. – Campinas – SP – Brasil

EA

ANEXO IV

Curriculum Vitae do Pesquisador Principal

Professor MD Phd Gilberto De Nucci

1981 – Graduação pela Faculdade de Medicina de Ribeirão Preto, Universidade de São Paulo (USP).

1986 – PhD em Farmacologia pelo Royal College of Surgeons of England, Universidade de Londres, Inglaterra.

1989 – Livre Docente, Faculdade de Ciências Médicas, Universidade de Campinas (UNICAMP).

1995 – Professor Adjunto, Departamento de Farmacologia, Faculdade de Ciências Médicas (UNICAMP).

1996 – Professor Titular, Departamento de Farmacologia, Instituto de Ciências Biomédicas (USP).

ANEXO V – Brochura do Investigador

Inhaled Unfractionated Heparin as a Treatment of Patients Infected with COVID-19 and the Complications Arising from this Virus

INVESTIGATOR'S BROCHURE

Document version number: 1.0

Release date: 11 May 2020

Data cut-off date: 07 April 2020

Acknowledgment

The information in the following document is provided to you as an investigator, potential investigator or consultant for review by you, your staff and applicable Institutional Review/Ethics Committee. It has been prepared in a large part by Professor Janis Shute, University of Portsmouth and Ockham Biotech Ltd, with further updates from Professor Clive Page, King's College London.

Signature

Investigator's Brochure: Inhaled Unfractionated Heparin, Version1.0, 11 May 2020

S.D.Singh 11 MAY 2020

Professor Dave Singh MD, FERS, FBPhS
Professor of Clinical Pharmacology and Respiratory
Medicine & Honorary Respiratory Consultant

Date

TABLE OF CONTENTS

SIGNATURE 2

TABLE OF CONTENTS 3

LIST OF TABLES 5

LIST OF FIGURES 6

GLOSSARY OF ABBREVIATIONS 7

2.0 SUMMARY 8
- **2.1 Nonclinical Pharmacology** 9
- **2.2 Nonclinical Toxicology** 9
- **2.3 Clinical Experience** 10
- **2.4 References** 10

3.0 INTRODUCTION 12
- **3.1 Background of Indication Sought** 12
- **3.2 Current Pharmacological Therapy** 12
- **3.3 Inhaled Heparin** 12
- **3.4 References** 13

4.0 PHYSICAL, CHEMICAL AND PHARMACEUTICAL PROPERTIES AND FORMULATION 15
- **4.1 Chemical and Structural Formulae** 15
- **4.2 Physical Description:** 15
- **4.3 Brief Summary of Relevant Physical, Chemical and Pharmaceutical Properties** 16
- **4.4 Description of the Formulation** 17
- **4.5 Instructions for the Storage and Handling of the Dose Form** 17
- **4.6 Structural Similarities to Known Compounds** 17
- **4.7 Aerosol Drug Delivery** 17
- **4.8 References** 18

5.0 NONCLINICAL STUDIES 19
- **5.1 Non-Clinical Pharmacology** 19
- **5.2 Mechanism of Action Determined from in vitro Studies.** 19
- **5.3 Pharmacological effects and mechanism of action** 20
- **5.4 Safety pharmacology** 22
  - 5.4.1 RESPIRATORY SAFETY PHARMACOLOGY 22
  - 5.4.2 CARDIOVASCULAR SAFETY PHARMACOLOGY 22
  - 5.4.3 CENTRAL NERVOUS SYSTEM EFFECTS 24
- **5.5 Pharmacokinetics and Product Metabolism in Animals** 24
- **5.6 Analytical methodology** 24
- **5.7 References** 24

6.0 EFFECTS IN HUMANS 26
6.1 Pharmacokinetics and Product Metabolism in Humans 26
6.2 Safety and Efficacy 26
6.3 Marketing Experience 27
6.4 Rational for dose selection 27
6.5 References 27

7.0 SUMMARY OF DATA AND GUIDANCE FOR THE INVESTIGATOR 34
7.1 Indications and Subject Exposure 34
7.2 Dosage and Method of Administration 34
7.3 Possible Adverse Effects 34
7.4 Contraindications 36
7.5 Specific Guidance for Handling 36
7.6 Precautions 36
7.7 References 36

LIST OF TABLES

Table 1	Heparin Nomenclature	16
Table 2	Pharmacological Effects and Mechanism of Action of Inhaled Heparin	21
Table 3	Acute Animal Toxicity for Heparin Sodium	23
Table 4	Chronic Animal Toxicity	23
Table 5	Description of Studies Reporting Inhaled Heparin Use as an Anticoagulant in Adult Patients with Smoke Inhalation and Acute Lung Injury	28
Table 6	Description of Studies Reporting Effects of Inhaled Unfractionated Heparin in Adult Patients with Asthma or Allergy	29
Table 7	Studies of Nebulised Unfractionated Heparin in Adult Patients with Cystic Fibrosis	32
Table 8	Studies in Patients with Chronic Obstructive Pulmonary Disease	33

LIST OF FIGURES

Figure 1 Structures in a Heparin Octasaccharide 15
Figure 2 The Heterogeneous Nature of Unfractionated Heparin Polysaccharide Chains, Comprising Repeating Disaccharide Units with Varying Sulphation Patterns 16

GLOSSARY OF ABBREVIATIONS

AT	Antithrombin
ATC	Anatomical Therapeutic Chemical
CF	Cystic fibrosis
cGMP	Current Good Manufacturing Practice
COPD	Chronic obstructive pulmonary disease
EC-SOD	Extracellular superoxide dismutase
ECP	Eosinophil cationic protein
FDA	Food and Drug Administration
FEV_1	Forced expiratory volume in one second
FVC	Forced vital capacity
GAG	Glycosaminoglycan
IPF	Idiopathic pulmonary fibrosis
IL-8	Interleukin-8
IV	Intravenous
MBP	Major basic protein
NF	Nuclear factor
PK	Pharmacokinetic(s)
SARS-CoV-2	Severe acute respiratory syndrome coronavirus 2
SC	Subcutaneous
UFH	Unfractionated heparin

2.0 SUMMARY

**Indication:**	Complications arising from COVID-19 infections.
**Investigational Product Description and Pharmacology:**	Inhaled unfractionated heparin (UFH) has a range of pharmacological properties in addition to the well-established anticoagulant activity of this drug. It will bind and reduce the activity of many cytokines implicated in "cytokine storms" [1]. Heparin has many anti-inflammatory, mucolytic and anti-oxidant activities when inhaled directly into the airways [1,2]. Heparin has also been demonstrated to reduce acute lung injury experimentally [3], mortality in patients with COVID-19 [4] and to reduce the number of days requiring ventilation in critical care patients [5,6].
**Non-clinical Toxicology:**	Over the last 100 years, since the discovery of heparin in 1916, there have been many toxicological studies of inhaled heparin in rodents and other small animals indicating the safety of this approach. Lung tissue from dogs that received intrapulmonary heparin (10 to 15 mg/kg by intra-tracheal instillation) for a year showed no signs of haemorrhage, anaemia or ulcerative lesions [7]. Williams et al. [8] showed that when heparin was instilled intra-tracheally in rabbits using a micro-spray device every 48 hours in escalating doses of 0.2, 2, 20, 100, and 200 mg/kg signs of bleeding were seen only at the top two doses.
**Pharmacokinetics:**	Inhaled heparin does not readily cross the bronchial mucosa. In dogs, mice, rats and man, doses of intrapulmonary heparin >8 mg/kg are required for detectable intravenous anticoagulation [7,8]. More recent studies confirmed that at 8 mg/kg, instillation of liquid heparin into the lungs of rabbits resulted in low bioavailability for systemic anticoagulation. Therapeutic systemic anticoagulation was only achieved using dry particle heparin [9].
**Clinical Pharmacokinetics:**	Studies of the lung deposition and clearance of ^{99m}Tc-heparin in healthy volunteers showed that only 8% of a jet nebuliser dose reaches the lower respiratory tract, and of that 39% remained after 24 hours [10]. Additional pharmacokinetic studies in healthy volunteers confirmed the long half-life of intrapulmonary heparin [11]. The slow clearance of intrapulmonary heparin is believed to be due to uptake by pulmonary macrophages and its subsequent slow release.
**Clinical Safety:**	No acute or chronic toxicological effects have been associated with the inhaled route for any dose of UFH in man. In normal human volunteers, 300 to 1500 mg heparin was administered as an aerosol with no sign of toxicity [7]. No incidence of pulmonary haemorrhage associated with inhaled heparin was reported in any study in

536 patients with smoke inhalation injury, acute lung injury, asthma and allergy [12]. Not even pulmonary lavage with intra-tracheal instillation of 200 to 250 mg/kg of heparin to treat alveolar proteinosis induced bleeding into the lungs or was associated with lung damage a year later [7]. Inhaled heparin did not induce allergic reactions in any study, not even when followed up over long periods of up to 485 days. In a study of the effect of inhaled heparin in patients with idiopathic pulmonary fibrosis, 750 mg as the nebuliser fill dose was deemed to be the threshold dose above which effects on systemic coagulation could first be detected [11]. In a compassionate phase of the study, patients continued to use inhaled UFH for up to 100 weeks, without side effects.

Clinical Efficacy: Previous studies [1] have indicated inhaled UFH treats local inflammation, mucus hypersecretion and lung injury, without systemic anticoagulation (20 studies, 536 patients) [12] and is safe and effective in patients with smoke inhalation injury, acute lung injury, asthma and allergy, cystic fibrosis and chronic obstructive pulmonary disease (COPD). In another clinical trial in patients with moderate to severe COPD, approximately 60 mg inhaled UFH delivered to the lung significantly improved lung function, exercise capacity and dyspnoea with no evidence for toxicity or adverse side effects at this dose [13].

2.1 Nonclinical Pharmacology

UFH is a drug with a structurally diverse molecular scaffold naturally oriented to multiple targets, with mucolytic, wound healing, anti-inflammatory and anti-oxidant pharmacological activities when inhaled directly into the airways [1]. These effects are independent of the anticoagulant activity seen when UFH is administered intravenously (IV). Pre-clinical studies show that basic proteins (platelet factor-4, interleukin [IL]-8, lactoferrin, eosinophil cationic protein) present in cystic fibrosis (CF) sputum inhibit the anticoagulation (anti-Xa) activity of heparin, data that underpin the safety of this therapeutic approach [13].

Importantly, UFH significantly reduced the amount of extracellular DNA and the elasticity of CF sputum when added to CF sputum ex vivo at a concentration of 1 mg/mL [2]. Very recently heparin has also been reported to bind the Spike 1 protein used by the COVID-19 virus to enter cells [14].

2.2 Nonclinical Toxicology

Long-term inhalation studies: Lung tissue from dogs that received intrapulmonary heparin (10 to 15 mg/kg by intratracheal instillation) for a year showed no signs of haemorrhage, anaemia or ulcerative lesions [7]. There was no other type of lesion found. Heparin reduced the degree of cellular infiltration compared to untreated dogs.

Short-term inhalation studies: Acute toxicity associated with the inhaled route of delivery for UFH has been tested in dogs, mice, rats and human volunteers. In all species, a single administration produced systemic anticoagulation only at doses >8 mg/kg body weight [7]. When heparin was instilled intratracheally in rabbits using a microspray device every 48 hours in escalating doses of 0.2, 2, 20, 100, and 200 mg/kg signs of bleeding were seen only at the top two doses [8].

Nonclinical Absorption, Distribution Metabolism and Excretion: UFH delivered to the lung as a nebulised solution, or instilled directly intra-tracheally, is not absorbed from the tracheobronchial tree at doses <8 mg/kg, in any species, and doses higher than 8 mg/kg are required for detectable IV anticoagulation by the intrapulmonary route [7,9].

2.3 Clinical Experience

Others have reported no clinically significant effect on systemic coagulation of intrapulmonary UFH delivered from a jet nebuliser with loading doses of UFH up to 2000 mg of which 8% (160 mg) is delivered to the lung, equating to 2.6 mg/kg in an average 60-kg subject, in normal healthy subjects [15].

A clinical study of inhaled heparin in adult CF patients [16]. UFH was delivered from a jet nebuliser loaded with 250 mg UFH solution, twice a day for 14 days. Based on an estimated 8% efficiency of the nebuliser, the lung dose was estimated to be 20 mg. The study demonstrated the safety of this approach, with no effects on systemic coagulation parameters. However, anecdotal reports on improved mucus clearance did not translate into a significant effect on lung function at the doses used in this study [16].

More recently [13], and at an estimated lung dose of 60 mg, a clinical study of inhaled nebulised UFH was conducted in severe to very severe COPD patients with a significant improvement in lung function (forced expiry volume in 1 second [FEV_1]), exercise capacity and dyspnoea being observed over 7 to 21 days, which was maintained for 7 days after the end of treatment.

2.4 References

1. Mulloy B, Hogwood J, Gray E, Lever R, Page CP. Pharmacology of heparin and related drugs. Pharmacol Rev 2016;68:76-141.
2. Broughton-Head VJ, Shur J, Carroll MP, Smith JR, Shute JK. Unfractionated heparin reduces the elasticity of sputum from patients with cystic fibrosis. Am J Physiol Lung Cell Mol Physiol. 2007;293:L1240-9.
3. Chimenti L, Camprubi-Rimblas M, Guillamat-Prats R, Gomez MN, Tijero J, Blanch L, Artigas A. Nebulized heparin attenuates pulmonary coagulopathy and inflammation through alveolar macrophages in a rat model of acute lung injury. Thromb Haemost 2017;117:2125-2134.
4. Tang N, Bai H, Chen X, Gong J, Li D, Sun Z. Anticoagulant treatment is associated with decreased mortality in severe coronavirus disease 2019 patients with coagulopathy. J Thromb Haemost 2020, doi: 10.1111/jth.14817.

5. Ashoor TM, Hasseb AM, Esmat IM. Nebulized heparin and salbutamol versus salbutamol alone in acute exacerbation of chronic pulmonary disease requiring mechanical ventilation: a double blind randomized controlled trial. Korean J Anesthesiol 2020, doi: 10.4097/kja.19418.
6. Dixon B, Schultz MJ, Smith R, Fink JB, Santamaria JD, Campbell DJ. Nebulized heparin is associated with fewer days of mechanical ventilation in critically ill patients: a randomized controlled trial. Crit Care 2010, doi: 1186/cc9286.
7. Jacques LB, Mahadoo J, Kavanagh LW. Intrapulmonary heparin. Lancet 1976; 308:1157-1161.
8. Williams PD, Tyrrell DJ, Storm NE, Holme KR. Use of a precise intratracheal delivery system to compare the acute tolerance of heparin and the heparinoid GM2000 in rabbits. Toxicology Mechanisms and Methods 1997;7:1-7.
9. Qi Y, Zhao G, Liu D, Shriver Z, Sundaram M, Sengupta S, Venkataraman G, Langer R, Sasisekharan R. Delivery of therapeutic levels of heparin and low-molecular-weight heparin through a pulmonary route. Proc Natl Acad Sci U S A. 2004;101:9867-72.
10. Bendstrup KE, Chambers CB, Jensen JI, Newhouse MT. Lung deposition and clearance of inhaled (99m)Tc-heparin in healthy volunteers. Am J Respir Crit Care Med. 1999;160:1653-8.
11. Markart P, Nass R, Ruppert C, Hundack L, Wygrecka M, Korfei M, Boedeker RH,Staehler G, Kroll H, Scheuch G, Seeger W, Guenther A. Safety and tolerability of inhaled heparin in idiopathic pulmonary fibrosis. J Aerosol Med Pulm Drug Deliv. 2010;23:161-72.
12. Monagle K, Ryan A, Hepponstall M, Mertyn E, Monagle P, Ignjatovic V, Newall F. Inhalational use of antithrombotics in humans: Review of the literature. Thrombosis Res 2015;136:1059-1066.
13. Shute JK, Calzetta L, Cardaci V, di Toro S, Page CP, Cazzola M. Inhaled nebulised unfractionated heparin improves lung function in moderate to very severe COPD: A pilot study. Pulm Pharmacol Ther. 2018; 48:88-96.
14. Mycroft-West C, Su D, Eli S, Guimond S, Miller G, Turnball J, yates E, Guerrini M, Fernig D, Lima M, Skidmore M. The 2019 coronavirus (SARS-CoV-2) surface protein (Spike) s1 receptor binding domain undergoes conformational change upon heparin binding. bioRxiv preprint doi: http://doi.org/10.1101/2020.02.29.971093.
15. Bendstrup KE, Gram J, Jensen JI. Effect of inhaled heparin on lung function and coagulation in healthy volunteers. Eur Resp J 2002;19: 606-610.
16. Serisier DJ, Shute JK, Hockey PM, Higgins B, Conway J, Carroll MP. Inhaled heparin in cystic fibrosis. Eur Respir J. 2006;27:354-8.

3.0 INTRODUCTION

Heparin belongs to a family of polyanionic glycosaminoglycans (GAGs), polysaccharides composed of hexuronic acid and D-glucosamine residues joined by glycosidic linkages. Heparin is currently authorised for intravenous (IV) and subcutaneous (SC) use as an anticoagulant (Anatomical Therapeutic Chemical [ATC] BO1AB01) in the BO1A group of anti-thrombotic agents. However, unfractionated heparin (UFH) is a drug with a structurally diverse molecular scaffold naturally oriented to multiple targets, with mucolytic, wound healing, anti-inflammatory and anti-oxidant pharmacological activities that are often unrelated to anticoagulation when inhaled directly into the airways and be considered additional pharmacological properties.

3.1 Background of Indication Sought

Severe acute respiratory syndrome coronavirus 2 (SARS-CoV-2) is a novel coronavirus currently responsible for a global pandemic of COVID-19 disease. COVID-19 causes fever, cough, sputum, shortness of breath, fatigue, myalgia, diarrhoea, nausea and vomiting. In more severe cases, patients deteriorate rapidly to have acute respiratory distress syndrome, metabolic acidosis, septic shock, coagulopathy, and organ failure (particularly liver, kidney, and cardiac failure) leading to death. There are currently no effective medicines, with no vaccines and only symptomatic treatments available. In its more severe form, the disease is associated with "cytokine storms" and these are thought to be the basis of disease exacerbation and death. Interleukin (IL)-6 levels are particularly high in severely ill patients with COVID-19. It is proposed to investigate inhaled UFH to reduce the complications arising from a cytokine storm and to prevent the magnitude of the effects of a cytokine storm in patients with moderate to severe COVID-19 driven disease. Recent data suggest that heparin may also bind the Spike 1 protein that the COVID-19 virus uses to enter to cells, and so heparin may provide an additional anti-viral effect in patients with COVID-19.

3.2 Current Pharmacological Therapy

There is currently no effective therapy for COVID-19 infected patients available and it is largely being managed symptomatically. However, two papers from investigators who have been treating COVID-19 patients in China have reported that systemic heparin administration shows promise in the treatment of this disease. One study demonstrated an improvement in the hypercoaguability in COVID-19 patients, reducing IL-6 levels and blocking the effects of IL-6; in addition, heparin counters some of the lymphocytopenia observed in these patients [1]. A second study has reported a reduction in mortality in COVID-19 patients [2].

3.3 Inhaled Heparin

In addition to the anticoagulant and anti-inflammatory effects of systemically administered heparin described in the two studies from China [1,2], there are other published studies suggesting that inhaled heparin may offer additional benefit to COVID-19 patients, beyond and independent of the anticoagulant effects of this drug. These additional relevant pharmacological properties include:

- Inhaled heparin reduces acute lung injury [3] and binds to and counters the effect of multiple cytokines implicated in "cytokine storms" (eg, IL-6) and many other pro-inflammatory mediators [4].
- Inhaled heparin can reduce the number of ventilator free days in patients with other respiratory crises such as severe chronic obstructive pulmonary disease (COPD) [5] and the respiratory complications arising from smoke inhalation and burns [4,6].
- Inhaled heparin can improve lung function in patients with compromised airways from moderate to severe COPD [7], as well acting as a mucolytic in patients with cystic fibrosis (CF) [8].

Importantly, in all of these studies (and in earlier studies investigating inhaled heparin in patients with asthma [9,10]) there was no effect of inhaled heparin on systemic coagulation parameters. Furthermore, bleeding complications did not occur more frequently with heparin than placebo and, in particular, there were no episodes of haemoptysis while inhaling heparin indicating the safety of this route of heparin administration.

3.4 References

1. Shi C, Wang C, Wang H, Yang C, Cai F, Zeng F, Cheng F, Liu Y, Zhou T, Deng B, Li, Zhang Y. Clinical observations of low molecular weight heparin in relieving inflammation in COVID-19 patients: A retrospective cohort study. medRxiv preprint, doi: https://10.1101.2020.03.28.20046144.
2. Tang N, Bai H, Chen X, Gong J, Li D, Sun Z. Anticoagulant treatment is associated with decreased mortality in severe coronavirus disease 2019 patients with coagulopathy. J Thromb Haemost 2020, doi: 10.1111/jth.14817.
3. Chimenti L, Camprubi-Rimblas M, Guillamat-Prats R, Gomez MN, Tijero J, Blanch L, Artigas A. Nebulized heparin attenuates pulmonary coagulopathy and inflammation through alveolar macrophages in a rat model of acute lung injury. Thromb Haemost 2017;117:2125-2134.
4. Mulloy B, Hogwood J, Gray E, Lever R, Page CP. Pharmacology of heparin and related drugs. Pharmacol Rev 2016;68:76-141.
5. Ashoor TM, Hasseb AM, Esmat IM. Nebulized heparin and salbutamol versus salbutamol alone in acute exacerbation of chronic pulmonary disease requiring mechanical ventilation: a double blind randomised controlled trial. Korean J Anesthesiol 2020, doi: 10.4097/kja.19418.
6. Dixon B, Schultz MJ, Smith R, Fink JB, Santamaria JD, Campbell DJ. Nebulized heparin is associated with fewer days of mechanical ventilation in critically ill patients: a randomized controlled trial. Crit Care 2010, doi: 1186/cc9286.
7. Shute JK, Calzetta L, Cardaci V, di Toro S, Page CP, Cazzola M. Inhaled nebulised unfractionated heparin improves lung function in moderate to very severe COPD: A pilot study. Pulm Pharmacol Ther. 2018;48:88-96.
8. Serisier DJ, Shute JK, Hockey PM, Higgins B, Conway J, Carroll MP. Inhaled heparin in cystic fibrosis. Eur Respir J. 2006;27:354-8.

9. Diamant Z, Timmers MC, van der Veen H, Page CP, van der Meer FJ, Sterk PJ. Effect of inhaled heparin on allergen-induced early and late asthmatic responses in patients with atopic asthma. Am J Respir Crit Care Med. 1996,153: 1790-5.
10. Ahmed T, Garrigo J, Danta I. Preventing bronchoconstriction in exercise-induced asthma with inhaled heparin. N Engl J Med. 1993,329: 90-95.

4.0 PHYSICAL, CHEMICAL AND PHARMACEUTICAL PROPERTIES AND FORMULATION

4.1 Chemical and Structural Formulae

Heparin is a unique molecule, and the most negatively charged polyanionic molecule in biology. The unfractionated heparin used in medicine consists of polysaccharide chains of diverse lengths and therefore molecular weights from 5,000 to 50,000 Da [1]. These chains are comprised of repeating 1 → 4 linked disaccharide units in which one monosaccharide is an α-d-glucosamine residue and the other an uronic acid, with varying degrees of sulphation [2]. Heparin contains a unique pentasaccharide sequence (Figure 1) that binds antithrombin (AT), potentiating its inhibition of activated coagulation factors by approximately 1000-fold, although other interactions contribute to the overall haemostatic effect. Heparin is a highly diverse structure, and only one third of chains contain this sequence.

Figure 1 Structures in a Heparin Octasaccharide

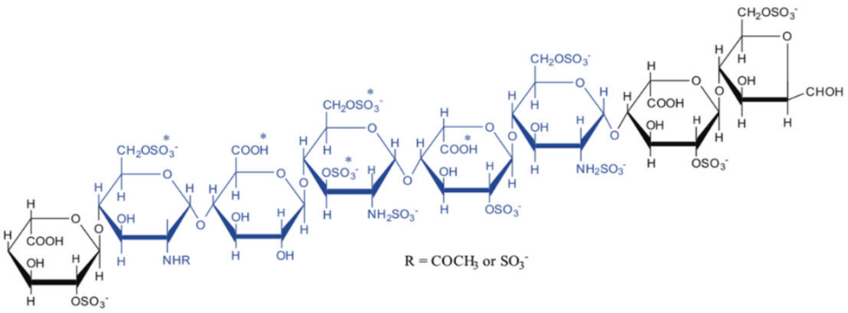

Structures in a heparin octasaccharide with high affinity for antithrombin containing the essential pentasaccharide sequence shown in blue.

4.2 Physical Description:

Molecular Formula: UFH is a polymer of repeating disaccharide units, of which the most common in UFH from pig intestinal mucosa (75%) is composed of a 2-O-sulphated iduronic acid and 6-O-sulphated, N-sulphated glucosamine, IdoA(2S)-GlcNS(6S) (C12H19NO20S3.).

Molecular Weight: UFH has an average molecular weight of 12 to 15 KDa.

Structural Formula: UFH is highly heterogeneous, and only one third of chains contain the specific AT binding sequence as illustrated in Figure 2.

Figure 2 The Heterogeneous Nature of Unfractionated Heparin Polysaccharide Chains, Comprising Repeating Disaccharide Units with Varying Sulphation Patterns

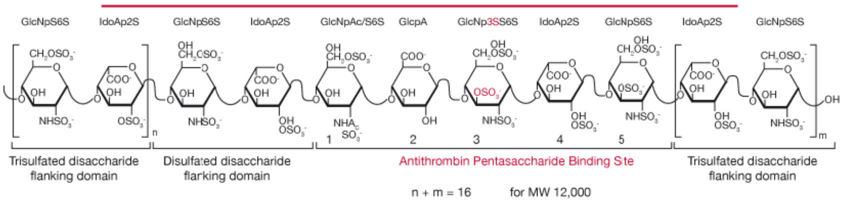

Table 1 Heparin Nomenclature

Chemical Name [IUPAC]	Unfractionated heparin
International Non-Proprietary Name (INN)	TBD
British Approved Name (BAN)	TBD
United States Adopted Name (USAN)	TBD
Laboratory Code	TBD

Abbreviations: IUPAC, International Union of Pure and Applied Chemistry; TBD, to be determined.

4.3 Brief Summary of Relevant Physical, Chemical and Pharmaceutical Properties

Heparin is readily soluble in aqueous solution and is soluble in water at 50 mg/mL. Heparin is a polysaccharide, which has excellent aqueous solution stability (up to 2 years at 2°C to 8°C) provided the solutions have been filtered through a 0.2-µm membrane. It is not intended to add preservatives, such as benzyl alcohol, as these can have negative effects on the respiratory system [3].

In accelerated stability studies, heparin samples prepared in 10-mM sodium phosphate buffer at pH 7.0 in sealed ampules that had been flushed with nitrogen and incubated at 100°C were relatively stable over the first 500 hours, after which it rapidly degraded as acidity of the solution increased [4].

A solution of heparin sodium in saline at the concentrations to be tested has a pH of 6.5 and it is expected that batches of heparin prepared for the clinical trial will be stable for the duration of the study.

4.4 Description of the Formulation

Active substance: UFH sodium from pig intestinal mucosa.

Clinical heparin marketed in the UK is an animal polysaccharide extracted from porcine intestinal mucosa. Unfractionated heparin, however, is a mixture of polysaccharide chains with molecular masses ranging from 5,000 to 50,000 Da, giving it a broad repertoire of positively-charged targets.

Heparin sodium 25,000 IU/mL will be provided in 1-mL ampoules of 25,000 IU/mL without preservative (Monoparin 25,000 IU/mL solution for injection, Wockhardt UK). The product is and is fully compliant with the European Pharmacopoeia Monograph for Heparin Sodium. Heparin sodium 25,000 IU/mL also contains as inactive excipients: water for injections, sodium hydroxide solution 3M, and hydrochloric acid 3M. Heparin sodium will be administered as a nebulised aerosol dose (detailed information in Sections 4.7 and 7.2).

4.5 Instructions for the Storage and Handling of the Dose Form

Vials should be stored at 2°C to 8°C and should NOT be frozen.

Doses will be made up immediately before use as follows: one 1-mL ampoule of Heparin sodium 25,000 IU/mL without preservative will be added to the nebuliser chamber followed by 4.0 mL Sodium Chloride Injection BP 0.9%. The chamber will be assembled and swirled to mix the heparin thoroughly.

4.6 Structural Similarities to Known Compounds

Low molecular weight heparins are derived from UFH by various chemical processes generating heparins with average molecular weights from 3,600 to 6,500 depending on the process [5]. These are not to be substituted for UFH, as they have different pharmacological properties and may be less effective.

4.7 Aerosol Drug Delivery

Lung Deposition Studies

The optimal aerodynamic particle size distribution for lung deposition is considered to be particles with a diameter 1 to 5 µm. Larger particles deposit in the oropharynx and are swallowed, and smaller particles with low mass may not deposit but be exhaled.

Lung deposition of solutions of unfractionated heparin (Wockhardt) in saline (31.25 mg/ml) aerosolised from a volume of 6 ml using the Aeroneb Go (Philips Respironics, UK) vibrating mesh nebuliser was tested using the Next Generation Cascade Impactor (NGI, Copley, UK). Aerosolisation was achieved when UFH was dissolved in salt solutions but not in water. Air was drawn at a flow rate of 15 L/min through a series of seven nozzles with progressively reducing jet diameter. Particles deposit at stages depending on their inertia, a function of both particle size and velocity. Each stage is associated with a cut-off diameter above which particles have sufficient inertia to escape the airflow and be deposited. UFH deposited on

each stage was dissolved in water and the concentration measured by UV absorbance at 206 nm.

In the NGI, delivery to stages 3 (3.3 to 4.7 uM) and 4 (2.1 to 3.3 uM) were on average 20% of the loading dose, this reflects bronchial deposition. However, overall delivery including to stages 3, 4, 5, 6, 7 (i.e., down to particles 0.43 to 0.65 uM) were higher, on average 35 %.

The respirable dose is reduced in constant-output nebulisers such as the AeroNeb Go where drug is lost during exhalation. However, the Aerogen Ultra reduces drug loss on exhalation and is considered to be the optimal nebuliser for delivery of UFH in the clinical setting.

4.8 References

1. Mulloy B, Heath A, Shriver Z, Jameison F, Al Hakim A, Morris TS, Szajek AY. USP compendial methods for analysis of heparin: chromatographic determination of molecular weight distributions for heparin sodium. Anal Bioanal Chem. 2014;406:4815-23.
2. Mulloy B, Hogwood J, Gray E, Lever R, Page CP. Pharmacology of heparin and related drugs. Pharmacol Rev. 2016;68:76-141.
3. CFNP TAP Review Heparin. 2002.
4. Jandik KA. Accelerated stability studies of heparin. J Pharm Sci. 1996;85:45-51.
5. Gray E, Mulloy B, Barrowcliffe TW. Heparin and low-molecular-weight heparin. Thromb Haemost. 2008; 99: 807-18.

5.0 NONCLINICAL STUDIES

5.1 Non-Clinical Pharmacology

Heparin has the highest negative charge of any molecule in biology and multiple protein binding partners [1]. Many of these interactions are charge-dependent although in some cases a specific saccharide protein-binding sequence has been determined (as for AT). As a result, heparin has multiple pharmacological properties beyond anticoagulation [1]. These include inhibition of neutrophil activation and degranulation, inhibition of the steps leading to transendothelial migration of neutrophils, including rolling and adhesion, and inhibition of heparinase activity [1]. The ability of heparin to bind many cytokines involved in inflammatory cell recruitment means that these chemokines are then unable to bind to their respective receptors [1]. This is exemplified by heparin forming a complex with IL-8 that is unable to activate its receptor [2] which in turn would result in reduced chemoattraction of neutrophils into tissues such as the lung following treatment with heparin [2,3].

Furthermore, heparin has direct inhibitory effects on neutrophil elastase and cathepsin G that correlate positively with the saccharide chain length and degree of sulphation [4], as well as inhibiting neutrophil activation and release of elastase [5]. In view of the fact that elastase is a potent mucus secretagogue and tissue elastolytic enzyme there are multiple beneficial effects of reducing the elastase burden in the lungs of COVID-19 patients, which should overall restore mucociliary clearance and the protease/antiprotease balance, while limiting tissue damage. Similar direct effects of heparin have been described against other cationic mediators known to cause tissue damage in the lung such as major basic protein (MBP) and eosinophil cationic protein (ECP) [6,7]. Heparin also has anti-oxidant activity [8] which may further reduce inflammation.

5.2 Mechanism of Action Determined from in vitro Studies.

As an anticoagulant, UFH is widely used clinically as an antithrombotic, delivered either IV or SC, to treat deep vein thrombosis, pulmonary embolism, acute coronary syndromes as well as prevention of thrombosis in extracorporeal circuits and haemodialysis.

However, UFH has further multiple pharmacological activities when inhaled that are unrelated to its anticoagulant activity (reviewed in [1]). These include;

1. Mucolytic:
   - Dissociates DNA/protein aggregates in sputum
   - Activates DNases to degrade extracellular DNA in sputum
   - Reduces electrostatic interaction between mucins
   - Reduces sputum elasticity
2. Anti-viral:
   - Inhibits COVID-19 spike 1 protein to prevent viral entry to cells
3. Anti-inflammatory:

- Inhibition of chemokines including the neutrophil chemoattractants, IL-6 and IL-8
- Inhibition of neutrophil elastase and cathepsin G activity
- Inhibition of complement activation
- Neutralisation of basic/cationic pro-inflammatory proteins such as ECP
- Inhibition of elastase release and neutrophil aggregation
- Inhibition of neutrophil recruitment into tissues in response to a wide variety of stimuli

4. Anti-oxidant:

- Inhibition of free radical release from activated neutrophils
- Increased expression and activity of extracellular superoxide dismutase (EC-SOD)

5.3 Pharmacological effects and mechanism of action

Pharmacological effects and mechanism of action of inhaled heparin determined from in vivo studies in animal models of acute lung injury [9], are summarized in Table 2.

Table 2 Pharmacological Effects and Mechanism of Action of Inhaled Heparin

Drug (dose)	Animal	Lung injury model and nebuliser	Effect parameter, observed effect and safety
Rh-APC (5,000 µg/kg)<br>Heparin (1,000 U/kg)<br>Plasma-derived human AT (500 IU/kg)<br>Danaparoid (250 E/kg)	Rats	*S. pneumoniae* pneumonia<br>Aeroneb Pro[a]	All agents attenuated pulmonary coagulation.<br>Plasma-derived human AT also attenuated pulmonary inflammation, bacterial outgrowth and changes in histopathology.<br>Only danaparoid affected systemic coagulation. Systemic bleeding was not reported.
Rh-APC (5,000 µg/kg)<br>Heparin (1,000 U/kg)<br>Plasma-derived human AT (500 IU/kg)<br>Danaparoid (250 E/kg)	Rats	i.v. LPS<br>Aeroneb Pro[a]	All agents attenuated pulmonary coagulation; pulmonary inflammation and histopathology were not affected.<br>Heparin and danaparoid affected systemic coagulation. Systemic bleeding was not reported.
Heparin (5 µg) i.t.	mice	*Legionella*<br>pneumonia<br>No nebuliser used	Heparin improved survival and decreased pulmonary inflammation, bacterial outgrowth, *Legionella*-adherence and endothelial permeability.<br>Effects on systemic coagulation or systemic bleeding were not reported.
Heparin (10,000 IU/4 h)<br>AT (290 IU)<br>or a combination	sheep	burn and smoke inhalation<br>Nebuliser not stated	Combination therapy improved haemodynamics and P/F ratio; airway obstruction and wet-to-dry weight decreased.<br>Systemic clotting time was unaffected. Systemic bleeding was not reported.
Combination therapy of Heparin (10,000 IU/4 h) and plasma-derived human AT i.v. (0.34 mg/kg/h)	sheep	burn and smoke inhalation<br>Nebuliser not stated	Heparin + plasma-derived human AT improved P/F ratio; central venous pressure, airway obstruction and wet-to-dry weight decreased.<br>Systemic levels of AT were elevated. Systemic bleeding was not reported.
Heparin (10,000 IU/4 h) or Heparin i.v. (5,300 U/kg/23 h)	sheep	burn and smoke inhalation + *P. aeruginosa*-pneumonia<br>Airlife Misty[d]	Nebulization of heparin improved haemodynamics and P/F ratio; airway obstruction, wet-to-dry weight and changes in histopathology were decreased.<br>Systemic clotting time was unaffected with nebulized heparin. Systemic bleeding was not reported.
Heparin (10,000 IU/4h)<br>and/or<br>Lisofylline i.v. (10 mg/kg/h after bolus 20 mg/kg)	sheep	burn and smoke inhalation<br>Nebuliser not stated	Combination therapy decreased the need of mechanical ventilation, P(A-a)O_2 and pulmonary shunt fraction; wet-to-dry weight and changes in histopathology were unaffected.<br>Effects on systemic coagulation and systemic bleeding were not reported.

Abbreviations: AT, antithrombin; EVLW, extravascular lung water; i.v., intravenously; i.t., intratracheally; LPS, lipopolysaccharide; P/F, PaO_2/FiO_2; Rh-APC, recombinant human-activated protein C; VCAM-1, vascular cell adhesion molecule-1.

Note: Drugs were delivered intravenously (i.v.) and intratracheally (i.t.) as described in the table. a) Aerogen, Galway, Ireland. b) Siemens-Elema AB, Solna, Sweden. c) Health Care Worldwide, Somerset, PA, USA. d) Allegiance Healthcare, McGaw Park, IL, USA.

Overall, nebulisation of heparin and intratracheally-installed heparin were found to attenuate pulmonary coagulopathy in these models. Intratracheally-installed heparin reduced pulmonary inflammation and endothelial permeability. In addition, intratracheally-installed heparin improved survival, reduced bacterial outgrowth and bacterial adherence to lung epithelium. In one study in rats, inhaled UFH at 1,000 IU/kg (approximately 5 mg/kg) affected systemic coagulation. However, others have reported no systemic anticoagulation in dogs, mice, rats, and humans at doses < 8 mg/kg body weight [10], and negligible uptake of liquid heparin instilled at 8 mg/kg in the airways of rabbits [11].

More recently, inhaled nebulised heparin (1,000 IU/kg) has been shown to limit pulmonary inflammation, without systemic bleeding, in a rat model of acute lung injury induced by lipopolysaccharide, via downregulation of nuclear factor (NF)-kB activity in pulmonary macrophages [12].

In sheep, inhaled heparin at 1000 IU/kg inhibited allergen-induced bronchoconstriction [13] an effect that was believed to be due to inhibition of mast-cell degranulation.

5.4 Safety pharmacology

5.4.1 RESPIRATORY SAFETY PHARMACOLOGY

Acute toxicity associated with the inhaled route of delivery for UFH has been tested in dogs, mice, rats, and humans. In all species, a single administration produced systemic anticoagulation only at doses >8 mg/kg body weight [10]. Williams et al [14] showed that when heparin was instilled directly into the trachea in rabbits using a micro-spray device every 48 hours in escalating single doses of 0.2, 2, 20, 100, and 200 mg/kg, signs of bleeding were seen at 100 and 200 mg/kg, and three out of four rabbits in these groups died. The 100 and 200 mg/kg doses are at least 100-fold higher than the lung dose that have shown to be safe and effective in COPD patients [15].

In recent short-term exposure studies in dogs, single doses of UFH up to 1,000 mg, aerosolised or instilled directly in to the trachea produced no local or systemic anticoagulation [16]. In mice exposed to an aerosol of heparin at 10 µg/mL for 30 minutes, animals showed no signs of haemorrhage, anaemia or ulcerative lesions [10]. There was no other type of lesion found.

In long term animal studies, lung tissue from dogs that received intrapulmonary heparin (10 to 15 mg/kg by intra-tracheal instillation) for a year showed no signs of haemorrhage, anaemia or ulcerative lesions. There was no other type of lesion found. Heparin reduced the degree of cellular infiltration compared to untreated dogs, indicating its anti-inflammatory effect on inhalation [10].

5.4.2 CARDIOVASCULAR SAFETY PHARMACOLOGY

Many toxicological studies have investigated routes of administration of heparin other than the inhaled route, reflecting its use as an anticoagulant. Toxicological information on acute

toxicity of heparin sodium via IV, SC, intraperitoneal and oral administration in animals is presented in Table 3.

Table 3 Acute Animal Toxicity for Heparin Sodium

Species	Route	LD_{50} (mg/kg)	Source of Data
Rat	Oral	3895	IYKEDH 23,201,1992
Rat	Subcutaneous	234	IYKEDH 23,201,1992
Rat	Intravenous	199	IYKDEH 23,201,1992
Mouse	Oral	5000	NIIRDN, 1084,1990
Mouse	Intraperitoneal	2500	NIIRDN, 1084,1990
Mouse	Subcutaneous	>2500	NIIRDN, 1084,1990
Mouse	Intravenous	2800	JPETAB 102,156,1951
Dog	Intravenous	1000	JPETAB 102,156,1951

Abbreviations: IYKDEH, Iyakuhin Kenkyu. Study of Medical Supplies; JPETAB, Journal of Experimental Pharmacology and Experimental Therapeutics; LD_{50}, median lethal dose; NIIRDN, Drugs in Japan. Ethical Drugs

Toxicological information based on chronic heparin administration with repeat exposure via the IV and SC routes is shown in Table 4.

Table 4 Chronic Animal Toxicity

Target	Species	Route	Dose	Effect
Reproduction	Rats	Subcutaneous	10 mg/kg/day	No effect on<br>conception (implantation sites)<br>pregnancy (foetal weight)<br>teratogenicity
Organogenesis	Pregnant rats and rabbits	Intravenous	50 mg/kg/day	Increased number of early resorptions<br>No evidence of teratogenicity
Mutagenicity	Rat	Bone marrow micronucleus test	Data not available	No evidence of mutagenicity

Heparin does not cross the placental barrier and no teratogenic effects have been reported. In addition, there is no evidence of mutagenicity in the Ames assay and human lymphocyte mutagenicity testing.

The maximum daily IV dose of heparin recommended in man is 60,000 units/day or 1000 units/kg/day (5 mg/kg/day) in an average 60-kg person [18]. In clinical trials of inhaled UFH in patients with COPD, delivery of aerosolised heparin at concentrations in the nebuliser to achieve lung doses of 1 mg/kg twice a day is proposed, of which _none_ is expected to reach the circulation or have systemic effects.

5.4.3 CENTRAL NERVOUS SYSTEM EFFECTS

Effects of inhaled heparin on the central nervous system have not been reported, but IV heparin is reported to be neuroprotective in rats [19].

5.5 Pharmacokinetics and Product Metabolism in Animals

The pharmacokinetics (PK) of IV heparin are well described [20]. Heparin clearance from blood involves a combination of rapid saturable clearance by the endothelium and macrophages and a slower non-saturable renal clearance. In dogs, mice, rats, and human, doses of intrapulmonary heparin >8 mg/kg are required for detectable IV anticoagulation [10]. More recent studies confirmed that at 8 mg/kg, instillation of liquid heparin into the lungs of rabbits resulted in low bioavailability for systemic anticoagulation [11,21]. Therapeutic systemic anticoagulation was only achieved using dry particle heparin.

Intratracheal heparin is taken up by pulmonary macrophages and, at doses >8mg/kg, by endothelial cells [22].

5.6 Analytical methodology

The anti-Xa activity of UFH cannot be measured in sputum or other airway samples as cationic proteins present in sputum samples neutralise heparin and inhibit this activity [15]. A number of different approaches for analysing UFH in biological samples have been reported, including the metachromatic (Farndale) assay, electrophoretic assay, and assays of hexosamine and uronic acid content, of which the hexosamine assay may be the most reliable [22].

5.7 References

1. Mulloy B, Hogwood J, Gray E, Lever R, Page CP. Pharmacology of Heparin and Related Drugs. Pharmacol Rev. 2016;68:76-141.
2. Shute JK. Glycosaminoglycan and chemokine/growth factor interactions. In Heparin-a century of progress. Handbook of Experimental Pharmacology. 2012;207:307-324.
3. Riffo-Vasquez Y, Somani A, Man F, Amison R, Pitchford S, Page CP. A non-anticoagulant fraction of heparin inhibits leukocyte diapedesis into the lung by an effect on platelets. Am J Respir Cell Mol Biol. 2016;55:554-563.
4. Spencer JL, Stone PJ, Nugent MA. New insights into the inhibition of human neutrophil elastase by heparin. Biochemistry. 2006;45:9104-20.
5. Lever R, Lo WT, Faraidoun M, Amin V, Brown RA, Gallagher J, Page CP. Size fractioned heparins have differential effects on human neutrophil function in vitro. Br J Pharmacol. 2007;151:837-843.
6. Acharya KR, Ackerman SJ. Eosinophil granule proteins: form and function. J Biol Chem. 2014;289:17406-15.
7. Coyle AJ, Uchida D, Ackerman SJ, Mitzner W, Irvin CG. Role of cationic proteins in the airway. Hyperresponsiveness due to airway inflammation. Am J Respir Crit Care Med. 1994;150:S63-71.

8. Grant S, Long WF, Mackintosh G, Williamson FB. The anti-oxidant activity of heparins. Biochem Soc Trans 1996;24:194S.
9. Tuinman PR, Dixon B, Levi M, Juffermans NP, Schultz MJ. Nebulized anticoagulants for acute lung injury - a systematic review of preclinical and clinical investigations. Crit Care. 2012;16:R70.
10. Jacques LB, Mahadoo J, Kavanagh LW. Intrapulmonary heparin. Lancet 1976;308:1157-1161.
11. Qi Y, Zhao G, Liu D, Shriver Z, Sundaram M, Sengupta S, Venkataraman G, Langer R, Sasisekharan R. Delivery of therapeutic levels of heparin and low-molecular-weight heparin through a pulmonary route. Proc Natl Acad Sci U S A. 2004;101:9867-72.
12. Chimenti L, Camprubí-Rimblas M, Guillamat-Prats R, Gomez MN, Tijero J, Blanch L, Artigas A. Nebulized Heparin Attenuates Pulmonary Coagulopathy and Inflammation through Alveolar Macrophages in a Rat Model of Acute Lung Injury. Thromb Haemost. 2017;117:2125-2134.
13. Ahmed T, Abraham WM, D'Brot J. Effects of inhaled heparin on immunologic and nonimmunologic bronchoconstrictor responses in sheep. Am Rev Respir Dis. 1992;145:566-70.
14. Williams PD, Tyrrell DJ, Storm NE, Holme KR. Use of a precise intratracheal delivery system to compare the acute tolerance of heparin and the heparinoid GM2000 in rabbits. Toxicology Mechanisms and Methods 1997;7:1-7.
15. Shute JK, Calzetta L, Cardaci V, di Toro S, Page CP, Cazzola M. Inhaled nebulised unfractionated heparin improves lung function in moderate to very severe COPD: A pilot study. Pulm Pharmacol Ther. 2018;48:88-96.
16. Manion JS. Anticoagulant effects of inhaled unfractionated heparin in the dog as determined by partial thromboplastin time and factor Xa activity. J Vet Emerg Crit Care (San Antonio). 2016;26:132-6.
17. CFNP Technical Advisory Panel Review Heparin. 2002.
18. http://www.fda.gov/Safety/MedWatch/SafetyInformation/ucm219000.htm.
19. Zheng Y, Zhang YP, Shields LB, Zhang Y, Siu MW, Burke DA, Zhu J, Hu X, Dimar JR, Shields CB. Effect of heparin following cervical spinal cord injuries in rats. Neurosurgery. 2011;69:930-41;
20. Mahadoo J, Hiebert LM, Wright CJ, Jaques LB. Vascular distribution of intratracheally administered heparin. Ann N Y Acad Sci. 1981;370:650-5.
21. Hirsh J, Warkentin TE, Shaughnessy SG, Anand SS, Halperin JL, Raschke R, Granger C, Ohman EM, Dalen JE. Heparin and low-molecular-weight heparin: mechanisms of action, pharmacokinetics, dosing, monitoring, efficacy, and safety. Chest. 2001;119(1 Suppl):64S-94S.
22. Lammers G, van de Westerlo EM, Versteeg EM, van Kuppevelt TH, Daamen WF. A comparison of seven methods to analyze heparin in biomaterials: quantification, location, and anticoagulant activity. Tissue Eng Part C Methods. 2011;17:669-76.

6.0 EFFECTS IN HUMANS

6.1 Pharmacokinetics and Product Metabolism in Humans

Studies of the lung deposition and clearance of ^{99m}Tc-heparin in healthy volunteers showed that only 8% of a jet nebuliser dose reaches the lower respiratory tract, and of that, 39% remained after 24 hours [1]. Additional PK studies in healthy volunteers confirmed the long half-life of intrapulmonary heparin, reported as being 28 hours [2]. The slow clearance of intrapulmonary heparin is believed to be due to uptake by pulmonary macrophages and its subsequent slow release.

6.2 Safety and Efficacy

Importantly, no acute or chronic toxicological effects have been associated with the inhaled route for any dose of UFH in man. In healthy human volunteers, 300 to 1500 mg heparin was administered as an aerosol with no sign of toxicity [3]. Only high doses of heparin >8 mg/kg were reported to produce a moderate sustained increase in coagulation time in healthy volunteers [3]. Others have reported no clinically significant effect of nebuliser loading doses of inhaled heparin up to 2000 mg, of which 8% (160 mg) is delivered to the lung, equating to 2.6 mg/kg in an average 60-kg subject, on systemic coagulation in healthy subjects [4].

A 2015 review of 20 studies in 536 patients [5] showed that inhaled UFH was safe and effective in patients with smoke inhalation injury, acute lung injury, asthma, and allergy. No incidence of pulmonary haemorrhage associated with inhaled heparin has been reported in any study. These studies, including studies of inhaled UFH in patients with CF, are summarised in Table 5, Table 6, Table 7 and Table 8.

Not even pulmonary lavage with intratracheal instillation of 200 to 250 mg/kg of heparin to treat alveolar proteinosis induced bleeding into the lungs or was associated with lung damage a year later [3]. In addition, inhaled heparin did not induce allergic reactions in any study, not even when followed up over long periods of up to 485 days. In a study of the effect of inhaled heparin in patients with idiopathic pulmonary fibrosis (IPF), 750 mg as the nebuliser fill dose was deemed to be the threshold dose above which effects on systemic coagulation could first be detected. In a compassionate phase of the study, patients continued to use inhaled UFH for up to 100 weeks, without side effects [2].

In clinical trials in patients with CF it is proposed to deliver 60 mg of inhaled UFH to the lung, which equates to 1 mg/kg in an average 60-kg patient. There is no evidence for toxicity or adverse side effects at this dose from any previous study in man, in health and disease.

Limitations and Risks;

The side effects of IV heparin therapy are well known, and include bleeding, anaphylaxis, heparin-induced thrombocytopenia, and osteoporosis [reviewed in 6].

The risk of haemoptysis may be considered the greatest risk of inhaled heparin therapy in CF patients. However, major haemoptysis is a rare event, occurring in 4.1% of all patients with

CF [7] and 1% of children with CF [8]. It is believed to occur in patients with diffuse severe lung disease and is rarely seen in children under 10 years of age. Haemoptysis is believed to be due to erosion of enlarged thin-walled and newly developed bronchial vessels, found in areas of bronchiectasis secondary to chronic infection.

Observations that the anticoagulant activity of heparin is neutralised immediately on contact with CF sputum indicates that the mucolytic effects observed are unrelated to its anticoagulant activity. Thus, the perceived increased risk of haemoptysis is limited by airway sputum itself and the evidence is that inhaled heparin is a safe therapy in CF patients.

6.3 Marketing Experience

Heparin is not yet approved for use by inhalation for any indication.

6.4 Rational for dose selection

In a previous Phase IIB study in 15 patients with severe to very severe COPD, no effect was found of inhaled UFH on systemic coagulation with an estimated lung dose of 60 mg, or 1 mg/kg in an average 60-kg patient. It is believed that this is an effective and safe dose to use in patients.

6.5 References

1. Bendstrup KE, Chambers CB, Jensen JI, Newhouse MT. Lung deposition and clearance of inhaled (99m)Tc-heparin in healthy volunteers. Am J Respir Crit Care Med. 1999;160:1653-8.
2. Markart P, Nass R, Ruppert C, Hundack L, Wygrecka M, Korfei M, Boedeker RH,Staehler G, Kroll H, Scheuch G, Seeger W, Guenther A. Safety and tolerability of inhaled heparin in idiopathic pulmonary fibrosis. J Aerosol Med Pulm Drug Deliv. 2010;23:161-72.
3. Jacques LB, Mahadoo J, Kavanagh LW. Intrapulmonary heparin. Lancet 1976;308:1157-61.
4. Bendstrup KE, Gram J, Jensen JI. Effect of inhaled heparin on lung function and coagulation in healthy volunteers. Eur Resp J 2002;19:606-610.
5. Monagle K, Ryan A, Hepponstall M, Mertyn E, Monagle P, Ignjatovic V, Newall F. Inhalational use of antithrombotics in humans: Review of the literature. Thrombosis Res 2015;136:1059-1066.
6. Papa A, Danese S, Gasbarrini A, Gasbarrini G. Review article: potential therapeutic applications and mechanisms of action of heparin in inflammatory bowel disease. Aliment Pharmacol Ther 2000;14:1403-1409.
7. Flume PA, Yankaskas JR, Ebeling M, Hulsey T, Clark LL. Massive hemoptysis in cystic fibrosis. Chest. 2005;128:729-38.
8.
9. Barben JU, Ditchfield M, Carlin JB, Robertson CF, Robinson PJ, Olinsky A. Major haemoptysis in children with cystic fibrosis: a 20-year retrospective study. J Cystic Fibrosis 2003;2:105-111.

Table 5 Description of Studies Reporting Inhaled Heparin Use as an Anticoagulant in Adult Patients with Smoke Inhalation and Acute Lung Injury

Author (year)	Study design	Dose	Patients (n)	Clinical condition	Therapeutic outcome
Desai et al. (1998)	Controlled trial	5000 IU UFH	Total=90<br>47 treatment<br>age 8.2 years (±6)<br>43 controls<br>age 7.7 years (±5)	Inhalation injury in paediatric patients	• Decreased reintubation<br>• Decreased atelectasis<br>• Decreased mortality
Holt et al. (2008)	Retrospective chart review	UFH 5000 IU/mL+ N-acetylcystine+albuterol every 4 hours for 7 days or until extubation	Total=150<br>62 treatment<br>age 44 years (±25)<br>88 untreated<br>age 42 years (±26)	Inhalation injury	• No difference between groups re: mortality, length of stay, duration of ventilation or PaO_2/FIO_2<br>• Incidence of pneumonia higher in the treatment group vs untreated group<br>• Evident risk of bias in selection of patients to receive treatment
Miller et al. (2009)	Retrospective study with historical control	Treatment 10,000 IU UFH every 4 hours	Total=30<br>16 treatment<br>14 control<br>age range 18–80 years	Inhalation + mechanical ventilation	• Improvement in lung injury scores, resistance, compliance and hypoxia scores<br>• Significant survival benefit especially if APACHE III score>35
Dixon et al. (2008)	Open label trial of 4 doses of nebulised UFH	Nebulized UFH (50,000 to 400,000 IU/day) delivered 12-hourly for 2 days.	Total=16<br>age 58 years (±14)	Acute lung injury	• No effect on lung compliance, oxygenation or alveolar dead space fraction<br>• Dose-dependent impact on coagulation parameters: 50,000 IU/day had no impact APTT or TCT assays. All other UFH doses resulted in peak serum concentration on the Day 2<br>• 400,000 IU UFH rapidly produced a therapeutic APTT, which reduced quickly following cessation
Yip et al. (2011)	Retrospective study with historical control	Treatment 5000 IU UFH+ N-acetylcysteine+salbutamol delivered every 4 hours for 7 days	Total=63<br>52 treatment<br>age 42 years (±16.1)<br>11 control<br>age 42 years (±13.8)	Inhalation injury (in the setting of burns)	• No increased risk of bleeding with nebulised UFH<br>• Changes in APTT, PT and platelet counts were comparable between the groups<br>• No difference in number or type of bleeding episodes between the groups

Abbreviations: APTT, activated partial thromboplastin time, FIO_2, fractional inspired oxygen; IU, international units; PaO_2, partial pressure of arterial oxygen; PT, prothrombin time; TCT, thrombin clot time; UFH, unfractionated heparin

Source: [5]

Table 6 Description of Studies Reporting Effects of Inhaled Unfractionated Heparin in Adult Patients with Asthma or Allergy

Author (year)	Study design	Drug and dose	Patients	Clinical condition	Therapeutic outcome
Ahmed et al. (1993)	Single blind, randomized, placebo-controlled crossover	UFH 1000 IU/kg vs cromolyn sodium (20 mg) vs placebo	N=12<br>age 27 years (range 15–41)	Exercise-induced asthma in known asthmatics	• Attenuated ↓ in airway conductance<br>• Did not change APTT<br>• Did not modify bronchoconstrictor response
Ceyhan and Celikel (1995)	Single blind, randomized, placebo-controlled crossover	UFH 1000 IU/kg (max 60,000 IU)	N=13<br>age 30 years (±11)	Methacholine-provocation in mild asthmatics	• ↑ methacholine PD20 required to achieve pre-determined ↓ in FEV1<br>• ↓ airway hyperactivity
Diamant et al. (1996)	Double blind, placebo-controlled crossover	UFH 1000 IU/kg (max 80,000 IU) 5 doses over 7.5 hours	N=8<br>Age range 22–33 years	Inhaled dust-mite in mild to moderate asthmatics	• No effect on baseline FEV<br>• ↓ late asthmatic response by 36%<br>• ↓ late asthmatic response to allergen
Garrigo et al. (1996)	Single blind, Randomized crossover	UFH 80,000IU (4 mL, 20,000 IU/mL)	N=9<br>age 25 years (±4)	Exercise-induced asthma in known asthmatics	• Time dependent ↓ in exercise-induced asthma<br>• ↓ Bronchoconstriction responses by 58%, 78% and 67% when nebulised 15 min, 1 h and 3 h before exercise<br>• Ineffective when administered 6 h before exercise<br>• UFH prevents exercise-induced asthma for 3 h
Pavord et al. (1996)	Double blind, Randomized	UFH 1000 IU/kg (max 80,000 IU)	N=11<br>age 34 years (range 23–42)	Sodium metabisulphite and methacholine provocation in mild asthmatics	• UFH did not reduce sodium metabisulphite or methacholine-induced bronchoconstriction
Ceyhan and Celikel (1997)	Single blind, randomized crossover	UFH 1000 IU/kg (max 60,000 IU).	N=15<br>age 29 years (±11)	Methacholine provocation versus adenosine-induced bronchial reactivity in mild asthmatics	• Compared "direct" (methacholine) vs "indirect" (adenosine) stimulation of bronchoconstriction<br>• UFH demonstrated ↑ protection against bronchoconstriction induced by methacoline vs adenosine<br>• Inhaled UFH mechanism of action likely a direct effect on airway smooth muscle not inhibition of inflammatory mediators.
Polosa et al. (1997)	Double blind, randomized, placebo-controlled	UFH 15000 IU/mL	Phase 1: n=10<br>Phase 2: n=7	Methacholine and AMP provocation in asthmatics	• Inhaled UFH produced a 20% reduction in AMP-induced bronchoconstriction

Author (year)	Study design	Drug and dose	Patients	Clinical condition	Therapeutic outcome
			age 27 years (±1.7)		• No effect on methacholine-induced bronchoconstriction<br>• Positive benefit exhausted by 180min post-inhalation
Kalpaklioglu et al. (1997)	Double blind, randomized crossover	UFH 20,000 IU/mL	N=2<br>age 31.8 years (±3)	Methacholine provocation in allergic asthmatics	• Maximal expiratory air-flow parameters similar<br>• No treatment order effect<br>• Increased in PC20 methacholine post UFH
Ahmed et al. (1999)	Double blind, randomized cross over	UFH 80,000 IU vs placebo vs LMWH (0.5, 1 or 2 mg/kg)	N=13<br>age 27 years (range 16–42)	Exercise-induced asthma (+ a sub-population analysis of methacholine provocation) in known asthmatics	• 0.5 mg/kg LMWH produced equivalent inhibition of bronchoconstriction to UFH<br>• 2 mg/kg LMWH produced greatest inhibition of exercise-induced bronchoconstriction but did modify response to methacholine<br>• 2 mg/kg LMWH did not alter plasma anti-Xa activity<br>• Mechanism of action not due to systemic anticoagulation
Bendstrup and Jenson (2000)	Case report	UFH 7000 IU	N=2<br>57 and 67 years	Non-provoked exacerbation of asthma	• Inhaled UFH administered daily for 5 days<br>• Respiratory function improved immediately<br>• No impact on haemostatic parameters.
Ceyhan and Celikel (2000)	Single blind, randomized, placebo-controlled crossover	UFH 1000 IU/kg (max 60,000 IU) vs LMWH 0.8 mg/kg (max 60 mg) vs placebo	N=15<br>age 33 years (±13)	Methacholine-provocation in mild asthmatics	• UFH and LMWH ↓ bronchoconstriction with no statistical difference in relative protection<br>• 3 subjects in the UFH group and 2 subjects in the LMWH group showed increased reactivity after inhalation
Tranfa et al. (2000)	Double blind, randomized, placebo-controlled, cross over	UFH 1000 IU/kg (max 60,000 IU)	N=8<br>age 32 years (range 20–49)	Water-induced bronchoconstriction in allergic asthmatics	• UFH gave greater protection against all doses of water-induced bronchoconstriction compared to placebo.

Author (year)	Study design	Drug and dose	Patients	Clinical condition	Therapeutic outcome
Tutluoglu et al. (2001)	Double blind, Placebo-controlled	UFH 1000 IU/kg	N=38<br>Control n=18<br>age 32.6 years (±11.9)<br>Treatment n=20<br>age 30.4 years (±12.4)	Hypertonic KCl-induced bronchoconstriction in asthmatics	• UFH afforded a reduction in bronchospasm<br>• Proposed mechanism of UFH action is inhibition of mediator release, not via smooth muscle.
Stelmach et al. (2003)	Double blind, randomized, placebo-controlled crossover	UFH 5000 IU	N=23<br>Age range 12–18 years	Histamine or Leukotriene D4 provocation in children with mild asthma	• No systemic anticoagulant effect detected<br>• A single dose of inhaled UFH reduced bronchoconstriction<br>• No difference in FEV in any group.

Abbreviations: AMP, adenosine monophosphate; APTT, activated partial thromboplastin time; FEV(1), forced expiry volume (in 1 second); IU, international units; LMWH, low molecular weight heparin; PC, provocative concentration; PD, provocative dose; UFH, unfractionated heparin
Source: [5]

Table 7 Studies of Nebulised Unfractionated Heparin in Adult Patients with Cystic Fibrosis

Author (year)	Study design	Drug and dose	Patients	Clinical condition	Therapeutic outcome
Ledson et al.<br><br>(2001)	Quasi experimental (pretest-posttest design	UFH, 25,000 IU a day for 7 days	N=6<br><br>Age 27.7 yr (range 21-34 years)	Adult CF patients colonised with Burkholderia cepacia<br><br>FEV_1 55.0±4.8	No change in spirometry with UFH. Reduced sputum and serum IL-6 and IL-8 post-treatment. Subjective sputum mucolysis. No evidence of bleeding or thrombocytopenia.
Serisier et al.<br><br>(2006)	Randomised, double blind, placebo-controlled cross over	UFH, 50,000 IU twice a day for 14 days.	N=14<br><br>Age 23±7.8 years	Adult CF patients<br><br>FEV_1 52.1±15.56%	No effect of UFH on spirometry or inflammatory markers. No evidence of bleeding or thrombocytopenia.

Abbreviations: FEV_1, forced expiry volume in 1 second; IL, interleukin; IU, international units; UFH, unfractionated heparin

Inhaled Unfractionated Heparin Investigator's Brochure

Table 8 Studies in Patients with Chronic Obstructive Pulmonary Disease

Author (year)	Study design	Drug and dose	Patients	Clinical condition	Therapeutic outcome
Youngchaiyud et al. (1968)	Open label. Placebo (saline and heparin vehicle) controlled	Nebulised UFH in 0.5% phenol	N=20 in test arm<br><br>N=17 in saline arm<br><br>N=18 in 0.5% phenol vehicle control arm	COPD patients.<br><br>FEV_1/FVC = 36%-49%	Both heparin and the vehicle control induced expectoration. An expectorant action was not attributable to heparin. Heparin increased airway conductance, not seen in either control group.
Serisier et al<br><br>(2006-unpublished)	Randomised, double-blind, placebo-controlled crossover study	14 day inhaled, bd, 4 mL 0.9% saline or 50,000 IU of UFH (25,000 IU/mL, made up to 4 mL with 0.9% NaCl solution)	N=8 completed	Stable COPD<br><br>FEV_1; 52.6 (30.9)	No systemic coagulation. No effect on lung function or other parameters.
Shute et al<br><br>(2016)	Randomised, three parallel groups, double blind.	21 days, inhaled UFH, 75,000 or 150,000 IU bd from a jet nebuliser. Aerosolised water control.	N= 9, placebo (water)<br><br>N= 6, 75,000 IU<br><br>N=9, 150,000 IU<br><br>(completed)	COPD, post-exacerbation, using salbutamol and ICS. Some on oral steroids. FEV_1%;<br><br>Placebo; 50.45±3.12<br>75,000 IU; 48.57±2.25,<br>150,000 IU;41.53±2.51	No systemic coagulation. Significant improvement in FEV_1 at 7 days with 150,000 IU. At 21 days significant effect on gas trapping at high dose. Improvement in exercise capacity and dyspnoea with both doses.

Abbreviations: COPD, chronic obstructive pulmonary disease; FEV_1, forced expiry volume in 1 second; FVC, forced vital capacity; ICS, inhaled corticosteroids; IL, interleukin; IU, international units; UFH, unfractionated heparin

7.0 SUMMARY OF DATA AND GUIDANCE FOR THE INVESTIGATOR

7.1 Indications and Subject Exposure

Inhaled UFH has been investigated in acute lung injury, in ventilated patients, those with inhalation burns injury, patients with asthma, COPD, IPF, and CF. In no study has any adverse event associated with this route of administration been reported. Only high doses > 8 mg/kg are associated with systemic anticoagulation.

7.2 Dosage and Method of Administration

In healthy subjects, inhalation of 150,000 IU (750 mg) heparin ("filled dose") significantly increased the partial thromboplastin time and anti-factor Xa activity in blood samples indicating the threshold dose for systemic anticoagulation and the maximum dose to be delivered [1]. Assuming 8% of the dose was delivered to the lung, this equates to 60 mg.

In IPF patients, no acute deleterious effects on pulmonary function, gas exchange, or exercise capacity were noted after inhalation of the threshold dose. During chronic treatment, where one-quarter of the threshold dose was inhaled every 12 hours for 28 days to obtain a steady-state anticoagulant activity in the alveolar space approximating the anticoagulant activity observed after threshold dose inhalation, no heparin-related side effects, such as haemoptysis or heparin/platelet factor 4 antibodies and thrombocytopenia, were detected in any subject [1].

Heparin sodium (Monoparin 25,000 IU/mL solution for injection, Wockhardt UK) will be administered as a nebulised aerosol dose of 25,000 IU heparin every 6 hours diluted to 4 mL in 0.9% saline via an Aerogen Solo (Aerogen, Ireland) vibrating mesh aerosol drug nebuliser. It has been estimated that 20% of the heparin dose is administered to the lungs by this method using Anderson cascade impactor (data on file).

Doses will be made up immediately before use as follows: one 1-mL ampoule of Heparin sodium 25,000 IU/mL without preservative will be added to the nebuliser chamber followed by 4.0 mL Sodium Chloride Injection BP 0.9%. The chamber will be assembled and swirled to mix the heparin thoroughly.

Nebulised Heparin Sodium 25,000 IU will be delivered using air or oxygen every 6 hours, daily for up to 21 days, or until the patient has no respiratory symptoms.

The mode of delivery (via mask, mouthpiece, or endotracheal tube) will be chosen by the clinical team depending on the patient's needs for oxygen and ventilatory support.

7.3 Possible Adverse Effects

The risk of haemoptysis may be considered the greatest risk of inhaled heparin therapy in CF patients. Haemoptysis is a relatively common complication of CF in adults, occurring in approximately 9.1% of patients and is usually associated with older patients and more severe

disease [2]. Haemoptysis may be scant (<5 mL) but massive haemoptysis (>240 mL) is reported to occur in 4.1% of patients [3]. However, major haemoptysis is a rare event in childhood, occurring in 1% of children with CF [4].

The observation that the anticoagulant activity of heparin is neutralised immediately on contact with CF sputum indicates that the mucolytic effects observed are unrelated to its anticoagulant activity. Thus, the perceived increased risk of haemoptysis is limited by airway sputum itself and the evidence is that inhaled heparin is a safe therapy in CF patients.

In long-term inhalation studies, others used inhaled heparin in the treatment of migraine at doses of 5000 units (30 mg) weekly for periods of over a year and up to 4 years in one case. In these studies, heparin was well tolerated with no evidence of hypersensitivity. The only side effect reported was an occasional headache in this patient group and there was no significant change in clotting time [5].

Although high doses of heparin >8 mg/kg were reported to produce a moderate sustained increase in systemic coagulation time in normal healthy volunteers [6], others have reported no clinically significant systemic anticoagulation effect of nominal doses of inhaled heparin of up to 400,000 units (2,000 mg) in normal subjects, which equates to a lung dose of 160 mg [7]. In one patient with alveolar proteinosis who received 2.4 million units of heparin (200 to 250 mg/kg) in a pulmonary lavage, no bleeding of the bronchial system was observed [6].

Reference Safety Information:

For the purposes of regulatory reporting, all serious adverse reactions (SARs) are considered unexpected and will be reported as suspected unexpected serious adverse reactions (SUSARs).

Refer to the information in summary of product characteristics (SmPC) [8] for full safety details.

Patients can expect to expectorate larger volumes of sputum, reflecting the pharmacological effect of a mucolytic, and although this is not an adverse event, patients may score this effect negatively on visual analogue scales reflecting body image or social marginalization [9]. Therefore, patients should be advised of this likely effect and that it is a beneficial effect.

Possible adverse effects associated with inhaled heparin:

- Headache
- Epistaxis
- Systemic anticoagulation (this is not expected at the dose used)
- Haemoptysis (although for all the reasons given above this is unlikely to be associated with inhaled heparin and is as likely to be seen in the placebo group)

7.4 Contraindications

IV UFH is contraindicated for concomitant use of other anticoagulants, nonsteroidal anti-inflammatory drugs, and angiotensin-converting enzyme inhibitors. However, there are no reported contraindications for the non-anticoagulant local pulmonary effects of inhaled UFH at the proposed dose.

7.5 Specific Guidance for Handling

Patients with a recent history of haemoptysis, within the last 3 weeks, or heparin-induced thrombocytopenia or platelet count $<10^8$/mL should not use inhaled heparin.

7.6 Precautions

Even massive doses of intrapulmonary heparin are not associated with pulmonary haemorrhage [6], and overdose or dependency are unlikely. However, protamine sulphate is an appropriate antidote for heparin overdose.

7.7 References

1. Markart P, Nass R, Ruppert C, Hundack L, Wygrecka M, Korfei M, Boedeker RH,Staehler G, Kroll H, Scheuch G, Seeger W, Guenther A. Safety and tolerability of inhaled heparin in idiopathic pulmonary fibrosis. J Aerosol Med Pulm Drug Deliv. 2010;23:161-72.
2. Hurt K, Simmonds NJ. Cystic fibrosis: management of haemoptysis. Paediatr Respir Rev. 2012;13:200-5.
3. Flume PA, Yankaskas JR, Ebeling M, Hulsey T, Clark LL. Massive hemoptysis in cystic fibrosis. Chest. 2005;128:729-38.
4. Barben JU, Ditchfield M, Carlin JB, Robertson CF, Robinson PJ, Olinsky A. Major haemoptysis in children with cystic fibrosis: a 20-year retrospective study. J Cystic Fibrosis 2003;2:105-111.
5. Thonnard-Neumann E. Migraine therapy with heparin: pathophysiologic basis Headache 1977;16:284-292.
6. Jacques LB, Mahadoo J, Kavanagh LW. Intrapulmonary heparin. Lancet 1976;308:1157-1161.
7. Bendstrup KE, Gram J, Jensen JI. Effect of inhaled heparin on lung function and coagulation in healthy volunteers. Eur Resp J 2002;19:606-610.
8. Heparin sodium 25,000 I.U./ml solution for injection or concentrate for solution for infusion [Summary of Product Characteristics]. UK: Wockhardt UK Ltd; 2018.
9. Serisier DJ, Shute JK, Hockey PM, Higgins B, Conway J, Carroll MP. Inhaled heparin in cystic fibrosis. Eur Respir J. 2006;27:354-8.

Nebulized heparin in patients with mainly moderate coronavirus disease 2019: Randomized controlled trial

Tarek. I. Ismail

Anesthesia and Surgical Intensive Care, Faculty of Medicine, Helwan University, Egypt

Introduction

The COVID-19 pandemic has quickly spread throughout the world and threatens to overwhelm our critical care bed supply in Egypt in the upcoming weeks. Salvage use of therapeutics targeted at attenuating acute respiratory distress syndrome (ARDS) as a sequalae COVID-19-related mortality is of high interest [(1)]. There is growing evidence that lethal COVID-19 ARDS is associated with disseminated intravascular fibrin deposition and in the alveolar sac [(2)]. Current therapeutic strategy to decrease ARDS associated mortality is to utilize protective mechanical ventilation in combination with low tidal volume. However, morbidity and mortality remain high, both exceeding 40% [(3)]. The need for new specific pharmacological therapies has carried to examine the role of altered coagulation and fibrinolysis in the pathogenesis of ARDS. Nebulization of heparin may offer benefits over systemic administration because nebulization enhances delivery to the bronchial tree and the alveolar sacs and reduces the potential for systemic bleeding associated with intravenous administration. Furthermore, nebulized heparin has been shown to reduce levels of coagulation activation in the lungs both in animal studies and in patients with ALI [(4)]. As Heparin prevents further fibrin deposition but ineffective in the removal of pre-existing fibrin plug, so early use of heparin during the course of the disease may limit the complications of ARDS and/or reduce the burden on ventilatory support in intensive care units.

Aim of the study:

The aim of this study is to assess whether prophylaxis treatment with nebulized heparin could stop the progression of the disease and improve lung function by preventing further fibrin accumulation in the lungs in patients with COVID-19 who expected to require mechanical ventilation.

Ethical approval:

Patients and methods:

A randomized controlled clinical trial will be conducted. Adult patients with COVID-19 will be selected with the following inclusion criteria:Age:18-60 years old, recently diagnosed (within 24 h) and moderate symptoms of the disease ongoing SARS-CoV-2 infection confirmed in upper or lower respiratory tract specimens with real time reverse transcriptase polymerase chain reaction (RT-PCR), willingness to participate. Pneumonia on computed tomography of the chest will not be mandatory for inclusion.

Exclusion criteria will be: age below 18 years, severe conditions including malignancies, heart, liver, or kidney disease, poorly controlled metabolic diseases, pregnancy or lactation, severe hepatic impairment (e.g. Child Pugh grade C, alanine aminotransferase more than fivefold the upper limit), severe renal impairment (estimated glomerular filtration rate ≤30 mL/min/1.73 m2) , receipt of continuous renal replacement therapy, hemodialysis, peritoneal dialysis, allergy to heparin (including any history of heparin-induced thrombocytopenia), pulmonary hemorrhage in the previous 3 months, uncontrolled bleeding or a significant bleeding disorder, an intracranial hemorrhage in the past 12 months and patients with mild and severe COVID-19 will be excluded.

The definition of disease severity of covid-19 was based on the fifth version of the Chinese guideline for the management of covid-19 [(5)]: Mild disease includes patients with mild symptoms but no manifestation of pneumonia on imaging. Moderate disease includes patients with fever, cough, sputum production, and other respiratory tract or non-specific symptoms along with manifestation of pneumonia on imaging but no signs of severe pneumonia defined as the presence of SaO2/SpO2 below 94% on room air or a PaO2 to FiO2 ratio of 300 or lower and Respiratory rate ≥30 breaths/min .

Intervention:

This study will be conducted at 15th May or El Agmy hospital. Patients will be randomized into two groups: standard of care (SOC) group where patients will be managed with standard of care aligned with the indications from the updated national clinical practice guidelines of ministry of health in Egypt and (SOC plus) group where patients will be managed with the same standard care plus nebulized heparin (1,000 IU/kg [predicted body weight]) every 6 hours stared 24 hour after randomization and will continue for one week. The study medication will be reduced or withheld at the physicians' discretion if any of the following occurred: excessive blood staining of the sputum, other significant bleeding, a planned surgical procedure, or an excessively elevated activated partial thromboplastin time (APTT).

Measurements:

- Demographic data: age, sex, body mass index.

- Coexisting conditions (Diabetes, hypertension and others).

- Vital signs: Body temperature (°C), Pulse (beats/min), Respiratory rate, (breaths/min), mean arterial blood pressure and Pulse oximetry (%).

- The primary outcome will be the average daily ratio of partial pressure of oxygen to FiO2 (PaO2/FiO2) while the patient on room air for 7 days.

- Secondary outcomes will be Levels in pulmonary lavage fluid of fibrin degradation products D-DIMER as a marker of coagulation activation, measured at baseline and on study Days 3 and 7; it will be measured through mini bronchoalveolar lavage (BAL) fluid samples as patients remained non ventilated.

- Daily APPT levels in seconds and Platelet count (×109/L) will be recorded to assess the systemic effects of nebulized heparin.

- Incidence of serious respiratory events that need further respiratory support from randomization to 14 days.

References:

1. Rodriguez-Morales AJ, et al. Clinical, laboratory and imaging features of COVID-19: a systematic review and meta-analysis. Travel Medicine and Infectious Disease. 2020:101623.
2. Tang N, et al. Abnormal coagulation parameters are associated with poor prognosis in patients with novel coronavirus pneumonia. J Thromb Haemost.2020.
3. Villar J, Blanco J, Añón JM, Santos-Bouza A, Blanch L, Ambrós A, et al. The ALIEN study: incidence and outcome of acute respiratory distress syndrome in the era of lung protective ventilation. Intensive Care Med. 2011;37:1932–41.

4. Dixon B, Schultz MJ, Hofstra JJ, Campbell DJ, Santamaria JD: Nebulised heparin reduces pulmonary coagulation activation in acute lung injury. Crit Care 2010, 14:445.
5. National Health Commission of the People's Republic of China. Chinese guideline for the management covid-19 (version 5.0, in Chinese). 2020. http://www.nhc.gov.cn/yzygj/ (accessed on 21/5/2020).

INHALE HEP STUDY PROTOCOL

3.1 Research Design

The *INHALEd nebulised unfractionated HEParin for the treatment of hospitalised patients with COVID-19* meta-trial is a randomized controlled trial and an early-phase study analyzing prospective individual patient data. This research is being conducted in multiple countries: Argentina, Australia, Brazil, Egypt, Ireland, Italy, UAE, USA, and Indonesia.

3.2 Location and Time of Research

This randomized controlled trial will be conducted in the inpatient ward and/or intensive care unit (ICU) at Kiara Ultimate RSUPN Dr. Cipto Mangunkusumo. The research will take place from November 2021 to November 2022.

3.3 Population and Research Sample

The target population is COVID-19 patients being treated at RSUPN Dr. Cipto Mangunkusumo. The research sample consists of the population that meets the inclusion criteria and does not meet the exclusion criteria.

3.3.1 Inclusion Criteria

- Age ≥ 18 years
- Hospitalized
- Positive SARS-CoV-2 PCR results within the last 21 days (samples can be obtained via nasal/ pharyngeal swabs, sputum, tracheal aspiration, bronchoalveolar lavage, or other samples)
- WHO modified ordinal clinical scale 3–5

3.3.2 Exclusion Criteria

- Intubated and on mechanical ventilation or assessed as requiring intubation
- Allergy to heparin or heparin-induced thrombocytopenia
- APTT > 120 seconds, not due to anticoagulant therapy and uncorrected with fresh frozen plasma (FFP)
- Platelet count < $20x10^9$/L
- Pulmonary or uncontrolled bleeding
- Pregnant or possibly pregnant
- Acute brain injury with long-term disability potential
- Myopathy, spinal cord injury, or diseases with prolonged inability to breathe independently (e.g., Guillain-Barre syndrome)
- Limited management options (e.g., “do not intubate” cases)
- Imminent death prognosis (within 24 hours)
- Clinician’s disapproval
- Patient refusal

3.4 Estimated Sample Size

To demonstrate a significant reduction in the primary outcome, a total of 100 samples is required, assuming a reduction in invasive mechanical ventilation from 12% to 6%, with 80% power and a two-tailed significance level of 0.05. RSUPN Dr. Cipto Mangunkusumo will contribute 8 subjects to the total 12 institutions involved in Indonesia.

$$n1 = n2 = 2\left(\frac{(Z\alpha + Z\beta)S}{x1 - x2}\right)^2$$

n1 = Sample size in group 1
n2 = Sample size in group 2
Zα = Standard deviation for Type I error with α = 0.05 (1.96)
Zβ = Standard deviation for Type II error with β = 0.2 (0.84)
X1-X2 = Minimum difference considered significant
S = Standard deviation of the mean difference

3.5 Sample Collection

The selection of subjects is carried out randomly. COVID-19 patients in the Kiara Ultimate Inpatient Unit will be designated to participate in this study. Patients will be enrolled until the minimum sample size quota is met.

3.6 Sample Allocation

Allocation concealment is centrally managed through the randomization process website by the University of Sydney’s Northern Clinical School Intensive Care Research Unit under the supervision of Associate Professor Gordon Doig. Blocks of varying sizes and random seeds will be used for the allocation process. To maintain confidentiality, the block sizes will not be disclosed to local researchers. Stratified randomization will be conducted locally. Patients will be assigned to either the heparin nebulization group or the standard therapy group. The ratio between the two groups must be one-to-one.

3.7 Workflow

1. Researchers submit an ethical review application to the Research Ethics Committee of the Faculty of Medicine, Universitas Indonesia - RSUPN Dr. Cipto Mangunkusumo.
2. Sample recruitment is conducted when subjects meet the inclusion criteria and do not meet the exclusion criteria. Subjects who qualify will undergo the informed consent process.
3. Patients who agree must sign the informed consent form.
4. Research subjects are randomized into one of two groups: the intervention group and the control group. The intervention group consists of subjects who receive the study drug (heparin nebulization) and standard COVID-19 therapy according to the

RSUPN Dr. Cipto Mangunkusumo guidelines (Ventolin and NaCl inhalation). The control group consists of subjects who receive standard COVID-19 therapy according to the RSUPN Dr. Cipto Mangunkusumo guidelines and NaCl nebulization (placebo).

5. The intervention group receives standard COVID-19 therapy as well as inhalation of 25,000 units of sodium heparin in 5 mL normal saline, administered three times per day for 21 days or until symptoms resolve. The control group receives standard COVID-19 therapy as well as inhalation of 0.9% NaCl, administered three times per day for 21 days or until symptoms resolve. Nebulization sessions will last for 15 minutes for each group.
6. Supportive examinations are conducted in accordance with the RSUPN Dr. Cipto Mangunkusumo guidelines and under the supervision of the Patient's Attending Physician. No additional examinations specific to the study will be conducted.
7. Data based on case report forms will be recorded, focusing on the following: incidence of intubation (or death for patients who die before intubation) by day 28 post-randomization, the oxygen saturation ratio measured with an oximeter relative to the fraction of inhaled oxygen (SpO2/FiO2 ratio, highest and lowest daily values), changes in WHO modified ordinal score from admission to day 14, incidence of major bleeding, pulmonary bleeding, epistaxis incidence, heparin-induced thrombocytopenia (HIT) incidence, and any adverse events or other harmful reactions.
8. Each institution must record complete identification of participants involved in the research. Full identification does not need to be transferred or provided to the central coordinator but must allow for re-identification in case of an audit of source documents.
9. Dose modification is not required for patients with renal or liver impairment because there is no association between acute or chronic toxicological effects and inhaled heparin administration at any dose. Studies in healthy adults and previous research have proven that inhaled UFH administration does not result in systemic absorption at these doses.
10. The study drug may be discontinued if the following occurs: unresolvable bronchospasm by bronchodilators, sudden hypoxia, unacceptable clinical increases in APTT, significant hemoptysis, pulmonary or major bleeding, or suspected/confirmed HIT.
11. Although allergic reactions have not been reported in previous studies, anaphylaxis remains a possibility. If this occurs, it must be managed according to RSUPN Dr. Cipto Mangunkusumo guidelines.
12. Throughout the study, subjects will be closely monitored. If any unexpected incidents occur, such as the side effects mentioned above, immediate management will be performed in accordance with applicable SOPs, including guarantees for the subjects.
13. The following therapies may be administered concurrently during the study: deep vein thrombosis prophylaxis with UFH or low molecular weight heparin (LMWH); 'maximum' therapeutic doses of UFH or LMWH for clinically indicated

conditions; non-heparin anticoagulants; anti-thrombotic drugs; protamine; prone positioning; and inhaled nitric oxide. There are no restrictions during the study.

14. Paper records must be stored in a locked room with restricted access. Electronic records must be stored in password-protected computers with access limited to specific personnel.
15. All research materials, including case report forms (CRFs) and study databases, will be retained for a minimum of 15 years after the study's conclusion or as required by local laws. Any unnecessary paper-based materials will be destroyed using a standard-quality paper shredder or other methods that protect participant confidentiality. Any unnecessary electronic research materials will be permanently deleted from all electronic media in detail.
16. All de-identified data will be transferred to the INHALE HEP central data repository. This process and the obligations of each party are described in a separate agreement.
17. The implementation and progress of this research will be routinely monitored by the local management committee and the international executive committee.

3.8 Operational Definitions

Dependent Variable	Operational Definitions	Variable type
Intubated	Patients who were intubated before 28 days.	Categorical
Mortality	Subjects who died before being intubated.	Categorical
Oxygenation Index	The patient's oxygenation status includes driving pressure, APTT, Hb, platelet count, and changes in chest X-ray from baseline to day 2.	Numeric
Length of stay in the hospital	The time from patient admission to discharge from the hospital during treatment or until day 28.	Numeric
Major bleeding	Bleeding that causes death and/or symptomatic bleeding occurring in critical areas or organs (intracranial, intraspinal, intraocular, retroperitoneal, intra-articular, or intramuscular with compartment syndrome) and/or bleeding that results in a hemoglobin drop of 20 g/L or more, or requires the transfusion of two units of whole blood or red cells.	Categorical
Pulmonal bleeding	"Pulmonary bleeding" is bleeding that occurs in the lungs, trachea, or bronchi, accompanied by recurrent hemoptysis or requiring repeated suctioning and is associated with a decline in respiratory status.	Categorical
Heparin-induced thrombocytopenia (HIT)	A decrease in platelet count that cannot be explained and has a positive result for the heparin antibody test.	Categorical
Other harmful reactions	An event that is not part of the expected clinical course, may be related to the study, and is	Categorical

	medically significant or harmful to the patient.	

Indepenent Variable	Operational Definition	Variable Type
Intervensi Nebulisasi heparin	Heparin sodium 25,000 Units in 5 mL by Pfizer Australia Pty Ltd (Sydney, Australia) or equivalent unfractionated heparin, such as Inviclot.	Categorical
Standard protocol	NaCl 0,9% sebanyak 10 ml (placebo)	Categorical

3.9 Data Processing and Presentation

Prospective analysis is generally conducted in studies carried out across multiple countries, which enhances the estimation of effect size due to differing conditions and improves the external validity of the research results. We plan to conduct a prospective analysis on de-identified patient data. Common variables from all data will be combined for analysis. If participants do not consent to participate or withdraw their participation, their data will not be used unless the participants permit, including all time-to-death information. The analysis will be performed using the intention-to-treat method according to the group allocation of participants, regardless of treatment adherence. The subsequent analysis will include participants who declined participation but allowed the use of their data, including primary outcomes, and will exclude those who did not meet the inclusion criteria. Incomplete data will not be imputed. The described multilevel model can address incomplete data due to loss to follow-up. If there are missing observations, the number of observations used will be reported. A two-tailed hypothesis test with a significance level of 0.05 will be applied. Adjustments will not be made for multiple testing, as the interpretation of significance is consistent with the primary or secondary outcomes of the study. SPSS Research Engine Version 24.0 (IBM SPSS Statistics and above) and R Version 3.5.0 (and above) will be used for data analysis.

Title page

Can Nebulised HepArin Reduce acuTE lung injury in Patients with SARS-CoV-2 Requiring Mechanical Ventilation in Ireland

Protocol Version no.:	1	Date: 01/July/2020
Test Drug:	Unfractionated Heparin	
Clinical Phase:	1B/2A	
EudraCT number:	2020-003349-12	
Sponsor Number:	NUIG-2020-003	

Chief Investigator: Prof. John Laffey
School of Medicine
Clinical Science Institute
National University of Ireland, Galway
Galway, Ireland
John.laffey@nuigalway.ie

Co-ordinating Centre: HRB Clinical Research Facility Galway
National University of Ireland Galway
University Hospital Galway
Newcastle Road
Galway, Ireland
(091) 495 964

Sponsor: Prof. Lokesh Joshi
(Vice President of Research)
National University of Ireland Galway
University Road
Galway
(091) 495 678

The study will be conducted in compliance with the protocol, International Conference on Harmonization – Good Clinical Practice (ICH-GCP) and any applicable regulatory requirements.

Confidential

This document contains confidential information that must not be disclosed to anyone other than the sponsor, the investigative team, regulatory authorities, and members of the research ethics committee.

1. SPONSOR PROTOCOL AGREEMENT PAGE

I, the undersigned, am responsible for the initiation, management, and/or financing of a clinical trial and agree to the content of the final clinical trial protocol, as presented.

Signed

____________________________________ ___________________________

Sponsor or Sponsor Representative Date

2. INVESTIGATOR PROTOCOL AGREEMENTs

2.1. Chief Investigator Agreement

I, the undersigned, agree to the content of the final clinical trial protocol, as presented.

Signed

___________________________________ ________________________________

Chief Investigator Date

2.2 Site Investigator Agreement

I, the undersigned, am responsible for the conduct of the trial at this site and agree to the following:

I understand and will conduct the trial according to the protocol, any approved protocol amendments, ICH GCP and all applicable regulatory authority requirements and national laws.

I will not deviate from the protocol without prior written approval from the HPRA and the Ethics Committee, except where necessary to prevent any immediate danger to the participant.

I have sufficient time to properly conduct and complete the trial within the agreed trial period, and I have available an adequate number of qualified staff and adequate facilities for the foreseen duration of the trial to conduct the trial properly and safely. I will ensure that any staff at my site(s) who are involved in the trial conduct are adequately trained regarding the protocol and their responsibilities.

I will ensure that any staff at my site(s) who are involved in the trial conduct are adequately trained regarding the protocol and their responsibilities

Signed

___________________________________ _______________________________

Principal Investigator Date

Document History

Document	Date of Issue	Summary of Change
Version 1	01/July/2020	Initial Release

Abbreviations

APTT, activated partial thromboplastin time

ARDS, acute respiratory distress syndrome

AUC, Area under the curve

COPD, chronic obstructive pulmonary disease

COVID-19, coronavirus disease 2019

CPAP, continuous positive airway pressure

DSUR Development Safety Update Report

ECMO, extra corporeal membrane oxygenation

F_iO_2, fraction of inspired oxygen

HFOV, high frequency oscillation ventilation

HIT, Heparin Induced Thrombocytopaenia

HREC, human research ethics committee

ICU, intensive care unit

INR, international normalized ratio

IV, intravenous

LMWH, low-molecular weight heparin

NREC, National Research Ethics Committee

P_aCO_2, arterial partial pressure of carbon dioxide

P_aO_2, arterial partial pressure of oxygen

PEEP, positive end-expiratory pressure

P/F Ratio, Ratio of P_aO_2 to F_iO_2

pH, power of hydrogen

SARS-CoV-2, severe acute respiratory syndrome coronavirus 2; the virus that causes COVID-19

SC, subcutaneous

SmPC Summary of Product Characteristics

TNF, Tumour Necrosis Factor

t-PA, tissue plasminogen activator

Protocol Synopsis

Full Title	Can Nebulised HepArin Reduce acuTE lung injury in Patients with SARS-CoV-2 Requiring Mechanical Ventilation in Ireland
Short Title	CHARTER-Ireland
Protocol number	NUIG-2020-003
EudraCT number	2020-003349-12
Objectives	To evaluate the effect of nebulised unfractionated heparin on the procoagulant marker, d-dimer, in ICU patients with SARS-CoV-2 requiring invasive mechanical ventilation.<br><br>To evaluate the safety of nebulised unfractionated heparin in ICU patients with SARS-CoV-2 requiring invasive mechanical ventilation.
Study Design	Prospective, phase Ib/IIa, multi-centre, randomised, parallel-group, open label proof of principle study in Ireland.
Subject Population	This trial will be carried out in invasively ventilated ICU patients with suspected or confirmed COVID-19 infection<br><br>Inclusion criteria<br><br>To be eligible, a patient must satisfy all these inclusion criteria:<br><br>1. - Confirmed or suspected COVID-19<br><br>If 'suspected', results must be pending or testing intended<br><br>2. Age 18 years or older<br>3. Endotracheal tube in place<br>4. Intubated yesterday or today<br>5. PaO2 to FIO2 ratio less than or equal to 300 while intubated<br>6. Acute opacities on chest imaging affecting at least one lung quadrant<br>7. 'Acute opacities' do not include effusions, lobar/lung collapse or nodules<br>8. Currently in the ICU or scheduled for transfer to the ICU<br>The 'ICU' is an area designated for inpatient care of the critically ill where therapies including invasive mechanical ventilation can be provided.<br><br>Exclusion criteria<br><br>To be eligible, a patient must have none of these exclusion criteria:<br><br>1. Enrolled in another clinical trial that is unapproved for co-enrolment

	2. Heparin allergy or heparin-induced thrombocytopaenia<br>3. APTT > 120 seconds and this is not due to anticoagulant therapy<br>4. Platelet count < 20 x 109 per L<br>5. Pulmonary bleeding, which is frank bleeding in the trachea, bronchi or lungs with repeated haemoptysis or requiring repeated suctioning<br>6. Uncontrolled bleeding<br>7. Pregnant or suspected pregnancy (Urine or serum HCG will be recorded)<br>8. Receiving or about to commence ECMO or HFOV<br>9. Myopathy, spinal cord injury, or nerve injury or disease with a likely prolonged incapacity to breathe independently e.g. Guillain-Barre syndrome<br>10. Usually receives home oxygen<br>11. Dependent on others for personal care due to physical or cognitive decline<br>12. Death is imminent or inevitable within 24 hours<br>13. The clinical team would not be able to set up the study nebuliser and ventilator circuit as required including with active humidification<br>14. Clinician objection.
Sample Size	This is a proof of principle / feasibility study aiming to evaluate the effect of nebulised unfractionated heparin on procoagulant markers related to acute respiratory distress syndrome in patients invasively ventilated for covid 19 lung disease. This has not been studied previously, and as such, data does not exist to power the study accurately to assess clinical outcomes. We have chosen to base our power analysis on some of our own in house data for d-dimer levels in covid patients who required ICU care and those who didn't. Based on a calculation that nebulised heparin may reduce the d-dimer levels in ICU patients to those experienced in ward patients, with an alpha level of 0.05 and a power of 90% to detect a type II error 19 patients per group would be required. Increasing the number by 1 per group to allow for potential dropout gives a sample size of 20 patients per arm of the study, with a total of 40 patients to be enrolled. The safety profile of administering nebulised heparin to invasively ventilated patients with COVID 19 will be a co-primary outcome, but power analysis will not be done for this outcome.
Study Treatment	Participants randomised to 'nebulised heparin' will receive nebulised heparin in addition to the standard care required as determined by the treating team.<br><br>Participants randomised to 'standard care' will receive the standard care required as determined by the treating team and will not be treated with nebulised heparin.

Treatment Duration	Nebulised heparin is administered 6-hourly from enrolment to day 10 post enrolment, provided the patient is receiving invasive mechanical ventilation. Data collection will be completed at day 60.
Study Assessments	Background Information<br><br>Medical History<br><br>Baseline assessment<br><br>Clinical records for the duration of the administration of nebulised heparin will be assessed for:<br><br>Haemodynamic and respiratory parameters and interventions, blood loss, requirement for red cell transfusion, coagulation and other haematology and biochemical parameters, duration of invasive mechanical ventilation, total dose of study medication, survival parameters at day 28, and day 60, functional assessments at day 28 and day 60.<br><br>Assessments of safety and records of adverse events
Follow Up Duration	Participants will be followed up until day 60 after enrolment.
Objective	To determine if treatment with nebulised heparin, compared to standard care, reduces the procoagulant response in ICU patients with SARS-CoV-2 requiring invasive mechanical ventilation and to assess the safety of this treatment.
Primary Outcome	Effect of nebulised heparin on d-dimer profile, assessed via d-dimer AUC and via a mixed effects model, with data collected on days 1, 3, 5 and 10.<br><br>Safety of nebulised heparin delivered by aerogen solo nebuliser in patients with COVID-19 induced severe respiratory failure, as measured by the incidence of severe adverse events.
Secondary Outcomes	- Determine the impact of nebulised heparin on oxygenation index<br><br>- Effect of nebulised heparin on other inflammatory (Interleukin (IL)-1β, IL-6, IL-8, IL-10 and soluble TNF receptor 1 (sTNFR1), C-reactive protein, procalcitonin, Ferritin,) and coagulation indices (Fibrinogen; lactate dehydrogenase) will be assessed (AUC on days 1, 3, 5 and 10) as part of an exploratory outcome to assess for any potential markers of COVID 19 ARDS. The ratios of IL-1β/IL-10 and IL-6/IL-10 will also be assessed.<br><br>- Determine the effect of nebulised heparin on pulmonary compliance measured on days 1,3,5,10.<br><br>- Time to separation from invasive ventilation to day 28, where non survivors to day 28 are treated as though not separated from invasive ventilation.<br><br>- Number treated with neuromuscular blockers instituted after enrolment to day 10

	- Number treated with prone positioning instituted after enrolment to day 10<br><br>- Number treated with extra-corporeal membrane oxygenation instituted after enrolment to day 10<br><br>- Number tracheotomised to day 28<br><br>- Time to separation from invasive ventilation to day 28, among survivors<br><br>- Time to separation from the ICU to day 28, where non-survivors to day 28 are treated as though not separated from invasive care<br><br>- Time to separation from the ICU to day 28, among survivors<br><br>During the pandemic critically ill inpatients might be cared for outside of the walls of the usual physical environment of ICU. For this reason, 'ICU' is defined as an area designated for inpatient care of the critically ill where therapies including invasive mechanical ventilation can be provided.<br><br>- Survival to day 28; Survival to day 60; and Survival to hospital discharge, censored at day 60<br><br>- Number residing at home or in a community setting at day 60<br><br>- Number residing at home or in a community setting at day 60, among survivors
Investigational Medicinal Product	Heparin sodium 25,000 Units in 5 mL is manufactured by Wockhardt UK Ltd. Ash Road North Wrexham LL13 9UF United Kingdom. This is a fully licenced product in Ireland, with the marketing authorisation PA1339/009/007, however it is not licenced for the planned use in this study. The medication will be stored at room temperature in a secure area and dispensed by pharmacists and physicians under the supervision of investigators.
Formulation and Dose	Heparin sodium 25,000 Units in 5 mL
Route of Administration	Heparin sodium will be administered via Aeroneb Solo (Aerogen Ltd) vibrating mesh nebuliser, placed in the inspiratory limb of the ventilator circuit.

Table of Contents

1. SPONSOR PROTOCOL AGREEMENT PAGE 1

2. INVESTIGATOR PROTOCOL AGREEMENTS 3

2.1. CHIEF INVESTIGATOR AGREEMENT 3

2.2 SITE INVESTIGATOR AGREEMENT 4

DOCUMENT HISTORY 5

ABBREVIATIONS 6

PROTOCOL SYNOPSIS 7

INTRODUCTION 15

1. BACKGROUND 15

2. OBJECTIVES AND OUTCOME MEASURES 19

2.1 RESEARCH HYPOTHESIS 19

2.2 STUDY OBJECTIVE 19

2.3 PRIMARY OUTCOME MEASURE 20

2.4SECONDARY OUTCOME MEASURES 20

2.6 PROCESS OF CARE ASSESSMENTS 21

3. TRIAL DESIGN 21

PARTICIPANT IDENTIFICATION, TRIAL PROCEDURES AND INTERVENTIONS 22

4. DESCRIPTION OF TRIAL SUBJECTS 22

5. RECRUITMENT 22

6. ELIGIBILITY CRITERIA 22

6.1 INCLUSION CRITERIA 22

6.2 EXCLUSION CRITERIA 23

7. PARTICIPANT TIMELINE 23

8. TRIAL PROCEDURES 23

8.1 SCREENING, NEXT OF KIN ASSENT (DAY <1) 23

8.2 Enrolment, and Randomisation, Baseline data (Day 1) 23
8.3 Daily data for days 0 to 10 while in intensive care 24
8.4 Day 28 25
8.5 Day 60 25
8.6 Adverse event or adverse reaction data 25
8.7 How and When Interventions will be Administered 25
8.7 Criteria for Modifying or Discontinuing Allocated Interventions 28
8.8 Concomitant Care 29
8.9 Definition of End of Trial 29
9. Investigational Medicinal Product 29
9.1 IMP Description 29
9.2 IMP Supply, Labelling, Storage, and Handling 30
9.3 IMP Accountability 30
.....

ASSIGNMENT OF INTERVENTIONS 31

10. Allocation 31
10.1 Sequence Generation 31
10.2 Allocation Concealment 31
10.3 Implementation 31
10.4 Procedures for Handling Incorrectly Enrolled or Randomised Participants 31

DATA COLLECTION MANAGEMENT AND ANALYSIS 31

11. Data Collection Methods 31
12. Data Management 32
12.1 Source Data 32
12.2 Data Quality Assurance 32
12.3Clinical Trial Documentation 33
12.4 Access to Data 33
12.5 Data Sharing 33
12.6 Data Recording and Record Keeping 33

13. STATISTICAL METHODS 33
13.1 DESCRIPTION OF STATISTICAL METHODS 33
13.2 ANALYSIS SETS 33
13.3 DEMOGRAPHIC AND BASELINE DISEASE CHARACTERISTICS 34
13.4 EFFECTIVENESS ANALYSIS 34
13.5 THE LEVEL OF STATISTICAL SIGNIFICANCE 35
13.6 PROCEDURE FOR ACCOUNTING FOR MISSING, UNUSED AND SPURIOUS DATA 36
13.7 SAMPLE SIZE 36
13.8 INTERIM ANALYSES 36
13.9 CRITERIA FOR ENDING THE TRIAL 36
13.10 CRITERIA FOR THE TERMINATION OF THE TRIAL 36
13.11 INCLUSION IN ANALYSIS 37
13.12 PROCEDURES FOR REPORTING ANY DEVIATION(S) FROM THE ORIGINAL STATISTICAL PLAN 37
14. SAFETY REPORTING 37
14.1 DEFINITIONS 38
14.2 CAUSALITY 39
14.3 ASSESSMENT OF SEVERITY 39
14.4 ASSESSMENT OF EXPECTEDNESS 40
14.5 RELATION TO STUDY IMP 40
14.6 PROCEDURES FOR RECORDING ADVERSE EVENTS 40
14.7 PROCEDURES FOR RECORDING AND REPORTING SERIOUS ADVERSE EVENTS 41
14.8 SUSARS 41
14.9 PROCEDURES FOR DOCUMENTING AND REPORTING PREGNANCIES 41
14.10 DATA SAFETY MONITORING COMMITTEE 41
QUALITY ASSURANCE PROCEDURES 42
15.1 TRAINING OF STUDY SITE PERSONNEL 42
15.2 MONITORING AND AUDIT 42
16. SERIOUS BREACHES 43
ETHICS AND REGULATORY 43

17. ETHICAL AND REGULATORY CONSIDERATIONS 43
17.1 DECLARATION OF HELSINKI 43
17.2 GUIDELINES FOR GOOD CLINICAL PRACTICE 43
17.3 APPROVALS 44
17.4 REPORTING 44
18. PROTOCOL AMENDMENTS 44
19. CONSENT 44
20. PARTICIPANT CONFIDENTIALITY AND DATA PROTECTION 46
20.1 CONFIDENTIALITY OF STUDY DATA 47
21. DECLARATION OF INTERESTS 47
22. POST-TRIAL CARE 47
23 FINANCE AND INSURANCE 47
23.1 FUNDING AND INSURANCE 47
23.2 PARTICIPANT REIMBURSEMENT 47
24. PUBLICATION POLICY 47
REFERENCES 49

INTRODUCTION

1. Background

Under the sponsorship of National University of Ireland Galway, we present a randomised open label phase Ib/IIa trial of nebulised unfractionated heparin to evaluate the effect of nebulised unfractionated heparin on the procoagulant response in ICU patients with SARS-CoV-2 requiring invasive mechanical ventilation. As this is the first study of nebulised heparin in COVID 19 lung disease we will assess safety as a co-primary outcome.

Summary

The search for an effective treatment for COVID-19 is underway around the world. We outline why a trial of nebulised heparin is warranted.

A recent (not yet published; under journal review) pre-pandemic double-blind multi-centre randomised study of 256 mechanically ventilated patients with or at risk of developing ARDS led by our group found, among pre-specified secondary outcomes, that both deterioration in the Murray Lung Injury Score and the development of ARDS in those at risk were significantly reduced with nebulised heparin and that the proportion of day 60 survivors residing at home was significantly increased with nebulised heparin. There was no evidence of harm.

COVID-19 is associated with the development of ARDS displaying the typical features of diffuse alveolar damage with extensive pulmonary coagulation activation resulting in fibrin deposition in the microvasculature and formation of hyaline membranes in the air sacs. The anticoagulant actions of nebulised heparin limit fibrin deposition. Serendipitously, unfractionated heparin also inactivates the SARS-CoV-2 virus and prevents its entry into mammalian cells. Nebulisation of heparin may therefore limit fibrin-mediated lung injury and inhibit pulmonary infection by SARS-CoV-2. For these reasons we believe a trial of nebulised heparin in patients with COVID-19 is warranted.

Morbidity and mortality of ARDS and COVID-19

ARDS affects 23% of mechanically ventilated critically ill patients and has a mortality up to 46%.[1,2] The death rate in COVID-19 patients with ARDS may be higher, up to 66%.[3] Survivors of ARDS have marked limitations in physical function with increased need for rehabilitation and long-term care.[4,5]

Histological features of ARDS

The clinical syndrome of ARDS due to acute inflammatory changes in the lungs was first described in a 12-patient case series by Ashbaugh et al in 1967.[6] The histological changes found by Ashbaugh have been termed *diffuse alveolar damage*.[7] The hallmark histological feature of ARDS is a fibrin mesh in the air sacs, known as a hyaline membrane, on which leucocytes attach and manifest the inflammatory responses that result in diffuse alveolar damage.[7-9] Fibrin accumulation in pulmonary capillaries and venules, which leads to microvascular thrombosis is another early feature,[10-12] and the extent of this fibrin accumulation correlates with the severity of acute lung injury.[11,13] Many conditions can trigger an inflammatory response that results in diffuse alveolar damage including pneumonia, sepsis, aspiration, transfusion, cardiac surgery and trauma.[14]

Pulmonary microvascular thrombosis

In response to inflammatory cytokines the pulmonary capillary beds, venules and arterioles express tissue factor on endothelial cells and this triggers the conversion of plasma coagulation factors to

fibrin.[15] Extensive microvascular thrombosis has been demonstrated in histological studies of ARDS.[10,11] Angiographic studies showed the extent of microvascular obstruction correlated with the severity of respiratory failure and with mortality.[11,13] Microvascular thrombosis increases lung dead space and the increase in the dead space was shown to be an independent marker of mortality in ARDS.[16] Microvascular thrombosis also causes increased pulmonary vascular resistance, which may result in right heart failure.[17] A recent study (not yet published) which analysed the d-dimer response in COVID 19 ARDS showed a higher mortality in patients with d-dimers higher than the median in the study. (Paper attached) Our own data in UHG showed a far higher d-dimer level in patients admitted to ICU compared to those not requiring ICU admission, which was maintained over time, consistent with thrombosis.

Hyaline membrane formation

Hyaline membrane formation is a consistent and early manifestation of the inflammatory response in ARDS.[6,10,18-20] Hyaline membrane formation results from entry into the alveolar space of inflammatory exudate that is rich in plasma borne coagulation factors. The subsequent expression of tissue factor by alveolar epithelial cells and macrophages triggers the conversion of these coagulation factors to fibrin and the formation of the hyaline membrane. The concurrent expression of plasminogen activator inhibitor-1 by alveolar macrophages prevents the removal of this membrane through endogenous fibrinolysis.[8]

Hyaline membrane formation may contribute to lung injury through a number of mechanisms. Fibrin is a pro-inflammatory molecule attracting leucocytes which attach to fibrin promoting further inflammatory responses that results in diffuse alveolar damage.[7-9] The hyaline membrane forms a physical barrier thereby limiting the diffusion of gases. Alveolar compliance and the action of surfactant is also limited contributing to atelectasis. Finally, the laying down of a fibrin matrix may promote subsequent lung fibrosis.[8,21]

SARS-CoV-2 is associated with increased inflammatory cytokines and excessive coagulation activation and fibrin deposition in the lungs and other organs

Patients with SARS-CoV-2 who manifest severe disease including ARDS, multi-organ failure and death, have higher levels of inflammatory cytokines ("cytokine storm"), higher plasma markers of coagulation, such as D-Dimers, increased prothrombin time and a lower platelet count.[3,22-25]

Post-mortem studies and lung biopsies of SARS-CoV-2 patients with ARDS demonstrated pulmonary fibrin deposition with hyaline membranes in the alveolar spaces and extensive pulmonary microvascular thrombi (Figure 1).[26-31] Microvascular thrombi were also found in other organs including the heart, liver and kidneys.[29,31-33]

Figure 1. Pathogenic changes in COVID-19

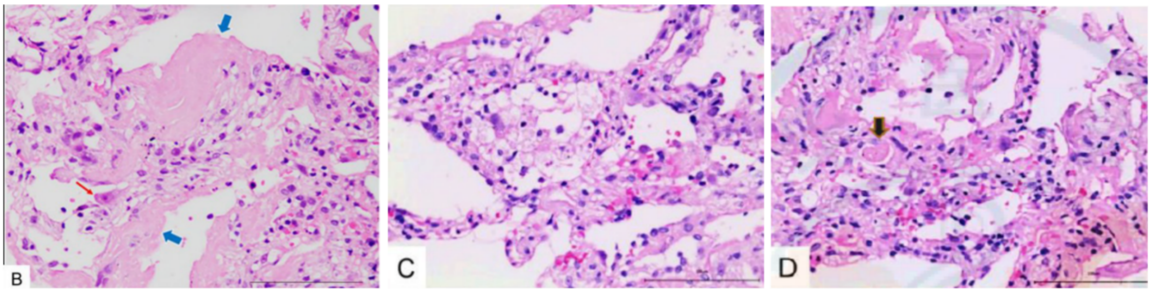

Hyaline membrane formation（blue arrow) | Interstitial mononuclear inflammatory infiltrates | Thrombus in pulmonary arterioles (black arrow)

Xiaohong Yao et al. Chinese Journal of Pathology. 2020,49 (2020-03-15).

4

Treatment of pulmonary microvascular thrombosis and hyaline membranes

Nebulised heparin targets pulmonary fibrin deposition. Heparin's anti-coagulant properties have been used in clinical practice to limit fibrin deposition since 1935. Heparin inhibits coagulation activation through a range of mechanisms, including catalysing the action of antithrombin, promoting tissue factor pathway inhibitor expression, reducing tissue factor expression, increasing endothelial expression of heparan sulphate and through release of t-PA by the endothelium.[34-37] Heparin also has other actions of potential benefit including inhibition of inflammatory cytokines, prevention of bronchospasm, increased nitric oxide release and limiting adhesion of microbes to respiratory epithelium.[38-41]

Early-phase trials in patients with acute lung injury and related conditions found heparin reduced pulmonary dead space, coagulation activation, microvascular thrombosis and deterioration in the Murray Lung Injury Score and increased time free of ventilatory support.[42-47]

A recent multi-centre randomised double-blind placebo-controlled trial of nebulised heparin in patients with or at risk of developing Acute Respiratory Distress Syndrome, to determine if nebulised heparin improves long term physical function (ANZCTR, 12612000418875), found that amongst 256 mechanically ventilated patients, 47% of whom had ARDS, the Murray Lung Injury Score and development of ARDS were both significantly reduced with nebulised heparin and the proportion of day 60 survivors residing at home was significantly increased with nebulised heparin. There was no evidence of harm. These results indicate that nebulised heparin limits pulmonary fibrin deposition, attenuates progression of acute lung injury and hastens recovery.

Heparin administration may be associated with improved clinical outcomes in SARS-CoV-2

Anecdotal data suggest patients with SARS-CoV-2 treated with systemic unfractionated heparin or LMWH had better clinical outcomes.[48] A non-randomised study found patients with a sepsis-induced coagulation score greater than 4 were more likely to survive if administered heparin or LMWH.[25]

SARS-CoV-2 inactivation by heparin

Heparin is a member of a family of proteoglycan molecules that include heparan sulphate, chondroitin sulphate, keratan sulphate and hyaluronic acid. These molecules are expressed throughout the body, with diverse biological roles and are usually associated with respiratory and endothelial cell surfaces, basement membrane and extracellular matrices.[49] In humans heparin is produced solely by mast cells and is stored in granules. Heparin makes up 30% of the dry weight of mast cell granules.[50]

There is evidence that heparin plays a role in host defence. Firstly, mast cells are mostly located along blood vessels and are particularly associated with capillaries and post-capillary venules.[51] Secondly, organs exposed to the external environment, such as the lungs and gut contain a large proportion of the body's mast cells.[52] Thirdly, heparin is conserved across a variety of different species, some of which do not have a blood coagulation system like ours (e.g. molluscs), suggesting heparin has significant biological roles unrelated to coagulation.[53] Studies in humans have also demonstrated that heparin has a complex range of biological actions independent of coagulation, some of which may also be beneficial in limiting lung damage associated with critical illness.

A large number of bacterial and viral pathogens depend upon interactions with proteoglycan molecules such as heparan sulphate, which is expressed on a range of human tissue surfaces, for adhesion and invasion of host tissues.[49] A number of studies found heparin competes with heparan sulphate for bacterial and viral adhesion and may therefore limit pathogen invasion.[54,55] Heparin limits adhesion of a number of pathogens including Pseudomonas aeruginosa, Burkholderia cenocepacia, Burkholderia pseudomallei, Legionella pneumophila, Staph aureus, Strep pyogenes, Strep pneumonia, Respiratory syncytial virus and Influenza A.[41,56-59] Human and animal studies suggest these actions may reduce the development of pneumonia and bacteraemia.[54,60]

Unfractionated heparin binds SARS-CoV-2 and prevents cell invasion by Coronavirus

A recent study demonstrated that the SARS-CoV-2 Spike S1 protein receptor binding domain attaches to unfractionated heparin and undergoes conformational change as a result.[61] Previous studies demonstrated that unfractionated heparin prevented SARS-associated coronavirus and other coronavirus strains from invading mammalian cells.[62-67]

Safety and tolerability of nebulised heparin

APTT. Treatment with nebulised heparin at the dose proposed for this study, while concomitantly receiving intravenous or subcutaneous unfractionated heparin, is likely to cause a mild (approximately 7 seconds) increase in the average APTT and a mild-moderate (approximately 18 seconds) increase in the peak APTT during the treatment period. The APTT of patients who are not receiving intravenous or subcutaneous unfractionated heparin is likely to be not affected by treatment with nebulised heparin.[68]

Bleeding and blood transfusion. Nebulised heparin at the dose proposed in this study has not been found to increase the risk of major non-pulmonary bleeding or of blood transfusion.[68] In clinical trials where more than 150 intensive care patients were treated with nebulised heparin for ARDS and related conditions, there were no cases of pulmonary haemorrhage with patient deterioration.[42,46,68,69] Bloodstaining of the airway secretions of invasively ventilated intensive care unit patients is common. Approximately 1 out of 20 patients treated with nebulised heparin experience greater-than-usual bloodstaining of secretions and, although this bloodstaining can be unsightly, rarely is it clinically deleterious. The risk of medically important haemoptysis is estimated to be less than 1 out of 100.

Use in asthma and COPD. Use in asthma and COPD is typically well-tolerated but, on rare occasions in patients with severe bronchospasm, airflow may be further impeded immediately following the start of nebulisation.[68] This may represent heparin accumulating on the luminal surface of terminal bronchioles, causing further narrowing. This typically resolves within minutes of stopping nebulisation and withholding the remainder of the dose. Subsequent doses are typically well-tolerated, but vigilance is required.

Heparin-induced thrombocytopenia (HIT). HIT is an immune-mediated adverse reaction caused by the emergence of antibodies that activate platelets in the presence of heparin. Patients in intensive care are commonly treated with subcutaneous heparin for the prevention of deep vein thrombosis. The risk of HIT from treatment with subcutaneous unfractionated is less than 1 out of 100. Nebulised heparin is not thought to increase the risk of HIT in patients already receiving unfractionated heparin and administration by nebulisation is not thought to increase the risk of HIT compared to administration by other routes and no patient to date in our earlier trials developed HIT.[42,46,68,69]

Clinician exposure to SARS-CoV-2. Administering nebulised heparin will not significantly increase the risk of SARS-CoV-2 exposure for clinicians who follow the standard recommended personal protection precautions for the care of confirmed or suspected COVID-19 cases. The Aerogen vibrating mesh membrane system has an in-line circuit design so the ventilation circuit does not need to be broken for drug delivery and is designed so that the medication reservoir is isolated from the breathing circuit and is positioned in the inspiratory limb of the ventilator circuit on the 'dry' side of the humidifier, remote from the patient's endotracheal tube. Once the nebuliser is in position it does not need to be removed until the course of treatment is finished.[70] An expiratory filter is positioned at the end of the expiratory limb of the ventilator circuit. The main purpose of the filter is to prevent exhaled heparin from depositing on the ventilator's expiratory sensors and valves, but it is also a highly efficient viral filter that will mitigate against entry of exhaled virus into the room.[71]

Conclusion

In view of the well-conducted clinical trials in patients with ARDS and related conditions, which found that nebulised heparin limits pulmonary fibrin deposition, attenuates progression of acute lung injury, hastens recovery and is safe, and with the knowledge that heparin inactivates SARS-CoV-2, we believe a feasibility study to determine whether or not nebulised heparin is beneficial in the treatment of COVID-19 is warranted.

2. Objectives and Outcome Measures

2.1 Research Hypothesis

Our hypothesis is that nebulised heparin will decrease severity of lung injury in COVID 19 through multiple mechanisms as shown above, and this will be shown through the decrease in pro-coagulantmarkers such as d-dimer. We intend to assess safety outcomes related to the administration of nebulised heparin as a co-primary outcome. We will also assess clinical outcomes such as oxygenation index, time to liberation from invasive mechanical ventilation in patients with COVID-19 lung disease, and functional outcomes at day 28 and 60 as secondary outcomes.

2.2 Study Objective

The objective of this feasibility study is to determine if treatment with **nebulised heparin**, compared to standard care, reduces the severity of the procoagulant response in ICU patients with SARS-CoV-2 requiring invasive mechanical ventilation.

To assess safety of nebulised heparin in patients with COVID-19 induced severe respiratory failure

2.3 Primary Outcome Measure

Effect of **nebulised heparin** on D-dimer profile (assessed via D-dimer AUC on days 1, 3, 5 and 10). Blood samples for analysis of d-dimer will be taken once every 24 hours on day 1, 3, 5 and 10.

Safety of nebulised heparin in patients with COVID-19 induced severe respiratory failure as measured by number who record severe adverse events.

2.4 Secondary Outcome Measures

- To determine the impact **nebulised heparin** on oxygenation index (OI)
  - OI will be recorded every 6 hours.
  - (OI= (FiO_2 x M_{PAW}) / PaO_2 where OI = Oxygenation index, FiO_2 = Fraction of inspired oxygen (percent), M_{PAW} = Mean airway pressure and PaO_2 = Partial pressure of oxygen in arterial blood.
- To determine the effect of nebulised heparin on pulmonary compliance measured on days 1,3,5 and 10.
- Effect of nebulised heparin on other inflammatory (Interleukin (IL)-1β, IL6, IL-8, IL-10 and soluble TNF receptor 1 (sTNFR1), C-reactive protein, procalcitonin, Ferritin,) and coagulation indices (Fibrinogen; lactate dehydrogenase) will be assessed (AUC on days 1, 3, 5 and 10) to assess for any potential markers of COVID 19 ARDS. The ratios of IL-1β/IL-10 and IL-6/IL-10 will also be assessed.
- Time to separation from invasive ventilation to day 28, where non-survivors to day 28 are treated as though not separated from invasive ventilation. ('Invasive ventilation' means any positive pressure ventilatory support via an endotracheal or tracheostomy tube. If a patient achieves separation from invasive ventilation more than once, it is the final separation that is used to calculate the outcome.)
- In this study, 'day 0' describes the period from randomisation to midnight on the day of enrolment, 'day 1' the first calendar day after the day of enrolment, 'day 2' the second calendar day after the day of enrolment, and so forth.
- Number treated with neuromuscular blockers instituted after enrolment to day 10
  - Any administration of neuromuscular blocking drugs will be recorded
- Number treated with prone positioning instituted after enrolment to day 10
- Number treated with extra-corporeal membrane oxygenation instituted after enrolment to day 10
- Number tracheotomised to day 28
- Time to separation from invasive ventilation to day 28, among survivors
- Time to separation from the ICU to day 28, where non-survivors to day 28 are treated as though not separated from invasive care
- Time to separation from the ICU to day 28, among survivors
- During the pandemic critically ill inpatients might be cared for outside of the walls of the usual physical environment of ICU. For this reason, 'ICU' is defined as an area designated for inpatient care of the critically ill where therapies including invasive mechanical ventilation can be provided.
- Survival to day 28; Survival to day 60; and Survival to hospital discharge, censored at day 60
- Number residing at home or in a community setting at day 60

- Number residing at home or in a community setting at day 60, among survivors

2.6 Process of care assessments

Process of care assessments are as follows:

- Time from intubation to randomisation

- Total cumulative dose of nebulised heparin in ICU to day 10

- Days of treatment with nebulised heparin in ICU to day 10

- Mean APTT in ICU to day 10 among all participants, among those treated with intravenous or subcutaneous unfractionated heparin, and among those not treated with intravenous or subcutaneous unfractionated heparin

- Highest APTT in ICU to day 10 among all participants, among those treated with intravenous or subcutaneous unfractionated heparin, and among those not treated with intravenous or subcutaneous unfractionated heparin

- Days of treatment with each of the following therapies while in ICU to day 10: unfractionated heparin, intravenous and subcutaneous; LMWH, intravenous and subcutaneous; lopinavir-ritonavir; remdesivir; hydroxychloroquine; interferon-β; interleukin antagonists; oseltamivir, laninamivir, zaninamivir or peramivir; macrolide; non-macrolide antibacterial; antifungal; corticosteroid; inotrope or vasopressor infusion; and renal replacement.

2.7 Safety assessments

The incidence of the listed safety outcomes will be recorded for each group

1. Air samples will be taken in the vicinity of the nebuliser and endotracheal tube to assess for any leak of infective particles related to the use of the aeroneb solo nebuliser and nebulised heparin.
2. Number transfused red blood cells (packed red cells and whole blood) to day 10
3. Volume of red blood cells (packed red cells and whole blood) transfused to day 10
4. Number who record major bleeding
5. 'Major bleeding' is defined as: bleeding that results in death and/or; bleeding that is symptomatic and occurs in a critical area or organ (intra-cranial, intra-spinal, intra-ocular, retroperitoneal, intra-articular, or intramuscular with compartment syndrome) and /or; bleeding that results in a fall in haemoglobin of 20g/L or more, or results in transfusion of two or more units of whole blood or red cells.
6. Number who record heparin-induced thrombocytopaenia (HIT)
    a. 'HIT' is an unexplained fall in platelet count and a positive heparin antibody test.
7. Number who record other adverse events and reactions

3. Trial Design

The CHARTER Study has been designed with reference to the SPIRIT checklist. It is a multicentre, international, phase II, randomised, parallel group, open label feasibility study. The study is designed

to evaluate whether unfractionated heparin, administered 6-hourly to day 10 while the patient is receiving invasive mechanical ventilation, will decrease the procoagulant state related to ARDS measured to day 10. The study will measure safety outcomes for the administration of heparin via this device as a co-primary endpoint, and will assess clinical outcomes as secondary endpoints.

The study will randomise patients via 1:1 allocation to usual care plus nebulised unfractionated heparin 6 hourly to day 10 while mechanically ventilated or usual care only.

PARTICIPANT IDENTIFICATION, TRIAL PROCEDURES AND INTERVENTIONS

4. Description of trial subjects

Selected intensive care units in Ireland where patients with COVID 19 are being treated. The rate of enrolment from each centre will be dependent on the underlying infection rates for COVID 19. If the rate of infection drops to a very low level, other international sites may be added.

5. Recruitment

Recruitment will be organised and supervised by intensive care research and clinical research facilities within recruiting centres and in line with national and international standards and guidelines, and local SOPs.

The Research Nurses/Coordinators and Investigators at each site will work with clinicians to identify potential candidates for enrolment. A screening log will be maintained of patients who met the inclusion criteria but were not enrolled, with the reason for exclusion recorded on the log. If a patient is deemed eligible, the patient's next of kin will be contacted, and a patient information leaflet will be sent to them or given to them in person if possible. A system of deferred informed patient consent and legal guardian / next of kin assent will be necessary for this study as patients will not have capacity to consent during the critical phase of their illness in the Intensive Care Unit, refer to section 19 for the consent process.

Should the relative providing assent decide to withdraw that assent, or the patient request to withdraw the assent from the study then the patient will be immediately withdrawn from the study and no further information will be collected.

6. Eligibility Criteria

6.1 Inclusion Criteria

To be eligible, a patient must satisfy all these inclusion criteria:

1. Confirmed or suspected COVID-19

If 'suspected', results must be pending or testing intended

2. Age 18 years or older
3. Endotracheal tube in place
4. Intubated yesterday or today
5. PaO2 to FIO2 ratio less than or equal to 300 while intubated
6. Acute opacities on chest imaging affecting at least one lung quadrant
7. 'Acute opacities' do not include effusions, lobar/lung collapse or nodules

8. Currently in the ICU or scheduled for transfer to the ICU
   The ‘ICU’ is an area designated for inpatient care of the critically ill where therapies including invasive mechanical ventilation can be provided.

6.2 Exclusion Criteria

To be eligible, a patient must have none of these exclusion criteria:

1. Enrolled in another clinical trial that is unapproved for co-enrolment
2. Heparin allergy or heparin-induced thrombocytopenia
3. APTT > 120 seconds and this is not due to anticoagulant therapy
4. Platelet count < 20 x 109 per L
5. Pulmonary bleeding, which is frank bleeding in the trachea, bronchi or lungs with repeated haemoptysis or requiring repeated suctioning
6. Uncontrolled bleeding
7. Pregnant or suspected pregnancy (Urine or serum HCG will be recorded)
8. Receiving or about to commence ECMO or HFOV
9. Myopathy, spinal cord injury, or nerve injury or disease with a likely prolonged incapacity to breathe independently e.g. Guillain-Barre syndrome
10. Usually receives home oxygen
11. Dependent on others for personal care due to physical or cognitive decline
12. Death is imminent or inevitable within 24 hours
13. The clinical team would not be able to set up the study nebuliser and ventilator circuit as required including with active humidification
14. Clinician objection.

7. Participant Timeline

Patients enrolled in this study will be incapable of providing consent at the time of enrolment, by virtue of the fact that they will be invasively ventilated. As a result, standard informed consent procedures cannot be followed. Given the importance of this process, we have referred to the consent process implemented for the WHO Solidarity trial which will enrol similar patients in some circumstances. This is detailed below and in section 19. This process has also been approved by the NREC and the Health Research Consent Declaration Committee.

8. Trial Procedures

8.1 Screening, next of kin assent (Day <1)

Research coordinators and investigators at each site will work with clinicians to identify potential candidates for enrolment. A screening log will be maintained of patients who met the inclusion criteria but were not enrolled, with the reason for exclusion recorded on the log.

The assent/consent process will be completed as detailed in section 19.

8.2 Enrolment, and Randomisation, Baseline data (Day 1)

Following documented next of kin assent, the patient will be enrolled in the study and randomised. At randomisation each participant is assigned to nebulised heparin or standard care. There is a one to one allocation ratio. Allocation concealment will be maintained by use of a central, secure web randomisation process. Blocks of variable size and a random seed will be used to ensure allocation concealment cannot be violated by deciphering the sequence near the end of each block. To further protect from deciphering, block size will not be revealed to site Investigators. Randomisation is stratified by site.

The following baseline data will be recorded on the baseline eCRF:

- Eligibility criteria

- Intubation date and time; and intubation setting

- Birth date; sex; height; and weight

- History of tobacco smoking; hypertension; diabetes mellitus; asthma; and COPD

- Hospital admission date; Intensive care admission date and time; and APACHE III ICU diagnosis

-Treatment in the 24 hours prior to randomisation with unfractionated heparin, intravenously or subcutaneously; LMWH, intravenously or subcutaneously; lopinavir-ritonavir; remdesivir; hydroxychloroquine; interferon-β; interleukin antagonists; oseltamivir, laninamivir, zaninamivir or peramivir; macrolide; non-macrolide antibacterial; antifungal; and corticosteroid

- Treatment at the time of randomisation with inotrope or vasopressor infusion; renal replacement therapy; neuromuscular blocker; and prone positioning

- Serum creatinine and bilirubin, blood haemoglobin, white cell count and platelet count, and blood APTT and INR, collected closest to and before randomisation

- For the chest radiograph (or chest CT) performed closest to and before randomisation: the number of lung quadrants with acute opacities that are not fully explained by effusions, lobar/lung collapse or nodules; whether the opacities are present bilaterally; whether, given all the medical information about the patient, the opacities are entirely attributable to cardiac failure or fluid overload; whether the patient was exposed to any risk factor for acute lung injury in the previous 7 days and; whether there is objective evidence to exclude the possibility of cardiac failure or fluid overload

- Arterial blood gas (pH, $PaCO_2$, PaO_2, bicarbonate and lactate) and corresponding ventilation parameters (respiratory rate, FIO_2, peak airway pressure, PEEP or CPAP and tidal volume) closest to and before randomisation.

8.3 Daily data for days 0 to 10 while in intensive care

The following data are gathered from health records at the treating institution for each day that the patient is in ICU from randomization (Day 0) up to and including day 10:

- Dose of nebulised unfractionated heparin

-Treatment with unfractionated heparin, intravenously or subcutaneously; LMWH, intravenously or subcutaneously; lopinavir-ritonavir; remdesivir; hydroxychloroquine; interferon-β; interleukin antagonists; oseltamivir, laninamivir, zaninamivir or peramivir; macrolide; non-macrolide antibacterial; antifungal; corticosteroid; inotrope or vasopressor infusion; renal replacement therapy; neuromuscular blocker; prone positioning; and extra-corporeal membrane oxygenation

- Volume of packed red cells and whole blood transfused

- Highest APTT

- Blood samples will be collected for inflammatory marker and coagulation parameter assessment as per the primary and secondary outcomes, on days 0, 3, 5 and 10.

8.4 Day 28

The following data are gathered from health records at the treating institution and, where necessary, other healthcare providers:

- Date of first diagnosis of COVID 19 (Clinical diagnosis)

- Date of first positive respiratory sample for SARS-CoV-2 and the sample type

- Acute hospital discharge status at day 28 and, if discharged, the date of discharge and discharge destination including deceased

- Readmission to ICU during the acute hospital admission and prior to day 28

- ICU status at day 28 and, if not in the ICU at the end of day 28, the final date and time of ICU discharge

- Invasive ventilation status at day 28 and, if not receiving invasive ventilation at the end of day 28, the final date and time that invasive ventilation was stopped

- Tracheotomy and, if performed, the procedure date

8.5 Day 60

The following data are gathered from health records at the recruiting institution and, where necessary, other healthcare providers and the patient or proxy:

- Vital status at day 60 and, if deceased, the date of death

- Place of residence at day 60

- If not discharged from the acute hospital discharge status by day 28, the acute hospital discharge status at day 60 is ascertained and, if now discharged, the date of discharge and the discharge destination including whether deceased.

8.6 Adverse event or adverse reaction data

Adverse event or adverse reaction data are gathered from health records at the recruiting institution and, where necessary, other healthcare providers and the patient or proxy.

The following information is ascertained: the type of event or reaction, including with regard to pre-defined events (major bleeding, pulmonary bleeding and HIT); date and time of onset; date and time of most recent administration of nebulised heparin; the extent of any causal link to the study; the significance/severity of the event; action taken regarding the further use of nebulised heparin; and a freeform summary.

8.7 How and When Interventions will be Administered

Receipt of allocated treatment

Participants assigned to ‘nebulised heparin’ will receive nebulised heparin in addition to the standard care required as determined by the treating team.

Participants assigned to ‘standard care’ will receive the standard care required as determined by the treating team and will <u>not</u> be treated with nebulised heparin.

Administering nebulised heparin

Supervision: Investigators or experienced research coordinators will be available to assist clinicians. Correct set-up of the nebuliser and ventilator circuit is essential to ensure the drug is administered correctly.

Set-up of the nebuliser, ventilator circuit and expiratory filter is shown in Figure 1 on the next page.

Active humidification: Active ventilator circuit humidification system will be used.

Nebuliser type and position: The *Aeroneb Solo* (Aerogen Ltd) vibrating mesh nebuliser is placed in the inspiratory limb of the ventilator circuit on the 'dry' side of the humidifier water bath. The nebuliser chamber must be upright. An Aerogen nebuliser control module that is external to the ventilator may be used or, if the ventilator has an internal Aerogen controller, that is also acceptable.

Expiratory filter: A *Servo Duo Guard* (Maquet Critical Care AB), or an equivalent filter, is placed between the expiratory limb of the circuit and the expiratory valve of the ventilator. The *Servo Duo Guard* is changed after 48 hours. The primary purpose of the filter is to prevent exhaled nebulised heparin from depositing on the expiratory sensors and valves of the ventilator, but it is also a highly efficient viral filter that will mitigate against entry of exhaled virus into the room.

Prescription: The nebulised heparin will be prescribed on the patient's medication administration record by a treating physician. The prescription will specify the drug (heparin sodium), the dose (25,000 Units in 5 mL), the frequency (6-hourly), the route (nebulised inhalation), the duration (10 days) and the indication (while receiving invasive ventilation in intensive care). DO <u>NOT</u> GIVE IV or SC.

Instilling the drug: Each dose of nebulised heparin should be instilled into the nebuliser chamber using a sterile, single-use 5 mL syringe. The nebuliser chamber must be upright during nebulisation.

Misting: The ventilator circuit should be inspected during nebulisation to verify that the medication is being delivered: it should be seen 'misting' in the circuit adjacent to the nebuliser chamber.

Respiratory cycle, dose duration: Nebulisation of each dose should occur throughout the respiratory cycle, rather than timed to nebulise with inspiration (a facility available with some Aerogen nebuliser control modules), and it should be complete within approximately 15 minutes.

Nebulising standard medications: The nebuliser used for heparin may also be used to administer preparations of salbutamol, ipratropium and budesonide that are approved for use by nebulisation.

Standard nebulised drugs should be scheduled so that the administration times of these drugs do not coincide with those of heparin.

Heparin should never be mixed with another drug or administered concurrently. Prior to instilling the heparin in the nebuliser chamber, the chamber must be inspected to ensure that the chamber is empty: drugs should never be mixed in the nebuliser chamber.

The nebuliser is very efficient and may deliver larger doses of standard nebulised drugs to the lungs compared to other nebulisers that are familiar to clinicians. The patient's response to standard nebulised medications should be carefully monitored, especially if the recommended dose is exceeded.

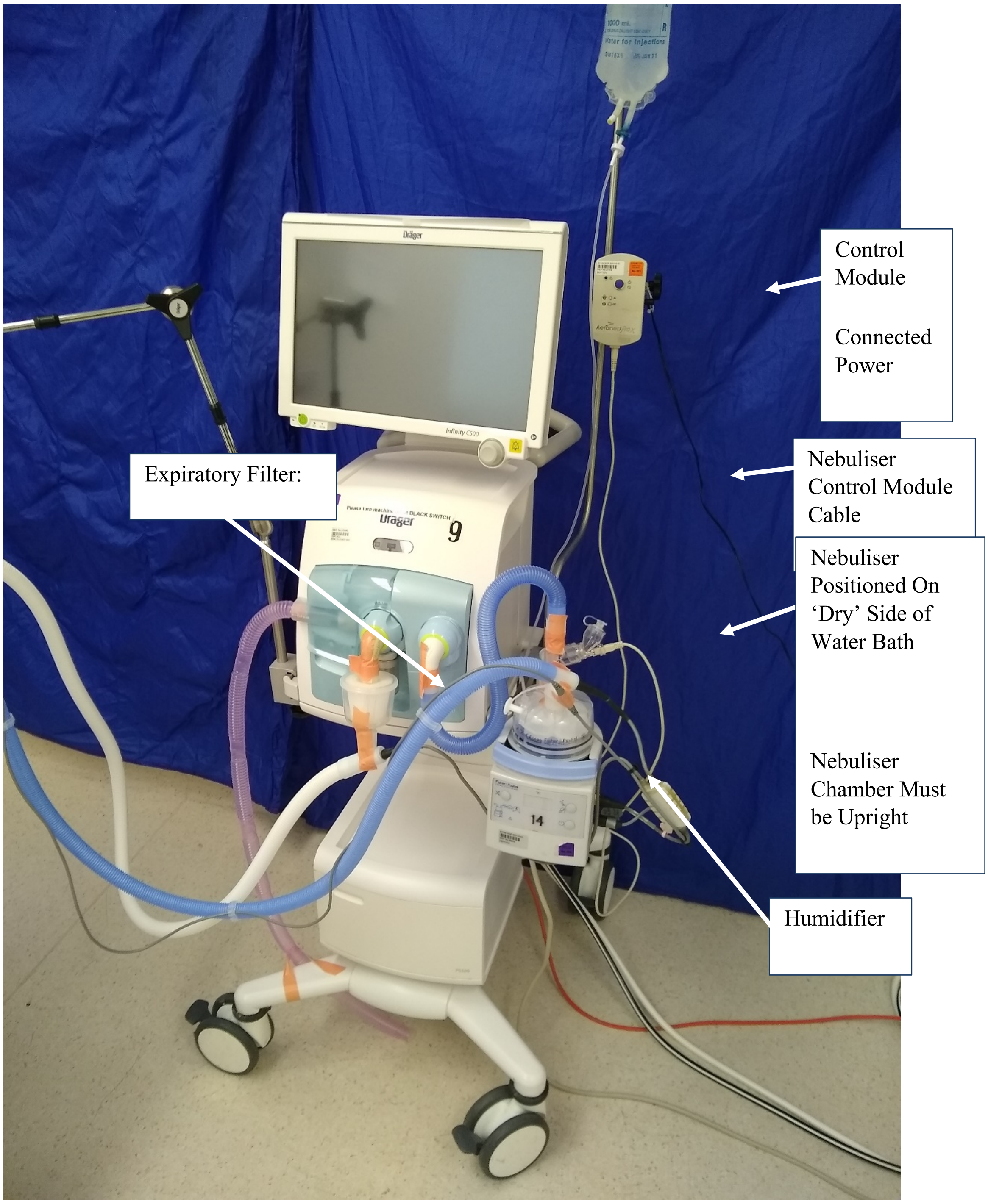

Figure 1. Ventilator circuit and nebuliser set-up

8.7 Criteria for Modifying or Discontinuing Allocated Interventions

Withholding nebulised heparin

Treatment with any or all of the following therapies is not of itself reason to withhold nebulised heparin: deep vein thrombosis prophylaxis with unfractionated heparin or LMWH; ‘full’ therapeutic dose unfractionated heparin or LMWH; non-heparin anticoagulants; anti-thrombotic medications; protamine; prone positioning; and inhaled nitric oxide.

Nebulised heparin should be withheld if any of the following occurs:

- More than 10 days have elapsed since randomisation
- The patient is outside of ICU
- The patient is not receiving invasive ventilation
- The treating physician deems that there is a clinically unacceptable increase in APTT
- The treating physician deems that there is excessive bloodstaining of respiratory secretions
- There is pulmonary bleeding, major bleeding or suspected or confirmed HIT
- The patient is receiving ECMO or HFOV.

Nebulised heparin should be recommenced if:

- Having been withheld because the patient was outside the ICU, the patient returns to ICU
- Having been withheld because the patient was not invasively ventilated, invasive ventilation is reinstituted
- Having been withheld because the APTT was unacceptably prolonged, the APTT becomes acceptable
- Having been withheld because there was excessive bloodstaining of respiratory secretions, the bloodstaining of the respiratory secretions has resolved
- Having been withheld for pulmonary bleeding or major bleeding, the bleeding is definitively controlled
- Having been withheld for suspected HIT, the patient is found not to have this condition
- Having been withheld for ECMO, the treatment with ECMO is stopped
- Having been withheld for HFOV, the treatment with HFOV is stopped.

Each participant has the right to withdraw from the trial at any time. A participant that decides to discontinue will always be asked about the reason(s) and the presence of any adverse events but are not required to provide a reason for their decision to withdraw. In addition, the Investigator may discontinue a participant from the trial at any time if the Investigator considers it necessary for any reason including:

Development of an adverse event that requires discontinuation of study treatment

Development of a contraindication to study treatment

The reason for withdrawal if given by the participant will be recorded. If withdrawal from or discontinuation of the study is due to an adverse event, the Investigator will arrange follow-up visits and/or telephone calls until the adverse event has resolved or stabilised. The participant will be encouraged to participate in this follow up; however, it is recognised that this is not mandated after withdrawal of consent. The data of all eligible participants will be included in the data analysis unless the participant requests to have their data withdrawn.

At a minimum, collect the following information when a subject discontinues:

The reason the subject discontinued if possible

The date of the last dose of study medications from the trial

The date of the last assessment and/or contact

(Serious) Adverse Events

Final Assessments: Every effort should be made to ensure that all procedures and evaluations scheduled for the final study visit are performed.

8.8 Concomitant Care

There are no restrictions on concomitant care for enrolled participants. Treatment with any or all of the following therapies *is not* of itself reason to withhold nebulised heparin: deep vein thrombosis prophylaxis with unfractionated heparin or LMWH; 'full' therapeutic dose unfractionated heparin or LMWH; non-heparin anticoagulants; anti-thrombotic medications; protamine; prone positioning; and inhaled nitric oxide.

8.9 Definition of End of Trial

The end of the trial is 60 days after the date and time of the last patient being enrolled in the study. This will allow for the full data set for that patient to be collected.

9. Investigational Medicinal Product

9.1 IMP Description

Heparin sodium 5,000 I.U./ml (25,000 I.U. in 5 ml), manufactured by Wockhardt UK Ltd., Ash Road North, Wrexham, LL13 9UF, United Kingdom. 5ml will be administered via the Aerogen solo nebuliser every 6 hours from enrolment to day 10. The investigator will ensure that heparin is prescribed in this manner on the electronic ICU drug prescription. It will be administered as prescribed by intensive care nursing staff in the manner described in section 8.7 of this document.

9.2 IMP Supply, Labelling, Storage, and Handling

IMP for this study will be taken from hospital standard stock. As an open label study, study drug labelling will not be required. The ICU drug prescription record will contain a record of all administered drug. The IMP does not require any special storage conditions – it can be stored at room temperature. All prescription medications are stored in a locked room in the intensive care unit. The IMP used in the trial is licenced in Ireland for their own specific uses but not for this indication proposed in this study thus it is an Investigational Medicinal Product under Irish and EU legislation. The supplies of these drugs used in the trial will come from commercial stock supplied to the hospitals in Ireland.

The study drug used for this study is not licenced for use to treat patients with COVID-19 and there is no evidence that this drug will work for this indication.

IMP labels will comply with regulation 43 of SI 190 labelling and shall be "such as to ensure protection of the subject and traceability, to enable identification of the product and trial, and to facilitate proper use of the investigational medicinal product".

While the IMP labelling does not comply with the labelling requirements of Annex 13, we will utilize the following key hospital procedures to control and document the storage, prescription, dispensing and administration of the IMPs. These will include:

- Drug will be prescribed by a physician and noted on the hospital system
- Email noting drug randomisation will be filed in the patients notes
- Drug usage instructions noted
- Double check of drugs as per normal hospital practice
- IMP delivered by health care professionals in a health care setting only (no trial medication doses to be taken at home)
- Ward staff will be trained on IMP preparation by PI, as necessary, using trial specific instructions.
- Clearly recorded in patient medical notes that person is in the trial and what trial medication(s) patient is taking

The nebulised heparin will be prescribed on the patient's medication administration record by a treating physician. The prescription will specify the drug (heparin sodium), the dose (25,000 Units in 5 mL), the frequency (6-hourly), the route (nebulised inhalation), the duration (10 days) and the indication (while receiving invasive ventilation in intensive care). DO NOT GIVE IV or SC.

9.3 IMP Accountability

The IMP will be supplied to site as per the process detailed above. The investigator will use a standard prescription form and the investigator/research delegate will collect the medication from its designated storage space. The drug will be dispensed by authorised personnel according to local and study procedures. A dispensing log will be kept for each participant to document all drug dispensed to the participant.

Assignment of Interventions

10. Allocation

10.1 Sequence Generation

Allocation will be carried out after consent is confirmed, via a central, secure web randomisation process hosted by the CRO. Allocation will be in a one to one ratio, with variable block size randomisation.

10.2 Allocation Concealment

Blocks of variable size and a random seed will be used to ensure allocation concealment cannot be violated by deciphering the sequence near the end of each block. To further protect from deciphering, block size will not be revealed to site Investigators. Site level randomisation will be used. At randomisation each participant is assigned to nebulised heparin or standard care. There is a one to one allocation ratio.

10.3 Implementation

Research nurses and investigators at each site will work with clinicians to identify potential candidates for enrolment. A log will be maintained of patients who met the inclusion criteria but were not enrolled, with the reason for exclusion recorded on the log.

10.4 Procedures for Handling Incorrectly Enrolled or Randomised Participants

Where a participant does not meet all the eligibility criteria but is randomised in error, or incorrectly started on treatment, the Investigator should inform the Sponsor/sponsor representative immediately.

DATA COLLECTION MANAGEMENT AND ANALYSIS

11. Data Collection Methods

Data will be collected by trained staff at each site under the supervision of the principal investigator using a case report form and data dictionary developed by the management committee. This information will be re-identifiable by the participating site to facilitate audits against source documents.

Baseline Data

Baseline data will be gathered from health records at the treating institution. It may also be necessary in a small number of cases to obtain information from other healthcare providers.

Daily data for days 0 to 10 while in intensive care

Data are gathered from health records at the treating institution for each day that the patient is in ICU up to and including day 10. Day 28 and day 60 data are gathered from health records at the treating institution and, where necessary, other healthcare providers.

Adverse event or adverse reaction data are gathered from health records at the recruiting institution and, where necessary, other healthcare providers and the patient or proxy.

12. Data Management

All electronic information held will be kept on password protected systems/computers accessible only to authorised study personnel and complaint with ICH GCP.

All study material, including case report forms and the study database, will be stored for a minimum period of 15 years after the conclusion of the study or as per national/European legislation.

12.1 Source Data

Source documents are the documents or media where the patients data are first recorded, and from which the data participants' entered into the electronic Case Report Form (eCRF) data is obtained. These include, but are not limited to, data gathered from health records at the treating institution, medical records from other institutions where applicable.

Sources of data will be identified and pre-specified on a study-specific source data agreement relevant to the study site. The source data agreement will be verified by the Principal Investigator with a copy of the agreement to be filed within the Site File.

The site investigator(s) will facilitate the permit of study-related monitoring, audit, research ethics committee approval review and regulatory inspection by the relevant national authority/international, providing direct access to source documents where necessary.

Data collected will be pseudo-anonymised; on all study-specific documents other than the signed consent, the subject will be referred to by the study subject identification number/code.

Subject participation and subject progress should also be recorded in the subject medical records to ensure relevant healthcare providers have knowledge of the subject's participation in the study.

Data from medical records will be entered by authorised and delegated site personnel into the case report form. The data will be verifiable against original records and source notes by the Monitor during monitoring visits. Data reported on the CRF that are derived from source documents must be consistent with the source documents or the discrepancies must be explained.

CRF completion instructions will be provided and any questions about recording specific information on the CRF should be directed to the investigator. Each participating centre will maintain a subject identification log of enrolled patients that includes patient identifiers. Patient identifiers are not transferred to the study coordinating centre.

12.2 Data Quality Assurance

The study will be conducted in accordance with the current approved protocol, ICH GCP, relevant regulations and standard operating procedures. All effort shall be taken to ensure that data collected is accurate, complete and reliable. The site must facilitate all required monitoring and respond to queries in a timely manner. All data will be reviewed for completeness and logical consistency by the Data Management team. Data queries will be generated via the electronic data capture system to correct or clarify data or request missing information. The designated site staff will be required to respond to these queries in accordance with data entry and data query timelines for the study.

The investigator will be responsible for the review and sign off of data entered and corrected for their site.

12.3Clinical Trial Documentation

Study documents will be stored within the Site File according to Institutional standard operating procedures and will adhere to the requirements of section 8 of ICH GCP

12.4 Access to Data

Only authorised study staff will be permitted access to the password protected study database where study specific data will be stored. Paper documents will be stored in a locked site with restricted access. On all trial-specific documents, other than the signed consent form and the enrolment log, the participant will be referred to by the unique participant code, not by name. Patient Identifiable data will not leave the site, as per the Data Protection Act.

Access will be granted to authorised representatives from the sponsor and the regulatory authorities to permit trial-related monitoring, audits and reports.

12.5 Data Sharing

Data sharing agreements will be drawn up between participating centres and the CRO who will administer the eCRF. Participant consent will include a provision for sharing of anonymised data for the purposes of meta-analysis. Data sharing will adhere to GDPR legislation.

12.6 Data Recording and Record Keeping

Study data will be recorded on an electronic Case Report Form (eCRF). The eCRF will be designed and administered by the contract research organisation.

13. Statistical Methods

13.1 Description of Statistical Methods

A broad description of the statistical methods is outlined below. A complete statistical analysis plan (SAP) will be finalised prior to study enrolment, at the latest before any substantial information in the trial has accumulated.

13.2 Analysis Sets

13.2.1 Intention-to-Treat Analysis Set

The intention-to-treat analysis set, also termed full analysis set in the International Conference on Harmonization (ICH) E9 guideline, will include all randomised participants.

13.2.2 Per protocol and safety analysis set

The per protocol analysis set will include all randomised patients who received at least one dose of the study drug, or who had data collection completed at least one time point in the standard care group.

13.3 Demographic and baseline disease characteristics

Demographic and baseline characteristics of the study population will be summarized using appropriate graphical and numerical summaries for each treatment group and by site.

13.4 Effectiveness Analysis

Linear and non-linear mixed effects models, using appropriate link functions depending on type of outcome variable, will be used to compare the treatment effect, while adjusting for patient characteristics (e.g. P/F ratio, SOFA Score, BMI, sex and age) as appropriate for all primary and secondary outcomes. A Cox Proportional Hazards model will be used to model time to event responses. Primary and secondary outcome data will be suitable for meta-analysis to further assess clinically relevant outcomes.

Interval estimates (95% confidence intervals) for the treatment effect will be reported accordingly.

13.4.1 Primary Effectiveness Outcomes

The co-primary efficacy outcomes are:

1 A between group difference in d-dimer levels over time.

2 A between group difference in the occurrence of serious adverse events as listed in the safety analysis

13.4.2 Secondary outcomes

Secondary efficacy outcomes include:

Analysis of between group differences in:

1. Oxygenation index (OI)
    a. OI will be recorded every 6 hours.
        i. (OI= (FiO_2 x M_{PAW}) / PaO_2 where OI = Oxygenation index, FiO_2 = Fraction of inspired oxygen (percent), M_{PAW} = Mean airway pressure and PaO_2 = Partial pressure of oxygen in arterial blood.
2. Pulmonary compliance
    a. Static compliance as measured by the ICU ventilator / recorded on the electronic ICU record
3. Inflammatory and coagulation markers
    a. IL-1β, IL-6, IL-8, IL-10 and soluble TNF receptor 1 (sTNFR1), C-reactive protein, procalcitonin, Ferritin, Fibrinogen; lactate dehydrogenase will be assessed
    b. AUC on days 1, 3, 5 and 10 as part of an exploratory outcome to assess for any potential markers of COVID 19 ARDS
    c. Ratio of IL-1β/IL-10 and IL-6/IL-10 will also be assessed
4. Time to separation from invasive ventilation to day 28, where non-survivors to day 28 are treated as though not separated from invasive ventilation.

a. ('Invasive ventilation' means any positive pressure ventilatory support via an endotracheal or tracheostomy tube. If a patient achieves separation from invasive ventilation more than once, it is the final separation that is used to calculate the outcome.)
b. In this study, 'day 0' describes the period from randomisation to midnight on the day of enrolment, 'day 1' the first calendar day after the day of enrolment, 'day 2' the second calendar day after the day of enrolment, and so forth.

5. Number treated with neuromuscular blockers instituted after enrolment to day 10
   a. Any administration of neuromuscular blocking drugs will be recorded
6. Number treated with prone positioning instituted after enrolment to day 10
7. Number treated with extra-corporeal membrane oxygenation instituted after enrolment to day 10
8. Number tracheotomised to day 28
9. Time to separation from invasive ventilation to day 28, among survivors
10. Time to separation from the ICU to day 28, where non-survivors to day 28 are treated as though not separated from invasive care
11. Time to separation from the ICU to day 28, among survivors
    a. During the pandemic critically ill inpatients might be cared for outside of the walls of the usual physical environment of ICU. For this reason, 'ICU' is defined as an area designated for inpatient care of the critically ill where therapies including invasive mechanical ventilation can be provided.
12. Survival to day 28; Survival to day 60; and Survival to hospital discharge, censored at day 60
13. Number residing at home or in a community setting at day 60
14. Number residing at home or in a community setting at day 60, among survivors

13.4.3 Safety outcomes

1 Air quality samples from an infection control perspective
2 Number of patients transfused red blood cells (packed red cells and whole blood) to day 10
3 Volume of red blood cells (packed red cells and whole blood) transfused to day 10
4 Number who record major bleeding
   a. 'Major bleeding' is defined as: bleeding that results in death and/or; bleeding that is symptomatic and occurs in a critical area or organ (intra-cranial, intra-spinal, intra-ocular, retroperitoneal, intra-articular, or intramuscular with compartment syndrome) and /or; bleeding that results in a fall in haemoglobin of 20g/L or more, or results in transfusion of two or more units of whole blood or red cells.
5 Number who record heparin-induced thrombocytopenia (HIT)
   i. 'HIT' is an unexplained fall in platelet count and a positive heparin antibody test.
6 Number who record other adverse events and reactions

13.5 The level of statistical significance

The level of statistical significance will be set at α=0.05 for the primary outcome of change in d-dimer. i.e. a p-value <0.05 with 95% CI's not containing zero will be considered statistically significant.

13.6 Procedure for accounting for missing, unused and spurious data

An analysis of all missing data will be carried out to identify the likely missing data mechanism (e.g. missing completely at random, missing at random, and missing not at random) and to investigate reasons for missingness as a useful outcome for the corresponding subsequent definitive trial.

13.7 Sample Size

This is a proof of principle / feasibility study aiming to evaluate the effect of nebulised unfractionated heparin on procoagulant markers related to acute respiratory distress syndrome in patients invasively ventilated for covid 19 lung disease. This has not been studied previously, and as such, data does not exist to power the study accurately to assess clinical outcomes. We have chosen to base our power analysis on some of our own in house data for d-dimer levels in covid patients who required ICU care (12 patients) and those who didn't (26 patients).

Based on a calculation that nebulised heparin may reduce the d-dimer levels in ICU patients (mean = 944.8ng/ml [SD = 485.3]) to those experienced in ward patients (mean = 436.5ng/ml[SD = 604.0]), with an alpha level of 0.05 and a power of 90% to detect a type II error 19 patients per group would be required. Increasing the number by 1 per group to allow for potential dropout gives a sample size of 20 patients per arm of the study, with a total of 40 patients to be enrolled. The safety profile of administering nebulised heparin to invasively ventilated patients with COVID 19 will be a co-primary outcome, but power analysis will not be done for this outcome.

13.8 Interim Analyses

There is no plan to carry out an interim analysis as the primary outcome is not a clinical outcome, and the numbers recruited are small.

13.9 Criteria for Ending the Trial

If external data from other studies or regulators becomes available showing unexpected harms associated with the drug or the method of administration, following discussion with the NREC / sponsor, a decision may be made to stop the trial.

13.10 Criteria for the Termination of the Trial

Early termination may occur at the discretion of the Investigators for any reason that is believed may present a safety risk.

Investigator: If the investigator terminates or suspends a trial without prior agreement of the Sponsor, the investigator should inform the institution where required, by the applicable regulatory requirements and the investigator/institution should promptly inform the sponsor and the REC, and should provide the sponsor and the REC a detailed written explanation for the termination or suspension.

Sponsor: If the Sponsor terminates or suspends a trial, the Sponsor should promptly inform the investigator. The investigator should then promptly inform the REC and HPRA and provide a detailed written explanation for the termination or suspension.

NREC: If the responsible Research Ethics Committee terminates or suspends its approval/favourable opinion of a trial, the investigator should inform the Institution where required, by the applicable regulatory requirements, and the investigator/institution should promptly notify the sponsor and provide the sponsor with a detailed written explanation for the termination or suspension.

13.11 Inclusion in Analysis

All participants who are enrolled will be included in the analysis. Any participants who withdraw and wish to have their data withdrawn will not be included. Analysis will be carried out using the intention to treat principle. A separate per-protocol analysis will be carried out including all patients who received any amount of the study drug, or had data collection for at least part of one day after randomisation in the standard care arm.

13.12 Procedures for Reporting any Deviation(s) from the Original Statistical Plan

Any deviation(s) from the original statistical plan will be described and justified in the final report.

14. Safety Reporting

The following adverse events and reactions must be reported:

- Major bleeding which is bleeding that results in death and/or; bleeding that is symptomatic and occurs in a critical area or organ (intra-cranial, intra-spinal, intra-ocular, retroperitoneal, intra-articular, or intramuscular with compartment syndrome) and/or; bleeding that results in a fall in haemoglobin of 20g/L or more, or bleeding that results in transfusion of two or more units of whole blood or red cells

- Pulmonary bleeding, which is frank bleeding in the lungs, trachea or bronchi with repeated haemoptysis or requiring repeated suctioning and associated with acute deterioration in respiratory status

- HIT, which is an unexplained fall in platelet count and a positive heparin antibody test

- Adverse events and reactions that, in the site principal investigator's judgement, are not part of the expected clinical course and could be related (at least possibly) to the study and are medically significant or had serious sequelae.

Blood transfusion is common in the study population, is recorded daily on the case report form up to day 10 and should not be reported as an adverse event unless the reason for transfusion is major bleeding or pulmonary bleeding.

Simple bloodstaining of the respiratory secretions is common in the study population and should not be reported as an adverse event.

Changes in APTT are common in the study population and should not be reported as an adverse event. The highest daily APTT is recorded on the case report form up to day 10.

Taking account of the pharmacodynamic profile of nebulised heparin and allowing a margin of safety, investigators should be alert to possible adverse events or reactions during the period from enrolment until 96 hours after the last dose of nebulised heparin.

14.1 Definitions

Adverse Event (AE)	Any untoward medical occurrence in a participant to whom a medicinal product has been administered, including occurrences which are not necessarily caused by or related to that product.
Adverse Reaction (AR)	An untoward and unintended response in a participant to an investigational medicinal product which is related to any dose administered to that participant.

The phrase "response to an investigational medicinal product" means that a causal relationship between a trial medication and an AE is at least a reasonable possibility, i.e. the relationship cannot be ruled out.

All cases judged by either the reporting medically qualified professional or the Sponsor as having a reasonable suspected causal relationship to the trial medication qualify as adverse reactions.

Serious Adverse Event (SAE)	A serious adverse event is any untoward medical occurrence that:<br>1. Results in death<br>2. Is life-threatening<br>3. Requires inpatient hospitalisation or prolongation of existing hospitalisation<br>4. Results in persistent or significant disability/incapacity<br>5. Consists of a congenital anomaly or birth defect

Other 'important medical events' may also be considered serious if they jeopardise the participant or require an intervention to prevent one of the above consequences.

NOTE: The term "life-threatening" in the definition of "serious" refers to an event in which the participant was at risk of death at the time of the event; it does not refer to an event which hypothetically might have caused death if it were more severe.

Serious Adverse Reaction (SAR)	An adverse event that is both serious and, in the opinion of the reporting Investigator, believed with reasonable probability to be due to one of the trial treatments, based on the information provided.
Suspected Unexpected Serious Adverse Reaction (SUSAR)	A serious adverse reaction, the nature and severity of which is not consistent with the information about the medicinal product in question set out:<br>In the case of this IMP which has a marketing authorisation, in the SmPC for this product

NOTE: To avoid confusion or misunderstanding of the difference between the terms "serious" and "severe", the following note of clarification is provided: "Severe" is often used to describe intensity of a specific event, which <u>may</u> be of relatively minor medical significance. "Seriousness" is the regulatory definition supplied above.

Any pregnancy occurring during the clinical trial and the outcome of the pregnancy should be recorded and followed up for congenital abnormality or birth defect, at which point it would fall within the definition of "serious".

14.2 Causality

The investigator's assessment of causality must be made for all AEs (serious and non-serious).

All adverse events judged by either the investigator or the sponsor as having a reasonable suspected causal relationship to an investigational medicinal product qualify as adverse reactions to the study drug.

The causality assessment given by the investigator should not be downgraded by the sponsor.

The investigator must make an assessment of whether the AE/SAE is likely to be related to treatment according to the following definitions:

Unrelated	Where an event is not considered to be related to the study medication.
Possibly Related	Although a relationship to the study medication cannot be completely ruled out, the nature of the event, the underlying disease, concomitant medication or temporal relationship make other explanations possible.
Probably Related	The temporal relationship and absence of a more likely explanation suggest the event could be related to the study medication.

All AEs/SAEs judged as having a reasonable suspected causal relationship (e.g. possibly, probably) to the study medication will be considered as adverse reactions (ARs) or serious adverse reactions (SARs). All AEs/SAEs judged as being related (e.g. possibly, probably) to an interaction between the study medication and another medication will also be considered to be ARs/SAR.

Alternative causes such as natural history of the underlying disease, concomitant therapy, other risk factors and the temporal relationship of the event to the treatment should be considered.

14.3 Assessment of Severity

The investigator will make an assessment of severity for each AE/SAE and record this on the CRF according to one of the following categories:

Grade 1	Mild; asymptomatic or mild symptoms; clinical or diagnostic observations only; intervention not indicated.
Grade 2	Moderate; minimal, local or non-invasive intervention indicated; limiting age appropriate instrumental ADL*.
Grade 3	Severe or medically significant but not immediately life-threatening; hospitalization or prolongation of hospitalization indicated; disabling; limiting self-care ADL**.
Grade 4	Life-threatening consequences; urgent intervention indicated.
Grade 5	Death related to AE.
Activities of Daily Living (ADL)	*Instrumental ADL refer to preparing meals, shopping for groceries or clothes, using the telephone, managing money, etc.

**Self care ADL refer to bathing, dressing and undressing, feeding self, using the toilet, taking medications, and not bedridden.

AEs considered related to the trial medication as judged by a medically qualified investigator or the Sponsor will be followed either until resolution, or the event is considered stable.

It will be left to the Investigator's clinical judgment to decide whether or not an AE is of sufficient severity to require the participant's removal from treatment. A participant may also voluntarily withdraw from treatment due to what he or she perceives as an intolerable AE. If either of these occurs, the participant must undergo an end of trial assessment and be given appropriate care under medical supervision until symptoms cease, or the condition becomes stable.

14.4 Assessment of expectedness

The expectedness of an adverse reaction will be determined by the sponsor according to the reference document. For the study drugs involved in this study which have a marketing authorization (commercial agent), the expectedness of an adverse event will be determined by whether or not it is listed in the summary of product characteristics (SmPC) – section 4.8 undesirable effects.

For this study, the current version of the unfractionated heparin SmPC will be used to assess the expectedness of the event in the study (*See SmPC available from HPRA website*).

14.5 Relation to Study IMP

The investigator (or designee) must determine the likelihood that the IMP caused the adverse event. The investigator must record the causal relationship in the patients' notes, as appropriate, and report such an assessment in accordance with serious adverse reporting requirements, if applicable.

An adverse event is considered associated with the use of the IMP/treatment if the attribution is definitely, probably or possibly related.

14.6 Procedures for Recording Adverse Events

Reportable adverse events and reactions will be communicated by site investigators to the chief investigator. In general, this will occur in writing within than 3 days of the site investigator becoming aware of the event. The management committee will assess all safety reports received from investigators.

All AEs occurring during the trial that are observed by the Investigator or reported by the participant, will be recorded on the CRF, whether or not attributed to trial medication.

The following information will be recorded: description, date of onset and end date, severity, assessment of relatedness to trial medication, other suspect drug or device and action taken. Follow-up information should be provided as necessary. Only non-serious Adverse Events which are considered related to the IMP will be recorded routinely on the Adverse Event CRF from the time of informed consent (or enrolment into the study) up to 96 hours after the last dose of study drug has been received. All Adverse Events meeting "serious" criteria should be recorded on the Serious Adverse Event CRF as per Section below.

14.7 Procedures for Recording and Reporting Serious Adverse Events

All SAEs must be reported on the SAE reporting form to the Sponsor within 24 hours of the Study Team becoming aware of the event.

The Sponsor will perform an initial check of the report, request any additional information, and ensure it is reviewed by the Medical Monitor on a weekly basis.

AEs meeting the definition of SAEs must be reported using the SAE Report Form located in the Investigator Site File (ISF). All AEs meeting "serious" criteria occurring in each patient should be reported from the time of informed consent (or enrolment into the study) up to 90 days after the last dose of study drug has been received. All SAE information must also be recorded on the SAE form within the CRF. Additional and further requested information (follow-up or corrections to the original case) may be captured within the SAE form and CRF. Follow-up/ new information is required within the same reporting timeline, i.e. within 24 hours of the Study Team becoming aware of the new information.

SAEs will also be reviewed by the Data Safety Monitoring Committee, in accordance with their Charter.

Sponsor Responsibilities:

The Sponsor, NUIG, will delegate appropriate adverse event reporting for the study according to the applicable regulatory guidelines.

14.8 SUSARs

NUIG will submit local SUSARs on an expedited basis to the local regulatory authority and other Competent Authorities involved, concerned Ethics Committees (EC), all participating investigators and the DSMC. NUIG sends annual Development Safety Update Reports (DSUR) to the concerned regulatory authority and Ethics Committee. Investigators will be informed of SUSARs.

For fatal and life-threatening SUSARS, this will be done no later than 7 calendar days after the Sponsor is first aware of the reaction. Any additional relevant information will be reported within 8 calendar days of the initial report. All other SUSARs will be reported within 15 calendar days.

14.9 Procedures for Documenting and Reporting Pregnancies

Females pregnant, breastfeeding or planning pregnancy at the time of recruitment will be excluded from participating in the trial.

Exposure during Pregnancy or Breastfeeding (even if not associated with an adverse event) will be reported.

14.10 Data Safety Monitoring Committee

An independent international Data Safety Monitoring Committee (DSMC) will oversee the conduct of the trial and verify the correct implementation of the clinical trial design. Just as with a traditional trial design, the role of the DSMC is to ensure the design is implemented with integrity, that the original design remains scientifically and ethically appropriate in light of the accumulating data and

any external information, and to protect research participants from avoidable risk. The DSMC for this trial will include expertise in critical care, biostatistics and clinical trials.

The responsibilities of the DSMC are to advise on all matters related to the safety of subjects enrolled in this study, integrity of trial data and conclusions and the appropriateness of continued trial conduct.

The aims of the DSMC include:

1. To identify any trends, such as increases in un/expected events, and take appropriate action
2. To seek additional advice or information from investigators where required
3. To evaluate the risk of the trial continuing and take appropriate action where necessary

The researchers will provide the DSMC with a report at interim analyses of adverse events and early results. The DSMC will also undertake a review of enrolments and withdrawals according to the DSMC Charter, to ensure adequate study safety, and minimal risk to participants. Where necessary, the DSMC will make recommendations for corrective action which may include early termination, suspension or modification of a trial, or changes in consent processes. However, the final decision will rest with the Sponsor.

QUALITY ASSURANCE PROCEDURES

15.1 Training of Study Site Personnel

The PI will ensure that appropriate training relevant to the study is given to all relevant staff, and that any new information relevant to the performance of this study is forwarded to the staff involved. The appropriate manuals and guidelines will be issued to investigators in order that they are able to perform the study as per protocol. All study site personnel will receive GCP training.

The PI will maintain a record of all individuals involved in the study and the training they have received at that site.

15.2 Monitoring and Audit

The trial will be conducted in accordance with the current approved protocol, GCP, relevant regulations and standard operating procedures.

Regular monitoring will be performed according to GCP and as defined within the study Monitoring Plan. Data will be evaluated for compliance with the protocol and accuracy in relation to source documents. The Monitor will perform SDV to verify that the clinical trial is conducted and data are generated, documented and reported in compliance with the protocol, GCP and the applicable regulatory requirements.

All data will be stored securely. Details of outcome measures and adverse events will be documented in hospital healthcare records, in individual research participant case report forms and in an encrypted electronic database.

The study investigators will ensure that all source data and healthcare records will be storage and archived in compliance with the legislation governing clinical research. The investigators and authorised designees will ensure that the confidentiality of the participants' data is preserved at all times.

This trial may be subject to internal or external auditing or inspections to ensure adherence to GCP and all required legislation and standards. Access to all trial-related documents will be given at that time.

A quality assurance audit/inspection may be conducted by the competent authority, sponsor, or an agent delegated responsibility. The investigator will facilitate this independent audit/inspection. The purpose of an audit/inspection is to confirm that the study is conducted as per protocol, GCP and applicable regulatory requirements, that the rights and well-being of the patients enrolled have been protected, and that the data relevant for the evaluation of the investigational medicinal product have been captured, processed and reported in compliance with the planned arrangements. The investigator will permit direct access to all study documents, drug accountability records, medical records and source data as required.

Data will also be monitored by the DSMC in accordance with the DSMC Charter.

16. Serious Breaches

A serious breach is defined as "A breach of GCP or the trial protocol which is likely to affect to a significant degree:

1. Participants rights
2. Participant safety or well-being
3. The integrity of research data
4. The conduct or management of the study
5. Participant's willingness to continue study Participation

In the event that a serious breach is suspected the Sponsor must be informed within 1 working day. In collaboration with the Investigator, the serious breach will be reviewed by the Sponsor and the DSMC. If deemed appropriate, the Sponsor will report the breach to the Research Ethics Committee, regulatory authority, and local governance body (as applicable) within seven calendar days.

ETHICS AND REGULATORY

17. Ethical and Regulatory Considerations

17.1 Declaration of Helsinki

This study will be conducted in accordance with the regulatory requirements, sponsor standard operating procedures (SOPs), ICH GCP and ethical principles laid out in the Declaration of Helsinki (2013) and the National Statement on Ethical Conduct in Research involving Humans.

17.2 Guidelines for Good Clinical Practice

The Investigator will ensure that this trial is conducted in accordance with relevant regulations and with Good Clinical Practice (the EU-Clinical Trial Directive (Directive 2001/20/ECEU-CDC) and with the Note for Guidance on Good Clinical Practice (CPMP/ICH/135/95).

17.3 Approvals

The study requires submission to the Health Products Regulatory Authority (HPRA) in line with the regulation of therapeutic products for clinical trials. The investigational product is a registered, approved therapeutic product but is being used off-label for the purposes of the trial.

Ethics submission will be made to the relevant Research Ethics Committee. The opinion of the Ethics Committee will be given in writing. Institutional approval must be granted at each site before any participants are recruited, as per Ethics Committee guidelines. The Ethics Committee should approve all participant facing material for the study.

The Sponsor will approve all modifications to the Protocol and/or Participant Information Leaflet and Informed Consent Form/s that are needed.

The CI or delegate will submit all substantial amendments to the original approved documents to the REC.

17.4 Reporting

The CI shall submit once a year throughout the clinical trial, or on request, a DSUR to the approving Ethics Committee and the competent authority.

An End of Trial Notification and Final Report will also be submitted in accordance with the relevant guidelines and standards.

18. Protocol Amendments

Protocol amendments will be reviewed for their impact (substantial or non-substantial) as per requirements and all substantial amendments will not be implemented prior to pre-approval by the competent authority and the EC. Protocol amendments will be updated on relevant clinical trial registries by an appropriate delegated person the sponsor or the sponsors representative.

If a protocol amendment requires a change to the Informed Consent Form or any relevant document the Sponsor or their delegate will ensure that all related documents will be updated or considered for update, and all approvals sought as required.

Protocol version changes and reasons will be listed in this document under Revision History.

19. CONSENT

The study will be conducted in accordance with the ethical principles that have their origin in the Declaration of Helsinki. Eligible patients may only be included in the trial after obtaining written informed assent/consent. Informed assent/consent must be obtained prior to conducting any trial specific procedures and the process for obtaining informed consent must be documented in the patient's medical records (source documents which will be reviewed at the time of on-site monitoring visits and at inspection/audit).

It is the responsibility of the Investigator to ensure that Subjects meet the eligibility criteria for the study and are consented into the trial prior to any study specific procedures are performed:

The conduct of a regulatory trial in the midst of pandemic presents several substantial challenges.

- Eligible subjects are by definition in isolation and any documentation that enters their room has to stay there.
- Many patients will by the time of formal diagnosis be too sick to be able to provide informed consent
- Next of kin (the legal representatives as per SI 190, for most incapacitated patients) are not in a position to travel to the hospital and are not in a position to sign a consent form.

The study participants in this trial will not have capacity to consent during the critical phase of their illness in the Intensive Care Unit as they will be in medically induced coma to facilitate mechanical ventilation and other forms of organ support. In order to comply as far as is possible with Irish and European Legislation (GDPR and HRR) in the current crisis the following modifications have been made to:

- For patients who are too ill to consent, the physician will telephone the patients legal representative (per SI 190:2004) and obtain their consent. A second person will also speak to the relative on the telephone to witness this telephone consent. The relative may be offered a copy of the blank Information leaflet/ consent form by email or post if they wish to receive this.
- Telephone consent by the relative will be recorded in the medical notes by the physician. The name of the relative who gave consent, their relationship with the participant and the date and time will be recorded in the medical notes. If the patient regains capacity they will be given the information about the trial and asked to sign the consent form before they are discharged from hospital as detailed below.
- We have obtained approval from the HR Consent Declaration Committee for this deferral of patient consent.

For patients who are conscious/ well enough to understand the participant information leaflet and sign an informed consent form, and for patients who previously had assent taken on their behalf, and are now able to provide their own informed consent:

- The doctor supplies the Patient Information Leaflet to the patient and talks to the patient about the study including answering any questions that the patient has. If the patient is willing to participate in the trial they sign and date the informed consent form. The doctor taking consent also signs and dates the informed consent form.
- The patient keeps the original signed informed consent form.
- The doctor makes a note in the patient’s medical notes to the effect that patient has agreed to take part in the WHO Solidarity Trial and has signed an informed consent form on DD-MMM- YYYY, using form A (attached)
- A picture or scan of form A will be uploaded by the physician to the WHO Solidarity database as evidence that the patient was consented. This provides evidence of consent to the study, while keeping confidentiality re the patient’s identity.
- When discharged from hospital the patient may take a picture of the original consent, or may ask to receive a copy of the blank information leaflet / consent form by email or post and destroy the paper copy, for infection control reasons.

Subjects have the right to voluntarily discontinue study treatment or withdraw from the study at any time for any reason without consequences. The investigator has the right to discontinue a subject

from study treatment or withdraw a subject from the study at any time if it is in the best interest of the subject.

Subjects must discontinue the investigational medicinal product(s) and be withdrawn from the study for any of the following reasons

- withdrawal of consent by the subject
- any medical condition that the investigator or sponsor determines may jeopardize the subject's safety if she or he continues receiving the study treatment
- pregnancy
- ineligibility (arising during the study screening)
- an adverse event which requires discontinuation of the study medication
- treatment failure and disease progression.

A number of procedures will continue to be required until the end of the study even if the treatment has been withdrawn. Patients withdrawn from the study for safety reasons after IMP administration will continue if agreeable to have long term health and ancillary studies performed. Patients that withdraw from the trial for reasons other than safety reasons before IMP administration will have no long term follow up data collected.

All subjects who discontinue should comply with the protocol specified follow-up procedures. The only exception to this requirement is when a subject withdraws consent for all study procedures.

If a subject is withdrawn before completing the study, the reason for withdrawal must be entered on the appropriate CRF page.

If a subject is withdrawn due to an adverse event, the investigator will arrange for follow-up visits until the adverse event has resolved or stabilised.

20. Participant Confidentiality and Data Protection

The research staff will ensure that participants' anonymity is maintained as per the requirements of the Data Protection Act and the Health Research Regulations.

For any pseud-anonymised data transferred outside the European Economic Area (EEA), NUIG, on behalf of Irish sites, will enter into the appropriate standard contractual clauses for European data export outside the EEA.

By signing the protocol, the investigator agrees that the sponsor, ethics committee or regulatory authorities may consult and/or copy study documents to verify information in the case record form. By signing the consent form the subject or their relative agrees to this process.

Subject confidentiality will be maintained at all times and no documents containing the subject's name or other identifying information will be collected by the sponsor on the study database. It may be necessary for the sponsor's representatives, the ethics committee and regulatory authority representatives to have direct access to the subject's medical records

20.1 Confidentiality of Study Data

By signing this protocol, the investigator affirms to the sponsor that information provided to the investigator by the sponsor will be maintained in confidence and will be divulged only as necessary to the ethics committee and institution employees directly involved in the study. Both ethics committee members and employees must also understand the confidentiality requirements for any information divulged to them. The data generated by this study will be considered confidential by the investigator, except to the extent that it is included in a publication as agreed in the publication policy of this protocol.

21. Declaration of Interests

This study has been designed in direct response to the COVID-19 disease pandemic. We report no financial or other competing interests for any of the investigators involved.

22. POST-TRIAL CARE

There will be no formal provision of the intervention beyond the trial period, as recommended by the WHO interim guidance that any specific therapy targeted to COVID-19 infection should be provided only as part of a research protocol.[18] All subsequent requirement of both COVID-19 and unrelated needs will be under the existing healthcare system in the recruiting centres.

23 Finance And Insurance

23.1 Funding and Insurance

The NATIONAL UNIVERSITY OF IRELAND, GALWAY is the SPONSOR and it will ensure that every investigator is covered by a Public Liability ('negligent harm') insurance that applies for the clinical trial. All investigators are qualified and practicing physicians and are thus insured by the clinical indemnity scheme (CIS).

All relevant insurance documentation will be obtained prior to conduct of the trial.

Funding for this study is from CURAM/SFI and Aerogen. The funders will have no input into recruitment, data collection or analysis, writing scientific papers nor the decision to publish any papers that may arise as a result of this trial.

23.2 Participant Reimbursement

Participants will not receive any reimbursement for their time and we do not expect participants to incur any costs through their involvement in the study.

24. PUBLICATION POLICY

The study site(s), represented by the chief investigator (CI), will take responsibility to report the results in a scientific peer reviewed journal, according to the International Committee of Medical Journal Editors recommendations. The Investigators listed on page one will be listed as authors, in recognition of their contribution to the design, implementation and oversight of the study.

Publication of the study outcomes will comprise publication of the study as a whole and is encouraged by the Sponsor regardless of outcome. The Sponsor retains editorial rights to protect the Sponsor's proprietary information and intellectual property.

Results of the study will be sent to participants on request (once available) and will be made available on a publicly available trial registry website, recognised by the World Health Organisation International Clinical Trials Registry Platform (WHO ICTRP) as a Primary Registry.

REFERENCES

1. Bellani G, Laffey JG, Pham T, et al. Epidemiology, Patterns of Care, and Mortality for Patients With Acute Respiratory Distress Syndrome in Intensive Care Units in 50 Countries. JAMA 2016;315:788-800.

2. Thompson BT, Chambers RC, Liu KD. Acute Respiratory Distress Syndrome. N Engl J Med 2017;377:562-72.

3. Wu C, Chen X, Cai Y, et al. Risk Factors Associated With Acute Respiratory Distress Syndrome and Death in Patients With Coronavirus Disease 2019 Pneumonia in Wuhan, China. JAMA Intern Med 2020.

4. Herridge MS, Cheung AM, Tansey CM, et al. One-year outcomes in survivors of the acute respiratory distress syndrome. N Engl J Med 2003;348:683-93.

5. Iwashyna TJ. Trajectories of recovery and dysfunction after acute illness, with implications for clinical trial design. Am J Respir Crit Care Med 2012;186:302-4.

6. Ashbaugh DG, Bigelow DB, Petty TL, Levine BE. Acute respiratory distress in adults. Lancet 1967;2:319-23.

7. Castro CY. ARDS and diffuse alveolar damage: a pathologist's perspective. Semin Thorac Cardiovasc Surg 2006;18:13-9.

8. Idell S. Coagulation, fibrinolysis, and fibrin deposition in acute lung injury. Crit Care Med 2003;31:S213-20.

9. Burns AR, Smith CW, Walker DC. Unique structural features that influence neutrophil emigration into the lung. Physiol Rev 2003;83:309-36.

10. Blaisdell FW. Pathophysiology of the respiratory distress syndrome. Arch Surg 1974;108:44-9.

11. Tomashefski JF, Jr., Davies P, Boggis C, Greene R, Zapol WM, Reid LM. The pulmonary vascular lesions of the adult respiratory distress syndrome. Am J Pathol 1983;112:112-26.

12. Dixon B. The role of microvascular thrombosis in sepsis. Anaesth Intensive Care 2004;32:619-29.

13. Greene R, Zapol WM, Snider MT, et al. Early bedside detection of pulmonary vascular occlusion during acute respiratory failure. Am Rev Respir Dis 1981;124:593-601.

14. Rubenfeld GD, Caldwell E, Peabody E, et al. Incidence and outcomes of acute lung injury. N Engl J Med 2005;353:1685-93.

15. Muller I, Klocke A, Alex M, et al. Intravascular tissue factor initiates coagulation via circulating microvesicles and platelets. Faseb J 2003.

16. Nuckton TJ, Alonso JA, Kallet RH, et al. Pulmonary dead-space fraction as a risk factor for death in the acute respiratory distress syndrome. N Engl J Med 2002;346:1281-6.

17. Cooper JR, Jr., Abrams J, Frazier OH, et al. Fatal pulmonary microthrombi during surgical therapy for end-stage heart failure: possible association with antifibrinolytic therapy. J Thorac Cardiovasc Surg 2006;131:963-8.

18. Ware LB, Matthay MA. The acute respiratory distress syndrome. N Engl J Med 2000;342:1334-49.

19. Nicholls JM, Poon LL, Lee KC, et al. Lung pathology of fatal severe acute respiratory syndrome. Lancet 2003;361:1773-8.

20. Scully RE, Galdabini JJ, McNeely BU. Case records of the Massachusetts General Hospital. Weekly clinicopathological exercises. Case 22--1977. N Engl J Med 1977;296:1279-87.

21. Gunther A, Ruppert C, Schmidt R, et al. Surfactant alteration and replacement in acute respiratory distress syndrome. Respir Res 2001;2:353-64.

22. Qin C, Zhou L, Hu Z, et al. Dysregulation of immune response in patients with COVID-19 in Wuhan, China. Clinical Infectious Diseases 2020.

23. Huang C, Wang Y, Li X, et al. Clinical features of patients infected with 2019 novel coronavirus in Wuhan, China. Lancet 2020;395:497-506.

24. Deng Y, Liu W, Liu K, et al. Clinical characteristics of fatal and recovered cases of coronavirus disease 2019 (COVID-19) in Wuhan, China: a retrospective study. Chin Med J (Engl) 2020.

25. Tang N, Bai H, Chen X, Gong J, Li D, Sun Z. Anticoagulant treatment is associated with decreased mortality in severe coronavirus disease 2019 patients with coagulopathy. J Thromb Haemost 2020.

26. Zhang H, Zhou P, Wei Y, et al. Histopathologic Changes and SARS-CoV-2 Immunostaining in the Lung of a Patient With COVID-19. Ann Intern Med 2020.

27. Tian S, Hu W, Niu L, Liu H, Xu H, Xiao SY. Pulmonary Pathology of Early-Phase 2019 Novel Coronavirus (COVID-19) Pneumonia in Two Patients With Lung Cancer. J Thorac Oncol 2020.

28. Lu R, Zhao X, Li J, et al. Genomic characterisation and epidemiology of 2019 novel coronavirus: implications for virus origins and receptor binding. Lancet 2020;395:565-74.

29. Medicine NHCSAoTC. Diagnosis and treatment Protocol for Novel Coronavirus Pneumonia. National Health Commission & State Administration of Traditional Chinese Medicine on March 3, 2020 2020.

30. Yao X. Chinese Journal of Pathology 2020;49.

31. Tang. Z, Luxi. S, F R. Comparison of clinical and pathological features between severe acte respiratory syndrome and coronavirus disease 2019. Chinese Journal Tuberculosis and Respiratory Disease 2020.

32. Tang N, Li D, Wang X, Sun Z. Abnormal coagulation parameters are associated with poor prognosis in patients with novel coronavirus pneumonia. J Thromb Haemost 2020;18:844-7.

33. Wang D, Hu B, Hu C, et al. Clinical Characteristics of 138 Hospitalized Patients With 2019 Novel Coronavirus-Infected Pneumonia in Wuhan, China. JAMA 2020.

34. Cadroy Y, Gaspin D, Dupouy D, Lormeau JC, Boneu B, Sie P. Heparin reverses the procoagulant properties of stimulated endothelial cells. Thromb Haemost 1996;75:190-5.

35. Gori AM, Pepe G, Attanasio M, et al. Tissue factor reduction and tissue factor pathway inhibitor release after heparin administration. Thromb Haemost 1999;81:589-93.

36. Pepe G, Giusti B, Attanasio M, et al. Tissue factor and plasminogen activator inhibitor type 2 expression in human stimulated monocytes is inhibited by heparin. Semin Thromb Hemost 1997;23:135-41.

37. Huber K, Resch I, Rosc D, Probst P, Kaindl F, Binder BR. Heparin induced increase of t-PA antigen plasma levels in patients with unstable angina: no evidence for clinical benefit of heparinization during the initial phase of treatment. Thromb Res 1989;55:779-84.

38. Tangphao O, Chalon S, Moreno HJ, Jr., Abiose AK, Blaschke TF, Hoffman BB. Heparin-induced vasodilation in human hand veins. Clin Pharmacol Ther 1999;66:232-8.

39. Ahmed T, Garrigo J, Danta I. Preventing bronchoconstriction in exercise-induced asthma with inhaled heparin. N Engl J Med 1993;329:90-5.

40. Koenig A, Norgard-Sumnicht K, Linhardt R, Varki A. Differential interactions of heparin and heparan sulfate glycosaminoglycans with the selectins. Implications for the use of unfractionated and low molecular weight heparins as therapeutic agents. J Clin Invest 1998;101:877-89.

41. Thomas R, Brooks T. Common oligosaccharide moieties inhibit the adherence of typical and atypical respiratory pathogens. J Med Microbiol 2004;53:833-40.

42. Dixon B, Schultz MJ, Hofstra JJ, Campbell DJ, Santamaria JD. Nebulized heparin reduces levels of pulmonary coagulation activation in acute lung injury. Crit Care 2010;14:445.

43. Dixon B, Campbell DJ, Santamaria JD. Elevated pulmonary dead space and coagulation abnormalities suggest lung microvascular thrombosis in patients undergoing cardiac surgery. Intensive Care Medicine 2008;34:1216-23.

44. Dixon B, Schultz MJ, Smith R, Fink JB, Santamaria JD, Campbell DJ. Nebulized heparin is associated with fewer days of mechanical ventilation in critically ill patients: a randomized controlled trial. Crit care 2010;14:R180.

45. Dixon B, Opeskin K, Stamaratis G, et al. Pre-operative heparin reduces pulmonary microvascular fibrin deposition following cardiac surgery. Thromb Res 2011;127:e27-30.

46. Dixon B, Smith R, Santamaria JD, et al. A trial of nebulised heparin to limit lung injury following cardiac surgery. Anaesth Intensive Care 2016;44:28-33.

47. Dixon B, Smith R. Nebulised Heparin for Lung Injury - Clinical Protocol V1: St.Vincent's Hospital Melbourne Australia; 2011.

48. Lin L, Lu L, Cao W, Li T. Hypothesis for potential pathogenesis of SARS-CoV-2 infection-a review of immune changes in patients with viral pneumonia. Emerg Microbes Infect 2020;9:727-32.

49. Rostand KS, Esko JD. Microbial adherence to and invasion through proteoglycans. Infect Immun 1997;65:1-8.

50. Carr J. The anti-inflammatory action of heparin: heparin as an antagonist to histamine, bradykinin and prostaglandin E1. Thromb Res 1979;16:507-16.

51. Porzionato A, Macchi V, Parenti A, De Caro R. The distribution of mast cells in the human area postrema. J Anat 2004;204:141-7.

52. Valent P, Baghestanian M, Bankl HC, et al. New aspects in thrombosis research: possible role of mast cells as profibrinolytic and antithrombotic cells. Thromb Haemost 2002;87:786-90.

53. Nader ND, Knight PR, Bobela I, Davidson BA, Johnson KJ, Morin F. High-dose nitric oxide inhalation increases lung injury after gastric aspiration. Anesthesiology 1999;91:741-9.

54. Idanpaan-Heikkila I, Simon PM, Zopf D, et al. Oligosaccharides interfere with the establishment and progression of experimental pneumococcal pneumonia. J Infect Dis 1997;176:704-12.

55. Bryan R, Feldman M, Jawetz SC, et al. The effects of aerosolized dextran in a mouse model of Pseudomonas aeruginosa pulmonary infection. J Infect Dis 1999;179:1449-58.

56. Liang OD, Ascencio F, Fransson LA, Wadstrom T. Binding of heparan sulfate to Staphylococcus aureus. Infect Immun 1992;60:899-906.

57. Tsang KW, Shum DK, Chan S, et al. Pseudomonas aeruginosa adherence to human basement membrane collagen in vitro. Eur Respir J 2003;21:932-8.

58. Martinez I, Melero JA. Binding of human respiratory syncytial virus to cells: implication of sulfated cell surface proteoglycans. J Gen Virol 2000;81:2715-22.

59. Hosoya M, Balzarini J, Shigeta S, De Clercq E. Differential inhibitory effects of sulfated polysaccharides and polymers on the replication of various myxoviruses and retroviruses, depending on the composition of the target amino acid sequences of the viral envelope glycoproteins. Antimicrob Agents Chemother 1991;35:2515-20.

60. Pierce CM, Wade A, Mok Q. Heparin-bonded central venous lines reduce thrombotic and infective complications in critically ill children. Intensive Care Med 2000;26:967-72.

61. Mycroft-West C, Su D, Elli S, et al. The 2019 coronavirus (SARS-CoV-2) surface protein (Spike) S1 Receptor Binding Domain undergoes conformational change upon heparin binding. bioRxiv 2020.

62. Lang J, Yang N, Deng J, et al. Inhibition of SARS pseudovirus cell entry by lactoferrin binding to heparan sulfate proteoglycans. PLoS One 2011;6:e23710.

63. Vicenzi E, Canducci F, Pinna D, et al. Coronaviridae and SARS-associated coronavirus strain HSR1. Emerg Infect Dis 2004;10:413-8.

64. de Haan CA, Li Z, te Lintelo E, Bosch BJ, Haijema BJ, Rottier PJ. Murine coronavirus with an extended host range uses heparan sulfate as an entry receptor. J Virol 2005;79:14451-6.

65. Madu IG, Chu VC, Lee H, Regan AD, Bauman BE, Whittaker GR. Heparan sulfate is a selective attachment factor for the avian coronavirus infectious bronchitis virus Beaudette. Avian Dis 2007;51:45-51.

66. Cagno V, Tseligka ED, Jones ST, Tapparel C. Heparan Sulfate Proteoglycans and Viral Attachment: True Receptors or Adaptation Bias? Viruses 2019;11.

67. Milewska A, Zarebski M, Nowak P, Stozek K, Potempa J, Pyrc K. Human coronavirus NL63 utilizes heparan sulfate proteoglycans for attachment to target cells. J Virol 2014;88:13221-30.

68. Dixon B, Smith R, Santamaria J, Moran J. Nebulized Heparin for Patients With or at Risk of the Acute Respiratory Distress Syndrome. 2020 (Unpublished).

69. Dixon B, Santamaria JD, Campbell DJ. A phase 1 trial of nebulised heparin in acute lung injury. Crit Care 2008;12:R64.

70. Lowering risk of COVID-19 transmission for health care professionals. 2020. https://www.aerogen.com/COVID-19/

71. Servo Duo Filter. https://www.puls-norge.no/media/300508/servoduoguard_data-sheet.pdf

FREDERICK HEALTH HOSPITAL
APPLICATION FOR APPROVAL OF PHARMACY RESEARCH PROTOCOL

Send completed application packet to the Nancy Trimble (ntrimble@fmh.org), Frederick Memorial Hospital, 400 West 7th Street, Frederick, Maryland, 21701; 240-566-3513.

A complete application packet includes:

1. Pharmacy Research Protocol
2. Informed Consent Form (if needed)
3. Curriculum Vitae
4. Financial Disclosure Form
5. Principal Investigator Signature Page

PROJECT TITLE:_INHALEd unfractionated HEParin for the treatment of hospitalized patients with COVID-19 (INHALE-HEP)

INVESTIGATOR TO CONTACT: _Thomas Smoot________________________________
Institution or Agency: ____Frederick Health Hospital____________________

Address: _400 West 7th Street Frederick, MD 21701_____________________________

Phone: _240-566-3300 Ext. 3250__________ Email: TLSmoot@frederick.health

____
(Investigator Signature) (Date)

____
(Signature of Advisor, if student) (Date)

Frederick Health Hospital
Pharmacy Research Protocol

**Investigator(s) Information**	
1. Protocol Title:	INHALEd unfractionated HEParin for the treatment of hospitalized patients with COVID-19 (INHALE-HEP)
2. Principal Investigator Name & Contact Information:	Thomas Smoot PharmD, BCPS<br>tlsmoot@frederick.health<br>240-566-3250
3. Co-investigator Name & Contact Information:	Khine Sann (Ksann@frederick.health)<br>Caitlin Sas (Csas@frederick.health)<br>Sabrina Staas (Rstaas@frederick.health)<br>Anusha Belani (Abelani@frederick.health)<br>Chris Hillman (Chillman@frederick.health)<br>Sue Archer (SArcher@frederick.health)<br>Nicholas Biggs (NBiggs@frederick.health)
**Study Overview**	
4. Scientific/scholarly rationale for the study (summary of literature review)	In December 2019, a novel coronavirus (severe acute respiratory syndrome coronavirus 2, SARS-CoV-2) emerged in China and has since spread globally. Nearly 20% of patients with coronavirus disease 2019 (COVID-19) experience hypoxemia, which is the primary reason for hospitalization. (1) A significant proportion of patients admitted to hospital for COVID-19 develop acute hypoxemic respiratory failure, requiring invasive mechanical ventilation. (2, 3)<br>The pathophysiology of COVID-19 associated lung injury is summarized in Figure 1A and is characterized by diffuse alveolar damage, hyperinflammation, coagulopathy, DNA neutrophil extracellular traps (NETS), hyaline membranes and microvascular thrombosis.<br>The scientific rationale and current pre-clinical and clinical evidence for the use of nebulized unfractionated heparin (UFH) as a treatment for COVID-19 has been outlined in a comprehensive review article. (4) Nebulized UFH has anti-viral, anti-inflammatory, anticoagulant, and mucolytic effects. The SARS-CoV-2 Spike S1 protein receptor binding domain attaches to UFH and undergoes conformational change that prevents it from binding ACE-2 and as a result the virus is inactivated.(5, 6) This antiviral effect of heparin has been confirmed in studies performed by Public Health England where an UFH preparation produced a concentration dependent inhibition of SARS-CoV-2 infection of Vero E6 cells, further suggesting that UFH may prevent invasion of pulmonary epithelium and vascular endothelium.(7) Administration of nebulized heparin may therefore also provide some protection for hospital staff through inactivation of the virus in the patient's respiratory tree thus reducing the expired active viral load. The anti-inflammatory effects of inhaled UFH are thought to reduce pulmonary hyperinflammation and the generation of DNA NETs, both of which contribute to COVID-19 lung injury. The anticoagulant actions of

	nebulized UFH limit fibrin deposition, hyaline membrane formation and microvascular thrombosis, which are also important features of COVID-19. The proposed effects of nebulized UFH in COVID-19 are summarized in Figure 1B. Animal studies of nebulized UFH in different acute lung injury models have consistently shown a positive effect on pulmonary coagulation, inflammation and oxygenation.(4) Small human studies indicate that nebulized UFH limits pulmonary fibrin deposition, attenuates progression of acute lung injury and hastens recovery.(4) Early-phase trials in patients with acute lung injury and related conditions found that nebulized UFH reduced pulmonary dead space, coagulation activation, microvascular thrombosis and deterioration in the Murray Lung Injury Score and increased time free of ventilator support.(8-12) A multi-center randomized double-blind placebo-controlled trial of nebulized heparin in 256 patients with or at risk of developing ARDS, investigated whether nebulized UFH accelerated recovery. This study demonstrated reduced progression of lung injury, fewer cases of ARDS and accelerated recovery with more survivors at home by day 60. [REF Dixon et al Lancet]<br><br>1. Wu Z, McGoogan JM. Characteristics of and Important Lessons From the Coronavirus Disease 2019 (COVID-19) Outbreak in China: Summary of a Report of 72314 Cases From the Chinese Center for Disease Control and Prevention. JAMA. 2020.<br>2. Grasselli G, Zangrillo A, Zanella A, Antonelli M, Cabrini L, Castelli A, et al. Baseline Characteristics and Outcomes of 1591 Patients Infected With SARS-CoV-2 Admitted to ICUs of the Lombardy Region, Italy. JAMA. 2020.<br>3. Wu C, Chen X, Cai Y, Xia J, Zhou X, Xu S, et al. Risk Factors Associated With Acute Respiratory Distress Syndrome and Death in Patients With Coronavirus Disease 2019 Pneumonia in Wuhan, China. JAMA Intern Med. 2020.<br>4. van Haren FMP, Page C, Laffey JG, Artigas A, Camprubi-Rimblas M, Nunes Q, et al. Nebulised heparin as a treatment for COVID-19: scientific rationale and a call for randomised evidence. Crit Care. 2020;24(1):454.<br>5. Mycroft-West C, Su D, Elli S, Guimond S, Miller G, Turnbull J, et al. The 2019 coronavirus (SARS-CoV-2) surface protein (Spike) S1 Receptor Binding Domain undergoes conformational change upon heparin binding. bioRxiv. 2020.<br>6. Kwon PS, Oh H, Kwon SJ, Jin W, Zhang F, Fraser K, et al. Sulfated polysaccharides effectively inhibit SARS-CoV-2 in vitro. Cell Discov. 2020;6:50.<br>7. Tree JA, Turnbull JE, Buttigieg KR, Elmore MJ, Coombes N, Hogwood J, et al. Unfractionated heparin inhibits live wild-type SARS-CoV-2 cell infectivity at therapeutically relevant concentrations. Br J Pharmacol.

	2020.<br>8. Dixon B, Schultz MJ, Hofstra JJ, Campbell DJ, Santamaria JD. Nebulized heparin reduces levels of pulmonary coagulation activation in acute lung injury. Crit Care. 2010;14(5):445.<br>9. Dixon B, Campbell DJ, Santamaria JD. Elevated pulmonary dead space and coagulation abnormalities suggest lung microvascular thrombosis in patients undergoing cardiac surgery. Intensive Care Medicine. 2008;34(7):1216-23.<br>10. Dixon B, Schultz MJ, Smith R, Fink JB, Santamaria JD, Campbell DJ. Nebulized heparin is associated with fewer days of mechanical ventilation in critically ill patients: a randomized controlled trial. Crit care. 2010;14(5):R180.<br>11. Dixon B, Smith R, Santamaria JD, Orford NR, Wakefield BJ, Ives K, et al. A trial of nebulised heparin to limit lung injury following cardiac surgery. Anaesth Intensive Care. 2016;44(1):28-33.<br>12. Dixon B, Smith R. Nebulised Heparin for Lung Injury - Clinical Protocol V1. St.Vincent's Hospital Melbourne Australia; 2011.
5. Purpose/Objective of this study:	The primary objective is to demonstrate that inhaled nebulized UFH in hospitalized patients with COVID-19 who do not require immediate invasive mechanical ventilation, significantly reduces the proportion of patients who receive invasive mechanical ventilation at day 28, compared to placebo.
6. Research design:	Double Blind, Randomized, Placebo Controlled Trial
7. Research Hypothesis and/or questions:	Nebulized UFH will reduce the number of patients that need invasive mechanical ventilation by day 28.<br><br>Nebulized heparin will reduce the mean FiO2 requirements of patients hospitalized with COVID-19.
**Methods/Procedures:**	
8. Describe all research procedures to include what will be done, how it will be done, where it will be done, what data will be collected, and who will be involved.	The term "meta-trial" has been previously referenced by Li, Pavlov et al[1] referring to a prospective meta-analysis planned to streamline data collection from multiple individual trials, allowing for faster accumulation of data for major clinical endpoints during the pandemic. This meta-trial is designed as a collaborative individual participant prospective data meta-analysis of investigator-initiated, multi-center, randomized, studies either as blinded placebo-controlled trials or as open label and early phase studies of inhaled nebulized UFH in addition to standard care compared to standard care alone in hospitalized patients with confirmed COVID-19.<br><br>The design of the research at Frederick Health Hospital (FHH) will be a randomized double-blind, placebo-controlled trial. The procedures for screening, randomization and therapy are described below.

Screening

Investigators will evaluate patients admitted to FHH with a positive COVID-19 PCR on a daily basis to determine eligibility for enrollment. The investigation team will contact the attending physician of record to obtain permission to enroll the patient. They will be provided with education, study materials and contact number for coordinating patient care. Additionally, education will be provided to all providers to ensure they are aware of the study and its potential implications on care. If eligible, the investigators will attempt to obtain informed consent from the patient or if the patient is unable to provide consent, their authorized healthcare agent will be contacted. Once consent has been obtained, the patient will undergo randomization.

Randomization

A blocked randomization sequence will be generated by a pharmacist that is not involved with the study. The randomization sequence will consist block sizes of 2 and 4. This sequence will be stored in the central pharmacy in a secure location. If consent is obtained, the investigator will enter an order for the study medication in the electronic medical record and take a copy of the completed consent form to the central pharmacy. Once the pharmacist receives the consent form, they will determine the group assignment from the randomization sequence and record the patient information on the randomization sheet. A copy of the informed consent will be stored in a binder with the randomization sheet.

Intervention

Patients will be randomized to receive heparin 25,000 units or 0.9% sodium chloride (placebo) via nebulizer every 6 hours while admitted to FHH. Treatment will begin after randomization and continue until patient is discharged from FHH.

Study Medication Process

The investigator will place an order in the electronic medical record for the study medication. The medication entry will be for:

- Med: INV-Heparin/Placebo 25,000 units/5 mL neb
- Route: IH
- SIG: QID-RC
- Start: Next scheduled time
- Dispense 5 doses at a time
- Label comments: **Do not give IV or Sub-Q**

Once the patient has been randomized, the pharmacist or designee will prepare a 24-hour supply of the group assigned medication to dispense as follows:

- **Placebo:** 5 mL of 0.9% sodium chloride drawn into a 5 mL syringe with the medication label and an auxiliary

	label that states "Not for injection" affixed.<br>• **Active:** 5 mL of heparin 5,000 units/mL drawn into a 5 mL syringe with the medication label and an auxiliary label that states "Not for injection" affixed.<br><br>The investigator will take the medication to the patients registered nurse, who will store the medication according to normal operating procedures for patient specific, room temperature medications. At this time a binder will be left with the patients chart that will contain investigator contact information, a copy of the approved IRB protocol, a form to document suspected adverse events. Subsequent doses will be prepared and delivered daily by pharmacy. The nurse will sign for all subsequent doses not delivered by the investigator. The signed delivery forms will be stored in the primary investigators locked cabinet.<br><br>The respiratory therapist will administer the medication as scheduled and record the administration in the medical record.<br><br>**End points and Data Collection**<br>The primary outcome for the meta-trial is the proportion of patients receiving invasive mechanical ventilation at day 28. At FHH, we plan to power a secondary endpoint to detect a difference in the average daily PaO2/FiO2 ratio as well. Hospital length of stay will also be compared.<br><br>The following information will be collected at baseline:<br>• Hospital admission date<br>• Unit of Admission<br>• Age<br>• Gender<br>• Ethnicity<br>• Body weight<br>• Medical history related to outcomes in COVID-19 and/or lung function<br>• Presence of infiltrates on chest X-ray<br>• Temperature<br>• Ferritin<br>• D-Dimer<br>• C-reactive protein<br><br>The following data will be collected daily until the patient is discharged or expires.<br>• Average FiO_2<br>• Average SpO_2<br>• Minimum and maximum peak inspiratory pressure<br>• Minimum and maximum plateau pressure<br>• Average positive end expiratory pressure<br>• Medications administered for COVID or pulmonary indications

	• Use of prone position<br>• Hemoglobin<br>• Platelets<br>• White blood cell count<br>• Maximum temperature<br>• Intake and output<br>• Occurrence of suspected adverse events such as blood tinged sputum, or bleeding<br><br>aPTT will be monitored at baseline to ensure eligibility and every 72 hours unless suspicion of coagulopathy. The cost of this monitoring will be covered by the department of pharmacy services.<br><br>Early stopping Criteria<br>We plan to perform frequent monitoring and analysis of the accumulating data, with use of Bayesian stopping rules that allow timely decisions without the penalties for multiple data looks and alpha spending associated with the classic RCT monitoring approach.[2,3,4] At each interim analysis (after 200 and 400 patients), the posterior distribution of the proportion of patients receiving invasive mechanical ventilation will be reported and the pre-specified stopping criteria will guide the recommendations of the meta-trial's executive committee<br><br>1. Li J, Pavlov I, Laffey JG, Roca O, Mirza S, Perez Y, et al. Meta-trial of awake prone positioning with nasal high flow therapy: Invitation to join a pandemic collaborative research effort. J Crit Care. 2020;60:140-2<br>2. Petkova E, Antman EM, Troxel AB. Pooling Data From Individual Clinical Trials in the COVID-19 Era. JAMA. 2020.<br>3. Lewis RJ, Angus DC. Time for Clinicians to Embrace Their Inner Bayesian?: Reanalysis of Results of a Clinical Trial of Extracorporeal Membrane Oxygenation. JAMA. 2018;320(21):2208-10.<br>4. Saville BR, Connor JT, Ayers GD, Alvarez J. The utility of Bayesian predictive probabilities for interim monitoring of clinical trials. Clin Trials. 2014;11(4):485-93.
9. What is the anticipated sample size and how was it determined?	There are two different sample size calculations involved in this research. First the overall sample size of the meta-trial and a sample size calculation for one of the secondary endpoints being completed only at FHH.<br><br>Sample size for **meta-trial primary endpoint**: Proportion of patients receiving invasive mechanical ventilation at day 28<br>• Assuming a baseline intubation rate of 35%, 656

	patients will be required to detect a 10% reduction in need for mechanical ventilation with 80% power and a two-sided significance level of 0.05.<br>• Based on discussion with trial coordinators, FHH will aim to contribute **50 patients** towards this sample size.<br><br>Sample size for **FHH secondary endpoint**: Average daily PaO_2 to FiO_2 ratio<br>• Based on previous research using nebulized heparin to improve oxygenation, 50 patients will be enrolled, 25 in each arm. This will provide 80% power to detect a 50-point difference in average daily PaO2 to FiO2 ratio with a two sided significance of 0.05.
10. How will participants be invited to participate (recruitment)?	Patients admitted to FHH will be screened by investigators on a daily basis for possible enrollment.
11. What are the inclusion and exclusion criteria?	Inclusion Criteria<br>• Age 18 years or older<br>• Currently admitted to FHH<br>• There is a PCR positive sample for SARS-CoV-2 within the past 21 days. The sample can be a nasal or pharyngeal swab, sputum, tracheal aspirate, bronchoalveolar lavage, or another sample from the patient<br>• Modified Ordinal Clinical Scale for COVID-19 Score of 3-5<br>Exclusion Criteria<br>• Enrolled in another clinical trial that is unapproved for co-enrolment<br>• Heparin allergy or heparin-induced thrombocytopenia (HIT)<br>• APTT > 120 seconds and this is not due to anticoagulant therapy<br>• Platelet count < 20 x 109 per L<br>• Pulmonary bleeding, which is frank bleeding in the trachea, bronchi or lungs with repeated hemoptysis or requiring repeated suctioning<br>• Uncontrolled bleeding<br>• Pregnant or might be pregnant. Females aged 18-49 years are excluded unless there is documented hysterectomy or a pregnancy test was performed and is negative.<br>• Myopathy, spinal cord injury, or nerve injury or disease with a likely prolonged incapacity to breathe independently e.g. Guillain-Barre syndrome<br>• Acute brain injury that may result in long-term disability<br>• Death is imminent or inevitable within 24 hours<br>• Treatment limitations in place, i.e. not for

	resuscitation, not for ICU admission, not for invasive mechanical ventilation<br>• Clinician objection<br>• Refusal of participant or their authorized healthcare agent consent<br>Note: Patients will be eligible for enrollment if they are receiving therapeutic or prophylactic anticoagulation. Participation in the study will not preclude them from being treated with prophylactic or therapeutic anticoagulation therapy. This investigation will not preclude the patient from receiving any additional therapies.
12. What is the duration of the entire study (dates)?	The planned enrollment period will begin upon IRB approval and is expected to take between 6 and 12 months. However, this may vary dramatically given the fluctuations in the number of cases and on-going vaccine administration.
13. Provide copies of any tools, instruments or surveys to be used.	N/A
14. Provide copies of all advertisements and recruitment materials.	N/A
**Human Subjects Protection**	
15. Will you be recruiting vulnerable populations? (see definitions at end)	No
16. What provisions are being taken to protect the privacy and ensure the confidentiality of research participants?	De-identified data will be stored on a password protected encrypted computer in a password protected file. An alpha-numeric code will be used to link the patients to the data and only the investigators will have access to the key. No patient identifiers will be disclosed on any potential publications.
17. Describe the plan to store and code the data.	Data will be collected using Microsoft access and excel. The patient identifiers will be kept in a separate password protected file and linked simply by a sequential study ID, e.g. Subject 1, Subject 2, etc
18. Describe the informed consent process if applicable (attach a copy)	Once a patient has been evaluated and is eligible, an investigator will call the patients decision maker in the presence of the patient's nurse or another party not involved with the present study and they shall serve as a witness to informed consent. They will both verbally confirm with the patient's decision maker that they wish to proceed with enrollment.
19. Describe measures that will be implemented to avoid participant coercion or undue influence.	
20. List each research-related risk for participants. Next to each, identify the likelihood of the risk and what will be done to minimize such risks	Heparin is an anticoagulant and carries a risk of bleeding. However, in previous studies, no reports of systemic or clinically significant anticoagulation occurred, suggesting a minimal risk of bleeding. In these studies, the only adverse event reported was blood tinged sputum. To minimize this risk, hemoglobin will be monitored daily, which is standard

	practice in the intensive care unit. Additionally, nurses will be asked to report any concerns or observations of suspected bleeding.<br>Heparin has also been linked to heparin induced thrombocytopenia. Platelets will also be monitored daily, which is also standard practice in the intensive care unit. If concerns develop for heparin induced thrombocytopenia, all heparin will be discontinued and laboratory work up for heparin induced thrombocytopenia will be sent. These patients will also be on DVT prophylaxis, so the risk for developing heparin induced thrombocytopenia is no greater than normal. No reports of heparin induced thrombocytopenia were reported from nebulized heparin.
21. Describe the potential benefit(s) to participants, or lack thereof.	Previous studies have demonstrated benefit in acute lung injury and this may be demonstrated in ALI from COVID-19. When initiated early, nebulized heparin may reduce the severity of inflammation and lung injury associated with COVID-19.
22. Describe how the potential risks to participants are reasonable in relationship to the potential benefits.	Given the lack of adverse reactions to nebulized heparin, there is minimal risk associated with this therapy. Previous studies have demonstrated benefit in acute lung injury and this may be demonstrated in ALI from COVID-19. This may lead to a reduced rate of intubation and COVID-19 related complications.
**Cost**	
23. Identify any costs associated with the study and the funding source.	The cost of the study medication will be approximately $5,500. This cost will be covered by the department of pharmacy as each dose will cost $1.76 and patients are not charged for medications that cost below $5.00. No other costs will be associated with this study as these patients are already connected to a nebulizer as part of their standard care. The projected cost of laboratory monitoring is $1,200 and will be covered by the pharmacy department. See attached budget for details. Additionally, there will be no charge for the investigators time, as this is a self-funded investigation.
24. Will participants receive payment (money, gift certificates, coupons etc.) for their participation in this study?	N/A
25. Will any one receive payment related to this study?	No
26. Submit a budget if applicable.	N/A
**Data Analysis**	
27. Provide a detailed description of how study data will be analyzed including statistical methods used.	Meta-trial statistical analysis<br>The statistical analysis plan for this research is attached as “appendix 1-Statistical analysis”. The data will be analyzed as part of an international meta-trial by a central statistician. Data will be de-identified prior to submission for final analysis to ensure confidentiality.

	FHH statistical analysis<br>Dichotomous data will be compared using a Chi^2 analysis, Continuous data will be analyzed using the student's T-test, time to hospital discharge will be evaluated with a survival analysis will be performed using a Kaplan-Meier estimate.
28. How will you ensure privacy, data storage and confidentiality?	De-identified data will be stored on a password protected encrypted computer in a password protected file. An alpha-numeric code will be used to link the patients to the data and only the investigators will have access to the key. Additionally, access to the files will be monitored.
29. How will you disseminate the results (internal, external, publish)?	Upon completion of data collection and analysis, the finding will be drafted into a manuscript and submitted for publication.

INFORMED CONSENT

1. **PROTOCOL NUMBER AND PROTOCOL TITLE:** INHALEd unfractionated HEParin for the treatment of hospitalized patients with COVID-19 (INHALE-HEP)

   This is a clinical trial (a type of research study). Clinical trials include only patients who choose to take part. Please take your time to make your decision. Discuss it with your friends and family. You are being asked to take part in this study because you have COVID-19.

2. **WHY IS THIS STUDY BEING DONE?**

   The purpose of this study is to determine if nebulized heparin can reduce the need for mechanical ventilation in patients with COVID-19. Additionally, it will determine if nebulized heparin can help your lungs absorb more oxygen if you have COVID-19.

3. **HOW MANY PEOPLE WILL TAKE PART IN THIS STUDY?**

   Approximately 50 people will take part in this study.

4. **WHAT IS INVOLVED IN THE STUDY?**

   Treatment Plan:

   You will be "randomized" into one of the study groups described below. Randomization means that you are put into a group by chance. It is like flipping a coin. A computer will determine in which group you will be placed. Neither you nor the researcher will choose what group you will be in. You will have an equal chance of being placed in either group. In addition to receiving the study medication, patients in both groups will have daily aPTT's monitored, which may result in an additional blood draw. Otherwise, all patients will continue to receive the same treatment as they would if not enrolled.

   Group 1: Nebulized Heparin **OR** **Group 2:** Placebo

5. **HOW LONG WILL I BE IN THE STUDY?**

   The study medication will be administered for the entire time you are in the hospital. If the study medication is stopped prior to hospital discharge, investigators will continue to monitor your progress until you are discharged from the hospital.

6. **WHAT ARE THE RISKS OF THE STUDY?**

   Likely: Blood tinged sputum

   Less Likely: Bleeding, reduced platelet count, abnormal blood tests, hemoptysis (coughing up blood), alveolar hemorrhage (significant bleeding in the lungs)

7. **ARE THERE BENEFITS TO TAKING PART IN THE STUDY?**

   The possible benefits of taking part in the study are the same as receiving either treatment without being in the study. We hope the information learned from this study will benefit other patients with COVID-19 acute lung injury in the future.

8. **WHAT OTHER OPTIONS ARE AVAILABLE?**

Please ask any questions you may have and take as much time as you need to make your decision. You may talk to your doctor about these and any other options available to you.

9. **WHAT ABOUT CONFIDENTIALITY?**
All data will be storied without patient identifying information. Only investigators will know who participates in the study and all disclosure of information will be in compliance with HIPPA. Any published results will not include identifying information.

10. **WHAT ARE THE COSTS?**
There will be no costs associated with this study.

11. **WHAT ARE MY RIGHTS AS A PARTICIPANT?**
Taking part in this study is voluntary. You may choose not to take part or may leave the study at any time. Leaving the study will not result in any penalty or loss of benefits to which you are entitled.

12. **WHO DO I CALL IF HAVE QUESTIONS OR PROBLEMS?**

For questions about the study or a research related injury, contact the researcher Thomas Smoot at 240-566-3250.

For questions about your rights as a research participant, contact the Frederick Health Hospital Institutional Review Board (which is a group of people who review the research to protect your rights) at
240-566-3513.

It may be necessary to contact you at a future date regarding new information about the treatment you have received. For this reason, we ask that you notify the institution where you received treatment on this study of any changes in address. If you move, please provide your new address to the following person:

Name: Thomas Smoot PharmD, BCPS Title: Primary Investigator

Address: 400 W. 7th street Frederick, MD 21701

Phone Number: 240-566-3520

You will get a copy of this form. You may also request a copy of the protocol (full study plan).

13. **SIGNATURE:**

I agree to take part in this study:

Participant ____ Date/Time____________________

Witness____Date/Time____________________

INVESTIGATOR PROTOCOL SIGNATURE PAGE

I Agree:

- To assure responsibility for the proper conduct of the clinical study at this site of which I am the principal investigator.
- To conduct the clinical trial in compliance with the study, any amendments, and any other study conduct procedures.
- Not to implement any deviations from or changes to the protocol without agreement from the sponsor and prior review and written approval from the Institutional Review Board (IRB), except where necessary to eliminate an immediate hazard to the participants, or for administrative aspects of the study (where permitted by all applicable regulatory requirements).
- That I am aware and/or will comply with “good clinical practices” (GCP) and all applicable regulatory requirements.
- To ensure that all persons assisting me with the study are adequately informed about the investigational drug(s) and of their study related duties and functions as described in the protocol.
- If the FDA has audited me for any study, or if there are any future changes involving the principal investigator or institution, I will notify the Institutional Review Board promptly.

____ Date ______________________

Investigator’s Name (Print)

____ Date ______________________

Investigator’s Signature

<table>
<tr><td colspan="3">Frederick Health Hospital
Financial Disclosure Form</td></tr>
<tr><td colspan="3">Instructions: Please complete all of the information below and retain a copy of this form for your records.</td></tr>
<tr><td colspan="3">1. Study Name: INHALEd unfractionated HEParin for the treatment of hospitalized patients with COVID-19 (INHALE-HEP)</td></tr>
<tr><td colspan="2">2. Protocol Number:</td><td>3. Investigator X 4. Sub-Investigator ↑</td></tr>
<tr><td colspan="3">4. Investigator/Sub-Investigator Name: Thomas Smoot</td></tr>
<tr><td colspan="3">5. Institution Name (if applicable): Frederick Health Hospital</td></tr>
<tr><td colspan="2">6. Address: 400 West 7th Street
Frederick, MD 21701</td><td>7. Telephone Number: 2405663250
8. Fax Number:</td></tr>
<tr><td>NONE: X↑</td><td colspan="2">I hereby certify that none of the financial interest or arrangements listed below exists for me, my spouse (legal partner), dependent children, or direct business partners.</td></tr>
<tr><td colspan="3">Indicate by marking the appropriate boxes below if any of the financial interests or arrangements of concern to FDA (and described fellow) apply to you, your spouse (legal partner), dependent children, or direct business partners.</td></tr>
<tr><td colspan="3">* Provide adequate information to allow an estimate of value, e.g. number of shares of stock, stock options, aggregate amount of payments or other consideration received for purposes <u>other than clinical study support,</u> patents, licenses, copyrights or trademarks or royalty interests.</td></tr>
<tr><td colspan="3">Yes ↑ Financial arrangements whereby the value of the compensation could be influences by the outcome of the study. For example, this could include compensation that is explicitly greater for a favorable outcome, or compensation to the investigator in the form of an equity interest in the sponsor or in the form of compensation tied to sales of the product, such as a royalty interest. If yes, please describe*:

No X↑</td></tr>
<tr><td colspan="3">Yes ↑ Significant payments of other sorts, <u>excluding the costs of conducting the study or other clinical studies</u>. For example, this could include payments made to the investigator or FMH to support activities that have an aggregate monetary value of greater than $25,000 (i.e. a grant to fund ongoing research, compensation in the form of equipment, or retainers for ongoing consultation or honoraria). If yes, please describe*:</td></tr>
</table>

No ↑X
Yes ↑ A proprietary or financial interest in the test product such as a patent, trademark, copyright, or licensing agreement. If yes, please describe*:<br><br>No ↑X
Yes ↑ A significant equity interest in the sponsor of the study. For example, this would include any ownership interest, stock options, or other financial interest whose value cannot be easily determined through reference to public prices, or an equity interest in a publicly traded company exceeding $50,000. If yes, please describe*:<br><br>No ↑X
In accordance with 21 CFR Parts 54.1 to 54.8, I declare the information provided on this form is, to the best of my knowledge and belief, true, correct, and complete. Furthermore, if my financial interest and arrangements, or those of my spouse (legal partner), direct business partner, or dependent children, change from the information provided above during the court of the study or within one year after the last patient has completed the study as specified in the protocol, I will notify the organization/hospital promptly.

9. Name (please print): Thomas Smoot<br><br>Signature:	10. Date:
